# Mechanochemical Conversion of Aromatic Amines to Aryl Trifluoromethyl Ethers

**DOI:** 10.1021/jacs.2c02611

**Published:** 2022-06-02

**Authors:** Michał Jakubczyk, Satenik Mkrtchyan, Mohanad Shkoor, Suneel Lanka, Šimon Budzák, Miroslav Iliaš, Marek Skoršepa, Viktor O. Iaroshenko

**Affiliations:** † Institute of Bioorganic Chemistry, Polish Academy of Sciences, Noskowskiego 12/14, Poznań 61-704, Poland; ‡ Laboratory of Homogeneous Catalysis and Molecular Design at the Center of Molecular and Macromolecular Studies, Polish Academy of Sciences, Sienkiewicza 112, Łodź PL-90-363, Poland; § Department of Chemistry and Earth Sciences, Qatar University, P.O. Box 2713, Doha, Qatar; ∥ Lodz University of Technology, Stefana Żeromskiego 116, Lodz 90-924, Poland; ⊥ Department of Chemistry, Faculty of Natural Sciences, Matej Bel University, Tajovského 40, Banská Bystrica 97401, Slovakia; # Department of Chemistry, University of Helsinki, A.I. Virtasen aukio 1, Helsinki 00014, Finland

## Abstract

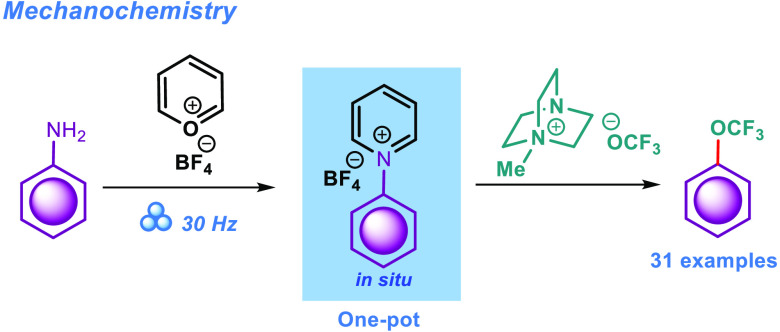

Increased interest in the trifluoromethoxy group in organic synthesis and medicinal chemistry has induced a demand for new, selective, general, and faster methods applicable to natural products and highly functionalized compounds at a later stage of hit-to-lead campaigns. Applying pyrylium tetrafluoroborate, we have developed a mechanochemical protocol to selectively substitute the aromatic amino group with the OCF_3_ functionality. The scope of our method includes 31 examples of ring-substituted anilines, including amides and sulfonamides. Expected S_N_Ar products were obtained in excellent yields. The presented concise method opens a pathway to new chemical spaces for the pharmaceutical industry.

## 1 Introduction

Introducing fluorine and fluorine-containing substituents into the structures of biologically active molecules is a standard strategy in drug design to modify properties such as acidity or basicity (which influences binding affinity, pharmacokinetics, and bioavailability), lipophilicity, steric properties, conformational constraint and metabolic stability.^1−4^ According to recent estimates, 20% of prescribed or clinically administered pharmaceuticals contain at least one fluorine atom. Moreover, in general, 30–50% of the most profitable drugs (depending on the sales period) contain fluorine.^5,6^

Among all the fluorine-containing substituents, the trifluoromethoxy group (OCF_3_) is the least-investigated and least-understood moiety. However, this “exotic” entity has attracted more and more attention,^7^ partially due to its specific features. Apart from the high electronegativity^8^ and excellent lipophilicity,^9^ in aryl trifluoromethyl ethers, the OCF_3_ moiety adopts an orthogonal orientation relative to the aromatic ring.^10,11^ In contrast to CH_3_, this group is not conjugated to the aromatic ring because the oxygen p-electrons are delocalized in the σ*-orbitals of the C–F bonds.^12^

In addition to biologically active molecules in medicine and agrochemicals, the OCF_3_ group can be found in compounds with applications as electro-optical materials.^13,14^ Moreover, in recent years, significant progress has been made in C–F bond activation strategies.^15−20^ Selective and efficient monodefluorination in (hetero)aryl di- and trifluoromethyl ethers is possible with frustrated Lewis pair (FLP) chemistry.^21,22^ This methodology was also shown to be valid for Ar-OCF_3_ ethers, opening new synthetic possibilities.^23^ Particularly, the monosubstitution of fluorine in OCF_3_ by a variety of nucleophiles can provide diversely substituted derivatives (including handles for further reactions) or chain elongation protocols. A transformation sequence that provides an efficient OCF_3_ introduction method in combination with C–F activation can give access to unexplored chemical space based on the −OCF_2_– connection. Additionally, such protocols could be potentially realized in a one-pot fashion if only they were independent of each other’s reactants and byproducts. Due to the large interest in the introduction of the difluoromethylenoxy moiety, -OCF_2_- intermediates are already finding applications as reagents, for example, in oxidative C–H aryloxydifluoromethylation with α,α-difluorophenoxyacetic acids.^24^

**Scheme 1:**
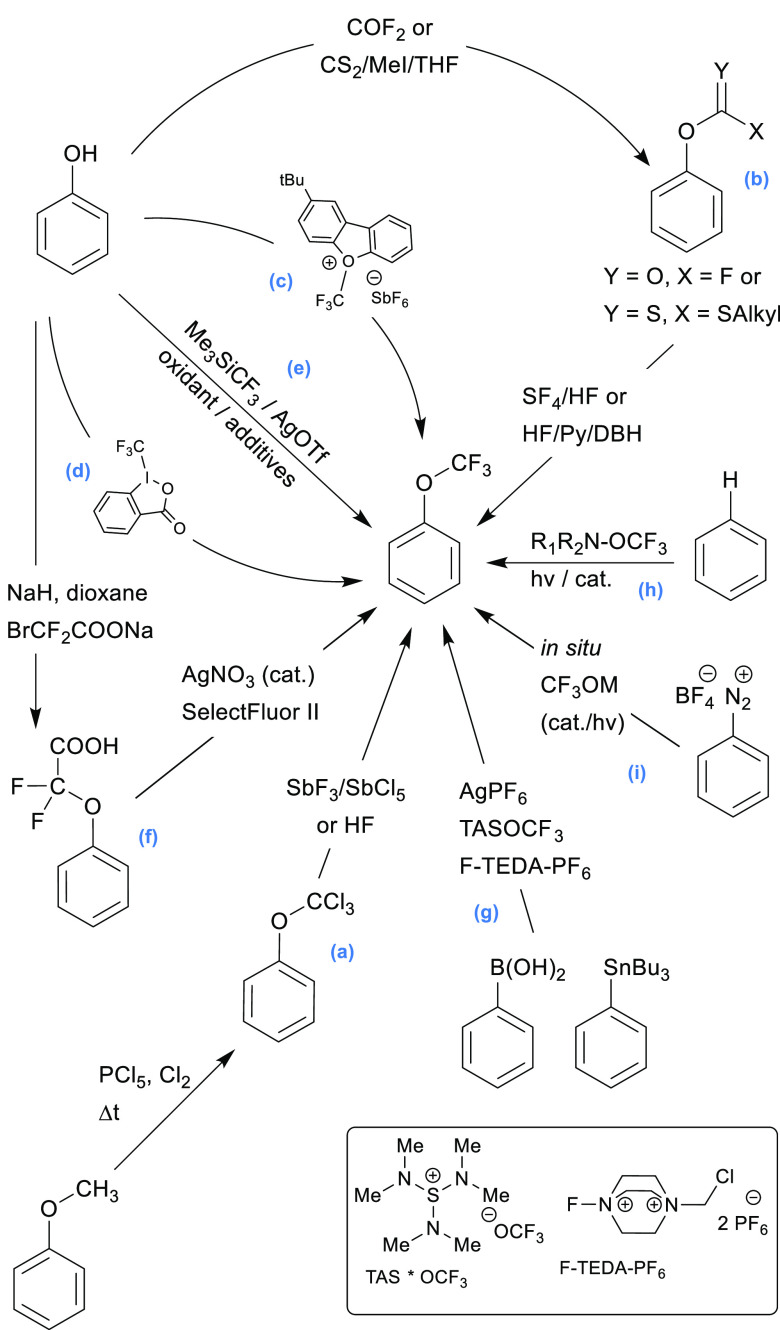
Known-to-Date Ar-OCF_3_ Ether Formation Methods

According to known methods, the synthesis of the aryl trifluoromethyl ethers can proceed in several ways, assuming the formation of a C**–**F, C–OCF_3_, or O**–**CF_3_ bond or a combination thereof. Chronologically, an aryl–OCF_3_ ether was obtained for the first time by Yagupolskii in 1955.^25^ In the first step of this method, the substituted anisole derivatives are converted to trichloromethyl intermediates, which are then submitted to a halogen exchange step (Scheme 1a). Similarly, the whole sequence can be realized in one-pot by heating phenols as starting materials in a CCl_4_/anhydrous HF mixture in a pressure vessel with BF_3_ as a catalyst.^26^ The trichloromethyl intermediate can also be obtained from chlorothionoformates,^27^ however, the high toxicities of those reagents limit their use substantially. The CF_3_ group can also be “constructed” on the phenol oxygen *via* nucleophilic fluorination using aryl fluoroformates^28,29^ or dithiocarbonates as intermediates (Scheme 1b).^30−33^ Unfortunately, these methods have limited scopes, require harsh conditions, and often suffer from low yields, precluding their use in any late-stage modifications of drug candidates. More versatile and useful methods for the direct formation of the ArO–CF_3_ bond rely on reagents that deliver the complete CF_3_ synthon as an electrophile, such as Umemoto’s oxonium reagents^34^ and Togni’s benziodoxolon reagent II^35^ (Scheme 1c and d, respectively), or proceed *via* silver-mediated oxidative trifluoromethylation with the Ruppert-Prakash reagent^36^ (Scheme 1e), which is formally a source of the nucleophilic CF_3_ synthon. Additionally, a two-step procedure catalyzed by silver salts was devised by combining *O*-carboxydifluoromethylation with subsequent decarboxylative fluorination with SelectFluor II (Scheme 1f).^37,38^

In the case of OCF_3_ transfer agents, the radical trifluoromethoxylation of arenes with trifluoromethyl-hypofluorite^39^ and the nucleophilic reaction of various trifluoromethoxylate salts with arynes^40^ offer low selectivities and limited scopes. The two-step OCF_3_ migration method is limited to *N*-aryl-*N*-hydroxyl amines that react with Togni’s reagent II to give *O*-trifluoromethylated adducts, which subsequently undergo thermal-induced migration.^41^ The direct silver-mediated trifluoromethoxylation of aryl precursors (boronic acids and stannanes) accepts a variety of starting materials and offers good yields (Scheme 1g).^42^ Additionally, the C–H trifluoromethoxylation of arenes using a transition-metal redox-active catalyst and a •OCF_3_ radical-generating photoactivated reagent (R_1_R_2_N–OCF_3_) was described (Scheme 1h).^43,44^ Later, in 2022, Qing and co-workers developed the C–H trifluoromethoxylation of arenes by combining trifluoromethyl 2-pyridyl sulfone with oxygen as a convenient trifluoromethyl source utilizing a unique electrochemical protocol with a graphite anode and a platinum cathode.^45^ A very interesting approach was proposed that used (hetero)aryldiazonium tetrafluoroborate salts as starting materials (Scheme 1i). Those methods use various trifluoromethyl alkyl- and arylsulfonates (R–SO_2_–OCF_3_) to generate the CF_3_OM salt *in situ*.^46−49^ Furthermore, very recently, two other papers worth mentioning were published. Togni and co-workers communicated the straightforward trifluoromethoxylation of aromatic substrates using the bench-stable pyridinium-based trifluoromethoxylation reagent *via* the non-directed functionalization of C–H bonds utilizing Ru(II)- and Ru(III)-mediated photoredox catalysis.^50^ This process involves the formation of OCF_3_ radicals. The work by Hu describes an original concept for the nucleophilic trifluoromethoxylation of alkyl (pseudo)halides and cross-coupling with aryl stannanes, where trifluoromethyl benzoate is used as an efficient and readily available trifluoromethoxylation reagent.^51^

In contrast to the diazonium salts, which are temperature-unstable, shock-sensitive, explosive, and require the use of strong acids and oxidants for generation, the pyridinium salts can be generated *via* condensation with pyrylium salts (Pyry-BF_4_) under relatively mild conditions in ethanol and used *in situ* in the nucleophilic aromatic substitution.^52,53^ The pyrylium tetrafluoroborate reagent can be prepared in large quantities and safely stored for long periods. Moreover, Pyry-BF_4_ selectively activates amino groups in synthetic and natural aminoheterocycles and therefore can also be used in the late-stage modification of drugs and drug candidates.

## 2 Results and Discussion

Inspired by the recent successful development of a deaminative chlorination protocol for aminoheterocycles that used Pyry-BF_4_,^54^ we envisioned that a similar method could be used to introduce the trifluoromethoxy group. In the current paper, we present a new methodology that enables the efficient installation of the OCF_3_ functionality onto aromatic substrates through the conversion of the NH_2_ group using a readily available and commercialized pyrylium tetrafluoroborate reagent (Pyry-BF_4_).

The activation of the C(sp^2^)–NH_2_ bond is complicated due to its low nucleophilicity. However, condensation with the pyrylium reagent (Pyry-BF_4_) gives pyridinium salts as intermediates in good yields for use in aromatic substitution reactions (S_N_Ar).^52^ The reaction of those salts with a variety of nucleophiles results in C–O, C–N, C–S, and C–SO_2_R bond formation. Those two steps can be realized in one-pot without isolation of the pyridinium salt. The simplicity and generality of this strategy allow for the selective functionalization of aromatic NH_2_ groups. Moreover, the Pyry-BF_4_ reagent is easy to prepare, stable and nowadays commercially available. We envisioned a similar substitution of pyridinium salts with ^⊖^OCF_3_.

Our first attempts to realize the title transformation were unsuccessful. The utilization of an isolated pyridinium salt, 1-(4-(methylsulfonyl)phenyl)pyridin-1-ium tetrafluoroborate, as well as one-pot approach starting from (methylsulfonyl)aniline **1e** in different solvents (DMF, DMA, dichloromethane, MeOH and acetonitrile) under a range of temperatures (also under reflux) did not result in the formation of the desired substitution product (for more details, see Table S1, entries 13–31 in the SI). Of note, only 1,4-dioxane showed promising results (Table S1, entries 18, 19), as the model compound was isolated in 8% and 32% yields, respectively.

On the basis of our previous experiences and the literature on mechanochemical realizations of S_N_Ar reactions,^55−59^ and as part of our general policy to search for green methodologies, we conducted a routine experiment under mechanochemical conditions (Table S1, entry 1) in a one-pot fashion, starting from the same substrate **1e**. To our content, the evident presence of the target product in the reaction mixture was demonstrated by TLC and confirmed after separation (12% yield). To the best of our knowledge, there are no other successful examples or attempts to realize such S_N_Ar pyridinium salt substitution with the ^⊖^OCF_3_ donor in the literature, neither in solution nor in the solid state. Encouraged, we conducted an optimization of the mechanochemical reaction conditions by adjusting the reagent equivalents and the ^⊖^OCF_3_ source (see Table S1, entries 1–12). Since some of the starting materials are liquids at r.t. and a molar equivalent of water is produced in the first step of the process (Scheme 4), we applied a grinding auxiliary material to both improve mixing and energy transfer and prevent the reaction mass from forming a gum or paste. Among the following oxides, ZrO_2_ gave the best yield (19%, 27%, 28%, and 34%, respectively; Table S1, entries 2–5): CeO_2_, TiO_2_, Yb_2_O_3_ and ZrO_2_.

**Scheme 2:**
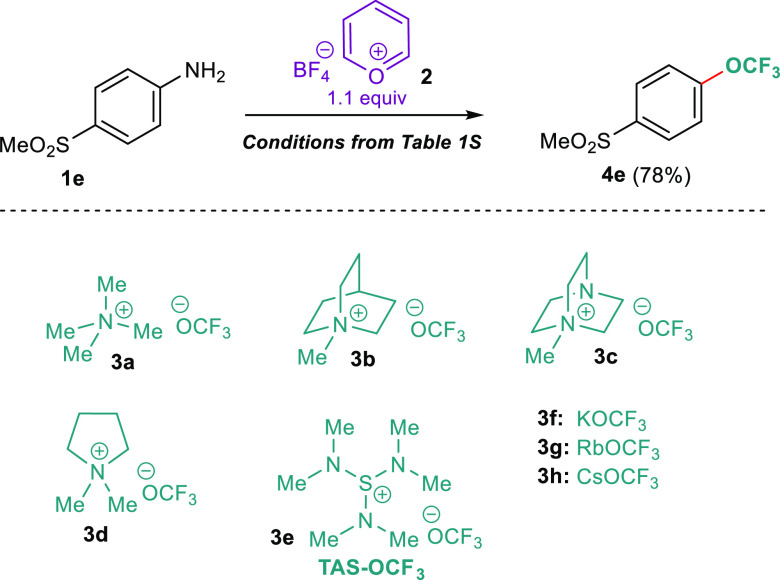
Optimization of the Reaction Conditions

The applied ^⊖^OCF_3_ sources included tetramethylammonium trifluoromethanolate (**3a**), 1-methyl-quinuclidin-1-ium trifluoromethanolate (**3b**), 1-methyl-1,4-diaza-bicyclo[2.2.2]octan-1-ium trifluoromethanolate (**3c**), 1,1-dimethyl-pyrrolidin-1-ium trifluoromethanolate (**3d**), tris(dimethylamino)sulfonium trifluoro-methanolate (**3e**), potassium trifluoromethanolate (**3f**), rubidium trifluoromethanolate (**3g**), and cesium trifluoromethanolate (**3h**) (Scheme 2). The best conditions, comprising **1e** (1.0 equiv), Pyry-BF_4_ (1.1 equiv), ZrO_2_ (1.0 equiv), and **3c** (1.5 equiv) ground at 30 Hz for 90 min, gave a 78% yield (Table S1, entry 7). Of note, the superiority of ZrO_2_ within this protocol might be explained by the stabilizing effect it has on the parental OCF_3_ anion, most likely as a result of the interaction of Zr with both the oxygen and fluorine atoms. Four-coordinate Zr has good affinity to aliphatic C–F bonds. It is also known that differences in the structures of the inorganic materials result in different friction energies.

**Scheme 3:**
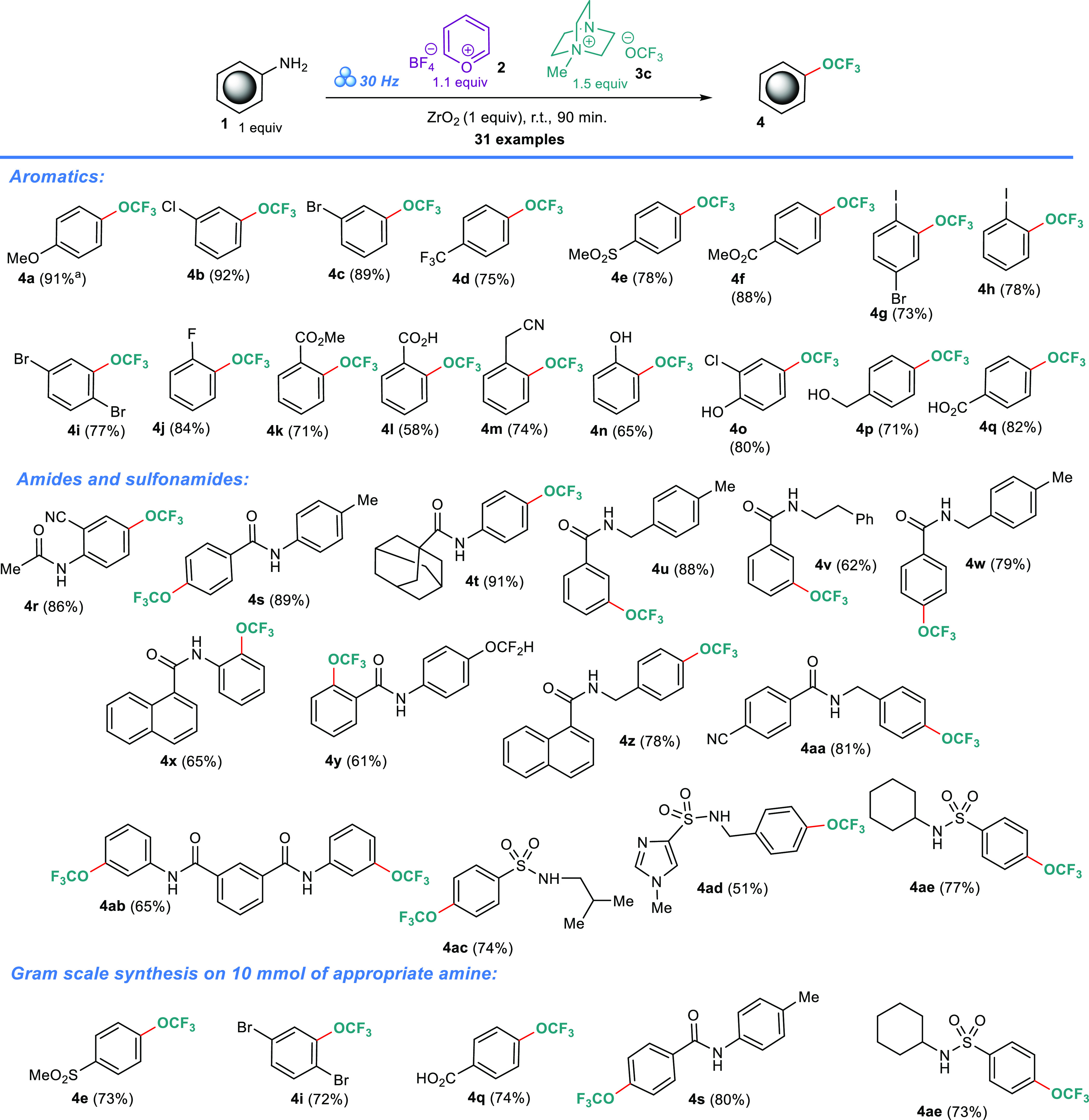
Reaction Scope

With the optimized conditions in hand, we embarked on an assessment of the method’s scope. Several substituted anilines were subjected to the protocol, yielding OCF_3_-substituted products **4a**–**4q** in 58–92% isolated yields (Scheme 3). The two-step one-pot mechanochemical protocol makes impossible any inquires on the efficiency of consecutive steps without the isolation of every single intermediate pyridinium salt. However, conclusions from the optimization and the literature on pyridinium salt generation and stability^52^ suggest that the first formal step (the generation of the pyridinium salt) is not the limiting one. On the other hand, one must remember that solid-state reaction kinetics is governed by entirely different rules than in-solution processes.^60−63^ Evidently, lower yields (Scheme 3) were observed for *ortho*-substituted starting materials **4l** (58%), **4n** (65%), **4x** (65%), **4g** (73%), **4h** (78%), **4i** (77%), etc. This could be explained by the bulkiness of the adjacent *ortho*-substituent and the hydrogen bond donor or acceptor properties of the COOH, OH, and C(O)NHR groups, which could interfere with all steps that lead to the desired product. The developed methodology is also feasible on the gram scale; thus, compounds **4e** (73%), **4i** (72%), **4q** (74%), **4s** (80%), and **4ae** (73%) were prepared in acceptable yields.

**Scheme 4:**
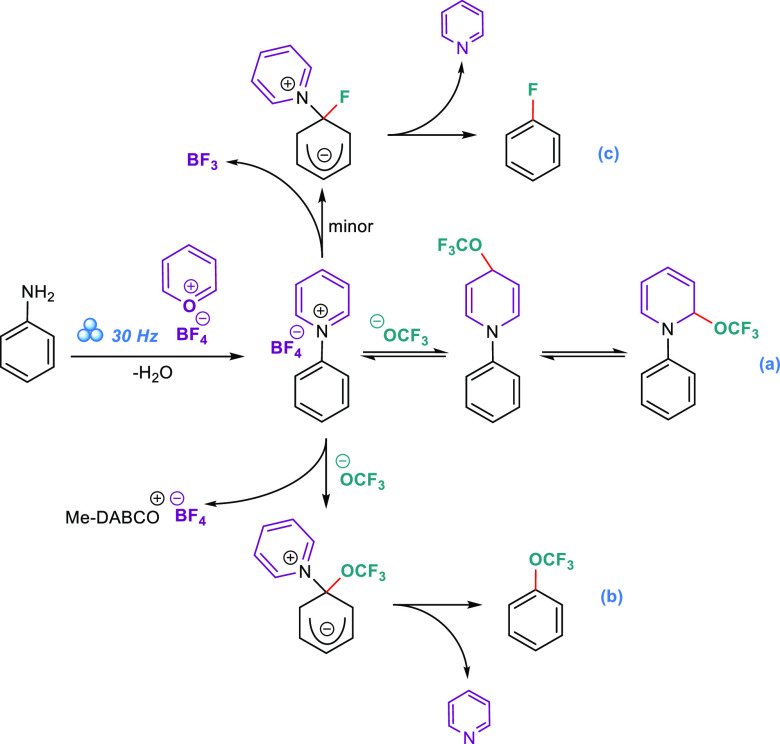
Proposed Mechanism of the Second Step

**Scheme 5:**
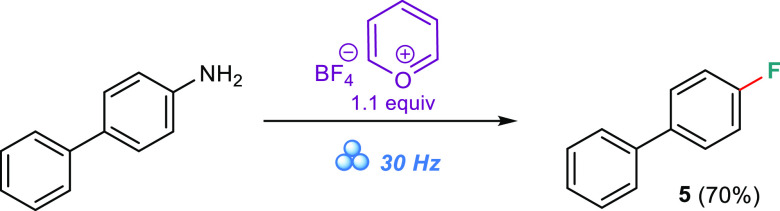
Test Reaction Excluding the ^⊖^OCF_3_ Source

In fact, the higher yield for the *ortho*-COOMe derivative **4k** (71%) compared to that of **4l** could suggest that intramolecular hydrogen bond formation stabilizes the starting material, interfering with the first step (Scheme 4, pyridinium salt formation). In contrast, the bulkiness makes the nucleophile approach difficult in the irreversible second step, leading to a quaternary carbon product (Scheme 4b). The nucleophilic attack of ^⊖^OCF_3_ on the 2- and 4-positions of the pyridinium ring is reversible, thus those equilibria are not responsible for diminishing the overall process efficacy significantly. However, upon analysis of the TLC and NMR profiles of the crude mixtures, a byproduct of the same type was observed as a trace and identified as the F substitution product. To explain the occurrence of this product, we performed a simple test reaction under mechanochemical conditions starting from [1,1′-biphenyl]-4-amine, without adding the trifluoromethoxyl anion source **3c** (Scheme 5). The TLC control revealed the presence of the byproduct after 1 h, which was accompanied by almost complete conversion of the starting material (to the pyridinium salt). After the reaction proceeded for a prolonged time (6 h), a 4-fluoro-1,1′-biphenyl 5 was isolated in a 70% yield, as evidenced by the NMR spectra of the purified sample (SI, spectral data for compound **5**). This concurrent reaction can be explained by the slow process of including ^⊖^BF_4_ as the fluoride nucleophile source (Scheme 4c), which possibly enters some equilibria or undergoes dimerization in the presence of water^64−66^

Even taking the above explained interference into account, our mechanochemical protocol gives good results for anilines with simple, small substituents, as well as for starting materials with amide (**4r–4ab**) and sulfonamide (**4ac–4ae**) connections. In the case of **4ab**, a 74% yield is quite high considering that four reaction events are required to produce this double-substitution product. Notably, we also tried several aminoheterocycles, such as 2-aminopyridine, benzo[*d*]thiazol-2-amine, etc. To our great disappointment, the title reaction experienced a failure, and we did not observe the formation of the desired OCF_3_ products.

**Figure 1:**
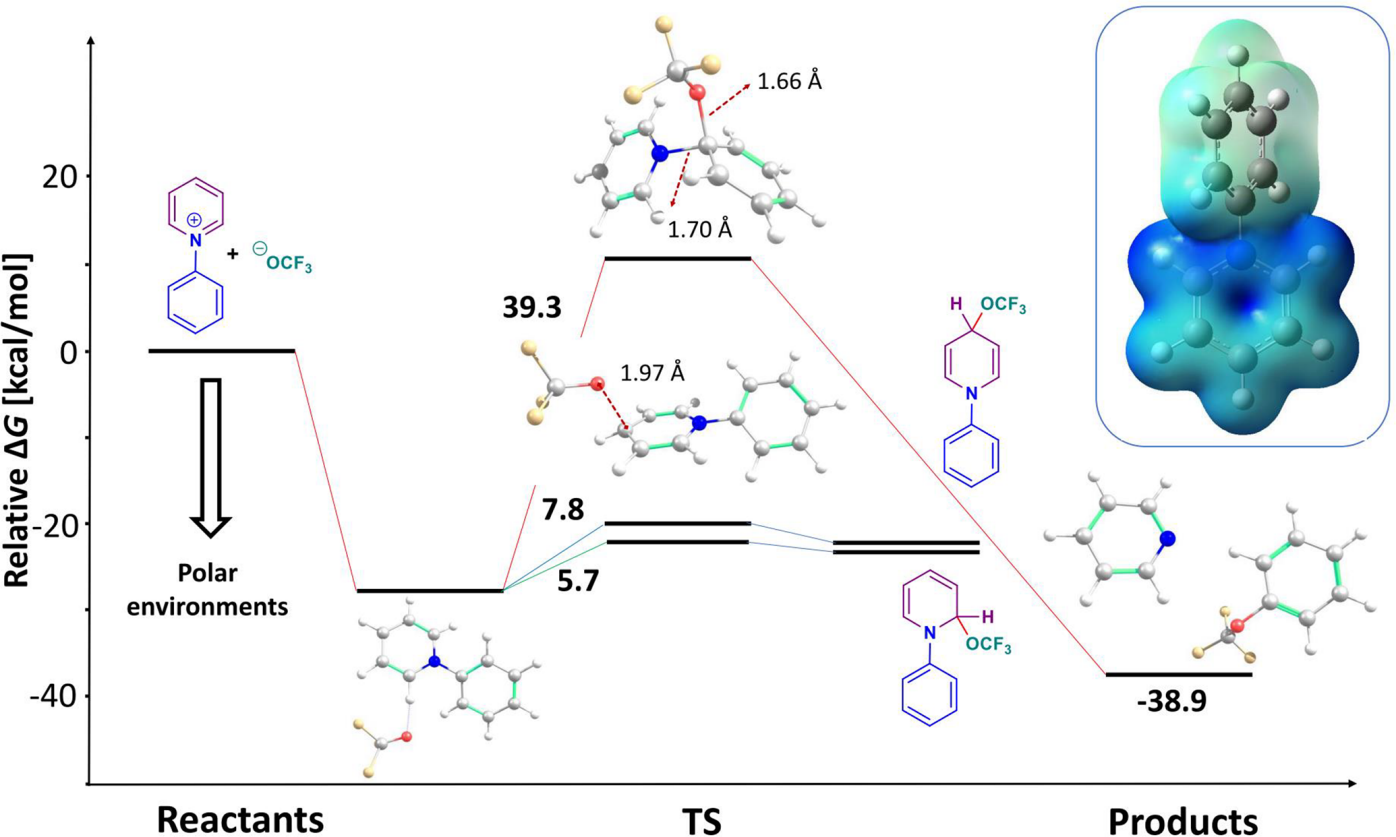
Energy profile diagram. All energies (kcal/mol) are from isolated reactants at a 0 kcal/mol reference. The barriers were calculated for 298 K.

To elucidate the mechanism of the synthesis, we performed computational modeling (Figure 1). Since the first step of the reaction mechanism is clearly established in the literature, we focused on the second step, namely the substitution and dissociation of the pyridium salts into respective products. We studied two possible mechanistic routes: (i) single electron transfer (SET) followed by radical formation and (ii) the direct nucleophilic attack of the ^⊖^OCF_3_ anion.

The adiabatic (including molecular relaxation) electron affinity of unsubstituted pyridinium salt was calculated as 4.86 eV at the CCSD(T)/def2-TZVPP level of theory in the gas phase. From the species in the reaction mixture, the ^⊖^OCF_3_ anion has a sufficiently low ionization potential of 4.21 eV in the gas phase, allowing SET. This situation is reversed by effects of the environment. Toluene as nonpolar solvent stabilizes the small ^⊖^OCF_3_ anion to the extent that its ionization potential becomes larger than the electron affinity of the larger pyridinium salt, minimizing the possibility of SET in the ground state of the system. Several authors studied the behavior of pyridinium salts following SET. Lorance *et al*. found that *N*-methoxypyridinium salts dissociate with minimal activation energy upon SET, giving rise to pyridine and a methoxy radical.^67,68^ We tested our methodology on these systems and consistently found either none or a minimal barrier for methoxy radical formation upon SET, in agreement with the mentioned experiment. When we approached systems studied here with the identical methodology, we observed a barrier of 35 kcal/mol that hindered the formation of the phenyl radical as a possible reactive intermediate. Moreover, the dissociation of the *N*-phenylpyridium radical into pyridine and the phenyl radical is also thermodynamically disfavored (Δ*G*_r_ = 18.1 kcal/mol). This is in line with results of Sevov *et al.*, who used structurally comparable pyridinium salts as anolytes to sustain many cycles of charging and discharging.^69^

Next, we turned our attention to the possible nucleophilic attack of ^⊖^OCF_3_ on the formed pyridinium salt. All possible substitution positions were considered. From a thermochemical viewpoint, nucleophilic attacks on carbons that already had hydrogens led to metastable intermediates relatively high in energy in case of the *ortho*- and *para*-positions (Δ*G* ≈ 5 kcal/mol) on pyridine and the *para*-position on phenyl (Δ*G* ≈ 14 kcal/mol). Such intermediates are protected from disintegration by a very small barrier less than 2 kcal/mol and require another particle, which would leave with hydrogen or proton to stabilize into the products. For other positions, it was impossible to stabilize nucleophilic substitution intermediate, with one notable exception that led to the observed products.

The nucleophilic attack on a carbon bonded to the pyridine nitrogen provides a channel to the stable products without any intermediate, and pyridine and the corresponding OCF_3_ derivative are generated directly. The reaction Δ*G* favors such a splitting by −10 kcal/mol compared to the reactants. Not only the ^⊖^OCF_3_ anion is capable of achieving such a dissociative substitution. Calculations using the fluorine anion corroborate the experimental finding that fluorinated products can be reached in the absence of an OCF_3_ source.

To gain more insight, we tried to replicate the non-reactive behavior of pyridinium salt (leading to **4e**) toward the ^⊖^OCF_3_ anion, which was observed for a range of different solvents and temperatures. In polar solvents (ACN and MeOH), separate ionic reactants tend to be over-stabilized compared to the transition state, and the resulting activation Δ*G*‡ reaches 37.6 kcal/mol, according to our calculations. Nonpolar solvents such as 1,4-dioxane seem to be the rational choice to overcome this issue. However, in dioxane, the hydrogen-bonded F_3_CO^⊖^···H–pyridinium complex stabilizes reactants, and the activation Δ*G*‡ reaches a value of 29.1 kcal/mol, which is still too high for a straightforward reaction. This was proven by the corresponding experiments in solution (Table S1, entries 18 and 19). The mechanochemical setup allows this reaction to proceed in a high yield in a short time. It is difficult to establish the exact reasons behind this. As an effect of the higher concentration, more frequent collisions^70^ of reactants can increase the prefactor in the Eyring equation, leading to effective acceleration.

It has been shown that concentration effects alone may not be sufficient to explain the observed changes in kinetics.^71^ The effects of unoriented mechanical forces experienced by the system between the walls of the grinding balls can modify the transition-state position and lower the activation energy. Here we note that the imaginary vibration in the transition state has a value of 242 cm^–1^, while the intermolecular vibrations of reactants mostly have values under 100 cm^–1^, meaning that relatively low force constants and thus external forces can significantly divert the system from the equilibrium position.

## 3 Conclusion

In summary, we have developed a mechanochemical one-pot procedure for the selective and highly efficient substitution of an aromatic amine group with an OCF_3_ substituent *via* the pyridinium salt intermediate. 1-Methyl-1,4-diazabicyclo[2.2.2]octan-1-ium trifluoromethoxide salt was selected as the best ^⊖^OCF_3_ nucleophile source. Our method accepts a variety of functionalities that can act as entry points for further transformations (including Br, I, and COOH groups) as well as moieties such as amide and sulfonamide. The lower yields for *ortho*-substituted starting materials point to steric hindrance as a limiting factor. Surprisingly, the developed procedure works only in the solid state; further studies must be conducted to explain this behavior and possibly harness its benefits in other synthetic applications. Nevertheless, the mechanochemical conditions ensure a mild temperature, a reduced workup time and solvent economy, corresponding to the principles of green chemistry. The generality of our protocol will be confirmed by further studies; however, the selectivity, efficiency and robustness of the presented method will indeed have an impact on medicinal chemistry in the near future considering the significance of the OCF_3_ substituent and the -OCF_2_- linkage as pharmacophores.

## Supporting Information Available

General information; Cartesian coordinates; HRMS and MG MS data; spectral data; and copies of ^1^H, ^19^F{1H} and ^13^C{1H} NMR spectra (PDF)
