# Supplementary material for: Mechanochemical Conversion of Aromatic Amines to Aryl Trifluoromethyl Ethers
Source: J Am Chem Soc. 2022 Jun 2;144(23):10438–45. doi: 10.1021/jacs.2c02611 (PMC9204773; doi:10.1021/jacs.2c02611)
Supplement: Supplementary file 1 — ja2c02611_si_001.pdf [file ja2c02611_si_001.pdf]

Mechanochemical conversion of aromatic amines into aryl trifluoromethyl ethers.

Michał Jakubczyk[a], Satenik Mkrtchyan[b]*, Mohanad Shkoor[c], Suneel Lanka[d], Simon Budzak[e], Miroslav Iliaš[e], Marek Skorsepa[e], Viktor O. Iaroshenko[e,f]*.

[a] Institute of Bioorganic Chemistry, Polish Academy of Sciences, Noskowskiego 12/14, 61-704 Poznań (Poland).
[b] Laboratory of Homogeneous Catalysis and Molecular Design at the Center of Molecular and Macromolecular Studies, Polish Academy of Sciences, Sienkiewicza 112, PL-90-363 Łodź (Poland).
[c] Department of Chemistry and Earth Sciences, Qatar University, P.O. Box 2713, Doha (Qatar).
[d] Lodz University of Technology, Żeromskiego str. 116, 90-924 Lodz (Poland).
[e] Department of Chemistry, Faculty of Natural Sciences, Matej Bel University, Tajovkého 40, 97401 Banska Bystrica, Slovakia. https://www.fpv.umb.sk/viaroshenko/ E-mail: viktor.iaroshenko@umb.sk
[f] Department of Chemistry, University of Helsinki, A.I. Virtasen aukio 1, 00014 Helsinki, (Finland). https://researchportal.helsinki.fi/en/persons/viktor-iaroshenko E-mail: iva108@gmail.com

Table of Contents

(A) Experimental Section S3
(B) Characterization of products S7
(C) Copies ^{1}H and ^{13}C NMR spectra S29
(D) Mass Spectra S94
(E) Computational study S136

(A) Experimental Section.

Commercially available starting materials, reagents, catalysts, anhydrous and degassed solvents were used without further purification. Flash column chromatography was performed with Merck Silica gel 60 (230-400 mesh). The solvents for column chromatography were distilled before the use. Thin layer chromatography was carried out using Merck TLC Silica gel 60 F_{254} and visualized by short-wavelength ultraviolet light or by treatment with potassium permanganate ($KMnO_4$) stain. ^{1}H, ^{13}C and ^{19}F NMR spectra were recorded on a Bruker 250, 400 and 500 MHz at 20ºC. All ^{1}H NMR spectra are reported in parts per million (ppm) downfield of TMS and were measured relative to the signals for $CHCl_3$ (7.26 ppm) and DMSO (2.50 ppm). All $^{13}C\{^{1}H\}$ NMR spectra were reported in ppm relative to residual $CHCl_3$ (77.00 ppm) or DMSO (39.70 ppm) and were obtained with ^{1}H decoupling. Coupling constants, *J*, are reported in Hertz (Hz). Gas chromatographic analyses was performed on Gas Chromatograph Mass Spectrometer GCMS-QP2010 Ultra instrument. Mechanochemical synthesis was performed using the Retsch MM400 mill using the standard kit. Liquid chemicals were dosed using gas tight micro syringes. Isolation of obtained compounds was achieved by column chromatography on Silica gel. All commercially available compounds were purchased from appropriate vendors. Compounds **3a-3h** were prepared according to the literature described procedure.[1-5]

Literature:

1. Newton, J. J.; Jelier, B. J.; Meanwell, M.; Martin, R. E.; Britton, R.; Friesen, C, M. Quaternary Ammonium Trifluoromethoxide Salts as Stable Sources of Nucleophilic OCF_3. *Org. Lett.* **2020**, *22*, 1785–1790.
2. Huang, C.; Liang, T.; Harada, S.; Lee, E.; Ritter, T. Silver-Mediated Trifluoromethoxylation of Aryl Stannanes and Arylboronic Acids. *J. Am. Chem. Soc.,* **2011**, *133*, 13308–13310.
3. Redwood, M. E.; Willis, C. J. *Can. J. Chem.* **1965**, *43*, 1893–1898.
4. Kolomeitsev, A. A.; Vorobyev, M.; Gillandt, H. Versatile application of trifluoromethyl triflate. *Tetrahedron Lett.*, **2008**, *49*, 449–454.
5. Qi, X.; Chen, P.; Liu, G. Catalytic Oxidative Trifluoromethoxylation of Allylic C-H Bonds Using a Palladium Catalyst. *Angew. Chemie - Int. Ed.* **2017**, *56*, 9517–9521.

A-1. Scope of the reagents used.

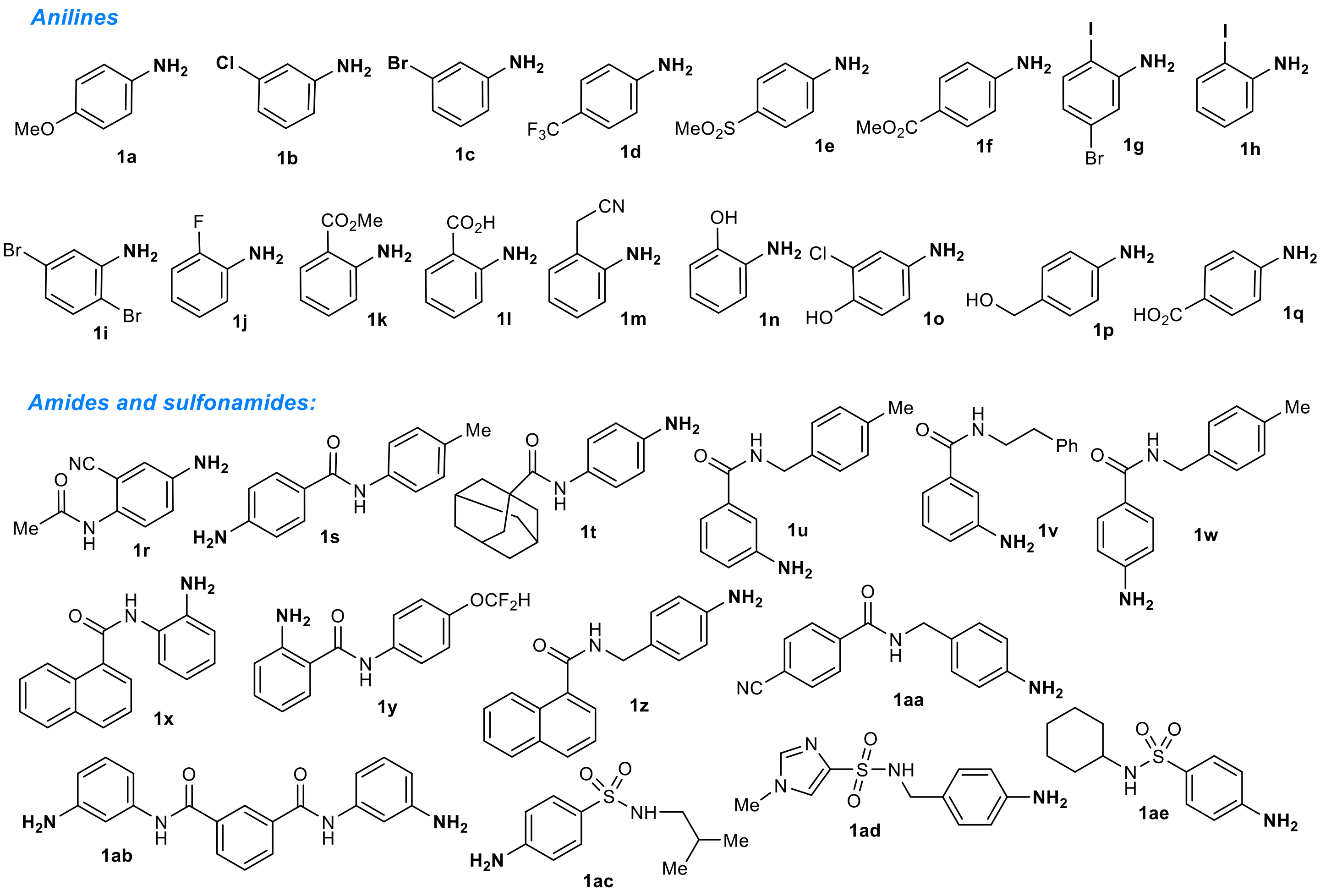

Scheme 1. List of starting anilines.

A-2. Reaction condition screening.

Table S1: Optimization of the reaction conditions.

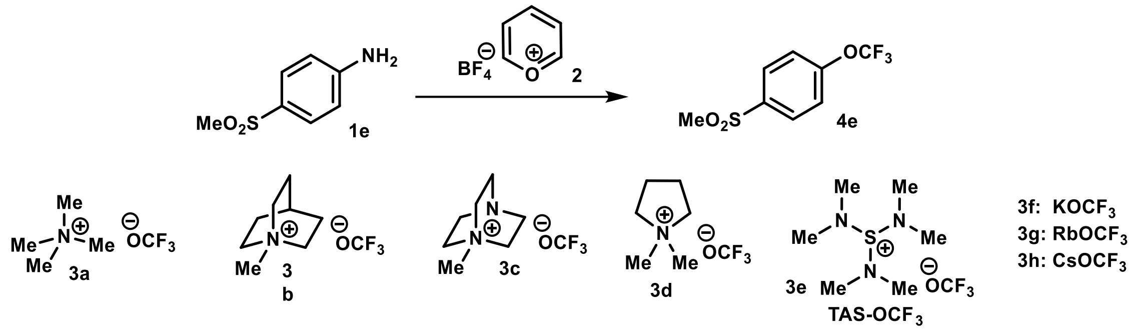

entry	reaction components	milling frequency/time	yield (%) **4e**
	**Reactions in the solid phase**		
1	4-(methylsulfonyl)aniline (1.0 equiv.), **2** (1.3 equiv.), **3a** (2.2 equiv.).	30Hz/90min	12
2	4-(methylsulfonyl)aniline (1.0 equiv.), **2** (1.1 equiv.), CeO_2 (1.0 equiv.), **3a** (1.5 equiv.)	30Hz/90min	19
3	4-(methylsulfonyl)aniline (1.0 equiv.), **2** (1.1 equiv.), TiO_2 (1.0 equiv.), **3a** (1.5 equiv.)	30Hz/90min	27
4	4-(methylsulfonyl)aniline (1.0 equiv.), **2** (1.1 equiv.), ZrO_2 (1.0 equiv.), **3a** (1.5 equiv.)	30Hz/90min	34
5	4-(methylsulfonyl)aniline (1.0 equiv.), **2** (1.1 equiv.), Yb_2O_3 (1.0 equiv.), **3a** (1.5 equiv.)	30Hz/90min	28
6	4-(methylsulfonyl)aniline (1.0 equiv.), **2** (1.1 equiv.), ZrO_2 (1.0 equiv.), **3b** (1.5 equiv.)	30Hz/90min	67
**7**	**4-(methylsulfonyl)aniline (1.0 equiv.), 2 (1.1 equiv.), ZrO_2 (1.0 equiv.), 3c (1.5 equiv.)**	**30Hz/90min**	**78**
8	4-(methylsulfonyl)aniline (1.0 equiv.), **2** (1.1 equiv.), ZrO_2 (1.0 equiv.), **3d** (1.5 equiv.)	30Hz/90min	49
9	4-(methylsulfonyl)aniline (1.0 equiv.), **2** (1.1 equiv.), ZrO_2 (1.0 equiv.), **3e** (1.5 equiv.)	30Hz/90min	60
10	4-(methylsulfonyl)aniline (1.0 equiv.), **2** (1.1 equiv.), ZrO_2 (1.0 equiv.), **3f** (1.5 equiv.)	30Hz/90min	0
11	4-(methylsulfonyl)aniline (1.0 equiv.), **2** (1.1 equiv.), ZrO_2 (1.0 equiv.), **3g** (1.5 equiv.)	30Hz/90min	0
12	4-(methylsulfonyl)aniline (1.0 equiv.), **2** (1.1 equiv.), ZrO_2 (1.0 equiv.), **3h** (1.5 equiv.)	30Hz/90min	0
	**Reactions in solution**		
13	4-(methylsulfonyl)aniline (1.0 equiv.), **2** (1.2 equiv.), **3c** (2.2 equiv.), MeOH, r.t.	------/24h	0
14	4-(methylsulfonyl)aniline (1.0 equiv.), **2** (1.2 equiv.), **3c** (2.2 equiv.), MeOH, 50 °C	------/24h	0
15	4-(methylsulfonyl)aniline (1.0 equiv.), **2** (1.2 equiv.), **3c** (2.2 equiv.), MeOH, reflux	------/12h	0
16	4-(methylsulfonyl)aniline (1.0 equiv.), **2** (1.2 equiv.), **3c** (2.2 equiv.), CH_3CN, r.t.	------/24h	0
17	4-(methylsulfonyl)aniline (1.0 equiv.), **2** (1.2 equiv.), **3c** (2.2 equiv.), CH_3CN, reflux	------/12h	0
18	4-(methylsulfonyl)aniline (1.0 equiv.), **2** (1.2 equiv.), **3c** (2.2 equiv.), 1,4-dioxane, reflux	------/24h	8
19	4-(methylsulfonyl)aniline (1.0 equiv.), **2** (1.2 equiv.), **3c** (2.2 equiv.), 1,4-dioxane, reflux	------/144h	32
20	4-(methylsulfonyl)aniline (1.0 equiv.), **2** (1.2 equiv.), **3c** (2.2 equiv.), dichloromethane, r.t.	------/24h	0
21	4-(methylsulfonyl)aniline (1.0 equiv.), **2** (1.2 equiv.), **3c** (2.2 equiv.), dichloromethane, reflux	------/24h	0

22	4-(methylsulfonyl)aniline (1.0 equiv.), **2** (1.2 equiv.), **3c** (2.2 equiv.), DMF, r.t.	------/12h	0
23	4-(methylsulfonyl)aniline (1.0 equiv.), **2** (1.2 equiv.), **3c** (2.2 equiv.), DMF, 60 °C	------/24h	0
24	4-(methylsulfonyl)aniline (1.0 equiv.), **2** (1.2 equiv.), **3c** (2.2 equiv.), DMF, 90 °C	------/24h	0
25	4-(methylsulfonyl)aniline (1.0 equiv.), **2** (1.2 equiv.), **3c** (2.2 equiv.), DMF, 130 °C	------/24h	0
26	4-(methylsulfonyl)aniline (1.0 equiv.), **2** (1.2 equiv.), **3c** (2.2 equiv.), DMA, r.t.	------/24h	0
27	4-(methylsulfonyl)aniline (1.0 equiv.), **2** (1.2 equiv.), **3c** (2.2 equiv.), DMA, 60 °C	------/24h	0
28	4-(methylsulfonyl)aniline (1.0 equiv.), **2** (1.2 equiv.), **3c** (2.2 equiv.), DMA, 90 °C	------/24h	0
29	4-(methylsulfonyl)aniline (1.0 equiv.), **2** (1.2 equiv.), **3c** (2.2 equiv.), DMA, 125 °C	------/24h	0
30	4-(methylsulfonyl)aniline (1.0 equiv.), **2** (1.2 equiv.), **3c** (2.2 equiv.), DMA, 160 °C	------/24h	0
31	[a] 1-(4-(methylsulfonyl)phenyl)pyridin-1-ium $^{-}BF_4$ (1 equiv.), **3c** (2.2 equiv.), 1,4-dioxane, reflux	------/24h	0
[a] obtained according to Moser *et al.* 2018			

Reaction procedures with optimised reaction conditions.

General procedure for the in-solution attempts.

Inside a glovebox, starting aniline (1.0 mmol, 1.0 euiv.), pyrylium tetrafluoroborate (1.2 mmol, 1.2 equiv.) and 1-methyl-1,4-diazabicyclo[2.2.2]octan-1-ium trifluoromethanolate (**3c**) (2.2 mmol, 2.2 equiv.) were weighed and placed successively into an Ace Pressure Tube equipped with a magnetic stir bar. Finally, 10 mL of dry methanol (or other solvent) were added inside the glovebox, then the reaction vessel was capped with a stopper. Subsequently, the Pressure Tube was taken out of the glovebox and heated at appropriate temperature for 12 or 24 hours. Upon completion, the reaction mixture was cooled to room temperature and analysed by TLC and GS MS. Finally, the reaction mixture was concentrated under vacuum, the formed crude was washed with water, filtrated and dried. The residue was subjected to preparative column chromatography on Silica gel using hexane/ethyl acetate mixtures.

General procedure for the solid-state reaction.

Inside a glovebox, a stainless steel 5 mL grinding vessel equipped with two balls (stainless steel, Φ=5 mm) was loaded consecutively with an appropriate aniline starting material (1.0 mmol, 1.0 equiv.), pyrylium tetrafluoroborate (1.1 mmol, 1.1 equiv.) and ZrO_2 (grinding auxiliary) (1.0 mmol, 1.0 equiv.). Lastly, freshly prepared salt **3c** (1-methyl-1,4-diazabicyclo[2.2.2]octan-1-ium trifluoromethoxide) (1.5 mmol, 1.5 equiv.) was added and the reaction vessel was properly sealed. The reaction vessel was installed on the ball mill and the contents were pulverized at 30Hz for 1.5h. After completion of the reaction, the content of the vessel was directly subjected to flash chromatography on silica gel to isolate the desired compound using gradient elution.

The gram scale synthesis was performed with 10 mmol of the starting amine in 25 mL grinding vessel using three Φ=10 mm balls.

(B) Characterization of products.

Example of a detailed spectral data for compound 4s.

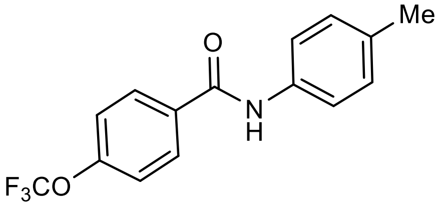

N-(p-tolyl)-4-(trifluoromethoxy)benzamide 4s.

White solid, mp 94 -95 °C **^{1}H NMR** (500 MHz, DMSO-*d*$_6$): δ 10.28 (s, 1H, NH), 8.07 (d, 2H, ^3J = 9.1 Hz, CH_{Ar}), 7.64 (d, 2H, ^3J = 9.1 Hz, CH_{Ar}), 7.51 (d, 2H, ^3J = 9.1 Hz, CH_{Ar}), 7.15 (d, 2H, ^3J = 7.6 Hz, CH_{Ar}), 2.28 (s, 3H, Me).

^{19}F NMR (188 MHz, DMSO-*d*$_6$): δ -56.1 (OCF_3).

^{13}C NMR (126 MHz, DMSO-*d*$_6$): δ 164.1, 150.4, 136.5, 134.2, 132.9, 130.0, 129.1, 120.7, 120.4, 120.0 (q, $^1J_{CF}$ = 256.9 Hz, OCF_3), 20.5.

HRMS (TOF MS ES+) m/z: $[M + H]^+$: Calcd for $C_{15}H_{13}NO_2F_3$ 296.0902. Found 296.0898.

MS (GC, 70eV): m/z (%) = 244 (M^+, 29), 202 (100), 182 (13), 162 (20), 133 (35).

Anal. calcd. for $C_{10}H_7F_3O_2N_2$: C 49.19; H, 2.89; N 11.47. Found: C 49.21; H, 3.01; N 11.39.

SpinWorks 4: IVA 936-2 1H DMSO

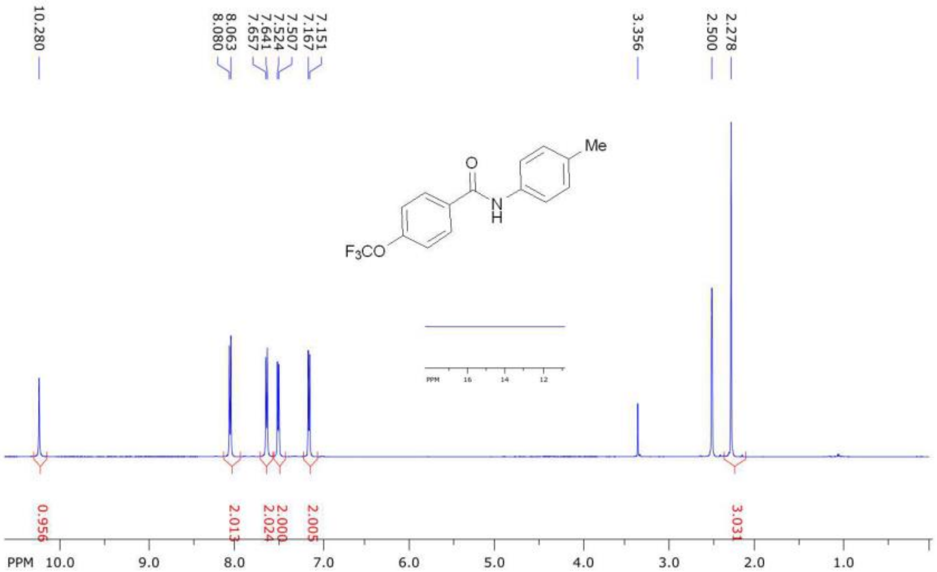

file: D:\NAPO\NMR\500-1\mkr13003\7\fid expt: <zg30>
transmitter freq.: 500.133001 MHz
time domain size: 65536 points
width: 12335.53 Hz = 24.6645 ppm = 0.188225 Hz/pt
number of scans: 24

freq. of 0 ppm: 500.130005 MHz
processed size: 65536 complex points
LB: 0.300 GF: 0.0000
Hz/cm: 213.961 ppm/cm: 0.42781

SpinWorks 4: IVA 936-2 13C DMSO

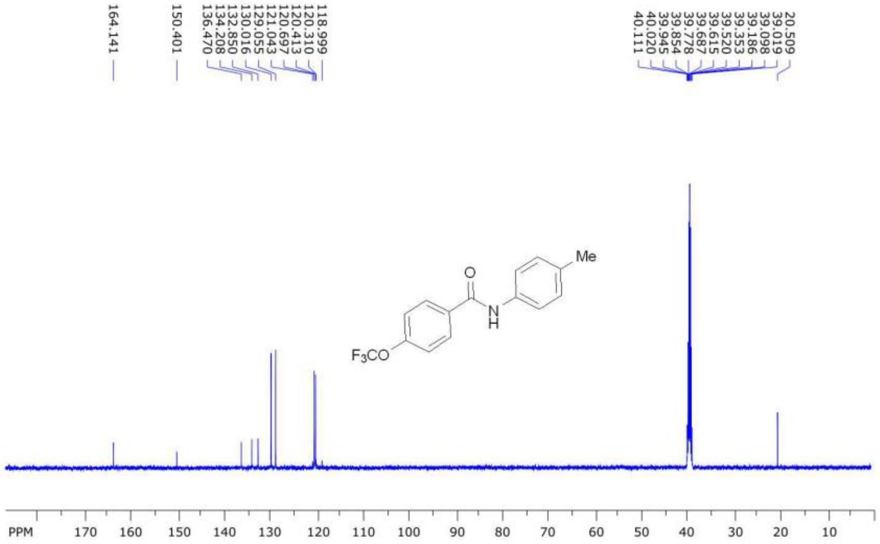

file: D:\NAPO\NMR\500-1\mkr13003\8\fid expt: <zgpg30>
transmitter freq.: 125.772879 MHz
time domain size: 65536 points
width: 36057.69 Hz = 286.6889 ppm = 0.550197 Hz/pt
number of scans: 256

freq. of 0 ppm: 125.757842 MHz
processed size: 32768 complex points
LB: 2.000 GF: 0.0000
Hz/cm: 941.329 ppm/cm: 7.48436

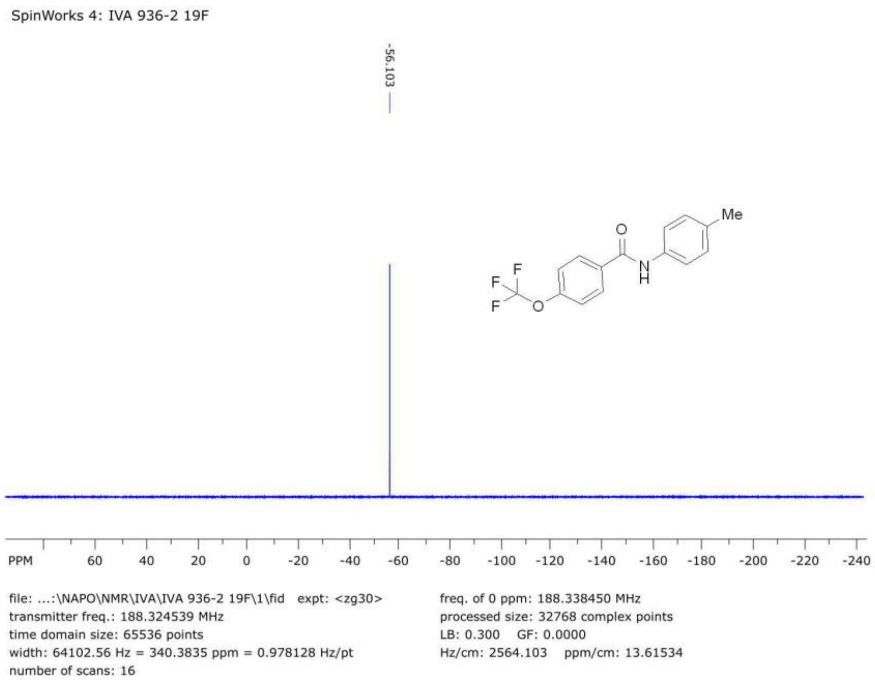
SpinWorks 4: IVA 936-2 19F
-56.103
F
F
F
O
O
N
H
Me
PPM 60 40 20 0 -20 -40 -60 -80 -100 -120 -140 -160 -180 -200 -220 -240
file: ...:\NAPO\NMR\IVA\IVA 936-2 19F\1\fid expt: <zg30>
transmitter freq.: 188.324539 MHz
time domain size: 65536 points
width: 64102.56 Hz = 340.3835 ppm = 0.978128 Hz/pt
number of scans: 16
freq. of 0 ppm: 188.338450 MHz
processed size: 32768 complex points
LB: 0.300 GF: 0.0000
Hz/cm: 2564.103 ppm/cm: 13.61534

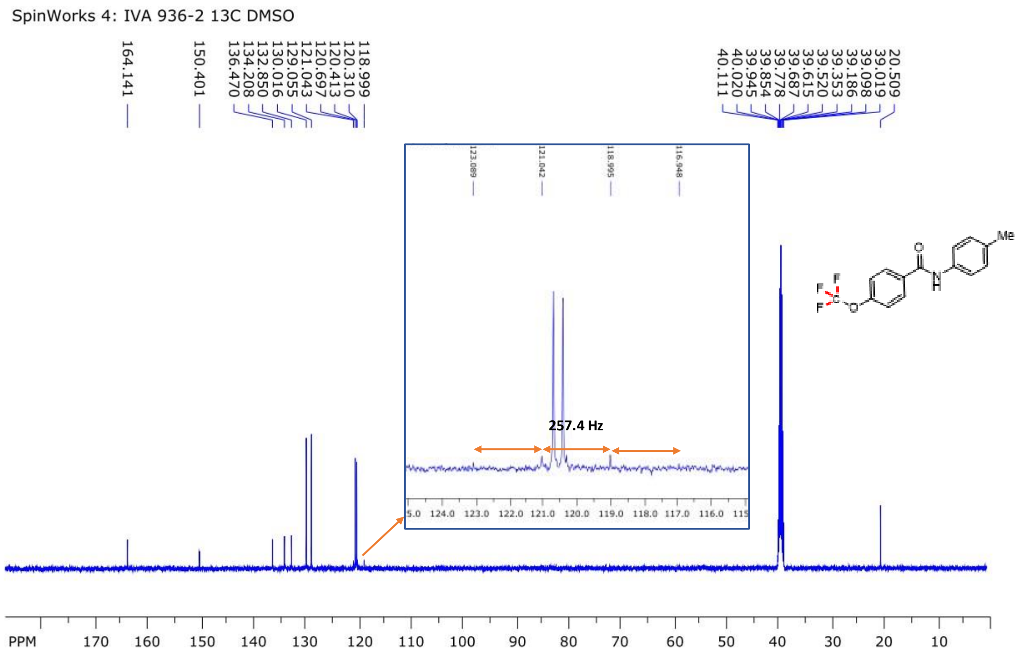
SpinWorks 4: IVA 936-2 13C DMSO
164.141
150.401
136.470
134.208
132.850
130.016
129.055
121.043
120.697
120.413
120.310
118.999
40.111
40.020
39.945
39.854
39.778
39.687
39.615
39.520
39.353
39.186
39.098
39.019
20.509
123.089
121.042
118.995
116.948
257.4 Hz
5.0 124.0 123.0 122.0 121.0 120.0 119.0 118.0 117.0 116.0 115
Me
PPM 170 160 150 140 130 120 110 100 90 80 70 60 50 40 30 20 10

Sample Information

Sample Name	: M.Satenik
Sample ID	: IVA936
Analyzed	: 2019-01-09 15:59:47
Vial #	: 12
Injection Volume	: 6.00
$EndIf$Data File	: C:\GCMSsolution\Data\widma\2019\01_2019\M
Method File	: C:\GCMSsolution\System\Tune1\DI EI 1 metoda
Tuning File	: C:\GCMSsolution\System\Tune1\tuning 07.01.2(

Method

[Comment]

═══ Analytical Line 1 ═══

[GC-2010]

Column Oven Temp.	:30.0 °C
Injection Temp.	:150.00 °C
Injection Mode	:Split
Pressure	:12.8 kPa
Total Flow	:6.6 mL/min
Column Flow	:0.60 mL/min
Linear Velocity	:27.9 cm/sec
Purge Flow	:3.0 mL/min
Split Ratio	:5.0

[GCMS-QP2010 Ultra]

IonSourceTemp	:200.00 °C
Interface Temp.	:200.00 °C
Solvent Cut Time	:0.30 min
Detector Gain Mode	:Relative
Detector Gain	:1.29 kV +0.00 kV
Threshold	:0

[MS Table]

--Group 1 - Event 1--

Start Time	:0.30min
End Time	:25.00min
ACQ Mode	:Scan
Event Time	:0.10sec
Scan Speed	:20000
Start m/z	:35.00
End m/z	:1090.00

Chromatogram M.Satenik C:\GCMSsolution\Data\widma\2019\01_2019\M.Satenik_IVA_936.QGD

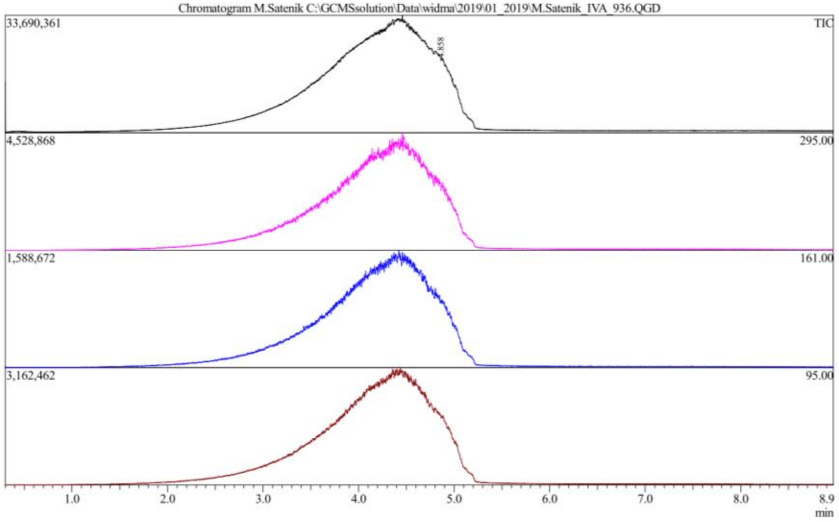

Spectrum

Line#:1 R.Time:4.858(Scan#:2736)
MassPeaks:593
RawMode:Single 4.858(2736) BasePeak:189(7546786)
BG Mode:None Group 1 - Event 1 Scan

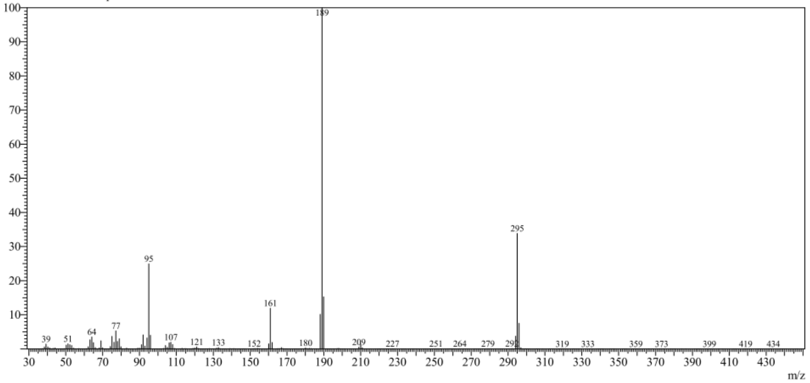

Mass Table
Line#:1 R.Time:4.858(Scan#:2736)
MassPeaks:593
RawMode:Single 4.858(2736) BasePeak:189(7546786)
BG Mode:None Group 1 - Event 1 Scan

#	m/z	Abs. Int.	Rel. Int.
1	35.00	545	0.01
2	36.35	1580	0.02
3	37.35	8428	0.11
4	38.35	43668	0.58
5	39.30	106705	1.41
6	40.25	45009	0.60
7	41.25	24544	0.33
8	42.25	6353	0.08
9	43.15	14644	0.19
10	44.10	22551	0.30
11	45.05	3577	0.05
12	46.10	442	0.01
13	47.25	2480	0.03
14	48.20	403	0.01
15	49.25	7958	0.11
16	50.25	86426	1.15
17	51.25	114153	1.51
18	52.15	93292	1.24
19	53.15	76711	1.02
20	54.15	16895	0.22
21	55.15	5499	0.07
22	56.25	3407	0.05
23	57.20	9552	0.13
24	58.15	3430	0.05
25	59.10	1202	0.02
26	60.15	2252	0.03
27	61.15	8180	0.11
28	62.15	48757	0.65
29	63.15	204826	2.71
30	64.10	265878	3.52
31	65.05	137823	1.83
32	66.05	32316	0.43
33	67.00	6108	0.08
34	68.05	20888	0.28
35	69.05	179297	2.38
36	69.95	26424	0.35
37	71.15	3247	0.04
38	72.10	455	0.01
39	73.15	5358	0.07
40	74.15	53984	0.72
41	75.10	277799	3.68
42	76.15	146839	1.95
43	77.10	399023	5.29
44	78.05	165944	2.20
45	79.05	224545	2.98

#	m/z	Abs. Int.	Rel. Int.
46	79.95	50921	0.67
47	80.95	2871	0.04
48	82.05	3263	0.04
49	83.00	18368	0.24
50	83.95	3910	0.05
51	84.95	2531	0.03
52	85.95	2950	0.04
53	87.05	1882	0.02
54	88.05	3546	0.05
55	89.05	18098	0.24
56	90.05	19428	0.26
57	91.05	94480	1.25
58	92.00	309315	4.10
59	92.95	63081	0.84
60	94.05	245124	3.25
61	95.00	1883270	24.95
62	95.95	301403	3.99
63	97.95	1607	0.02
64	99.05	2408	0.03
65	99.95	2076	0.03
66	101.05	3281	0.04
67	102.05	5671	0.08
68	103.05	14759	0.20
69	104.05	74960	0.99
70	105.05	35784	0.47
71	106.05	135923	1.80
72	106.95	146589	1.94
73	107.95	99717	1.32
74	108.95	15602	0.21
75	109.95	1062	0.01
76	111.05	1849	0.02
77	112.05	2718	0.04
78	113.00	15683	0.21
79	113.95	5909	0.08
80	114.80	7575	0.10
81	115.75	2882	0.04
82	117.05	2227	0.03
83	118.05	2351	0.03
84	119.05	13380	0.18
85	120.05	22168	0.29
86	121.00	46243	0.61
87	121.95	11373	0.15
88	122.95	4078	0.05
89	123.95	1103	0.01
90	124.90	907	0.01

#	m/z	Abs. Int.	Rel. Int.
91	125.95	2311	0.03
92	126.95	4410	0.06
93	127.95	8263	0.11
94	128.85	3160	0.04
95	129.85	2435	0.03
96	130.85	1523	0.02
97	131.85	17713	0.23
98	132.85	30064	0.40
99	133.75	9625	0.13
100	134.75	1546	0.02
101	135.80	50	0.00
102	136.80	117	0.00
103	138.15	4200	0.06
104	139.10	10698	0.14
105	140.05	6281	0.08
106	141.05	7318	0.10
107	142.05	2822	0.04
108	143.40	1423	0.02
109	144.40	435	0.01
110	145.60	593	0.01
111	146.55	3023	0.04
112	147.55	1875	0.02
113	149.15	1356	0.02
114	150.15	1150	0.02
115	151.15	2909	0.04
116	152.15	11215	0.15
117	153.15	8873	0.12
118	154.10	14212	0.19
119	155.05	7043	0.09
120	156.10	1643	0.02
121	157.00	734	0.01
122	158.05	1388	0.02
123	159.05	415	0.01
124	160.05	111902	1.48
125	161.00	902626	11.96
126	161.95	144639	1.92
127	164.15	2136	0.03
128	165.05	8017	0.11
129	166.15	9363	0.12
130	167.10	31700	0.42
131	168.05	11128	0.15
132	169.05	3726	0.05
133	170.05	2622	0.03
134	171.10	777	0.01
135	172.10	400	0.01

#	m/z	Abs. Int.	Rel. Int.
136	173.10	1022	0.01
137	174.10	670	0.01
138	175.10	382	0.01
139	176.10	286	0.00
140	177.15	2369	0.03
141	178.10	4893	0.06
142	179.15	5878	0.08
143	180.10	20669	0.27
144	181.05	9908	0.13
145	182.15	11986	0.16
146	183.15	8495	0.11
147	184.15	2679	0.04
148	185.05	2390	0.03
149	186.05	4054	0.05
150	188.05	767596	10.17
151	189.00	7546786	100.00
152	189.95	1154323	15.30
153	190.95	6175	0.08
154	191.95	2506	0.03
155	192.90	4451	0.06
156	193.85	2910	0.04
157	195.15	2764	0.04
158	196.15	5479	0.07
159	197.15	7916	0.10
160	198.10	12757	0.17
161	199.05	4580	0.06
162	200.05	1254	0.02
163	201.20	1366	0.02
164	204.20	414	0.01
165	205.20	89	0.00
166	206.00	955	0.01
167	206.95	1098	0.01
168	208.05	5040	0.07
169	209.05	41487	0.55
170	210.00	112690	1.49
171	210.95	25733	0.34
172	211.95	1596	0.02
173	212.95	1621	0.02
174	214.00	611	0.01
175	215.00	68	0.00
176	218.00	332	0.00
177	219.40	1313	0.02
178	220.40	315	0.00
179	221.40	415	0.01
180	222.40	206	0.00
181	223.40	483	0.01
182	224.40	446	0.01
183	225.15	1070	0.01
184	226.25	1427	0.02
185	227.20	4628	0.06
186	228.15	1468	0.02
187	232.20	319	0.00
188	233.20	710	0.01
189	234.20	510	0.01
190	236.20	27	0.00
191	237.20	180	0.00
192	238.30	427	0.01
193	239.35	1533	0.02
194	240.30	906	0.01
195	241.30	844	0.01
196	244.30	210	0.00
197	245.30	663	0.01
198	246.30	256	0.00
199	247.30	587	0.01
200	248.20	303	0.00
201	249.15	1405	0.02
202	249.90	376	0.00
203	250.90	6428	0.09
204	251.85	2534	0.03
205	252.85	889	0.01
206	253.90	628	0.01
207	255.90	789	0.01
208	256.90	215	0.00
209	257.90	565	0.01
210	258.50	424	0.01
211	259.50	1265	0.02
212	260.50	531	0.01
213	261.50	196	0.00
214	262.95	1388	0.02
215	263.85	5640	0.07
216	264.85	4244	0.06
217	265.95	3438	0.05
218	266.85	1871	0.02
219	267.80	1248	0.02
220	268.80	208	0.00
221	271.80	647	0.01

#	m/z	Abs. Int.	Rel. Int.
222	272.80	684	0.01
223	273.80	688	0.01
224	274.80	323	0.00
225	276.10	2834	0.04
226	277.00	673	0.01
227	277.95	2112	0.03
228	279.00	4427	0.06
229	279.95	1477	0.02
230	281.00	651	0.01
231	286.00	426	0.01
232	287.00	205	0.00
233	291.00	334	0.00
234	292.05	9291	0.12
235	293.05	2481	0.03
236	294.05	286090	3.79
237	295.00	2556511	33.88
238	295.95	568613	7.53
239	296.95	28709	0.38
240	297.95	2192	0.03
241	298.95	844	0.01
242	299.60	1688	0.02
243	300.55	1130	0.01
244	301.60	398	0.01
245	305.60	215	0.00
246	306.60	196	0.00
247	308.60	163	0.00
248	312.60	656	0.01
249	313.60	378	0.01
250	314.60	408	0.01
251	317.60	84	0.00
252	318.30	373	0.00
253	319.30	1421	0.02
254	320.30	655	0.01
255	321.30	26	0.00
256	325.30	14	0.00
257	326.30	238	0.00
258	327.30	457	0.01
259	328.30	111	0.00
260	332.30	570	0.01
261	333.30	721	0.01
262	334.30	539	0.01
263	335.30	205	0.00
264	338.30	280	0.00
265	339.30	992	0.01
266	340.30	593	0.01
267	341.30	258	0.00
268	345.30	105	0.00
269	346.30	179	0.00
270	347.30	177	0.00
271	352.30	391	0.01
272	353.30	554	0.01
273	354.30	365	0.00
274	357.30	95	0.00
275	358.30	266	0.00
276	359.30	1123	0.01
277	360.30	353	0.00
278	361.30	46	0.00
279	366.30	515	0.01
280	368.30	65	0.00
281	372.30	346	0.00
282	373.30	639	0.01
283	374.30	232	0.00
284	378.30	274	0.00
285	379.30	1039	0.01
286	380.30	434	0.01
287	384.30	45	0.00
288	386.30	445	0.01
289	391.30	1	0.00
290	392.30	402	0.01
291	393.30	521	0.01
292	394.30	536	0.01
293	397.30	19	0.00
294	398.30	284	0.00
295	399.20	1471	0.02
296	400.20	184	0.00
297	401.20	112	0.00
298	404.20	76	0.00
299	406.20	299	0.00
300	412.20	546	0.01
301	413.20	209	0.00
302	414.20	507	0.01
303	417.90	136	0.00
304	418.90	1325	0.02
305	419.90	389	0.01
306	420.90	424	0.01
307	425.90	39	0.00

#	m/z	Abs. Int.	Rel. Int.
308	426.90	100	0.00
309	431.90	444	0.01
310	432.90	480	0.01
311	433.90	687	0.01
312	437.90	82	0.00
313	438.90	1041	0.01
314	439.90	523	0.01
315	440.90	255	0.00
316	443.90	86	0.00
317	445.90	186	0.00
318	446.90	208	0.00
319	451.90	284	0.00
320	452.90	399	0.01
321	453.90	712	0.01
322	457.90	129	0.00
323	458.90	1070	0.01
324	459.90	452	0.01
325	460.90	107	0.00
326	464.90	77	0.00
327	465.90	135	0.00
328	466.90	236	0.00
329	471.90	77	0.00
330	472.90	561	0.01
331	473.90	562	0.01
332	477.90	401	0.01
333	478.90	841	0.01
334	479.90	730	0.01
335	480.90	18	0.00
336	484.90	445	0.01
337	485.90	23	0.00
338	486.90	300	0.00
339	491.90	336	0.00
340	492.90	429	0.01
341	493.90	488	0.01
342	496.90	68	0.00
343	497.90	288	0.00
344	498.90	1055	0.01
345	499.90	557	0.01
346	504.90	43	0.00
347	506.90	219	0.00
348	511.90	366	0.00
349	512.90	581	0.01
350	513.90	349	0.00
351	517.90	730	0.01
352	518.50	805	0.01
353	519.50	1205	0.02
354	526.50	486	0.01
355	531.50	325	0.00
356	532.50	497	0.01
357	533.50	438	0.01
358	534.50	81	0.00
359	537.50	328	0.00
360	538.95	1354	0.02
361	540.00	55	0.00
362	545.00	157	0.00
363	546.00	439	0.01
364	551.00	317	0.00
365	552.00	456	0.01
366	553.00	216	0.00
367	554.00	268	0.00
368	557.00	67	0.00
369	558.00	716	0.01
370	558.95	1211	0.02
371	559.90	116	0.00
372	564.90	212	0.00
373	565.90	188	0.00
374	566.90	85	0.00
375	570.90	28	0.00
376	571.90	727	0.01
377	572.90	52	0.00
378	573.90	471	0.01
379	576.90	224	0.00
380	577.90	603	0.01
381	578.75	1200	0.02
382	579.70	117	0.00
383	583.70	136	0.00
384	584.70	35	0.00
385	585.70	467	0.01
386	590.70	57	0.00
387	591.70	258	0.00
388	592.70	416	0.01
389	593.70	356	0.00
390	597.70	846	0.01
391	598.70	981	0.01
392	599.70	50	0.00
393	604.70	228	0.00

Elemental Composition Report **Page 1**

Single Mass Analysis
Tolerance = 5.0 PPM / DBE: min = -50.0, max = 80.0
Element prediction: Off
Number of isotope peaks used for i-FIT = 5

Monoisotopic Mass, Even Electron Ions
34 formula(e) evaluated with 1 results within limits (up to 50 closest results for each mass)
Elements Used:
C: 0-17 H: 0-15 N: 0-2 O: 0-2 F: 0-4
190110_IVA_936 16 (0.177) Cm (13:16-(2:10+48:71))
1: TOF MS ES+

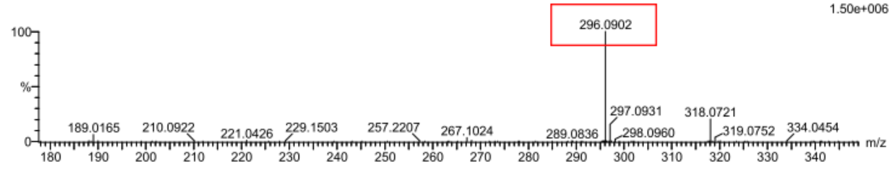

Minimum:				-50.0				
Maximum:		15.0	5.0	80.0				
Mass	Calc. Mass	mDa	PPM	DBE	i-FIT	Norm	Conf(%)	Formula
296.0902	296.0898	0.4	1.4	8.5	194.9	n/a	n/a	C15 H13 N O2 F3

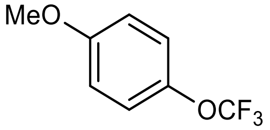

1-methoxy-4-(trifluoromethoxy)benzene 4a.

The title compound was prepared starting from aniline **1a** (123 mg, 1.0 mmol, 1.0 equiv.), pyrylium tetrafluoroborate (185 mg, 1.1 mmol, 1.1 equiv.), ZrO_2 (123 mg, 1.0 mmol, 1.0 equiv.) and **3c** (318 mg, 1.5 mmol, 1.5 equiv.). The purification was accomplished by column chromatography on silica gel to provide the desired product **4a** (175 mg, 0.91 mmol, 91%).

Colorless liquid, **^{1}H NMR** (500 MHz, $CDCl_3$): δ 7.16 (d, 2H, ^3J = 9.0 Hz, CH_{Ar}), 6.91 - 6.87 (m, 2H, CH_{Ar}), 3.81 (s, 3H, CH_3).

^{13}C NMR (126 MHz, $CDCl_3$): δ 158.3, 142.9, 122.6, 120.8 (q, $^1J_{CF}$ = 255.9 Hz, CF_3), 114.8, 55.7.

Anal. calcd. for $C_8H_7O_2F_3$: C, 50.01; H, 3.67. Found: C, 50.06; H, 3.76.

The ^{1}H, ^{13}C, ^{19}F NMR data of this compaunds is discribed in the literature: C. Huang, T. Liang, S. Harada, E. Lee, T. Ritter, *J. Am. Chem. Soc.* **2011**, *133*, 13308–13310.

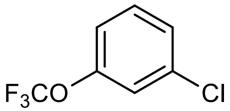

1-chloro-3-(trifluoromethoxy)benzene 4b.

The title compound was prepared starting from aniline **1b** (128 mg, 1.0 mmol, 1.0 equiv.), pyrylium tetrafluoroborate (185 mg, 1.1 mmol, 1.1 equiv.), ZrO_2 (123 mg, 1.0 mmol, 1.0 equiv.) and **3c** (318 mg, 1.5 mmol, 1.5 equiv.). The purification was accomplished by column chromatography on silica gel to provide the desired product **4b** (181 mg, 0.92 mmol, 92%).

Yellowish liquid, **^{1}H NMR** (500 MHz, $CDCl_3$): δ 7.33 (t, 1H, ^3J = 8.0 Hz, CH_{Ar}), 7.29 (dt, ^3J = 8.0 Hz, ^4J = 1.4 Hz, CH_{Ar}), 7.26 - 7.24 (m, 1H, CH_{Ar}), 7.16 - 7.11 (m, 1H, CH_{Ar}).

^{13}C NMR (126 MHz, $CDCl_3$): δ 149.8, 135.4, 130.7, 127.3, 121.8, 120.5 (q, $^1J_{CF}$ = 258.2 Hz, CF_3), 119.3.

Anal. calcd. for $C_7H_4OClF_3$: C, 42.78; H, 2.05. Found: C, 42.87; H, 2.13.

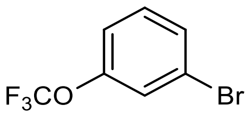

1-bromo-3-(trifluoromethoxy)benzene 4c.

The title compound was prepared starting from aniline **1c** (172 mg, 1.0 mmol, 1.0 equiv.), pyrylium tetrafluoroborate (185 mg, 1.1 mmol, 1.1 equiv.), ZrO_2 (123 mg, 1.0 mmol, 1.0 equiv.) and **3c** (318 mg, 1.5 mmol, 1.5 equiv.). The purification was accomplished by column chromatography on silica gel to provide the desired product **4c** (214 mg, 0.89 mmol, 89%).

Colorless liquid, **^{1}H NMR** (500 MHz, $CDCl_3$): δ 7.44 (d, 1H, ^3J = 8.0 Hz, CH_{Ar}), 7.42 - 7.40 (m, 1H, CH_{Ar}), 7.26 (t, 1H, ^3J = 8.1 Hz, CH_{Ar}), 7.20 - 7.16 (m, 1H, CH_{Ar}).

^{13}C NMR (126 MHz, $CDCl_3$): δ 149.8, 131.0, 130.2, 124.6, 122.9, 120.5 (q, $^1J_{CF}$ = 258.2 Hz, CF_3), 119.8.

Anal. calcd. for $C_7H_4OClF_3$: C, 34.89; H, 1.67. Found: C, 34.96; H, 1.59.

The ^{1}H, ^{13}C, ^{19}F NMR data of this compaunds is discribed in the literature: Y-M. Yang, J-F. Yao, W. Yan, Z. Luo**,** Z.-Y. Tang**,** *Org. Lett.* **2019**, *21*, 8003–8007.

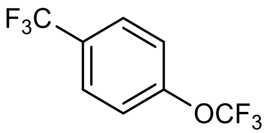

1-(trifluoromethoxy)-4-(trifluoromethyl)benzene 4d.

The title compound was prepared starting from aniline **1d** (161 mg, 1.0 mmol, 1.0 equiv.), pyrylium tetrafluoroborate (185 mg, 1.1 mmol, 1.1 equiv.), ZrO_2 (123 mg, 1.0 mmol, 1.0 equiv.) and **3c** (318 mg, 1.5 mmol, 1.5 equiv.). The purification was accomplished by column chromatography on silica gel to provide the desired product **4d** (172 mg, 0.75 mmol, 75%).

Colorless liquid, **^{1}H NMR** (500 MHz, $CDCl_3$): δ 7.68 (d, 2H, ^3J = 8.4 Hz, CH_{Ar}), 7.33 (d, 2H, ^3J = 8.2 Hz, CH_{Ar}).

^{13}C NMR (126 MHz, $CDCl_3$): δ 151.8, 129.3 (q, $^2J_{CF}$ = 33.1 Hz, C_{ipso}-CF_3), 127.5 (q, $^3J_{CF}$ = 3.5 Hz), 123.8 (q, $^1J_{CF}$ = 272.0 Hz, CF_3), 121.1, 120.5 (q, $^1J_{CF}$ = 258.6 Hz, OCF_3).

Anal. calcd. for $C_8H_4OF_6$: C, 41.76; H, 1.75. Found: C, 41.86; H, 1.86.

The ^{1}H, ^{19}F NMR data of this compaunds is discribed in the literature: S. Yang, M. Chen, P. Tang, *Angew. Chemie - Int. Ed.* **2019,** *58*, 7840–7844.

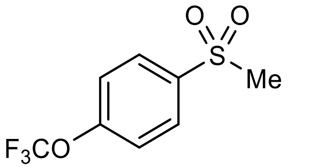

1-(methylsulfonyl)-4-(trifluoromethoxy)benzene 4e.

The title compound was prepared starting from aniline **1e** (171 mg, 1.0 mmol, 1.0 equiv.), pyrylium tetrafluoroborate (185 mg, 1.1 mmol, 1.1 equiv.), ZrO_2 (123 mg, 1.0 mmol, 1.0 equiv.) and **3c** (318 mg, 1.5 mmol, 1.5 equiv.). The purification was accomplished by column chromatography on silica gel to provide the desired product **4e** (187 mg, 0.78 mmol, 78%).

yellowish solid, mp = 64-65 °C **^{1}H NMR** (500 MHz, $CDCl_3$): δ 7.99 (d, 2H, ^3J = 8.9 Hz, CH_{Ar}), 7.38 (d, 2H, ^3J = 8.7 Hz, CH_{Ar}), 3.06 (s, 3H, CH_3).

^{13}C NMR (126 MHz, $CDCl_3$): δ 153.0, 138.9, 129.8, 121.3, 120.3 (q, $^1J_{CF}$ = 259.6 Hz, CF_3), 44.6.

Anal. calcd. for $C_8H_7O_3SF_3$: C, 40.00; H, 2.94. Found: C, 40.09; H, 2.98.

The ^{1}H, ^{13}C, ^{19}F NMR data of this compaunds is discribed in the literature: S. Yang, M. Chen, P. Tang, *Angew. Chemie - Int. Ed.* **2019,** *58*, 7840–7844.

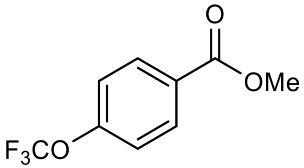

methyl 4-(trifluoromethoxy)benzoate 4f.

The title compound was prepared starting from aniline **1f** (151 mg, 1.0 mmol, 1.0 equiv.), pyrylium tetrafluoroborate (185 mg, 1.1 mmol, 1.1 equiv.), ZrO_2 (123 mg, 1.0 mmol, 1.0 equiv.) and **3c** (318 mg, 1.5 mmol, 1.5 equiv.). he purification was accomplished by column chromatography on silica gel to provide the desired product **4f** (194 mg, 0.88 mmol, 88%).

Colorless liquid, **^{1}H NMR** (500 MHz, $CDCl_3$): δ 8.07 (d,2H, ^3J = 8.9 Hz, CH_{Ar}), 7.24 (d, 2H, ^3J = 8.5 Hz, CH_{Ar}), 3.91 (s, 3H, CH_3).

^{13}C NMR (126 MHz, $CDCl_3$): δ 166.0, 152.7, 131.7, 128.7, 120.4 (q, $^1J_{CF}$ = 258.6 Hz, CF_3), 120.3, 52.4.

Anal. calcd. for $C_9H_7O_3F_3$: C, 49.10; H, 3.21. Found: C, 49.16; H, 3.32.

The ^{1}H, ^{13}C, ^{19}F NMR data of this compaunds is discribed in the literature: M. Zhou, C. Ni, Z. He, Jinbo Hu, *Org. Lett.* **2016**, *18*, 3754–3757.

and P. Boehm, S. Roediger, A. Bismuto, B. Morandi, *Angew. Chemie - Int. Ed.* **2020**, *59,* 17887–17896.

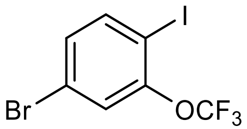

4-bromo-1-iodo-2-(trifluoromethoxy)benzene 4g.

The title compound was prepared starting from aniline **1g** (298 mg, 1.0 mmol, 1.0 equiv.), pyrylium tetrafluoroborate (185 mg, 1.1 mmol, 1.1 equiv.), ZrO_2 (123 mg, 1.0 mmol, 1.0 equiv.) and **3c** (318 mg, 1.5 mmol, 1.5 equiv.). The purification was accomplished by column chromatography on silica gel to provide the desired product **4g** (267 mg, 0.73 mmol, 73%).

Colorless liquid,**^{1}H NMR** (500 MHz, $CDCl_3$): δ 7.71 (d, 1H, ^3J = 8.5 Hz, CH_{Ar}), 7.42 (s, 1H, CH_{Ar}), 7.17 (dd, 1H, ^3J = 8.4 Hz, ^4J = 2.1 Hz, CH_{Ar}).

^{13}C NMR (126 MHz, $CDCl_3$): δ 150.0, 141.1, 131.7, 124.6, 122.8, 120.5 (q, $^1J_{CF}$ = 260.5 Hz, CF_3), 88.1.

Anal. calcd. for $C_7H_3OF_3BrI$: C 22.92; H, 0.82. Found: C, 23.01; H, 0.79.

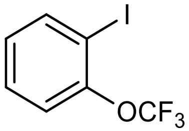

1-iodo-2-(trifluoromethoxy)benzene 4h.

The title compound was prepared starting from aniline **1h** (219 mg, 1.0 mmol, 1.0 equiv.), pyrylium tetrafluoroborate (185 mg, 1.1 mmol, 1.1 equiv.), ZrO_2 (123 mg, 1.0 mmol, 1.0 equiv.) and **3c** (318 mg, 1.5 mmol, 1.5 equiv.). The purification was accomplished by column chromatography on silica gel to provide the desired product **4h** (225 mg, 0.78 mmol, 78%).

Colorless liquid, **^{1}H NMR** (500 MHz, $CDCl_3$): δ 7.87 (dd, 1H, ^3J = 7.9 Hz, ^4J = 1.5 Hz, CH_{Ar}), 7.41 - 7.34 (m, 1H, CH_{Ar}), 7.31 - 7.27 (m, 1H, CH_{Ar}), 7.02 (td, 1H, ^3J = 7.8 Hz, ^4J = 1.4 Hz, CH_{Ar}).

^{13}C NMR (126 MHz, $CDCl_3$): δ 149.6, 140.4, 129.8, 128.4, 121.2, 120.7 (q, $^1J_{CF}$ = 259.2 Hz, CF_3), 89.8.

Anal. calcd. for $C_7H_4OF_3I$: C 29.19; H, 1.40. Found: C, 29.32; H, 1.45.

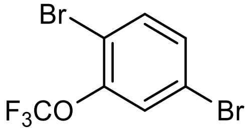

1,4-dibromo-2-(trifluoromethoxy)benzene 4i.

The title compound was prepared starting from aniline **1i** (250 mg, 1.0 mmol, 1.0 equiv.), pyrylium tetrafluoroborate (185 mg, 1.1 mmol, 1.1 equiv.), ZrO_2 (123 mg, 1.0 mmol, 1.0 equiv.) and **3c** (318 mg, 1.5 mmol, 1.5 equiv.). The purification was accomplished by column chromatography on silica gel to provide the desired product **4i** (246 mg, 0.77 mmol, 77%).

Colorless liquid, **^{1}H NMR** (500 MHz, $CDCl_3$): δ 7.50 (d, 1H, ^3J = 8.6 Hz, CH_{Ar}), 7.48 - 7.46 (m, 1H, CH_{Ar}), 7.31 (dd, 1H, ^3J = 8.6 Hz, ^4J = 2.2 Hz, CH_{Ar}).

^{13}C NMR (126 MHz, $CDCl_3$): δ 147.0, 135.0, 131.4, 125.7, 121.5, 120.5 (q, $^1J_{CF}$ = 260.3 Hz, CF_3), 115.3.

Anal. calcd. for $C_7H_3Br_2F_3O$: C 26.28; H, 0.95. Found: C, 26.31; H, 1.01.

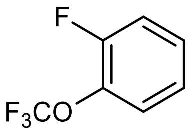

1-fluoro-2-(trifluoromethoxy)benzene 4j.

The title compound was prepared starting from aniline **1j** (111 mg, 1.0 mmol, 1.0 equiv.), pyrylium tetrafluoroborate (185 mg, 1.1 mmol, 1.1 equiv.), ZrO_2 (123 mg, 1.0 mmol, 1.0 equiv.) and **3c** (318 mg, 1.5 mmol, 1.5 equiv.). The purification was accomplished by column chromatography on silica gel to provide the desired product **4j** (151 mg, 0.84 mmol, 84%).

Colorless liquid, **^{1}H NMR** (500 MHz, DMSO-d_6): δ 7.53 - 7.39 (m, 3H, CH_{Ar}), 7.31 - 7.24 (m, 1H, CH_{Ar}).

^{13}C NMR (126 MHz, DMSO-d_6): δ 153.9 (d, $^1J_{CF}$ = 250.0 Hz, C_{ipso}-F), 135.5 (d, $^3J_{CF}$ = 12.2 Hz), 129.4, 125.5, 124.0, 120.1 (q, $^1J_{CF}$ = 257.6 Hz, CF_3), 117.5 (d, $^2J_{CF}$ = 18.0 Hz).

Anal. calcd. for $C_7H_4F_4O$: C 46.68; H, 2.24. Found: C, 46.61; H, 2.29.

The ^{19}F NMR data of this compaunds is discribed in the literature: A. V. Nyuchev, T. Wan, B. Cendón, C. Sambiagio, J. J. C. Struijs, M. Ho, M. Gulías, Y. Wang, T. Noël, *Beilstein J. Org. Chem.* **2020**, *16*, 1305–1312.

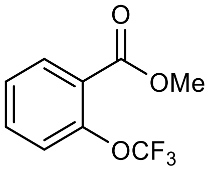

methyl 2-(trifluoromethoxy)benzoate 4k.

The title compound was prepared starting from aniline **1k** (151 mg, 1.0 mmol, 1.0 equiv.), pyrylium tetrafluoroborate (185 mg, 1.1 mmol, 1.1 equiv.), ZrO_2 (123 mg, 1.0 mmol, 1.0 equiv.) and **3c** (318 mg, 1.5 mmol, 1.5 equiv.). The purification was accomplished by column chromatography on silica gel to provide the desired product **4k** (156 mg, 0.71 mmol, 71%).

Colorless liquid, **^{1}H NMR** (500 MHz, $CDCl_3$): δ 7.93 (dd, 1H, ^3J = 7.8 Hz, ^4J = 1.7 Hz, CH_{Ar}), 7.53 (td, 1H, ^3J = 8.2 Hz, ^4J = 1.7 Hz, CH_{Ar}), 7.35 (t, ^3J = 7.6 Hz, CH_{Ar}), 7.31 (d, 1H, ^3J = 8.2 Hz, CH_{Ar}), 3.90 (s, 3H, CH_3).

^{13}C NMR (126 MHz, $CDCl_3$): δ 165.1, 147.8, 133.6, 132.1, 127.1, 125.1, 122.7, 120.5 (q, $^1J_{CF}$ = 257.7 Hz, CF_3), 52.5.

Anal. calcd. for $C_9H_3F_3O_3$: C 49.10; H, 3.21. Found: C, 49.21; H, 3.19.

The ^{1}H, ^{13}C, ^{19}F NMR data of this compaunds is discribed in the literature: W. Zheng, C. A. Morales-Rivera, J. W. Lee, P. Liu, M-Y. Ngai, *Angew. Chemie - Int. Ed.* **2018**, *57*, 9645–9649.

and W. Zheng, J. W. Lee, C. A. Morales-Rivera, P. Liu, M-Y. Ngai, *Angew. Chemie - Int. Ed.* **2018**, *57*, 13795–13799.

and J. W. Lee, S. Lim, D. N. Maienshein, P. Liu, M.-Y. Ngai, *Angew. Chemie - Int. Ed.* **2020**, *59*, 21475–21480.

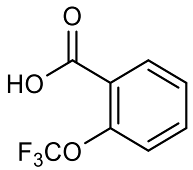

2-(trifluoromethoxy)benzoic acid 4l.

The title compound was prepared starting from aniline **1l** (137 mg, 1.0 mmol, 1.0 equiv.), pyrylium tetrafluoroborate (185 mg, 1.1 mmol, 1.1 equiv.), ZrO_2 (123 mg, 1.0 mmol, 1.0 equiv.) and **3c** (318 mg, 1.5 mmol, 1.5 equiv.). The purification was accomplished by column chromatography on silica gel to provide the desired product **4l** (119 mg, 0.58 mmol, 58%).

White solid, mp = 79-81 °C **^{1}H NMR** (500 MHz, $CDCl_3$): δ 11.90 (br s, 1H, COOH), 8.11 (dd, 1H, ^3J = 7.8 Hz, ^4J = 1.7 Hz, CH_{Ar}), 7.64 (td, 1H, ^3J = 8.1 Hz, ^4J = 1.7 Hz, CH_{Ar}), 7.46 - 7.40 (m, 1H, CH_{Ar}), 7.38 (d, 1H, ^3J = 8.3 Hz, CH_{Ar}).

^{13}C NMR (126 MHz, $CDCl_3$): δ 170.4, 148.6, 134.8, 133.0, 127.2, 123.8, 123.0, 120.5 (q, $^1J_{CF}$ = 258.2 Hz, CF_3).

Anal. calcd. for $C_8H_5F_3O_3$: C 46.62; H, 2.45. Found: C, 46.59; H, 2.43.

The ^{1}H, ^{13}C, ^{19}F NMR data of this compaunds is discribed in the literature: W. Zheng, C. A. Morales-Rivera, J. W. Lee, P. Liu, M-Y. Ngai, *Angew. Chemie - Int. Ed.* **2018**, *57*, 9645–9649.

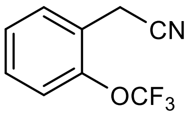

2-(2-(trifluoromethoxy)phenyl)acetonitrile 4m.

The title compound was prepared starting from aniline **1m** (132 mg, 1.0 mmol, 1.0 equiv.), pyrylium tetrafluoroborate (185 mg, 1.1 mmol, 1.1 equiv.), ZrO_2 (123 mg, 1.0 mmol, 1.0 equiv.) and **3c** (318 mg, 1.5 mmol, 1.5 equiv.). The purification was accomplished by column chromatography on silica gel to provide the desired product **4m** (149 mg, 0.74 mmol, 74%).

Colorless liquid, **^{1}H NMR** (500 MHz, $CDCl_3$): δ 7.53 (d, 1H, ^3J = 7.6 Hz, CH_{Ar}), 7.40 - 7.36 (m, 1H, CH_{Ar}), 7.31 (t, 1H, ^3J = 7.2 Hz, CH_{Ar}), 7.28 (d, 1H, ^3J = 8.1 Hz, CH_{Ar}), 3.77 (s, 2H, CH_2).

^{13}C NMR (126 MHz, $CDCl_3$): δ 146.9, 130.0, 129.9, 127.4, 123.0, 120.7, 120.5 (q, $^1J_{CF}$ = 258.6 Hz, CF_3), 116.8, 18.6.

Anal. calcd. for $C_9H_6F_3ON$: C 53.74; H, 3.01; N 6.96. Found: C 53.69; H, 3.11; N 7.01.

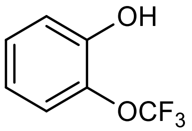

2-(trifluoromethoxy)phenol 4n.

The title compound was prepared starting from aniline **1n** (109 mg, 1.0 mmol, 1.0 equiv.), pyrylium tetrafluoroborate (185 mg, 1.1 mmol, 1.1 equiv.), ZrO_2 (123 mg, 1.0 mmol, 1.0 equiv.) and **3c** (318 mg, 1.5 mmol, 1.5 equiv.). The purification was accomplished by column chromatography on silica gel to provide the desired product **4n** (116 mg, 0.65 mmol, 65%).

Colorless liquid, **^{1}H NMR** (500 MHz, $CDCl_3$): δ 7.23 (d, 1H, ^3J = 8.2 Hz, CH_{Ar}), 7.20 - 7.14 (m, 1H, CH_{Ar}), 7.06 (d, 1H, ^3J = 8.1 Hz, CH_{Ar}), 6.90 (td, 1H, ^3J = 8.2 Hz, ^4J = 1.5 Hz, CH_{Ar}), 5.90 (br s, 1H, OH).

^{13}C NMR (126 MHz, $CDCl_3$): δ 147.9, 136.7, 128.2, 121.6, 121.1, 121.0 (q, $^1J_{CF}$ = 258.7 Hz, CF_3), 117.6.

Anal. calcd. for $C_7H_5F_3O_2$: C 47.21; H, 2.83. Found: C, 47.29; H, 2.85.

The ^{1}H NMR data of this compaunds is discribed in the literature: E. Castagnetti, M. Schlosser, *European J. Org. Chem.* **2001**, 691–695.

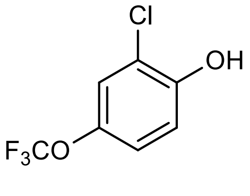

2-chloro-4-(trifluoromethoxy)phenol 4o.

The title compound was prepared starting from aniline **1o** (143 mg, 1.0 mmol, 1.0 equiv.), pyrylium tetrafluoroborate (185 mg, 1.1 mmol, 1.1 equiv.), ZrO_2 (123 mg, 1.0 mmol, 1.0 equiv.) and **3c** (318 mg, 1.5 mmol, 1.5 equiv.). The purification was accomplished by column chromatography on silica gel to provide the desired product **4o** (170 mg, 0.80 mmol, 80%).

Yellowish solid, mp 112-113. **^{1}H NMR** (500 MHz, $CDCl_3$): δ 7.19 (dd, 1H, ^3J = 8.9 Hz, ^4J = 1.1 Hz, CH_{Ar}), 6.96 (d, 1H, ^4J = 3.0 Hz, CH_{Ar}), 6.74 (dd, 1H, ^3J = 8.9 Hz, ^4J = 3.0 Hz, CH_{Ar}), 5.60 (br s, 1H, OH).

^{13}C NMR (126 MHz, $CDCl_3$): δ 154.4, 139.2, 128.5, 124.1, 120.7 (q, $^1J_{CF}$ = 258.3 Hz, CF_3) 117.7, 114.8.

Anal. calcd. for $C_7H_4F_3O_2Cl$: C 39.56; H, 1.90. Found: C, 39.49; H, 1.83.

The ^{1}H NMR data of this compaunds is discribed in the literature: G. Q. Shi, J. F. Dropinski, Y. Zhang, C. Santini, S. P. Sahoo, J. P. Berger, K. L. MacNaul, G. Zhou, A. Agrawal, R. Alvaro, T. Cai, M. Hernandez, S. D. Wright, D. E. Moller, J. V. Heck, P. T. Meinke, *J. Med. Chem.* **2005**, *48*, 5589–5599.

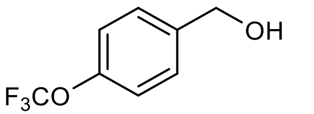

(4-(trifluoromethoxy)phenyl)methanol 4p.

The title compound was prepared starting from aniline **1p** (123 mg, 1.0 mmol, 1.0 equiv.), pyrylium tetrafluoroborate (185 mg, 1.1 mmol, 1.1 equiv.), ZrO_2 (123 mg, 1.0 mmol, 1.0 equiv.) and **3c** (318 mg, 1.5 mmol, 1.5 equiv.). The purification was accomplished by column chromatography on silica gel to provide the desired product **4p** (136 mg, 0.71 mmol, 71%).

Yellowish liquid, **^{1}H NMR** (500 MHz, $CDCl_3$): δ 7.31 (d, 2H, ^3J = 8.5 Hz, CH_{Ar}), 7.17 (d, 2H, ^3J = 8.3 Hz, CH_{Ar}), 4.58 (s, 2H, CH_2), 3.07 (br s, 1H, OH).

^{13}C NMR (126 MHz, $CDCl_3$): δ 148.7, 139.5, 128.3, 121.1, 120.6 (q, $^1J_{CF}$ = 256.9 Hz, CF_3), 64.2.

Anal. calcd. for $C_8H_7F_3O_2$: C 50.01; H, 3.67. Found: C, 50.09; H, 3.61.

The ^{1}H, ^{13}C, ^{19}F NMR data of this compaunds is discribed in the literature: K. Zhu, M. P. Shaver, S. P. Thomas, *European J. Org. Chem.* **2015**, 2119–2123.

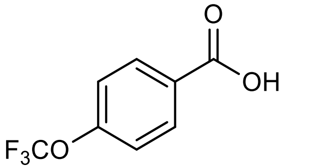

4-(trifluoromethoxy)benzoic acid 4q.

The title compound was prepared starting from aniline **1q** (137 mg, 1.0 mmol, 1.0 equiv.), pyrylium tetrafluoroborate (185 mg, 1.1 mmol, 1.1 equiv.), ZrO_2 (123 mg, 1.0 mmol, 1.0 equiv.) and **3c** (318 mg, 1.5 mmol, 1.5 equiv.). The purification was accomplished by column chromatography on silica gel to provide the desired product **4q** (169 mg, 0.82 mmol, 82%).
White solid, mp 152-153 °C **^{1}H NMR** (500 MHz, DMSO-d_6): δ 13.23 (br s, 1H, COOH), 8.07 - 8.00 (m, 2H, CH_{Ar}), 7.47 - 7.37 (m, 2H, CH_{Ar}).
^{13}C NMR (126 MHz, DMSO-d_6): δ 166.0, 151.2, 131.5, 129.6, 120.4, 119.7 (q, $^1J_{CF}$ = 257.5 Hz, CF_3).
MS (GC, 70eV): m/z (%) = 206 (M^+, 66), 189 (100), 161 (22), 95 (35).
Anal. calcd. for $C_8H_5F_3O_3$: C 46.62; H, 2.45. Found: C, 46.69; H, 2.39.
The ^{1}H, ^{13}C, ^{19}F NMR data of this compaunds is discribed in the literature: K.-J. Liu, S. Jiang, L.-H. Lu, L-L. Tang, S.-S. Tang, H.-S. Tang, Z. Tang, W.-M. He. X. Xu, *Green Chem.* **2018**, *20*, 3038–3043.

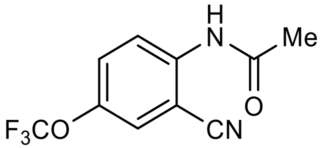

N-(2-cyano-4-(trifluoromethoxy)phenyl)acetamide 4r.
The title compound was prepared starting from aniline **1r** (175 mg, 1.0 mmol, 1.0 equiv.), pyrylium tetrafluoroborate (185 mg, 1.1 mmol, 1.1 equiv.), ZrO_2 (123 mg, 1.0 mmol, 1.0 equiv.) and **3c** (318 mg, 1.5 mmol, 1.5 equiv.). The purification was accomplished by column chromatography on silica gel to provide the desired product **4r** (210 mg, 0.86 mmol, 86%).
White solid, mp 137-139 **^{1}H NMR** (500 MHz, $CDCl_3$): δ 8.62 (d, 1H, ^3J = 8.7 Hz, CH_{Ar}), 7.86 (br s, 1H, NH), 7.55 (d, 1H, ^3J = 8.7 Hz, CH_{Ar}), 7.52 - 7.50 (m, 1H, CH_{Ar}), 2.26 (s, 3H, CH_3).
^{13}C NMR (126 MHz, $CDCl_3$): δ 168.9, 137.2, 135.1, 131.8, 123.8, 121.9, 120.4 (q, $^1J_{CF}$ = 261.4 Hz, CF_3), 117.6, 106.9, 25.0.
MS (GC, 70eV): m/z (%) = 244 (M^+, 29), 202 (100), 182 (13), 162 (20), 133 (35).
Anal. calcd. for $C_{10}H_7F_3O_2N_2$: C 49.19; H, 2.89; N 11.47. Found: C 49.21; H, 3.01; N 11.39.

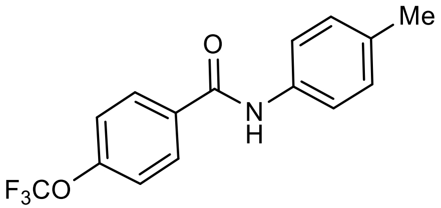

N-(p-tolyl)-4-(trifluoromethoxy)benzamide 4s.

The title compound was prepared starting from aniline **1s** (226 mg, 1.0 mmol, 1.0 equiv.), pyrylium tetrafluoroborate (185 mg, 1.1 mmol, 1.1 equiv.), ZrO_2 (123 mg, 1.0 mmol, 1.0 equiv.) and **3c** (318 mg, 1.5 mmol, 1.5 equiv.). The purification was accomplished by column chromatography on silica gel to provide the desired product **4s** (263 mg, 0.89 mmol, 89%).

White solid, mp 94 -95 °C **^{1}H NMR** (500 MHz, DMSO-d_6): δ 10.28 (s, 1H, NH), 8.07 (d, 2H, ^3J = 9.1 Hz, CH_{Ar}), 7.64 (d, 2H, ^3J = 9.1 Hz, CH_{Ar}), 7.51 (d, 2H, ^3J = 9.1 Hz, CH_{Ar}), 7.15 (d, 2H, ^3J = 7.6 Hz, CH_{Ar}), 2.28 (s, 3H, Me).

^{13}C NMR (126 MHz, DMSO-d_6): δ 164.1, 150.4, 136.5, 134.2, 132.9, 130.0, 129.1, 120.7, 120.4, 120.0 (q, $^1J_{CF}$ = 256.9 Hz, OCF_3), 20.5.

HRMS (TOF MS ES+) m/z: $[M + H]^+$: Calcd for $C_{15}H_{13}NO_2F_3$ 296.0902. Found 296.0898.

MS (GC, 70eV): m/z (%) = 244 (M^+, 29), 202 (100), 182 (13), 162 (20), 133 (35).

Anal. calcd. for $C_{10}H_7F_3O_2N_2$: C 49.19; H, 2.89; N 11.47. Found: C 49.21; H, 3.01; N 11.39.

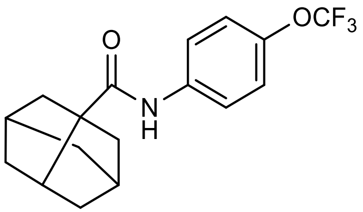

N-(4-(trifluoromethoxy)phenyl)adamantane-1-carboxamide 4t.

The title compound was prepared starting from aniline **1t** (270 mg, 1.0 mmol, 1.0 equiv.), pyrylium tetrafluoroborate (185 mg, 1.1 mmol, 1.1 equiv.), ZrO_2 (123 mg, 1.0 mmol, 1.0 equiv.) and **3c** (318 mg, 1.5 mmol, 1.5 equiv.). The purification was accomplished by column chromatography on silica gel to provide the desired product **4t** (308 mg, 0.91 mmol, 91%).

White solid, mp 164-165 °C **^{1}H NMR** (500 MHz, $CDCl_3$): δ 7.57 (d, 2H, ^3J = 9.0 Hz, CH_{Ar}), 7.41 (br s, 1H, NH), 7.15 (d, 2H, ^3J = 8.7 Hz, CH_{Ar}), 2.11 - 2.07 (m, 3H, CH), 1.95 (d, 6H, ^4J = 2.3 Hz, CH_2), 1.80 - 1.69 (m, 6H, CH_2).

^{13}C NMR (126 MHz, $CDCl_3$): δ 176.3, 145.2, 136.9, 121.8, 121.3, 120.6 (q, $^1J_{CF}$ = 256.6 Hz, CF_3), 41.6, 39.3, 36.5, 28.2.

MS (GC, 70eV): m/z (%) = 339 (M^+, 29), 135 (100).

Anal. calcd. for $C_{18}H_{20}F_3O_2N$: C 63.71; H, 5.94; N 4.13. Found: C 63.65; H, 5.91; N 4.01.

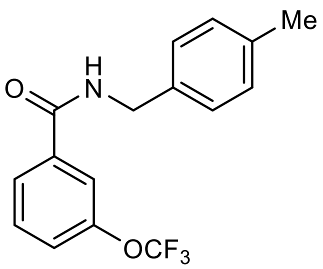

***N*-(4-methylbenzyl)-3-(trifluoromethoxy)benzamide 4u.**

The title compound was prepared starting from aniline **1u** (240 mg, 1.0 mmol, 1.0 equiv.), pyrylium tetrafluoroborate (185 mg, 1.1 mmol, 1.1 equiv.), ZrO_2 (123 mg, 1.0 mmol, 1.0 equiv.) and **3c** (318 mg, 1.5 mmol, 1.5 equiv.). The purification was accomplished by column chromatography on silica gel to provide the desired product **4u** (272 mg, 0.88 mmol, 88%).

White solid, mp 106-107 °C **^{1}H NMR** (500 MHz, $CDCl_3$): δ 7.68 – 7.67 (m, 2H, CH_{Ar}), 7.41 (t, 1H, ^3J = 8.1 Hz, CH_{Ar}), 7.32 (d, 1H, ^3J = 8.1 Hz, CH_{Ar}), 7.20 (d, 2H, ^3J = 8.1 Hz, CH_{Ar}), 7.13 (d, 2H, ^3J = 8.1 Hz, CH_{Ar}), 6.82 (br. s, 1H, NH), 4.54 (d, 2H, ^3J = 5.4 Hz, CH_2), 2.33 (s, 3H, Me).

^{13}C NMR (126 MHz, $CDCl_3$): δ 165.8, 149.3, 137.4, 136.5, 134.8, 130.0, 139.4, 127.9, 125.1, 123.7, 120.3 (q, $^1J_{CF}$ = 260.3 Hz, CF_3), 120.0, 43.9, 21.0.

MS (GC, 70eV): m/z (%) = 309 (M^+, 60), 294 (13), 189 (100), 161 (16), 120 (21).

HRMS (TOF MS ES+) m/z: $[M + H]^+$: Calcd for $C_{16}H_{15}NO_2F_3$ 310.1060. Found 310.1055.

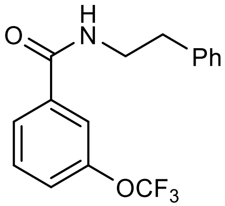

***N*-phenethyl-3-(trifluoromethoxy)benzamide 4v.**

The title compound was prepared starting from aniline **1v** (240 mg, 1.0 mmol, 1.0 equiv.), pyrylium tetrafluoroborate (185 mg, 1.1 mmol, 1.1 equiv.), ZrO_2 (123 mg, 1.0 mmol, 1.0 equiv.) and **3c** (318 mg, 1.5 mmol, 1.5 equiv.). The purification was accomplished by column chromatography on silica gel to provide the desired product **4v** (192 mg, 0.62 mmol, 62%).

White solid, mp 85-86 °C **^{1}H NMR** (500 MHz, DMSO-*d6*): δ 8.78 (s, 1H, NH), 7.88 – 7.87 (m, 1H, CH_{Ar}), 7.78 (s, 1H, CH_{Ar}), 7.61 (t, 1H, ^3J = 7.9 Hz, CH_{Ar}), 7.53 – 7.52 (m, 1H, CH_{Ar}), 7.31 – 7.28 (m, 2H, CH_{Ar}), 7.25 – 7.24 (m, 2H, CH_{Ar}), 7.21 – 7.18 (m, 1H, CH_{Ar}), 3.51 – 3.50 (m, 2H, CH_2), 2.86 (t, 2H, ^3J = 7.0 Hz, CH_{Ar}).

^{13}C NMR (126 MHz, DMSO-*d6*): δ 164.5, 148.3, 139.4, 136.8, 130.5, 128.7, 128.3, 126.2, 126.1, 123.7, 120.1 (q, $^1J_{CF}$ = 256.6 Hz, CF_3), 119.7, 41.0, 35.0.

MS (GC, 70eV): m/z (%) = 309 (M^+, 17), 189 (100), 161 (16), 104 (99).

Anal. calcd. for $C_{16}H_{14}F_3O_2N$: C 62.13; H, 4.56; N 4.53. Found: C 62.33; H, 4.51; N 4.49.

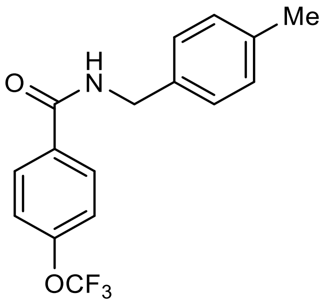

N-(4-methylbenzyl)-4-(trifluoromethoxy)benzamide 4w.

The title compound was prepared starting from aniline **1w** (240 mg, 1.0 mmol, 1.0 equiv.), pyrylium tetrafluoroborate (185 mg, 1.1 mmol, 1.1 equiv.), ZrO_2 (123 mg, 1.0 mmol, 1.0 equiv.) and **3c** (318 mg, 1.5 mmol, 1.5 equiv.). The purification was accomplished by column chromatography on silica gel to provide the desired product **4w** (244 mg, 0.79 mmol, 79%).

White solid, mp 158-159 ^oC **^{1}H NMR** (500 MHz, DMSO-d_6): δ 9.13 (t, 1H, ^3J = 5.9 Hz, NH), 8.03 – 8.01 (m, 2H, CH_{Ar}), 7.46 (d, 2H, ^3J = 8.0 Hz, CH_{Ar}), 7.20 (d, 2H, ^3J = 8.0 Hz, CH_{Ar}), 7.12 (d, 2H, ^3J = 8.0 Hz, CH_{Ar}), 4.44 (d, 2H, ^3J = 5.9 Hz, CH_2), 2.27 (s, 3H, Me).

^{13}C NMR (126 MHz, DMSO-d_6): δ 164.9, 150.3, 136.4, 135.8, 133.9, 129.6, 128.9, 127.2, 120.7, 120.0 (q, $^1J_{CF}$ = 258.7 Hz, CF_3), 42.5, 20.7.

MS (GC, 70eV): m/z (%) = 309 (M^+, 52), 189 (100), 161 (11), 120 (17).

Anal. calcd. for $C_{16}H_{14}F_3O_2N$: C 62.13; H, 4.56; N 4.53. Found: C 62.20; H, 4.49; N 4.61.

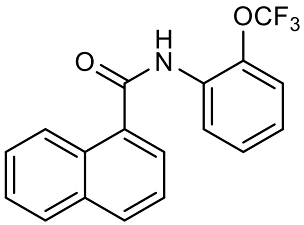

N-(2-(trifluoromethoxy)phenyl)-1-naphthamide 4x.

The title compound was prepared starting from aniline **1x** (262 mg, 1.0 mmol, 1.0 equiv.), pyrylium tetrafluoroborate (185 mg, 1.1 mmol, 1.1 equiv.), ZrO_2 (123 mg, 1.0 mmol, 1.0 equiv.) and **3c 3c** (318 mg, 1.5 mmol, 1.5 equiv.). The purification was accomplished by column chromatography on silica gel to provide the desired product **4x** (215 mg, 0.65 mmol, 65%).

White solid, mp 116-117 °C **^{1}H NMR** (500 MHz, $CDCl_3$): δ 8.67 (d, 1H, ^3J = 7.0 Hz, CH_{Ar}), 8.42 (d, 1H, ^3J = 8.2 Hz, CH_{Ar}), 8.07 (s, 1H, NH), 8.01 (d, 1H, ^3J = 8.2 Hz, CH_{Ar}), 7.92 (d, 1H, ^3J = 7.6 Hz, CH_{Ar}), 7.67 (d, 1H, ^3J = 7.2 Hz, CH_{Ar}), 7.63 – 7.52 (m, 3H, CH_{Ar}), 7.40 (t, 1H, ^3J = 7.7 Hz, CH_{Ar}), 7.32 (d, 1H, ^3J = 8.2 Hz, CH_{Ar}), 7.21 – 7.18 (m, 1H, CH_{Ar}).

^{13}C NMR (126 MHz, $CDCl_3$): δ 167.4, 138.5, 133.8, 131.5, 130.7, 130.1, 128.5, 127.6, 127.5, 126.7, 125.3, 125.1, 124.8, 124.6, 122.2, 120.6 (q, $^1J_{CF}$ = 259.7 Hz, CF_3), 120.5.

MS (GC, 70eV): m/z (%) = 331 (M^+, 11), 155 (100), 127 (66).

HRMS (TOF MS ES+) m/z: $[M + H]^+$: Calcd for $C_{18}H_{13}NO_2F_3$ 332.0909. Found 332.0898.

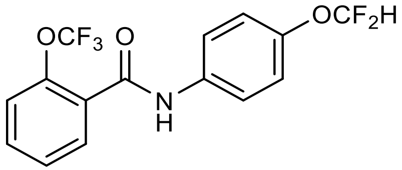

N-(4-(difluoromethoxy)phenyl)-2-(trifluoromethoxy)benzamide 4y.

The title compound was prepared starting from aniline **1y** (278 mg, 1.0 mmol, 1.0 equiv.), pyrylium tetrafluoroborate (185 mg, 1.1 mmol, 1.1 equiv.), ZrO_2 (123 mg, 1.0 mmol, 1.0 equiv.) and **3c** (318 mg, 1.5 mmol, 1.5 equiv.). The purification was accomplished by column chromatography on silica gel to provide the desired product **4y** (212 mg, 0.61 mmol, 61%).

White solid, mp 117-118 °C **^{1}H NMR** (500 MHz, $CDCl_3$): δ 8.44 (br s, 1H, NH), 7.99 (d, 1H, ^3J = 7.1 Hz, CH_{Ar}), 7.60 (d, 2H, ^3J = 9.2 Hz, CH_{Ar}), 7.53 (t, 1H, ^3J = 7.2 Hz, CH_{Ar}), 7.41 (t, 1H, ^3J = 7.9 Hz, CH_{Ar}), 7.31 (d, 1H, ^3J = 8.2 Hz, CH_{Ar}), 7.11 (d, 2H, ^3J = 8.2 Hz, CH_{Ar}), 6.49 (t, 1H, ^1J = 73.6 Hz, OCF_2H).

^{13}C NMR (126 MHz, $CDCl_3$): δ 162.4, 147.7, 147.7, 135.0, 132.8, 131.7, 128.0, 127.6, 121.8, 121.2, 120.4, 120.3 (q, $^1J_{CF}$ = 259.8 Hz, CF_3), 115.9 (t, $^1J_{CF}$ = 261.4 Hz, OCF_2H).

MS (GC, 70eV): m/z (%) = 347 (M^+, 30), 189 (100), 123 (36).

HRMS (TOF MS ES+) m/z: $[M + H]^+$: Calcd for $C_{15}H_{11}NO_3F_5$ 348.0661. Found 348.0659.

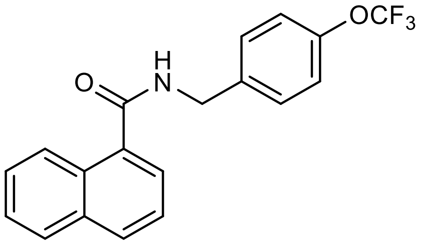

N-(4-(trifluoromethoxy)benzyl)-1-naphthamide 4z.

The title compound was prepared starting from aniline **1z** (276 mg, 1.0 mmol, 1.0 equiv.), pyrylium tetrafluoroborate (185 mg, 1.1 mmol, 1.1 equiv.), ZrO_2 (123 mg, 1.0 mmol, 1.0 equiv.) and **3c** (318 mg, 1.5 mmol, 1.5 equiv.). The purification was accomplished by column chromatography on silica gel to provide the desired product **4z** (269 mg, 0.78 mmol, 78%).

White solid, mp 122-123 °C **^{1}H NMR** (500 MHz, $CDCl_3$): δ 8.19 (d, 1H, ^3J = 8.1 Hz, CH_{Ar}), 7.84 (t, 2H, ^3J = 7.2 Hz, CH_{Ar}), 7.52 – 7.44 (m, 3H, CH_{Ar}), 7.31 – 7.24 (m, 3H, CH_{Ar}), 7.12 (d, 2H, ^3J = 7.9 Hz, CH_{Ar}), 7.09 (br. s, 1H, NH).

^{13}C NMR (126 MHz, $CDCl_3$): δ 169.5, 148.3, 137.0, 133.8, 133.5, 130.6, 130.0, 128.9, 128.2, 127.0, 126.3, 125.2, 124.9, 124.5, 121.0, 120.4 (q, $^1J_{CF}$ = 260.6 Hz, CF_3), 42.9.

MS (GC, 70eV): m/z (%) = 345 (M^+, 64), 155 (100), 127 (90).

HRMS (TOF MS ES+) m/z: $[M + H]^+$: Calcd for $C_{19}H_{15}NO_2F_3$ 346.1060. Found 346.1055.

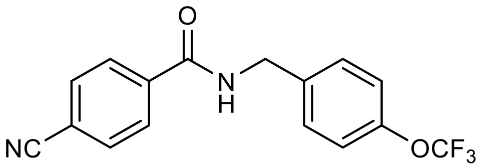

4-cyano-N-(4-(trifluoromethoxy)benzyl)benzamide 4aa.

The title compound was prepared starting from aniline **1aa** (251 mg, 1.0 mmol, 1.0 equiv.), pyrylium tetrafluoroborate (185 mg, 1.1 mmol, 1.1 equiv.), ZrO_2 (123 mg, 1.0 mmol, 1.0 equiv.) and **3c** (318 mg, 1.5 mmol, 1.5 equiv.). The purification was accomplished by column chromatography on silica gel to provide the desired product **4aa** (259 mg, 0.81 mmol, 81%).

White solid, mp 97-98 °C **^{1}H NMR** (500 MHz, $CDCl_3$): δ 7.86 (d, 2H, ^3J = 8.3 Hz, CH_{Ar}), 7.66 (d, 2H, ^3J = 8.3 Hz, CH_{Ar}), 7.31 (d, 2H, ^3J = 8.5 Hz, CH_{Ar}), 7.15 (d, 2H, ^3J = 8.2 Hz, CH_{Ar}), 7.07 (br.s, 1H, NH), 4.58 (d, 2H, ^3J = 6.1 Hz, CH_2).

^{13}C NMR (126 MHz, $CDCl_3$): δ 165.8, 148.7, 138.0, 136.5, 132.5, 129.3, 127.8, 121.3, 120.4 (q, $^1J_{CF}$ = 256.3 Hz, CF_3), 118.0, 115.2, 43.5.

MS (GC, 70eV): m/z (%) = 320 (M^+, 76), 235 (16), 190 (24), 130 (100), 102 (61).

Anal. calcd. for $C_{16}H_{11}F_3O_2N_2$: C 60.00; H, 3.46; N 8.75. Found: C 60.09; H, 3.52; N 8.63.
The ^{1}H, ^{13}C NMR data of this compaunds is discribed in the literature: S. Mkrtchyan, V. Iaroshenko, *Chem. Commun.* **2021**, *57*, 11029–11032.

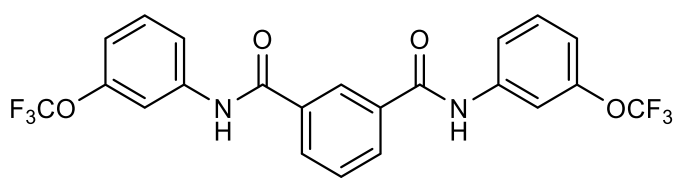

N1,N3-bis(3-(trifluoromethoxy)phenyl)isophthalamide 4ab.

The title compound was prepared starting from aniline **1x** (346 mg, 1.0 mmol, 1.0 equiv.), pyrylium tetrafluoroborate (185 mg, 1.1 mmol, 1.1 equiv.), ZrO_2 (123 mg, 1.0 mmol, 1.0 equiv.) and **3c** (466 mg, 2.2 mmol, 2.2 equiv.). The purification was accomplished by column chromatography on silica gel to provide the desired product **4ab** (358 mg, 0.74 mmol, 74%).

White solid, mp 117-118 °C **^{1}H NMR** (500 MHz, DMSO-*d$_6$*): δ 10.71 (br s, 2H, NH), 8.58 (s, 1H, CH_{Ar}), 8.18 (d, 2H, ^3J = 7.9 Hz, CH_{Ar}), 7.99 (s, 1H, CH_{Ar}), 7.81 (d, 1H, ^3J = 8.5 Hz, CH_{Ar}), 7.73 (t, 1H, ^3J = 7.9 Hz, CH_{Ar}), 7.51 (t, 2H, ^3J = 7.9 Hz, CH_{Ar}), 7.11 (d, 2H, ^3J = 8.9 Hz, CH_{Ar}).

^{13}C NMR (126 MHz, DMSO-*d$_6$*): δ 165.4, 148.5, 140.8, 134.8, 131.1, 130.4, 128.8, 127.2, 120.2 (q, $^1J_{CF}$ = 256.3 Hz, *C*F$_3$), 118.9, 115.9, 112.4.

MS (GC, 70eV): m/z (%) = 484 (M^+, 31), 308 (100), 254 (19), 242 (16), 222 (16), 104 (28).

HRMS (TOF MS ES+) m/z: $[M + H]^+$: Calcd for $C_{22}H_{15}N_2O_4F_6$ 485.0935. Found 485.0936.

The ^{1}H, ^{13}C NMR data of this compaunds is discribed in the literature: S. Mkrtchyan, V. Iaroshenko, *Chem. Commun.* **2021**, *57*, 11029–11032.

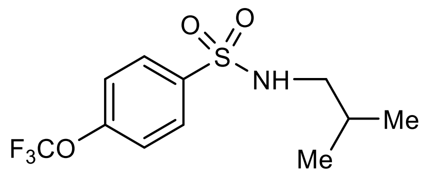

N-isobutyl-4-(trifluoromethoxy)benzenesulfonamide 4ac.

The title compound was prepared starting from aniline **1ac** (228 mg, 1.0 mmol, 1.0 equiv.), pyrylium tetrafluoroborate (185 mg, 1.1 mmol, 1.1 equiv.), ZrO_2 (123 mg, 1.0 mmol, 1.0 equiv.) and **3c** (466 mg, 2.2 mmol, 2.2 equiv.). The purification was accomplished by column chromatography on silica gel to provide the desired product **4ac** (220 mg, 0.74 mmol, 74%).

White solid, mp 69-70 °C **^{1}H NMR** (500 MHz, DMSO-*d$_6$*): δ 7.91 (d, 2H, ^3J = 8.7 Hz, CH_{Ar}), 7.76 (t, 1H, ^3J = 6.2 Hz, NH), 7.56 (d, 2H, ^3J = 7.2 Hz, CH_{Ar}), 2.56 (t, 2H, ^3J = 7.2 Hz, CH_2), 1.63 – 1.61 (m, 1H, CH), 0.79 (d, 6H, CH_3).

^{13}C NMR (126 MHz, DMSO-*d$_6$*): δ 150.7, 139.8, 129.0, 121.4, 119.9 (q, $^1J_{CF}$ = 265.4 Hz, *C*F$_3$), 50.0, 28.1, 19.8.

MS (GC, 70eV): m/z (%) = 297 (M^+, 4), 254 (99), 225 (100), 161 (62), 95 (44).
Anal. calcd. for $C_{11}H_{14}F_3O_3NS$: C 44.44; H, 4.75; N 4.71. Found: C 44.52; H, 4.71; N 4.77.

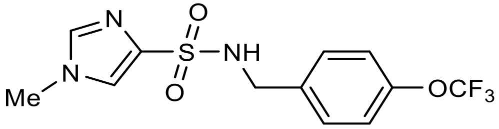

1-methyl-N-(4-(trifluoromethoxy)benzyl)-1H-imidazole-4-sulfonamide 4ad.
The title compound was prepared starting from aniline **1ad** (335 mg, 1.0 mmol, 1.0 equiv.), pyrylium tetrafluoroborate (185 mg, 1.1 mmol, 1.1 equiv.), ZrO_2 (123 mg, 1.0 mmol, 1.0 equiv.) and **3c** (466 mg, 2.2 mmol, 2.2 equiv.). The purification was accomplished by column chromatography on silica gel to provide the desired product **4ad** (171 mg, 0.51 mmol, 51%).
White solid, mp 170-171 °C **^{1}H NMR** (500 MHz, DMSO-*d6*): δ 8.07 (s, 1H, NH), 7.76 (s, 1H, Imidazole), 7.71 (s, 1H, Imidazole), 7.39 (d, 2H, ^3J = 10.0 Hz, CH_{Ar}), 7.27 (d, 2H, ^3J = 7.8 Hz, CH_{Ar}), 4.07 (s, 2H, CH_2), 3.68 (s, 3H, Me).
^{13}C NMR (126 MHz, DMSO-*d6*): δ 147.3, 139.7, 139.6, 137.9, 129.4, 124.2, 120.7, 120.1 (q, $^1J_{CF}$ = 255.7 Hz, CF_3), 45.3, 33.4.
MS (GC, 70eV): m/z (%) = 335 (M^+, 1), 190 (75), 146 (12), 82 (100).
Anal. calcd. for $C_{12}H_{12}F_3O_3N_3S$: C 42.99; H, 3.61; N 12.53. Found: C 46.05; H, 3.53; N 12.49.

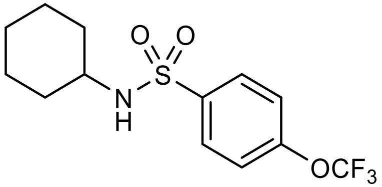

N-cyclohexyl-4-(trifluoromethoxy)benzenesulfonamide 4ae.
The title compound was prepared starting from aniline **1ae** (254 mg, 1.0 mmol, 1.0 equiv.), pyrylium tetrafluoroborate (185 mg, 1.1 mmol, 1.1 equiv.), ZrO_2 (123 mg, 1.0 mmol, 1.0 equiv.) and **3c** (318 mg, 1.5 mmol, 1.5 equiv.). The purification was accomplished by column chromatography on silica gel to provide the desired product **4ae** (249 mg, 0.77 mmol, 77%).
White solid, mp 65-66 °C **^{1}H NMR** (500 MHz, $CDCl_3$): δ 7.95 (dt, 2H, ^3J = 8.8 Hz, ^4J = 2.7 Hz, CH_{Ar}), 7.32 (d, 2H, ^3J = 8.1 Hz, CH_{Ar}), 5.13 (br s, 1H, NH), 3.13 (br. s 1H, Cy), 1.75 – 1.73 (m, 2H, Cy), 1.64 – 1.64 (m, 2H, Cy), 1.52 – 1.48 (m, 1H, Cy), 1.24 – 1.09 (m, 5H, Cy).
^{13}C NMR (126 MHz, $CDCl_3$): δ 151.9, 139.9, 129.0, 120.9, 120.2 (q, $^1J_{CF}$ = 251.5 Hz, CF_3), 52.7, 33.8, 25.0, 24.5.
MS (GC, 70eV): m/z (%) = 323 (M^+, 44), 280 (99), 225 (100), 161 (94).
HRMS (TOF MS ES+) m/z: $[M + H]^+$: Calcd for $C_{13}H_{17}NO_3F_3S$ 324.0884. Found 324.0881.

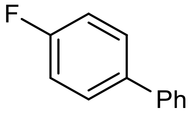

4-fluoro-1,1'-biphenyl 5.

The title compound was prepared starting from [1,1'-biphenyl]-4-amine (169 mg, 1.0 mmol, 1.0 equiv.), pyrylium tetrafluoroborate (185 mg, 1.1 mmol, 1.1 equiv.), ZrO_2 (123 mg, 1.0 mmol, 1.0 equiv.). The purification was accomplished by column chromatography on silica gel to provide the desired product **5** (120 mg, 0.70 mmol, 70%).

White solid, mp 75-77 °C **^{1}H NMR** (500 MHz, $CDCl_3$): δ 7.95 (dt, 2H, ^3J = 8.8 Hz, ^4J = 2.7 Hz, CH_{Ar}), 7.32 (d, 2H, ^3J = 8.1 Hz, CH_{Ar}), 5.13 (br s, 1H, NH), 3.13 (br. s 1H, Cy), 1.75 – 1.73 (m, 2H, Cy), 1.64 – 1.64 (m, 2H, Cy), 1.52 – 1.48 (m, 1H, Cy), 1.24 – 1.09 (m, 5H, Cy).

^{13}C NMR (126 MHz, $CDCl_3$): δ 151.9, 139.9, 129.0, 120.9, 120.2 (q, $^1J_{CF}$ = 251.5 Hz, CF_3), 52.7, 33.8, 25.0, 24.5.

MS (GC, 70eV): m/z (%) = 172 (M^+, 100).

HRMS (TOF MS ES+) m/z: $[M + H]^+$: Calcd for $C_{12}H_9F$ 172.0690. Found 172.0688.

Anal. calcd. for $C_{12}H_9F$: C 83.70; H, 5.27. Found: C 83.63; H, 5.31.

The ^{1}H, ^{13}C, ^{19}F NMR data of this compaunds is discribed in the literature: H. Yue, L. Guo, S.-C. Lee, X. Liu, M. Rueping, *Angew. Chemie - Int. Ed.* **2017**, *56*, 3972–3976.

(C) Copies ^{1}H and ^{13}C NMR spectra

Compound **4a**

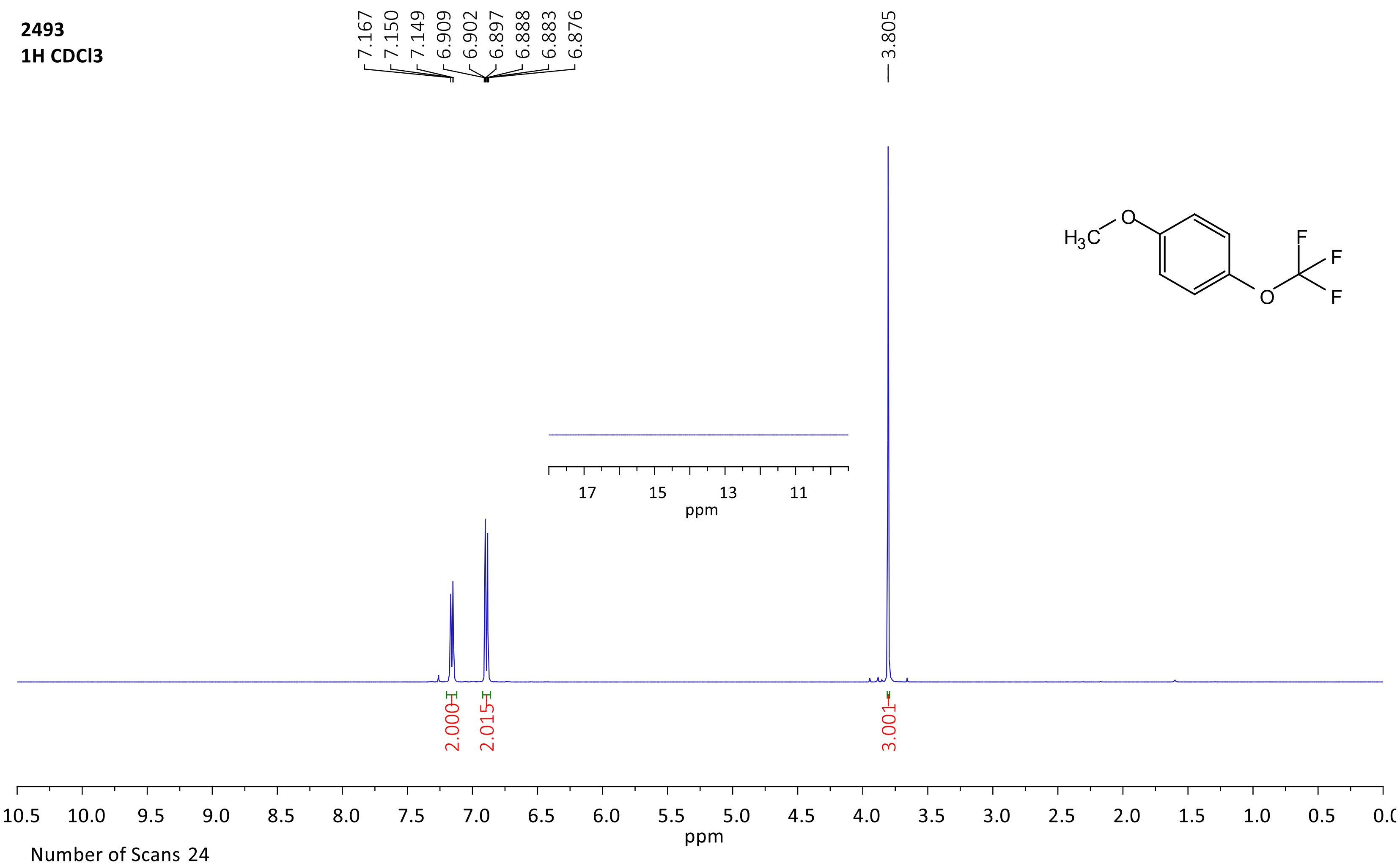

Number of Scans 24
Spectromet. Freq.500.13
Spectral Width 12335.5
Spectral Size 65536
freq. of 0 ppm: 500.1300236

Compound **4a**

2493
13C CDCl3

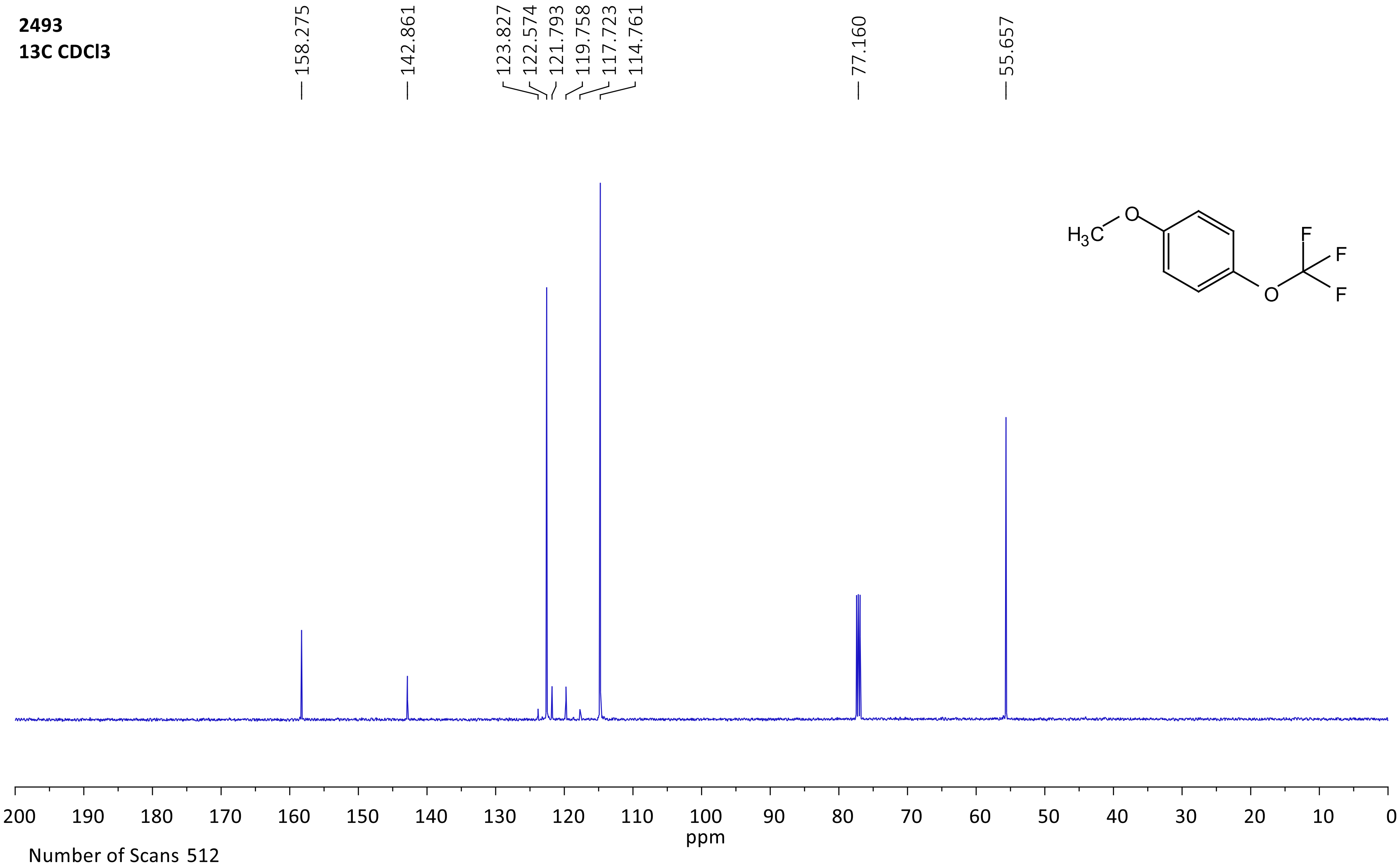

Number of Scans 512
Spectromet. Freq.125.76
Spectral Width 36057.7
Spectral Size 65536
freq. of 0 ppm: 125.7577890

Compound **4b**

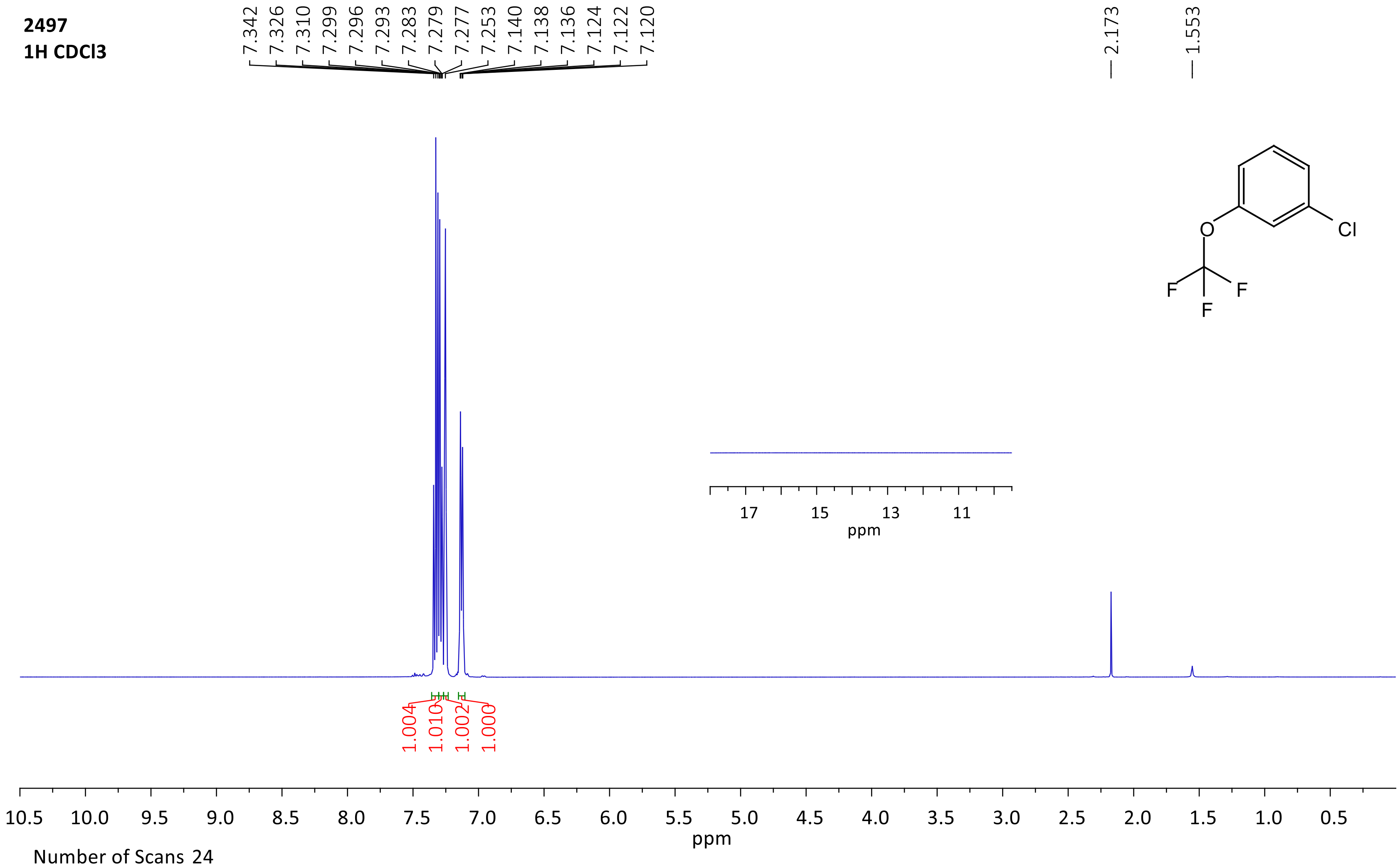

Number of Scans 24

Spectromet. Freq.500.13

Spectral Width 12335.5

Spectral Size 65536

freq. of 0 ppm: 500.1300236

Compound **4b**

2497
13C CDCl3

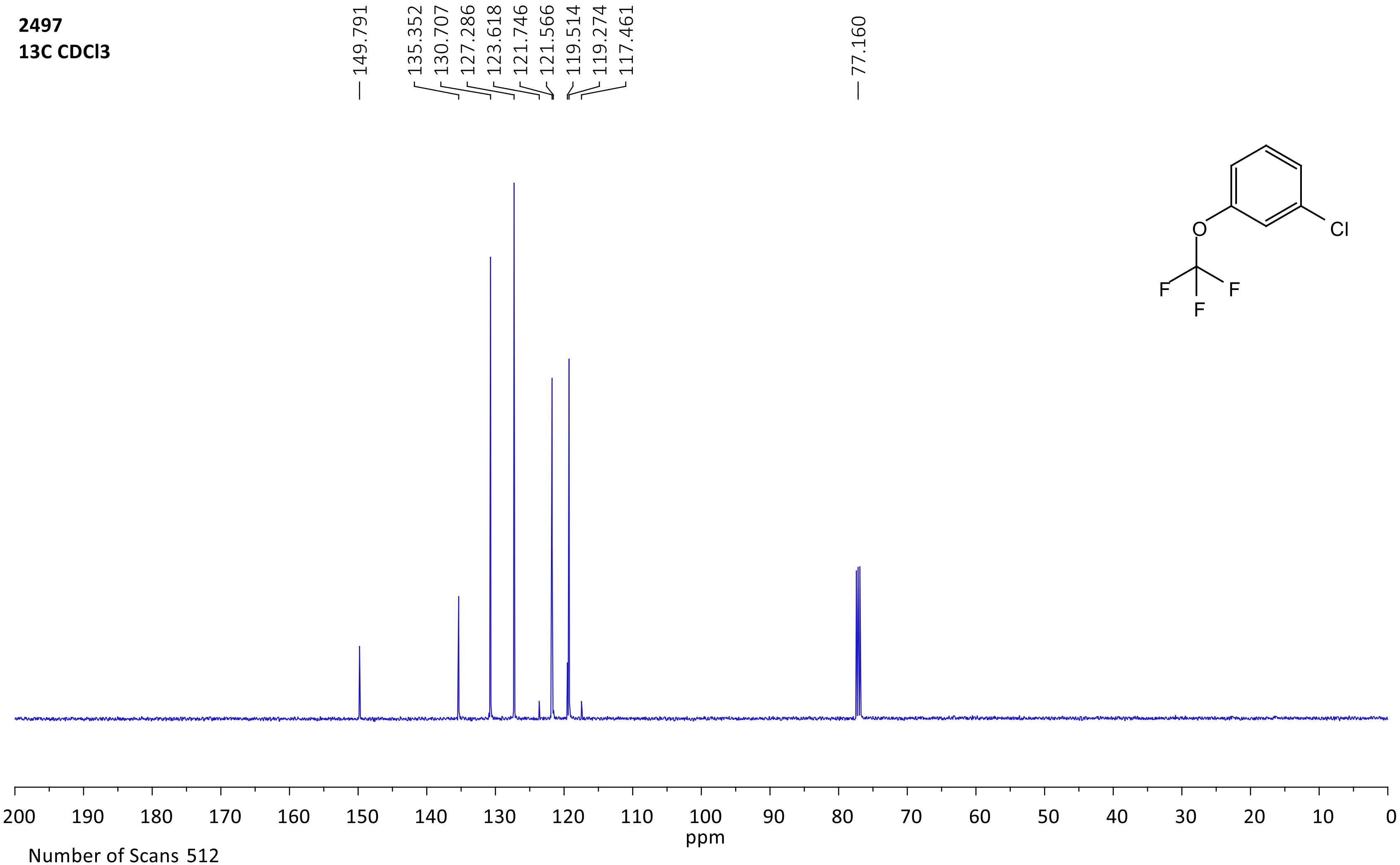

Number of Scans 512
Spectromet. Freq.125.76
Spectral Width 36057.7
Spectral Size 65536
freq. of 0 ppm: 125.7577890

Compound **4c**

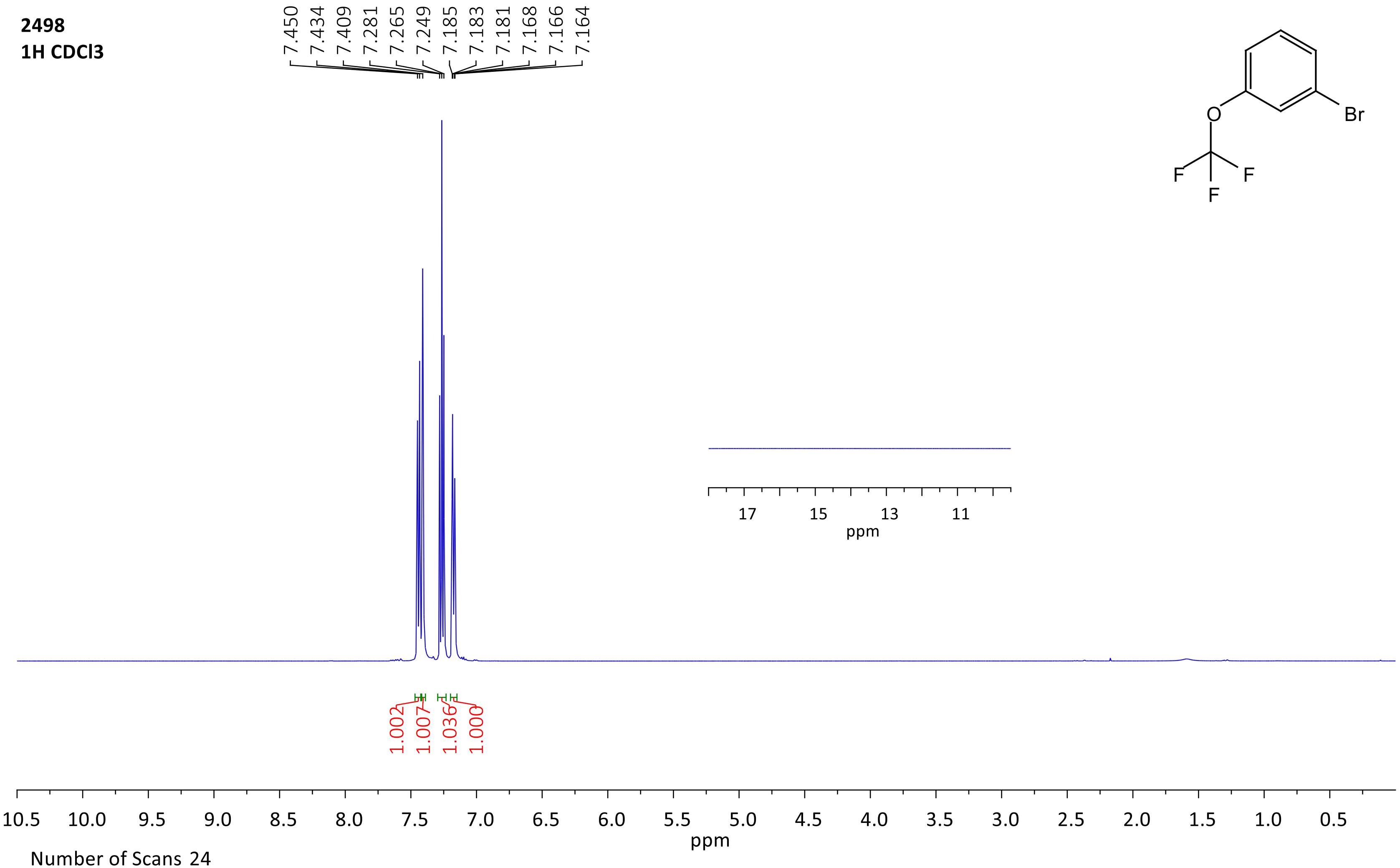

Number of Scans 24
Spectromet. Freq.500.13
Spectral Width 12335.5
Spectral Size 65536
freq. of 0 ppm: 500.1300236

Compound **4c**

2498
13C CDCl3

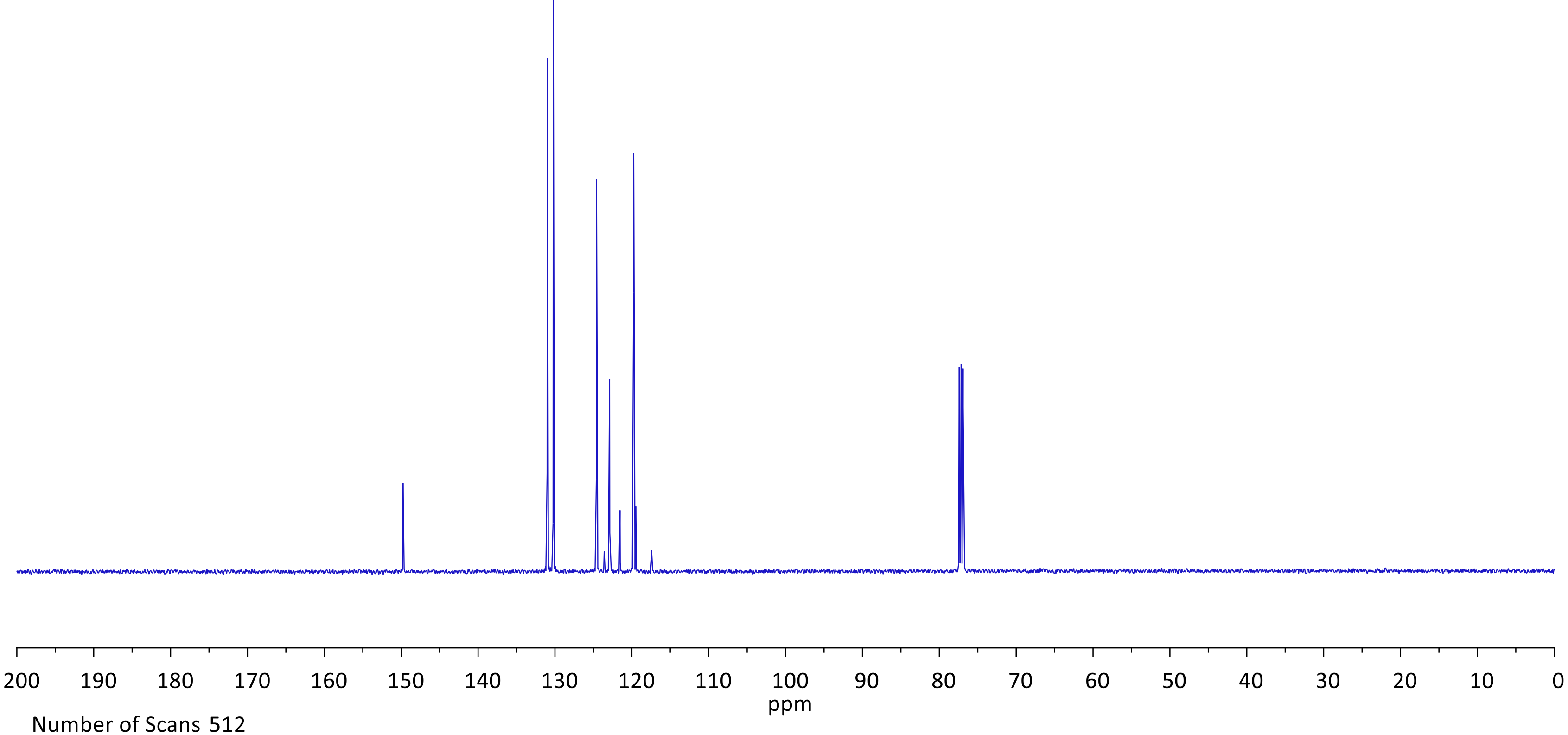

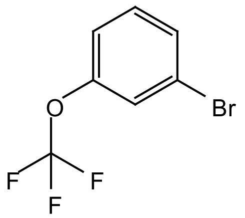

Number of Scans 512
Spectromet. Freq.125.76
Spectral Width 36057.7
Spectral Size 65536
freq. of 0 ppm: 125.7577890

Compound **4d**

IVA 2253
1H CDCl3

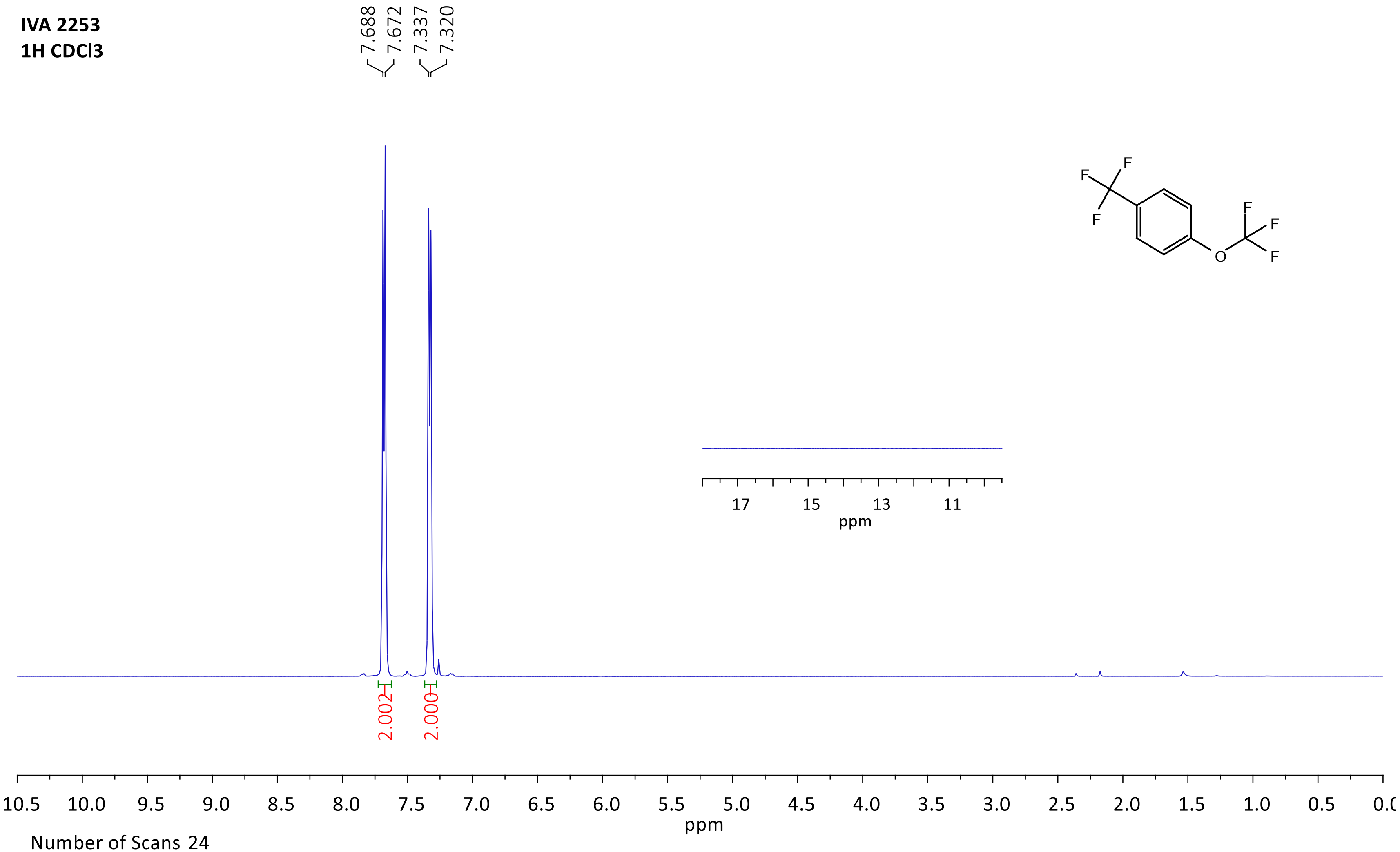

Number of Scans 24
Spectromet. Freq.500.13
Spectral Width 12335.5
Spectral Size 65536
freq. of 0 ppm: 500.1300236

IVA 2253
13C CDCl3

Compound **4d**

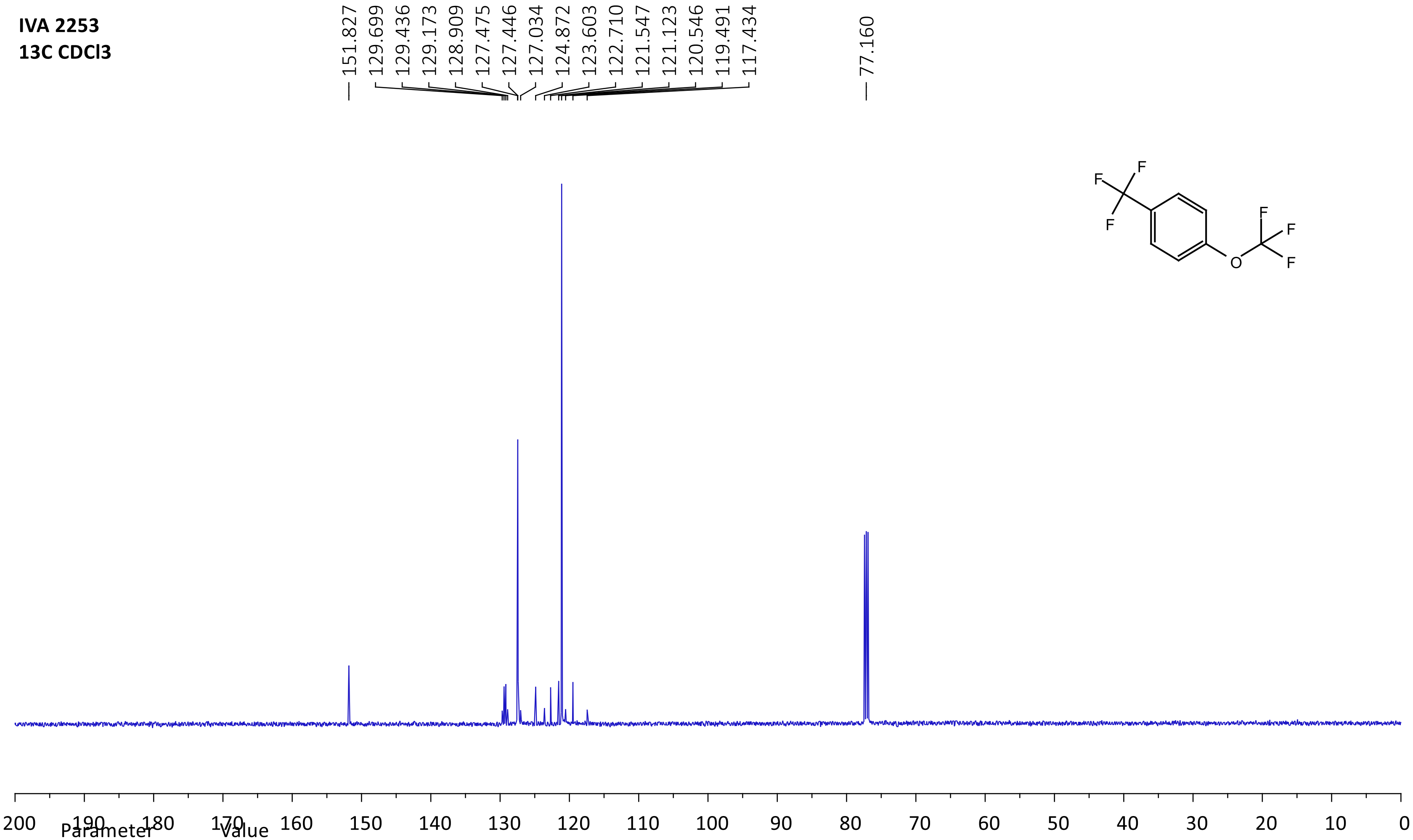

Parameter	Value
Number of Scans	512
Spectromet. Freq.	125.76
Spectral Width	36057.7
Spectral Size	65536
freq. of 0 ppm:	125.7577890

Compound **4e**

2489
1H CDCl3

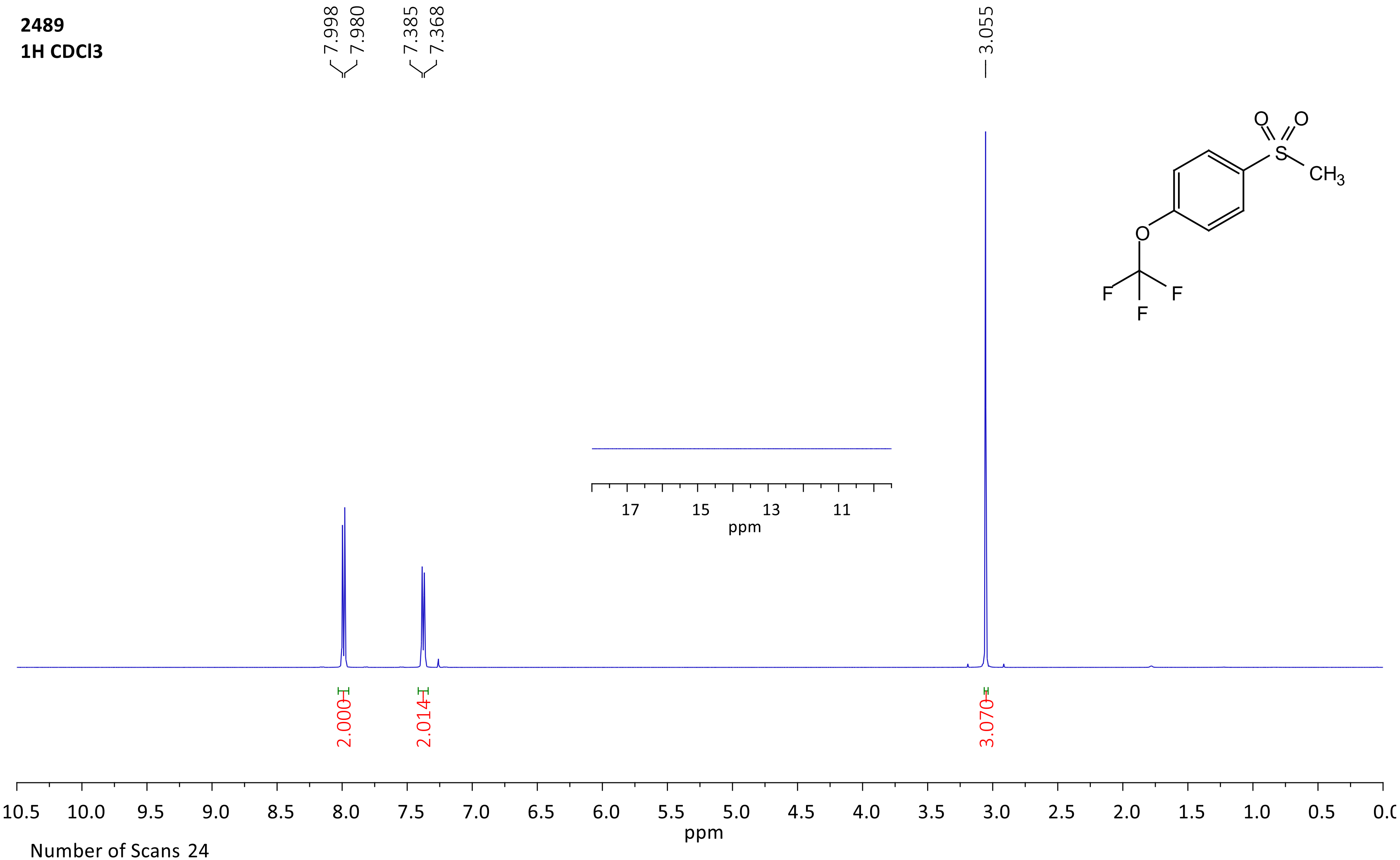

Number of Scans 24
Spectromet. Freq.500.13
Spectral Width 12335.5
Spectral Size 65536
freq. of 0 ppm: 500.1300236

Compound **4e**

2489
13C CDCl3

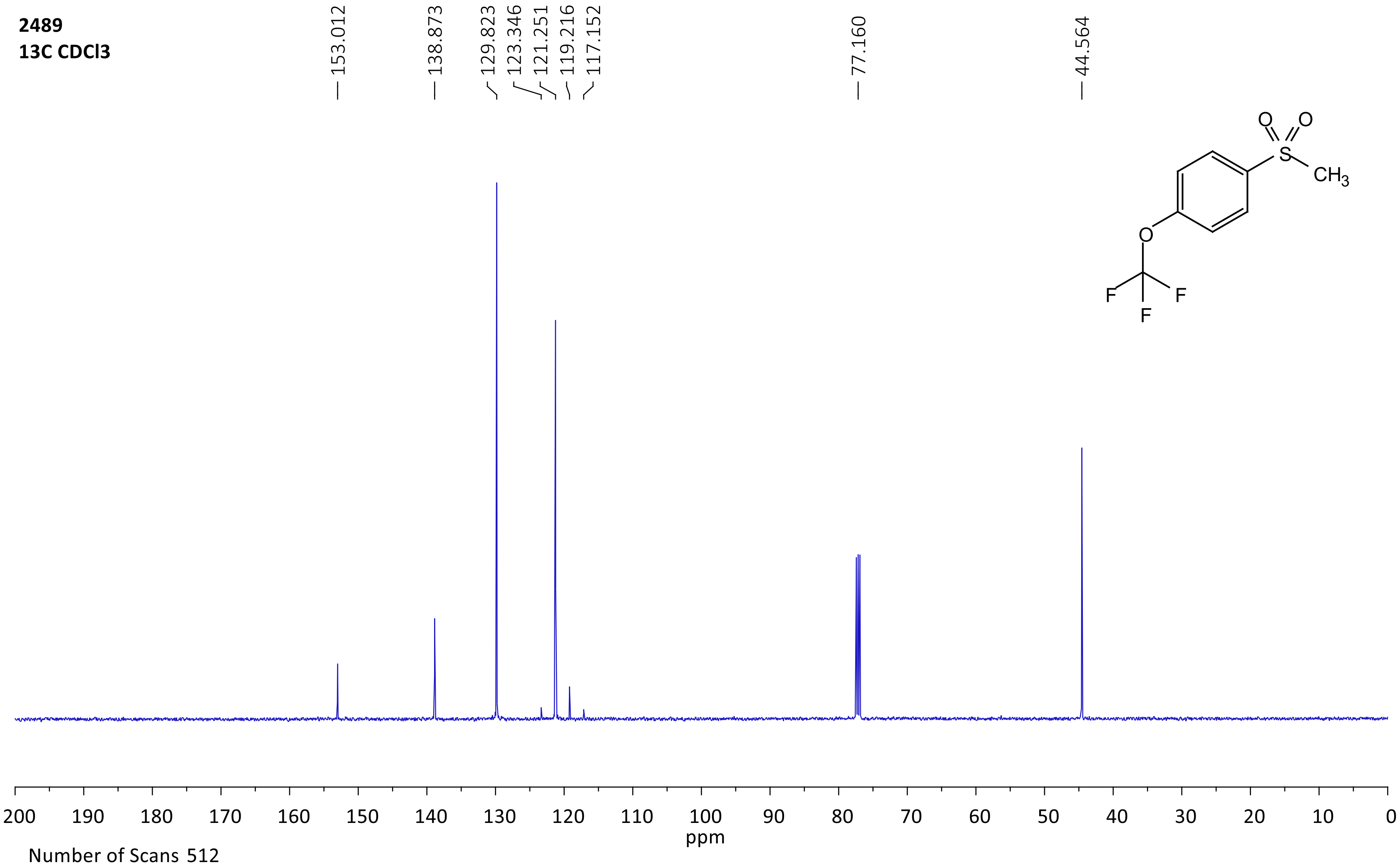

Number of Scans 512

Spectromet. Freq.125.76

Spectral Width 36057.7

Spectral Size 65536

freq. of 0 ppm: 125.7577890

Compound **4f**

2492
1H CDCl3

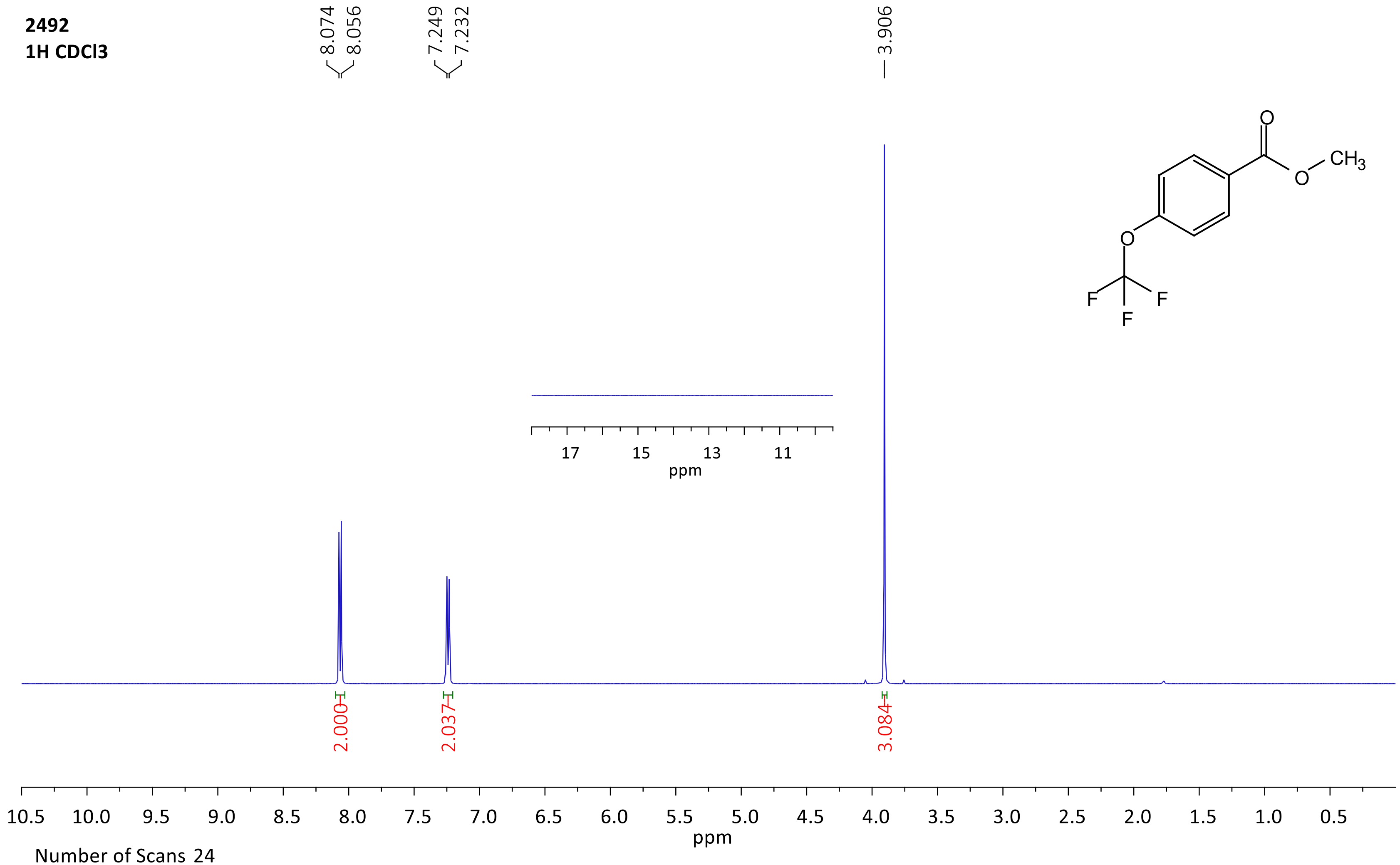

Number of Scans 24
Spectromet. Freq.500.13
Spectral Width 12335.5
Spectral Size 65536
freq. of 0 ppm: 500.1300236

Compound **4f**

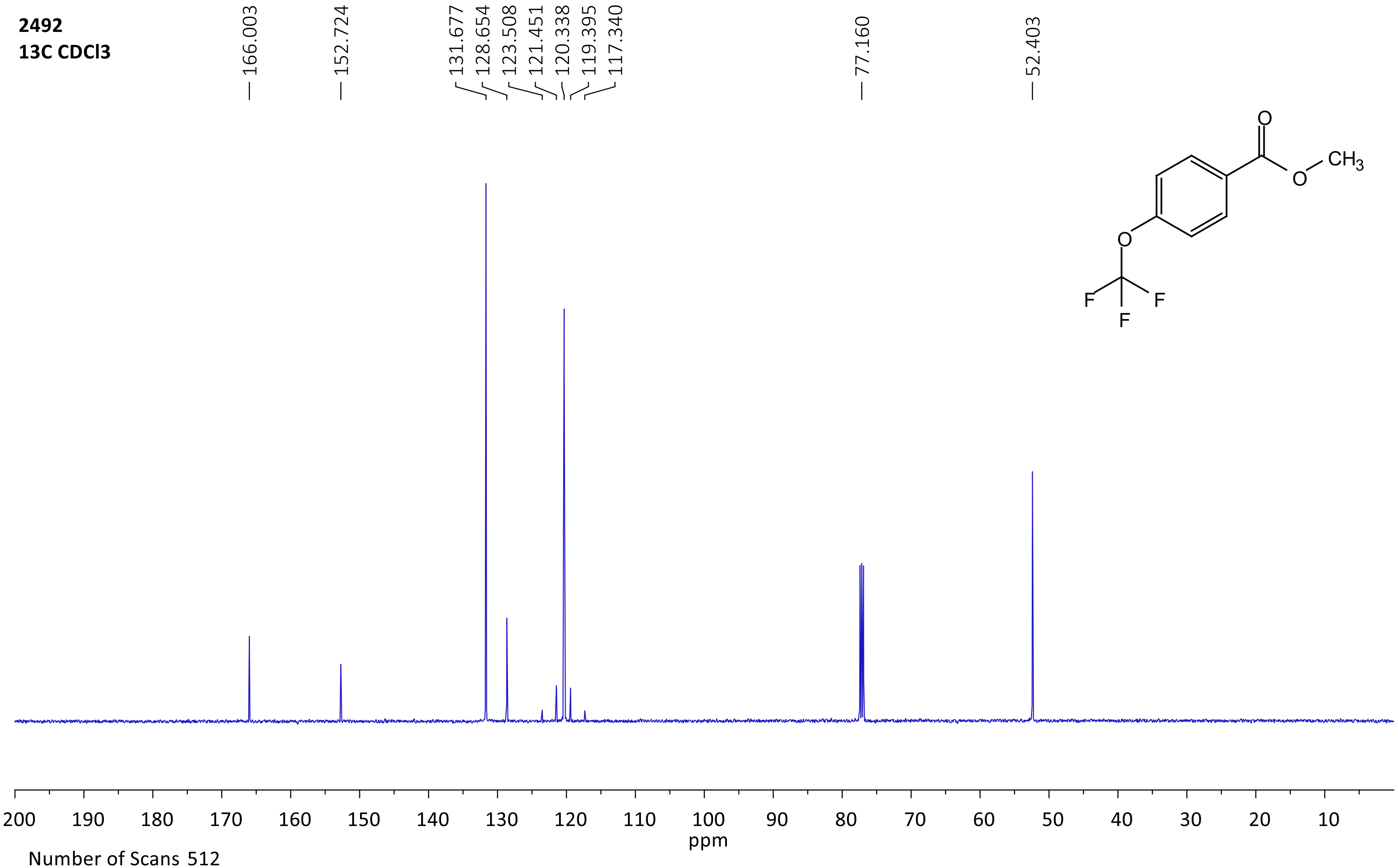

Number of Scans 512
Spectromet. Freq.125.76
Spectral Width 36057.7
Spectral Size 65536
freq. of 0 ppm: 125.7577890

Compound **4g**

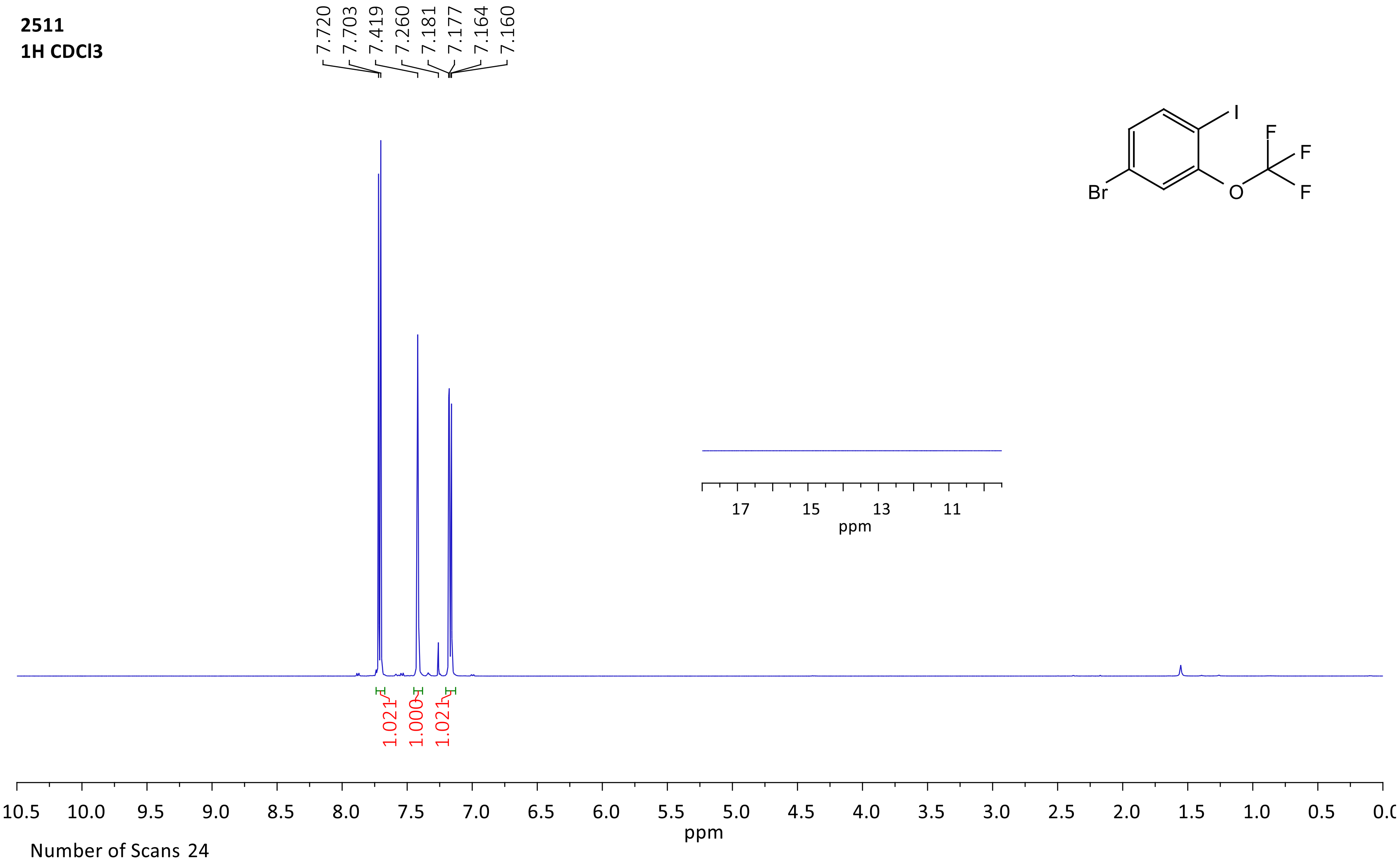

Number of Scans 24
Spectromet. Freq.500.13
Spectral Width 12335.5
Spectral Size 65536
freq. of 0 ppm: 500.1300236

Compound **4g**

2511
13C CDCl3

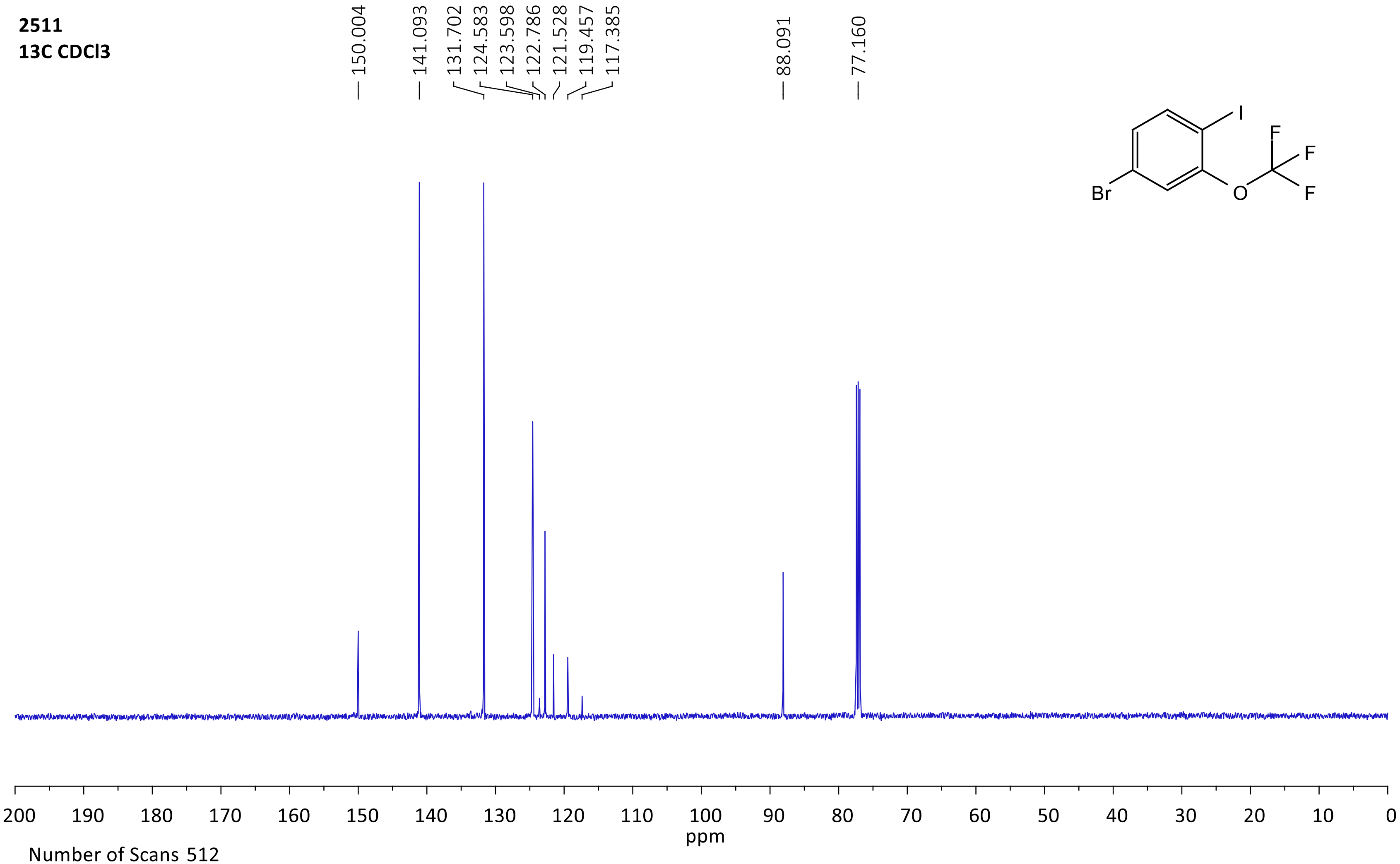

Number of Scans 512
Spectromet. Freq.125.76
Spectral Width 36057.7
Spectral Size 65536
freq. of 0 ppm: 125.7577890

Compound **4h**

2571
1H CDCl3

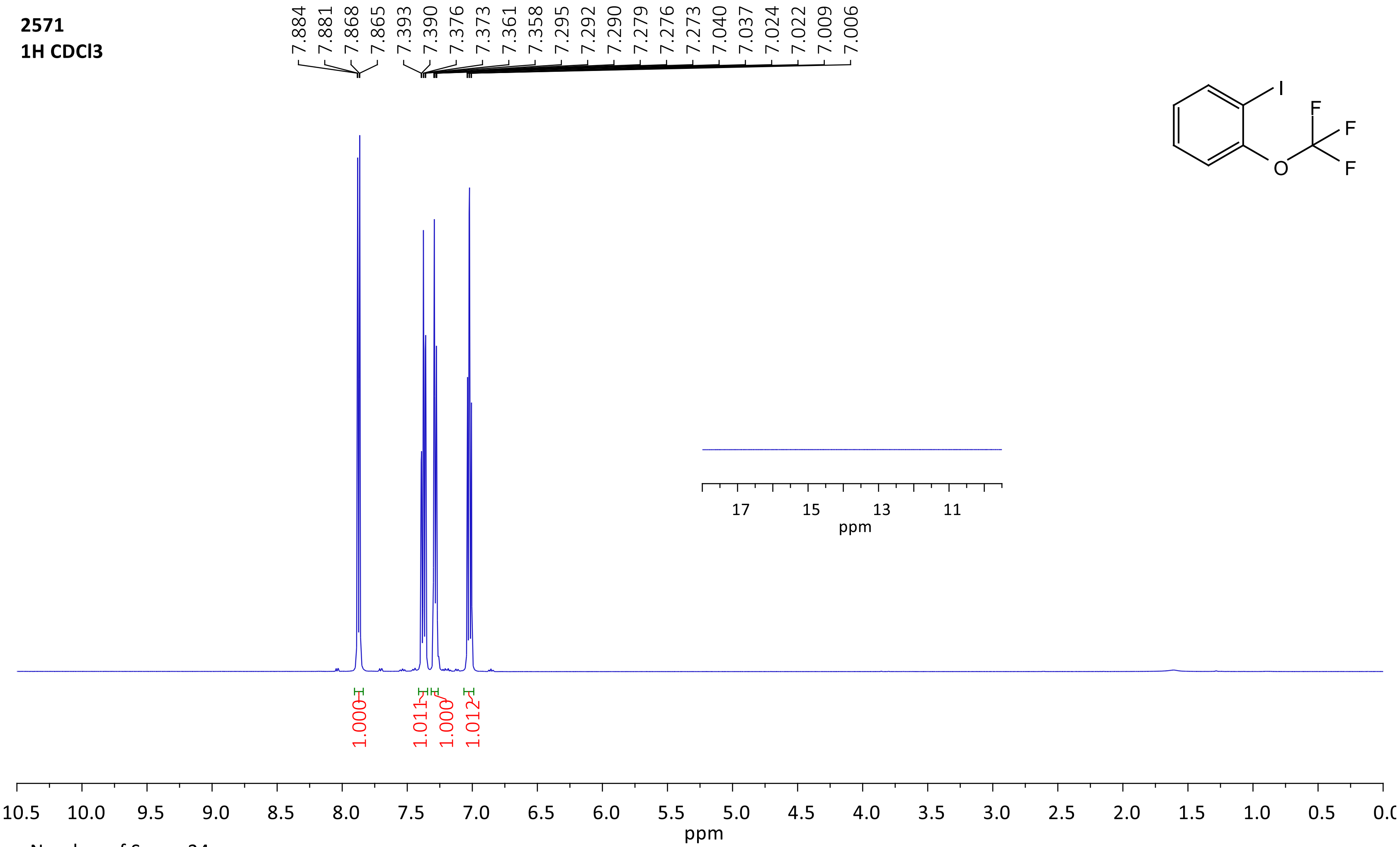

Number of Scans 24
Spectromet. Freq.500.13
Spectral Width 12335.5
Spectral Size 65536
freq. of 0 ppm: 500.1300236

Compound **4h**

2571
13C CDCl3

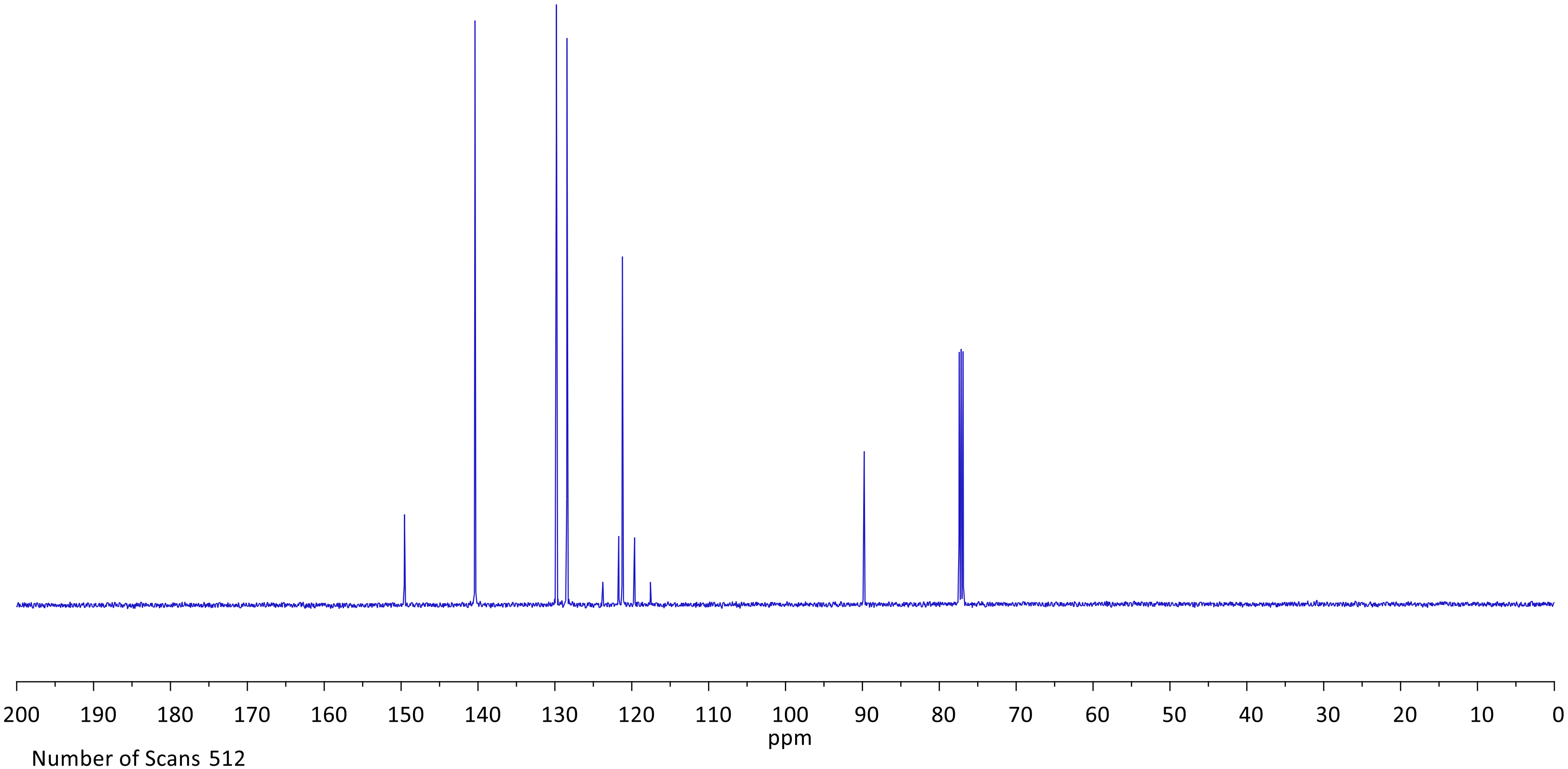

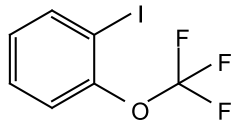

Number of Scans 512
Spectromet. Freq.125.76
Spectral Width 36057.7
Spectral Size 65536
freq. of 0 ppm: 125.7577890

Compound **4i**

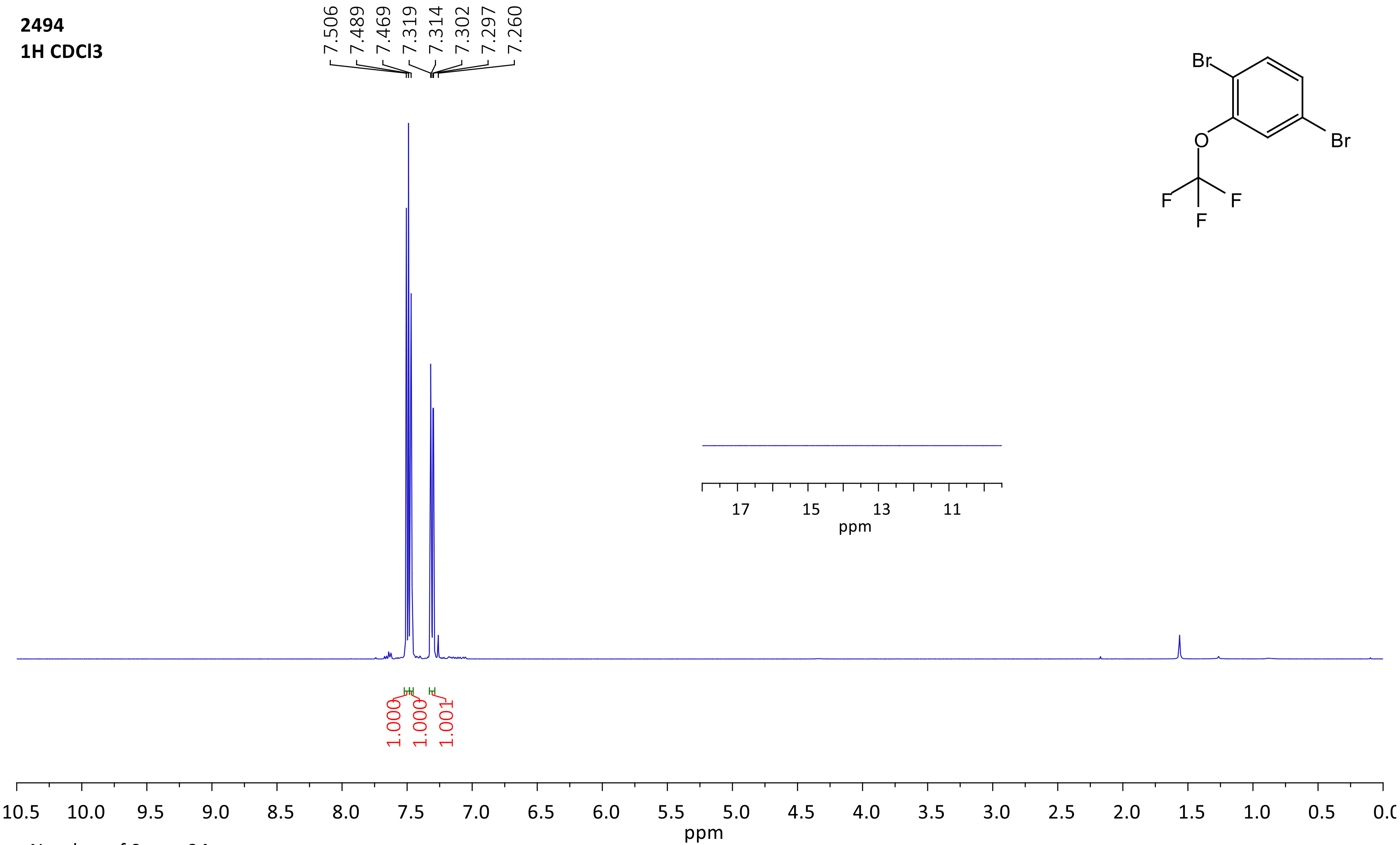

Number of Scans 24

Spectromet. Freq.500.13

Spectral Width 12335.5

Spectral Size 65536

freq. of 0 ppm: 500.1300236

Compound **4i**

2494
13C CDCl3

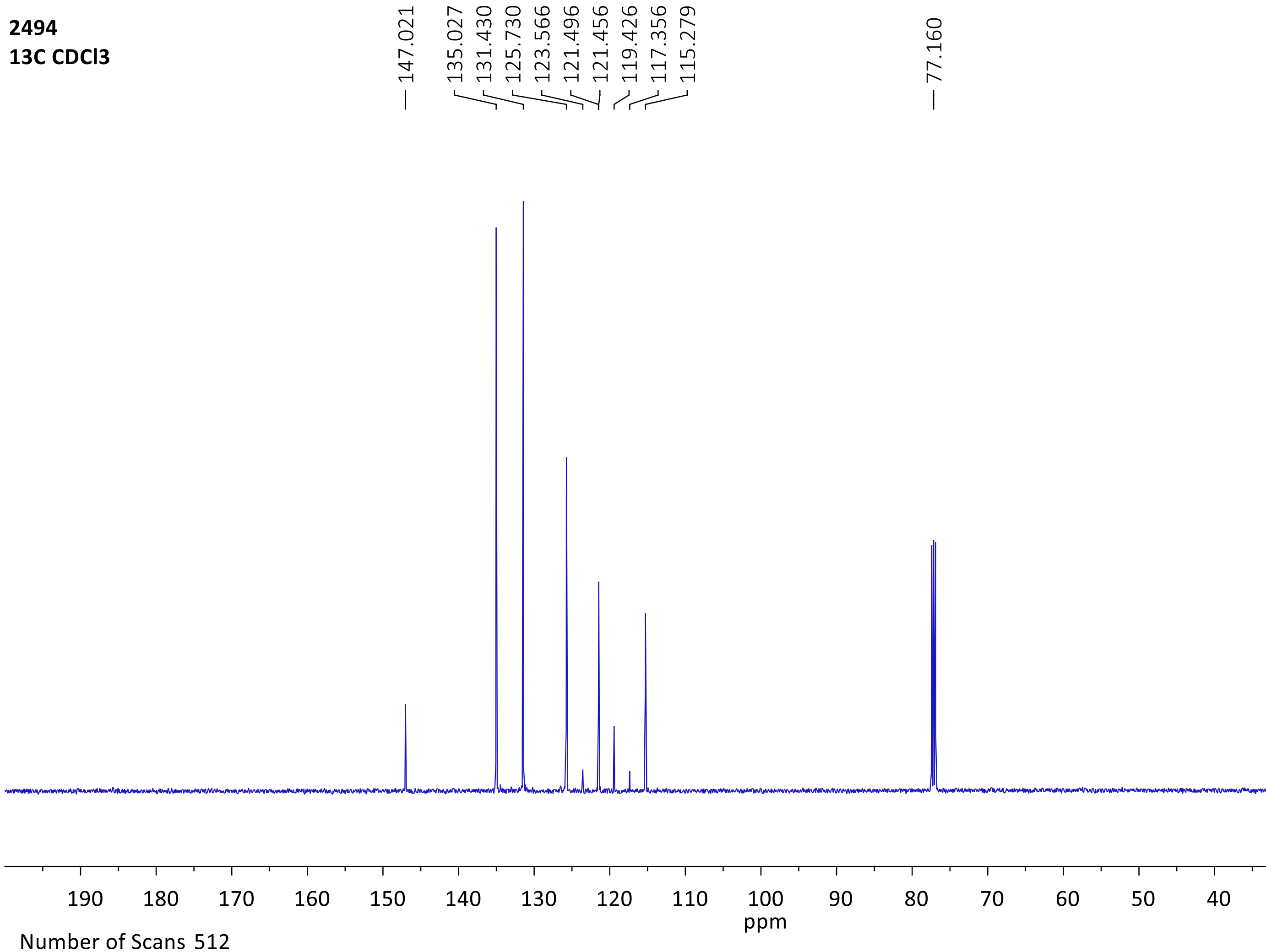

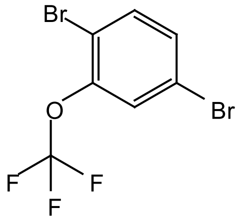

Number of Scans 512
Spectromet. Freq.125.76
Spectral Width 36057.7
Spectral Size 65536
freq. of 0 ppm: 125.7577890

Compound **4j**

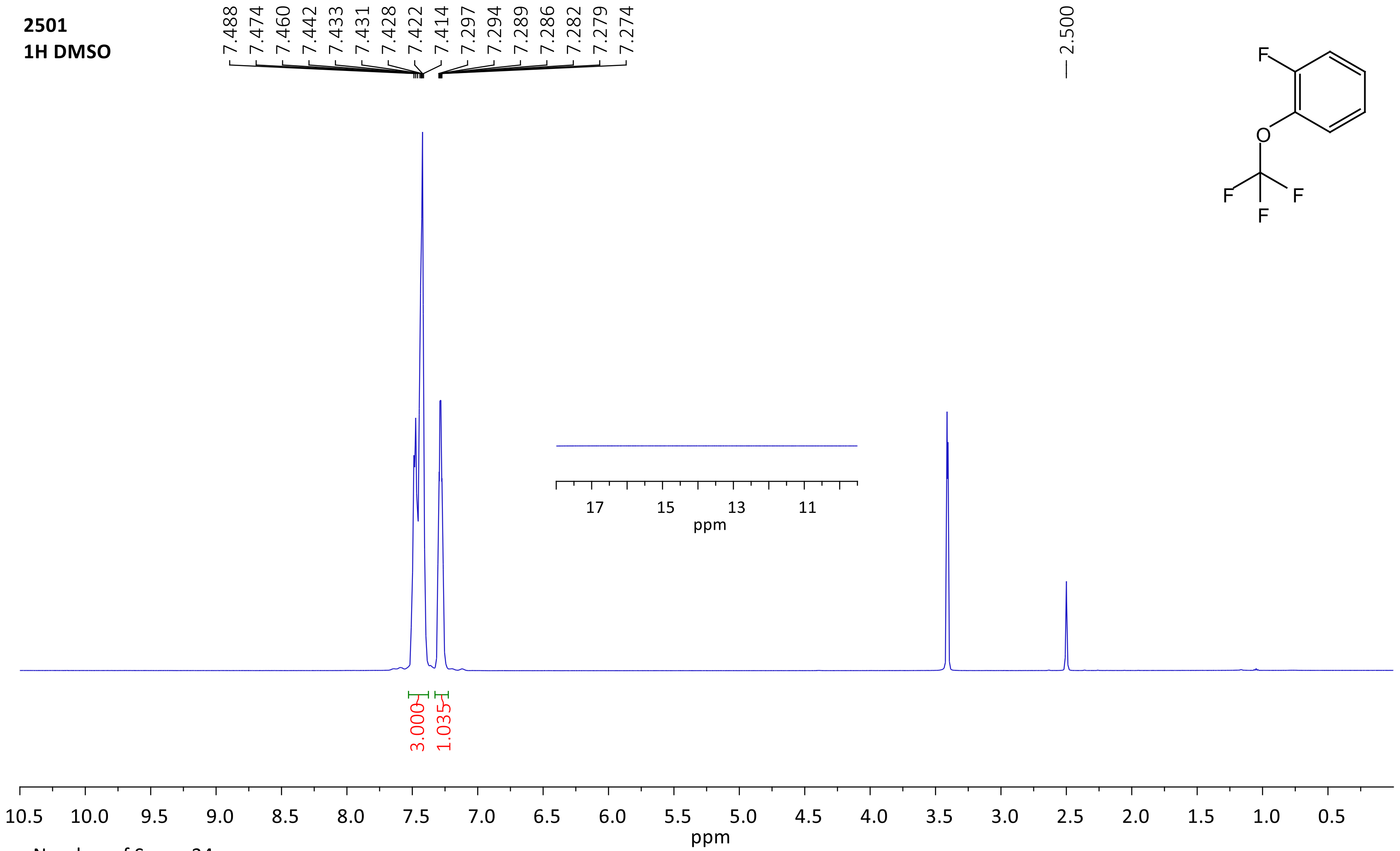

Number of Scans 24
Spectromet. Freq.500.13
Spectral Width 12335.5
Spectral Size 65536
freq. of 0 ppm: 500.1300236

Compound **4j**

2501
13C DMSO

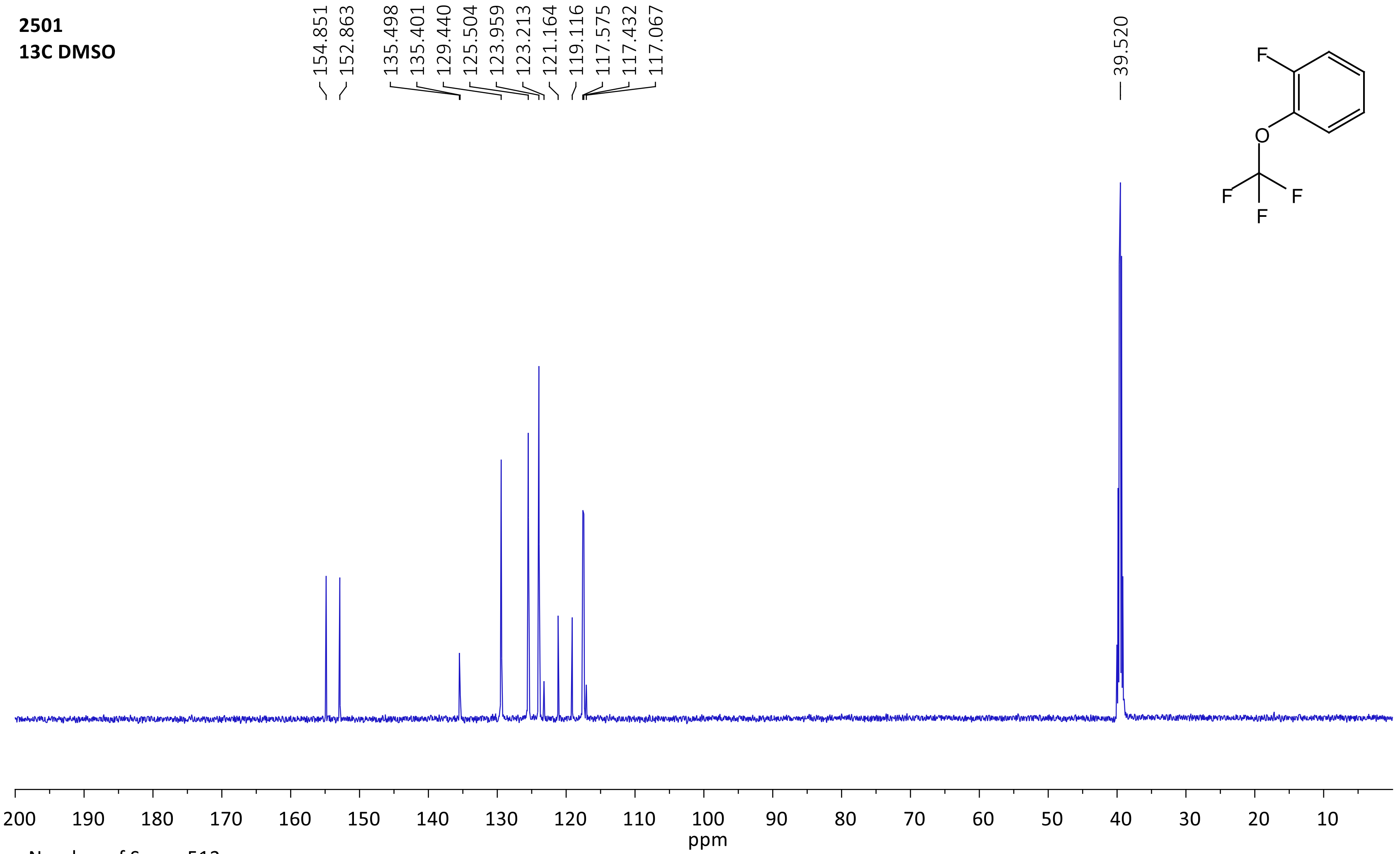

Number of Scans 512
Spectromet. Freq.125.76
Spectral Width 36057.7
Spectral Size 65536
freq. of 0 ppm: 125.7577890

Compound **4k**

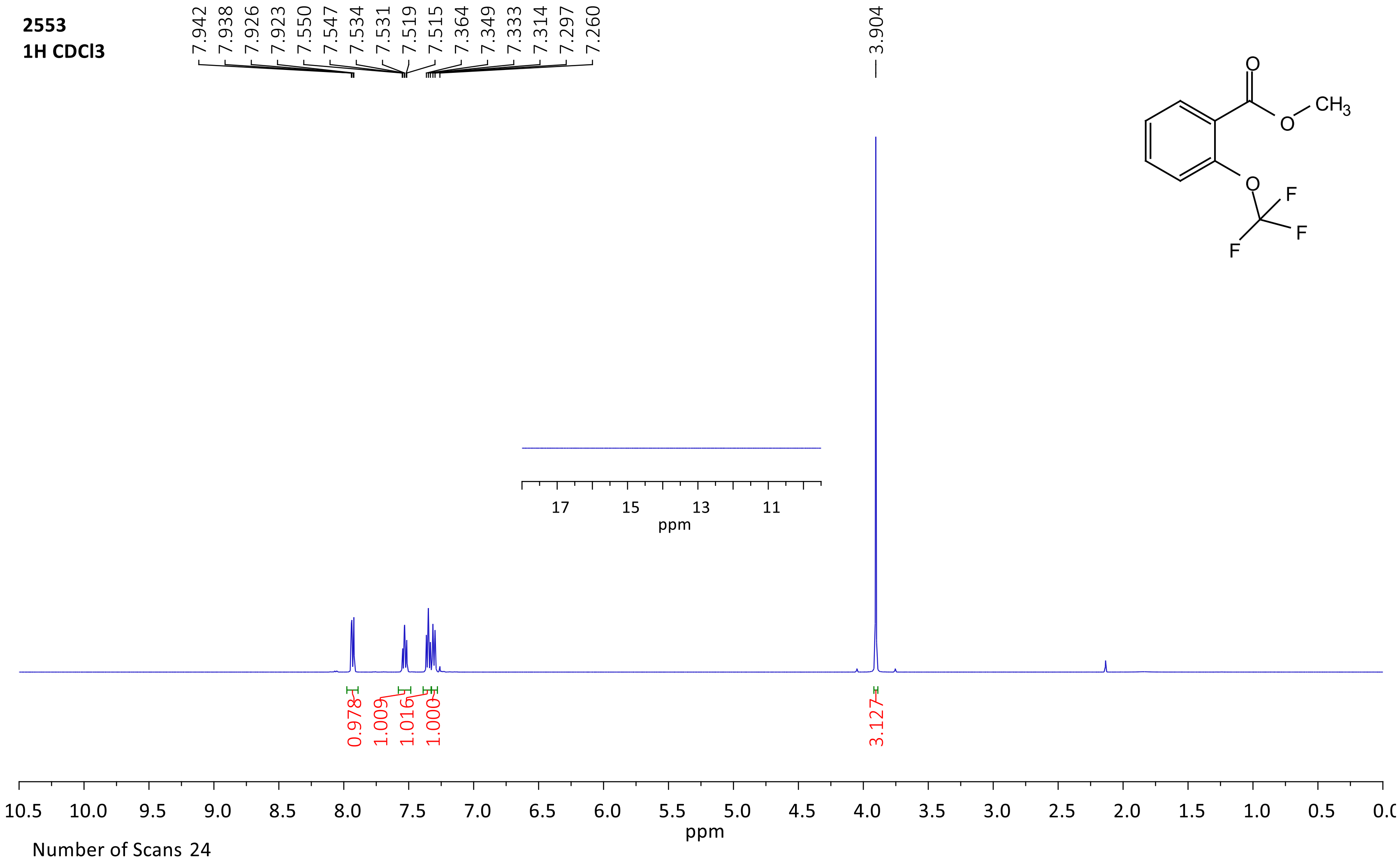

Number of Scans 24
Spectromet. Freq.500.13
Spectral Width 12335.5
Spectral Size 65536
freq. of 0 ppm: 500.1300236

Compound **4k**

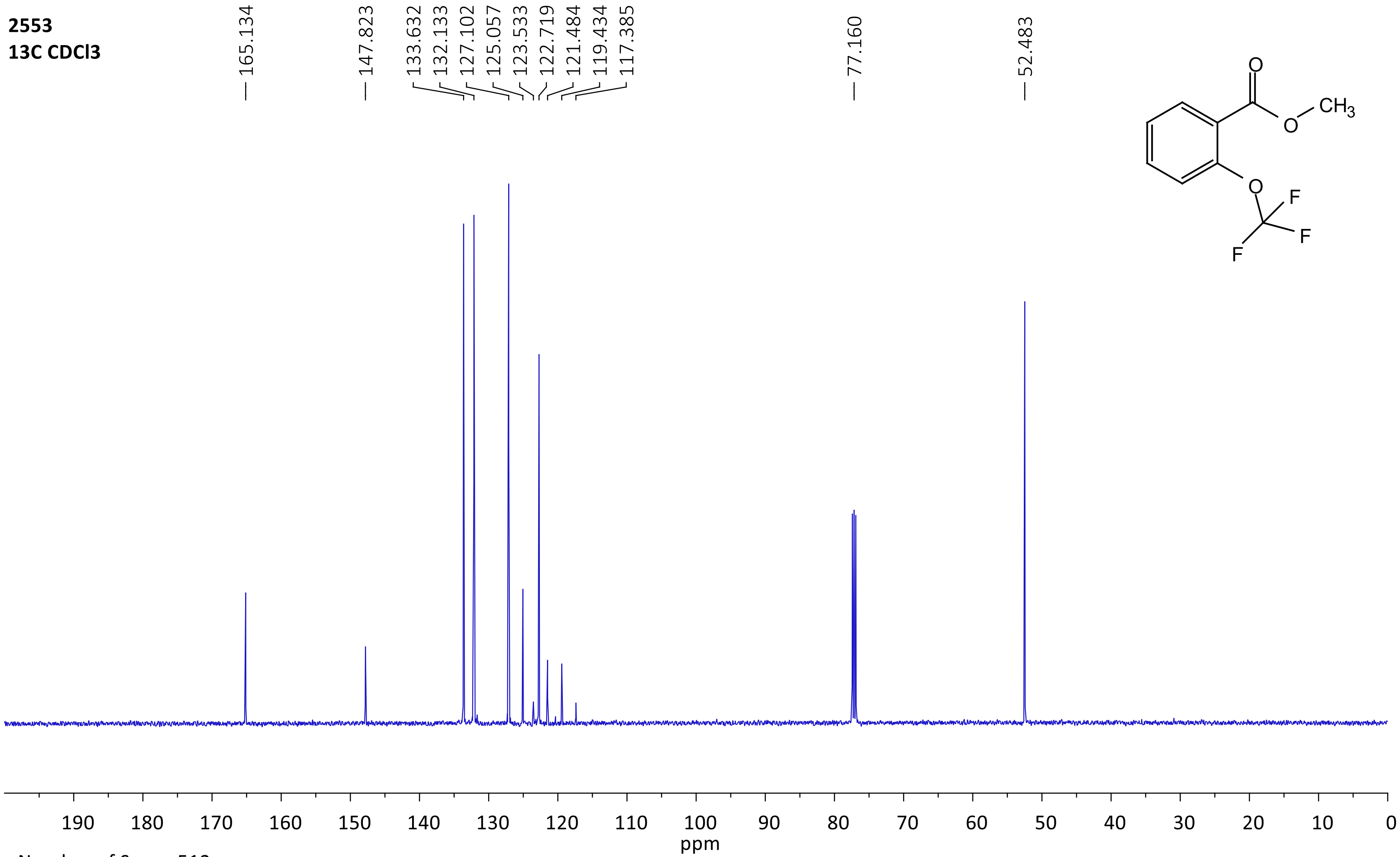

Number of Scans 512
Spectromet. Freq.125.76
Spectral Width 36057.7
Spectral Size 65536
freq. of 0 ppm: 125.7577890

Compound **4l**

2490
1H CDCl3

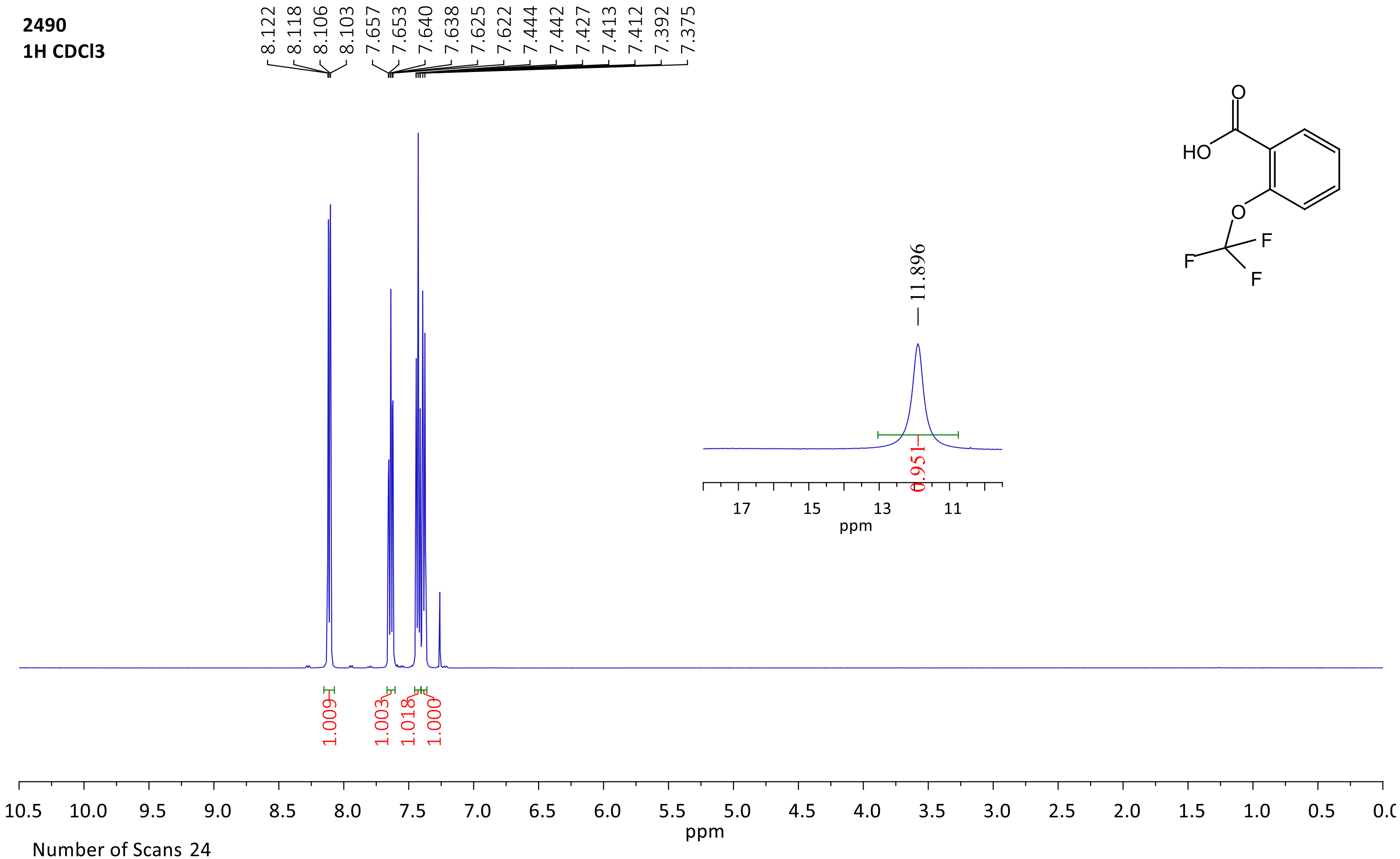

Number of Scans 24
Spectromet. Freq.500.13
Spectral Width 12335.5
Spectral Size 65536
freq. of 0 ppm: 500.1300236

Compound **4l**

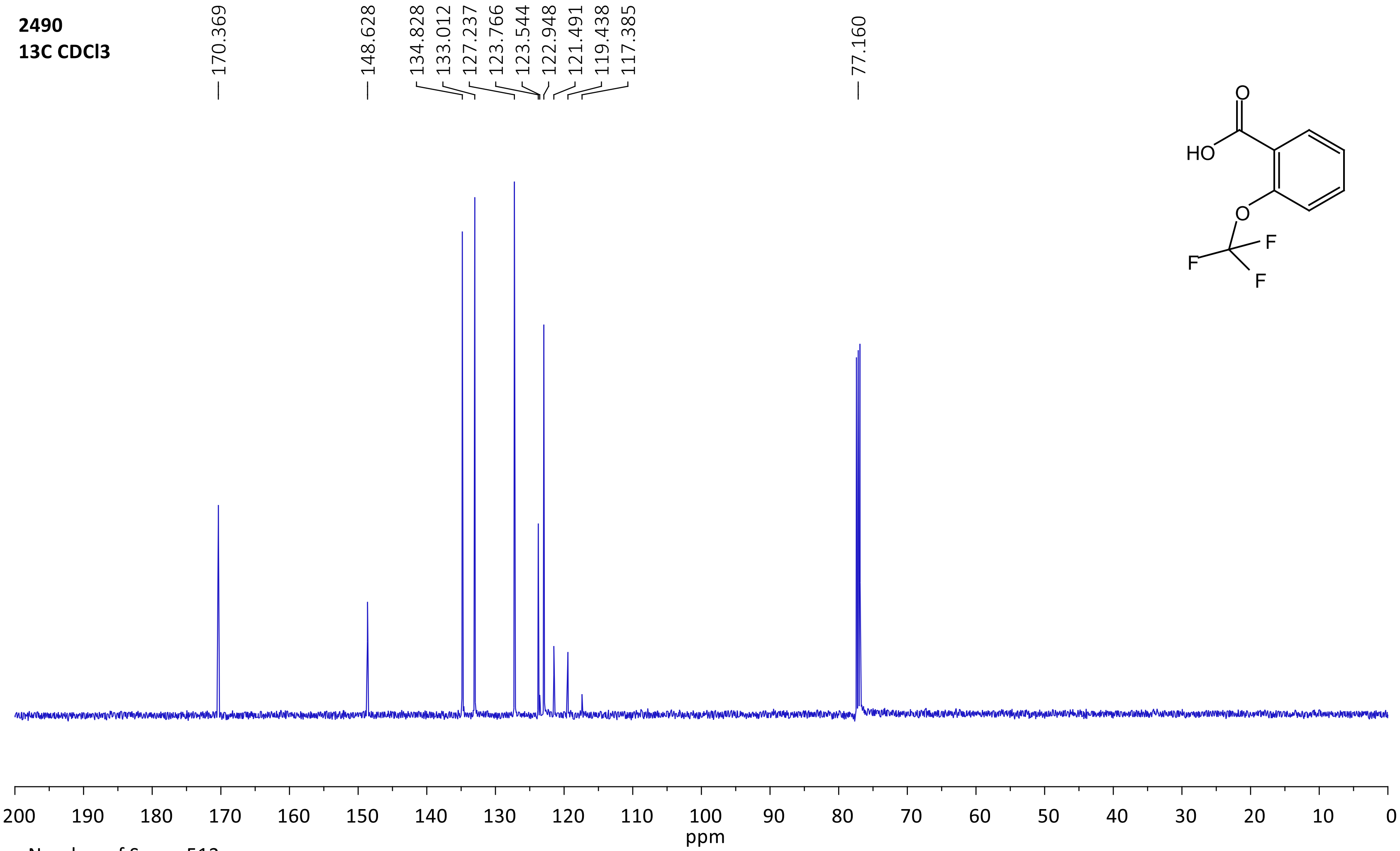

Number of Scans 512
Spectromet. Freq.125.76
Spectral Width 36057.7
Spectral Size 65536
freq. of 0 ppm: 125.7577890

Compound **4m**

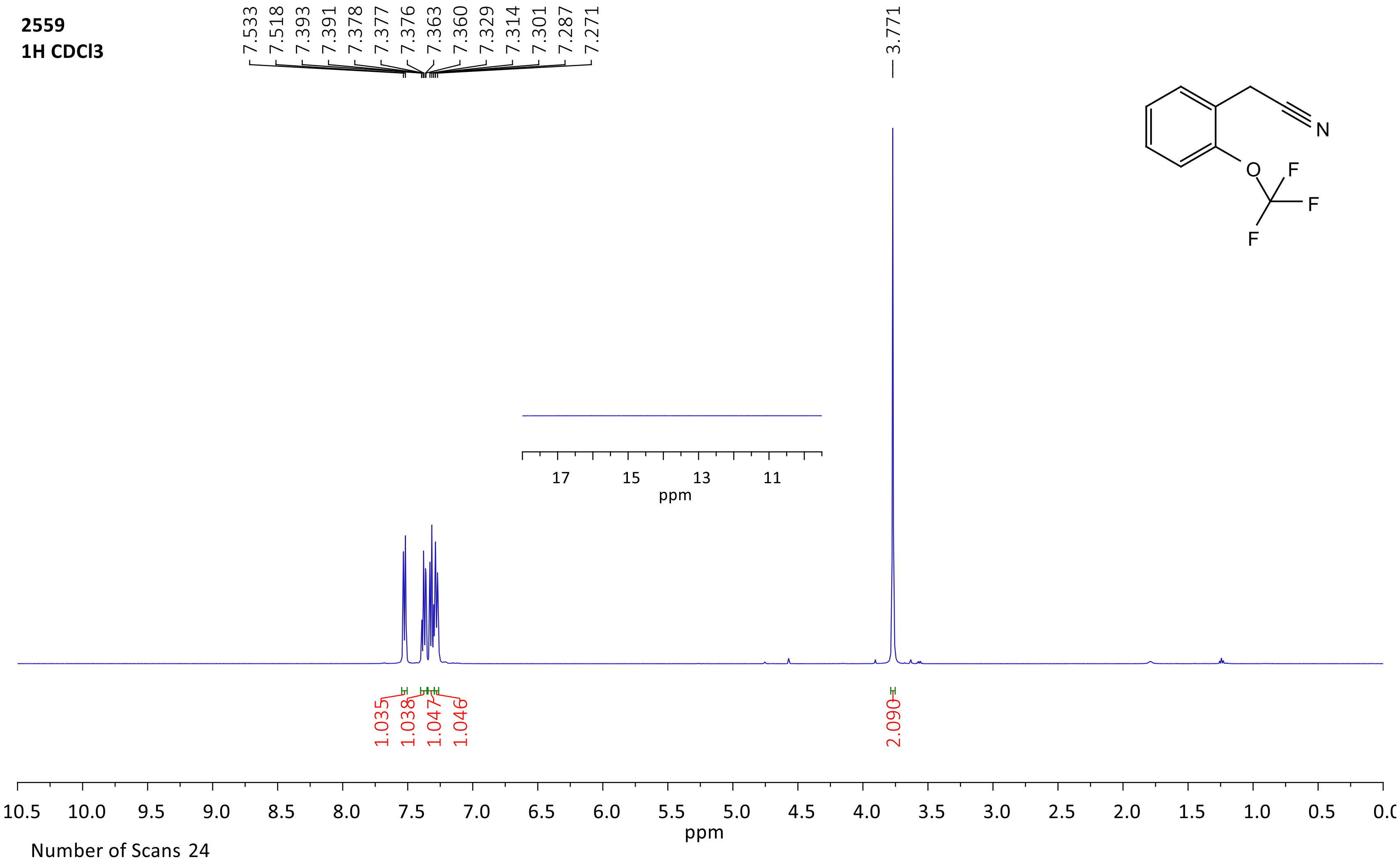

Number of Scans 24

Spectromet. Freq.500.13

Spectral Width 12335.5

Spectral Size 65536

freq. of 0 ppm: 500.1300236

Compound **4m**

2559
13C CDCl3

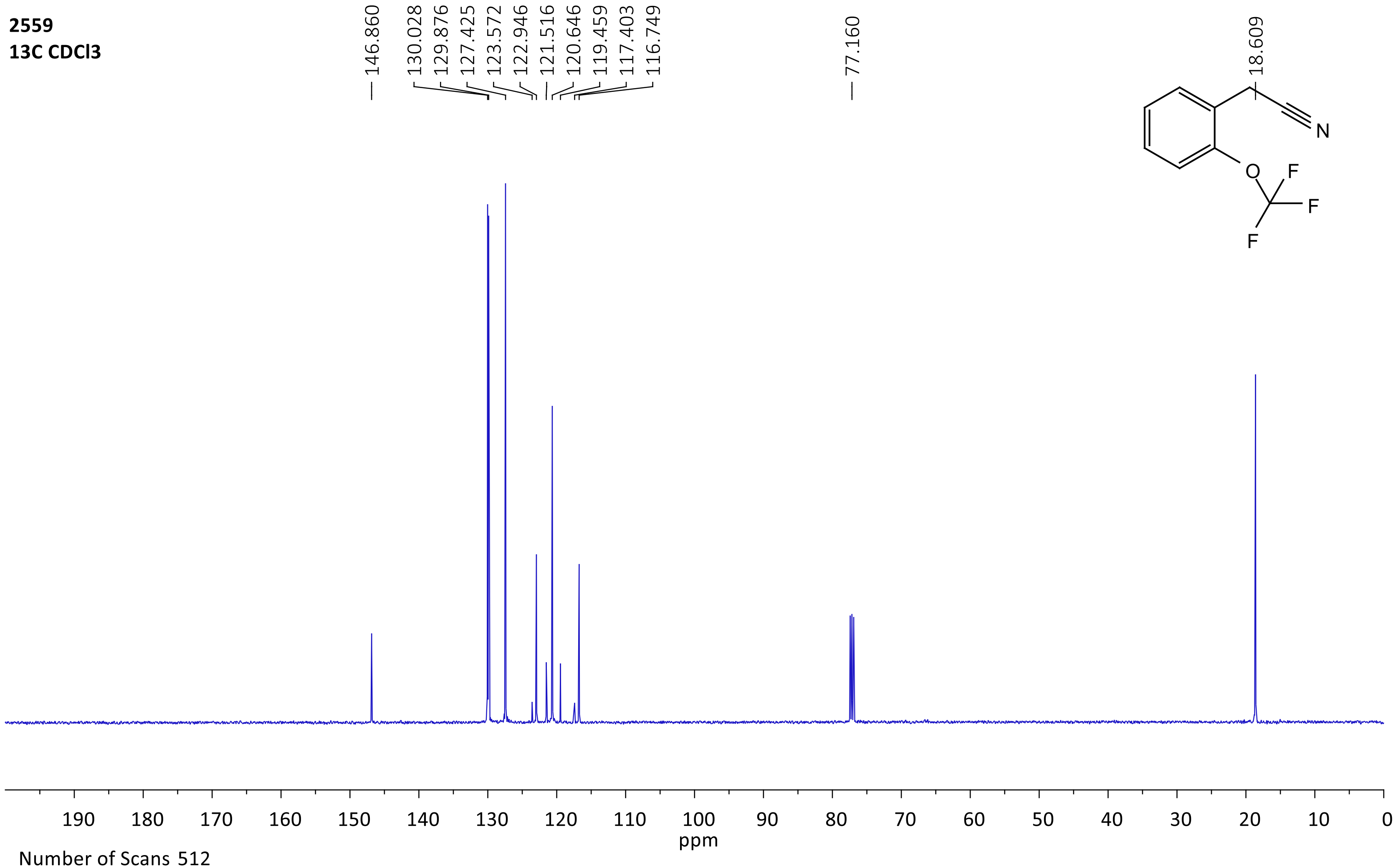

Number of Scans 512
Spectromet. Freq.125.76
Spectral Width 36057.7
Spectral Size 65536
freq. of 0 ppm: 125.7577890

Compound **4n**

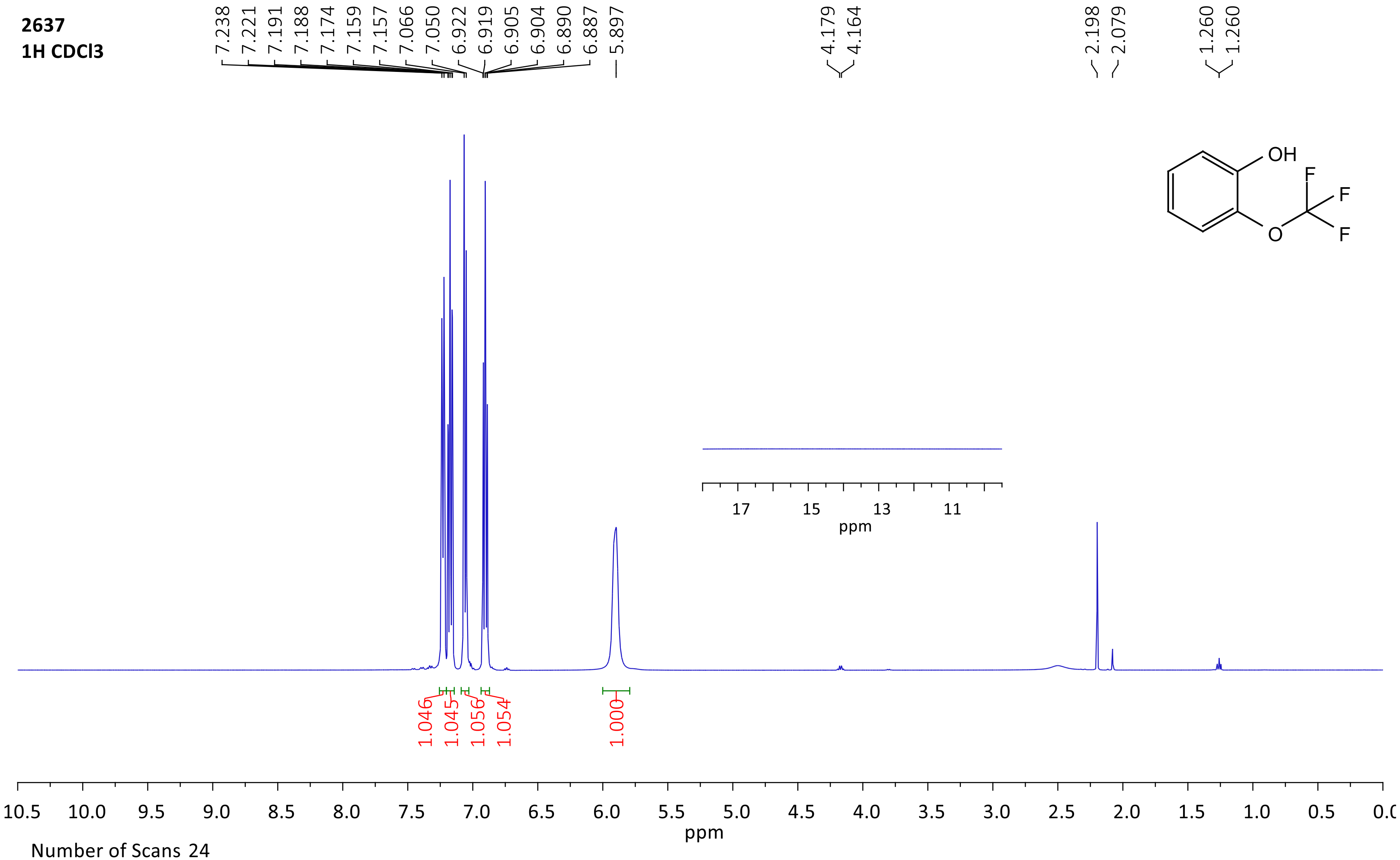

Number of Scans 24

Spectromet. Freq.500.13

Spectral Width 12335.5

Spectral Size 65536

freq. of 0 ppm: 500.1300236

Compound **4n**

2637
13C CDCl3

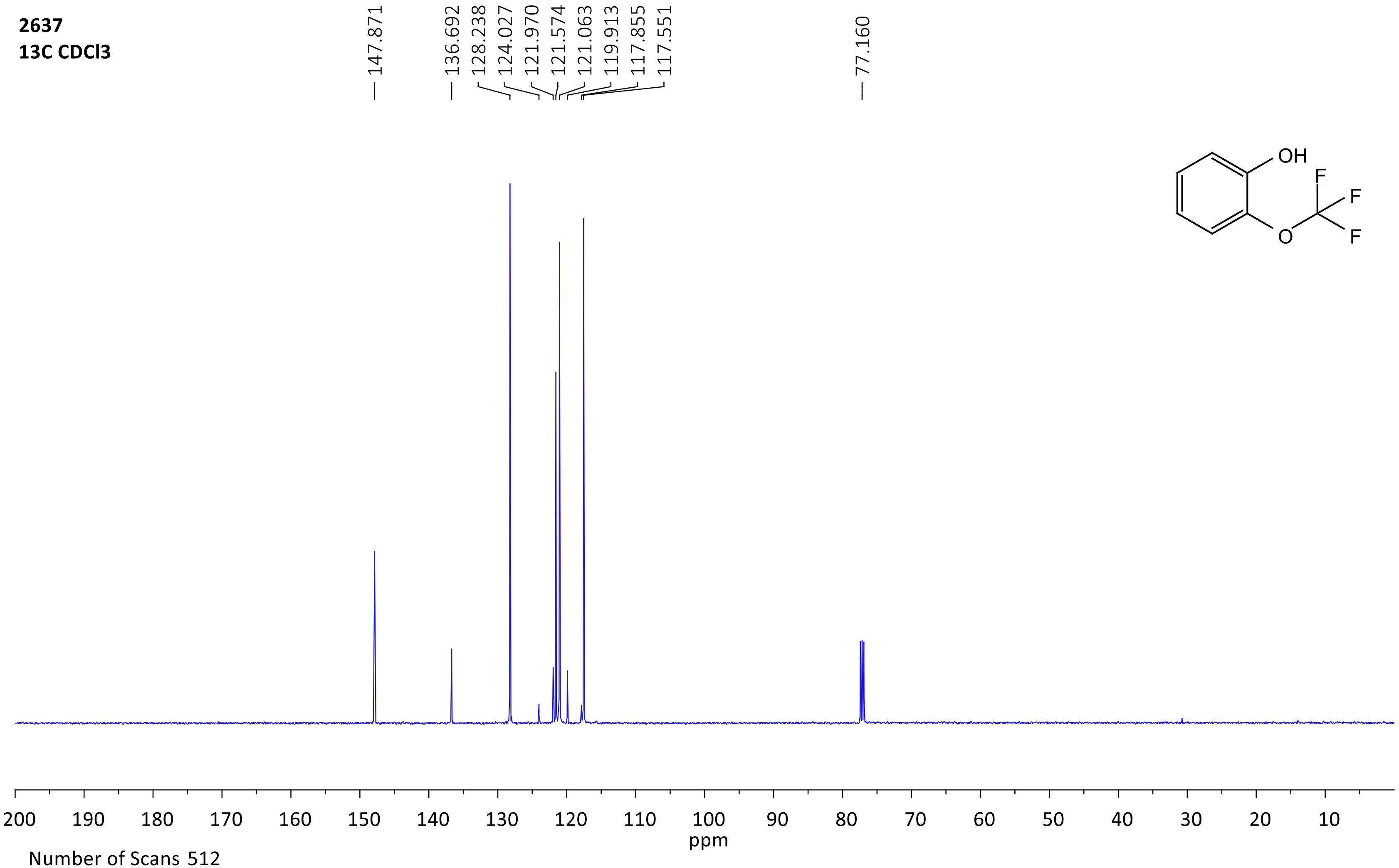

Number of Scans 512
Spectromet. Freq.125.76
Spectral Width 36057.7
Spectral Size 65536
freq. of 0 ppm: 125.7577890

Compound **4o**

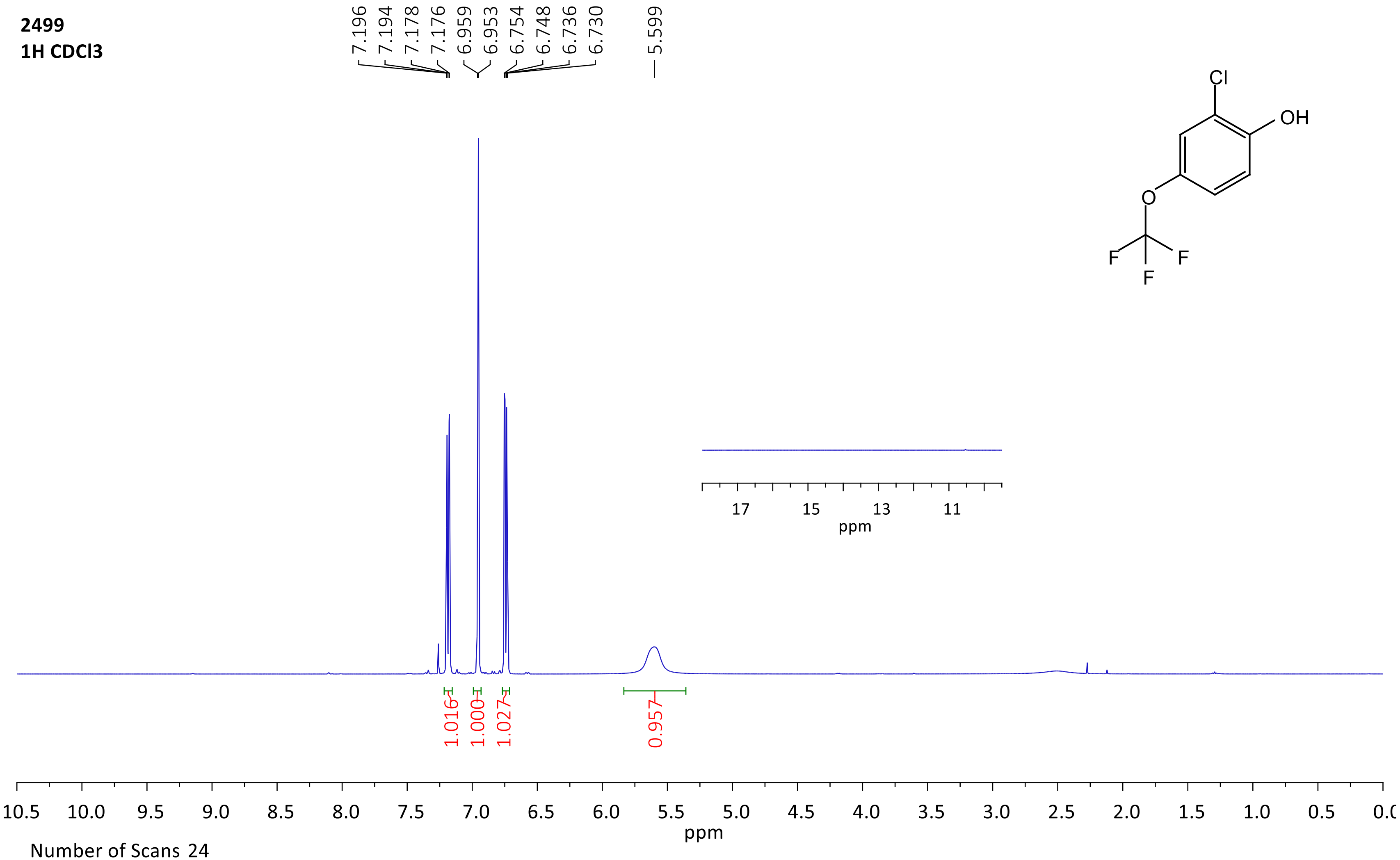

Number of Scans 24
Spectromet. Freq.500.13
Spectral Width 12335.5
Spectral Size 65536
freq. of 0 ppm: 500.1300236

Compound **4o**

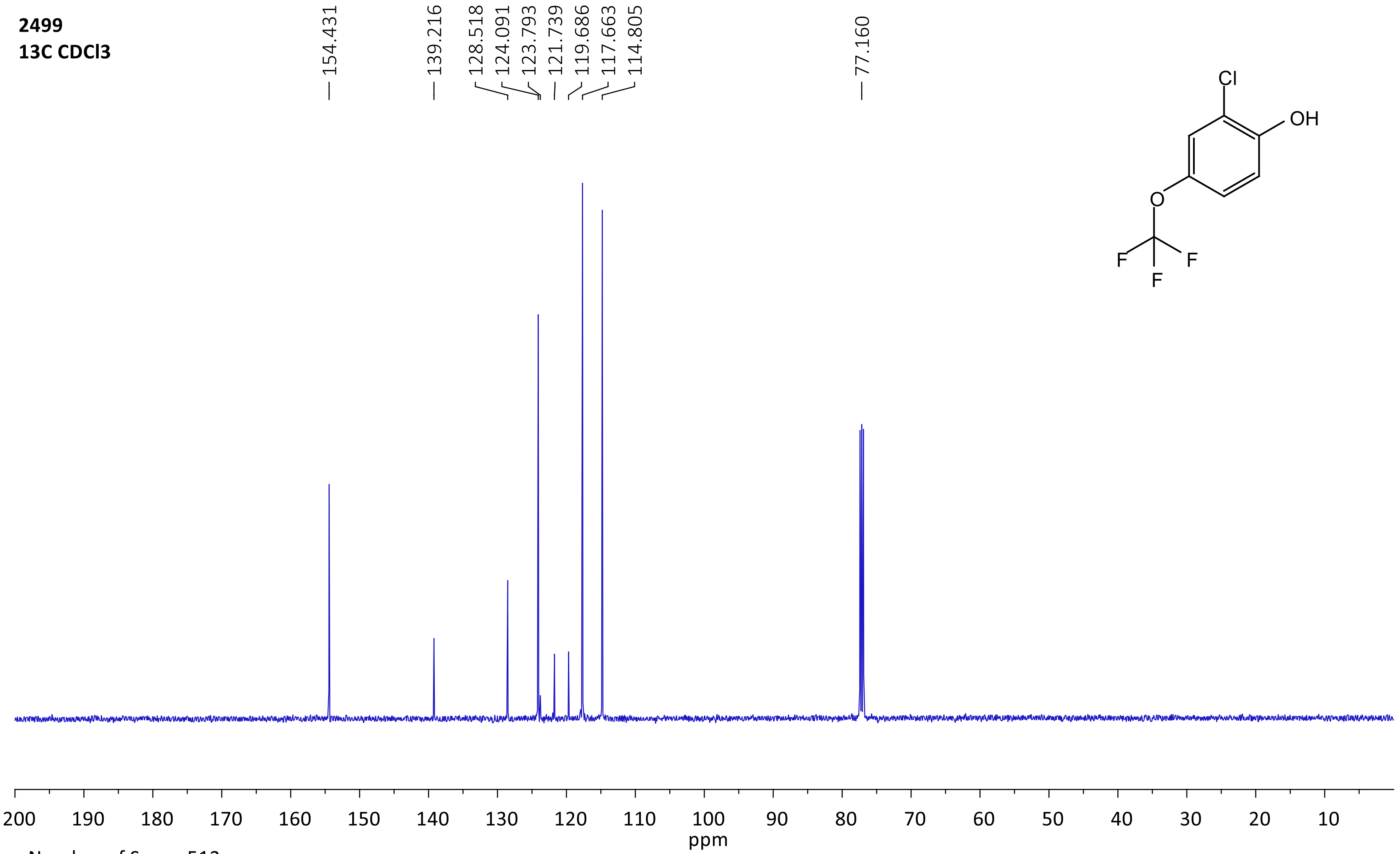

Number of Scans 512

Spectromet. Freq.125.76

Spectral Width 36057.7

Spectral Size 65536

freq. of 0 ppm: 125.7577890

Compound **4p**

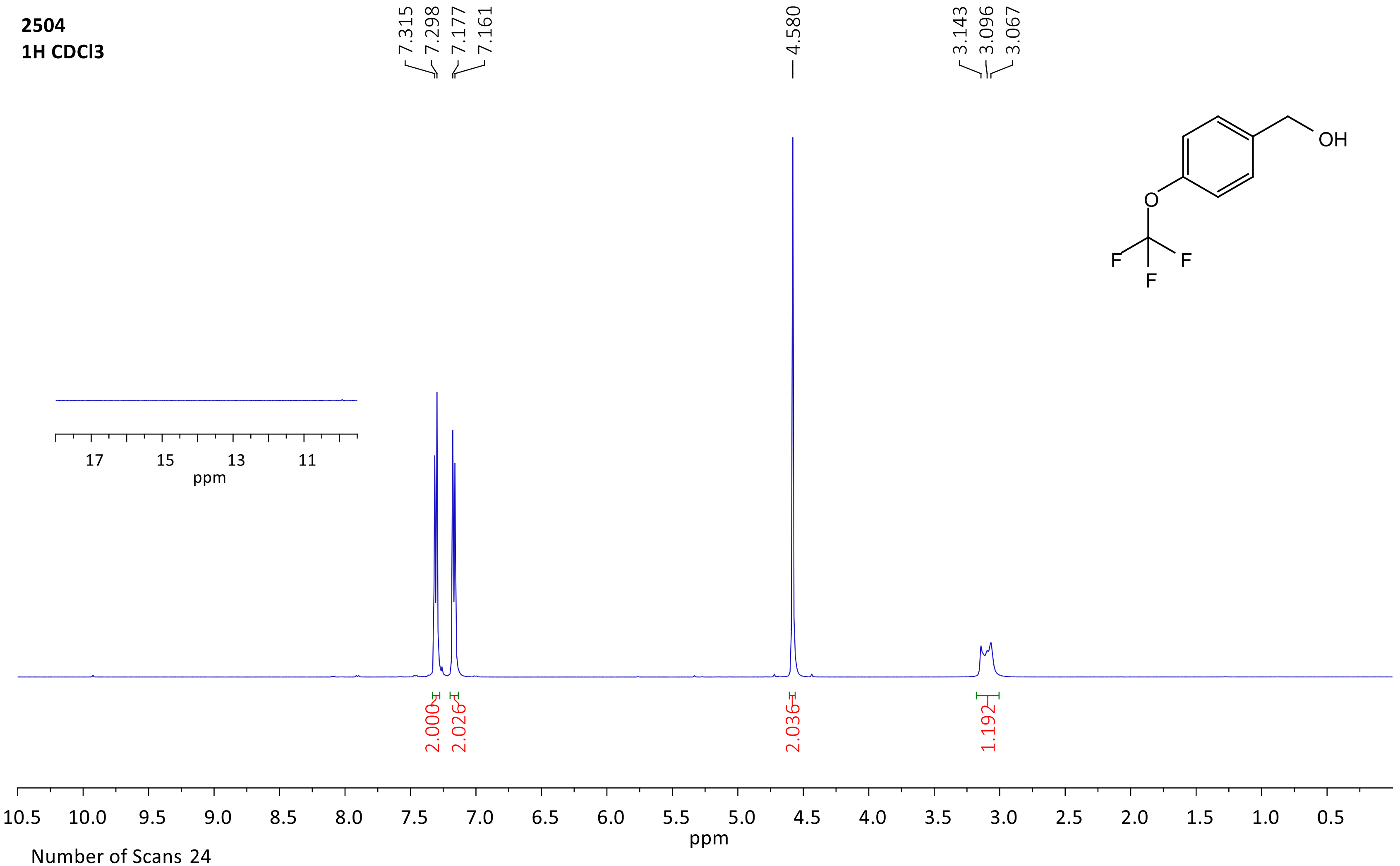

Number of Scans 24
Spectromet. Freq.500.13
Spectral Width 12335.5
Spectral Size 65536
freq. of 0 ppm: 500.1300236

Compound **4p**

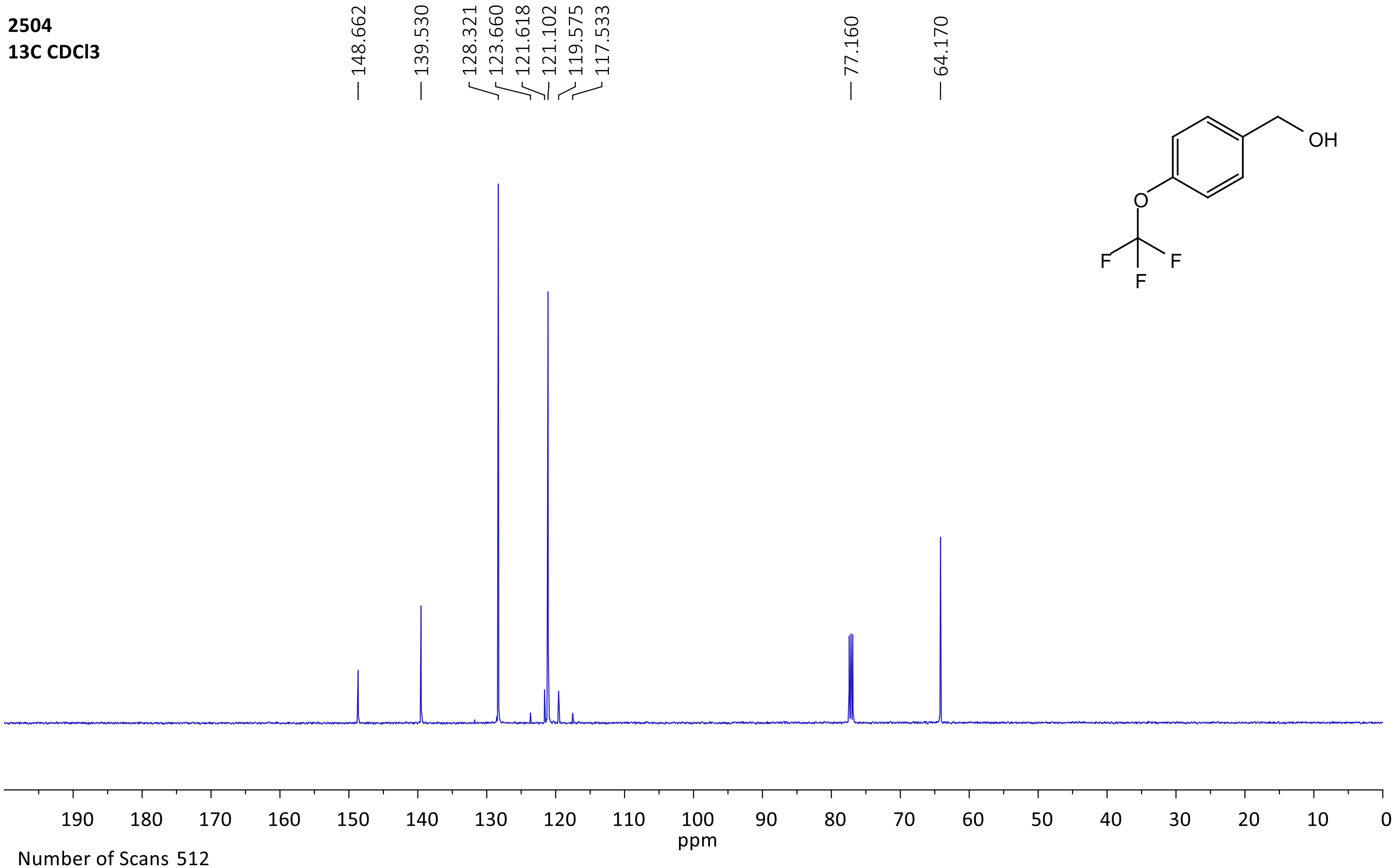

Number of Scans 512

Spectromet. Freq.125.76

Spectral Width 36057.7

Spectral Size 65536

freq. of 0 ppm: 125.7577890

Compound **4q**

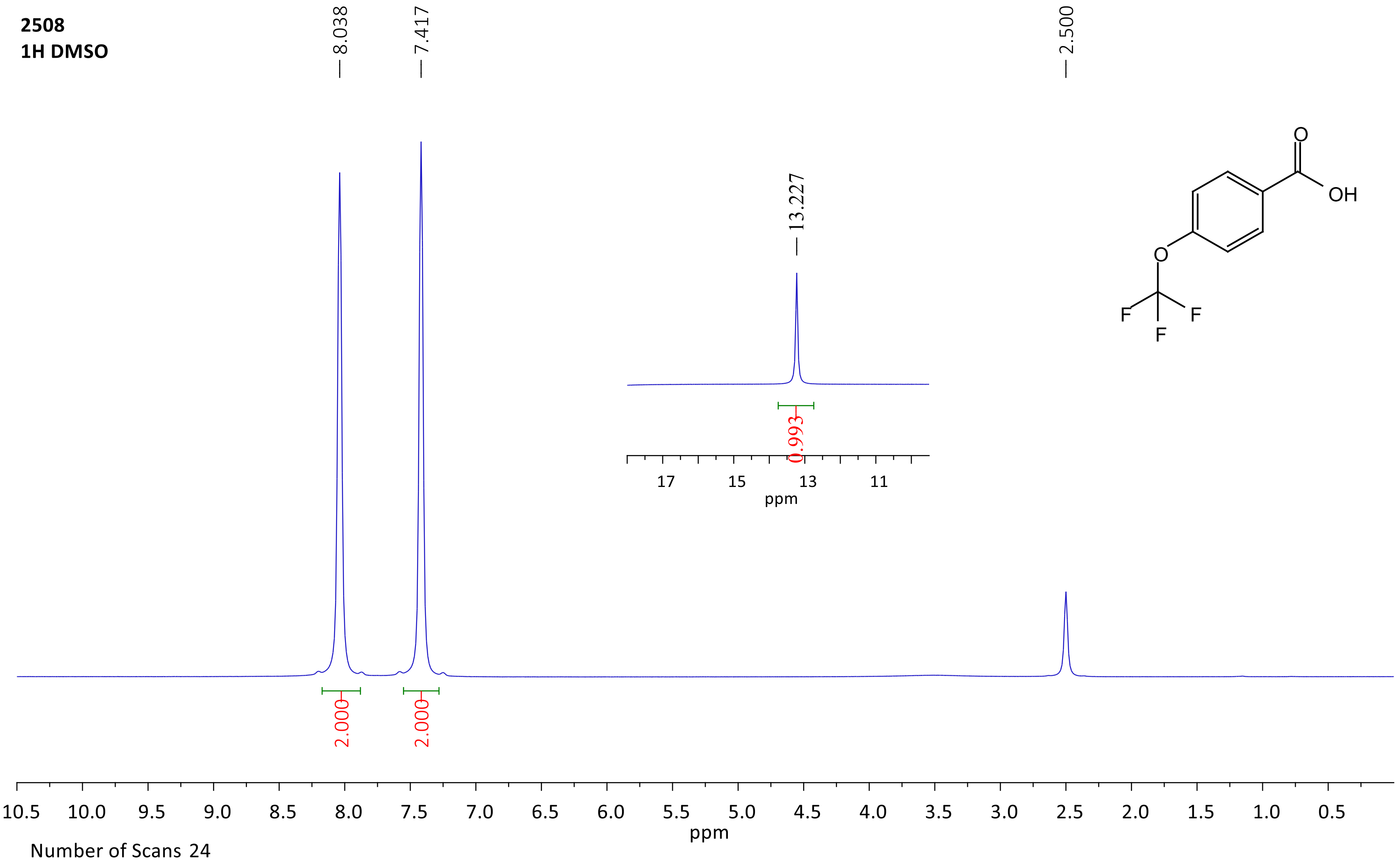

Number of Scans 24
Spectromet. Freq.500.13
Spectral Width 12335.5
Spectral Size 65536
freq. of 0 ppm: 500.1300236

Compound **4q**

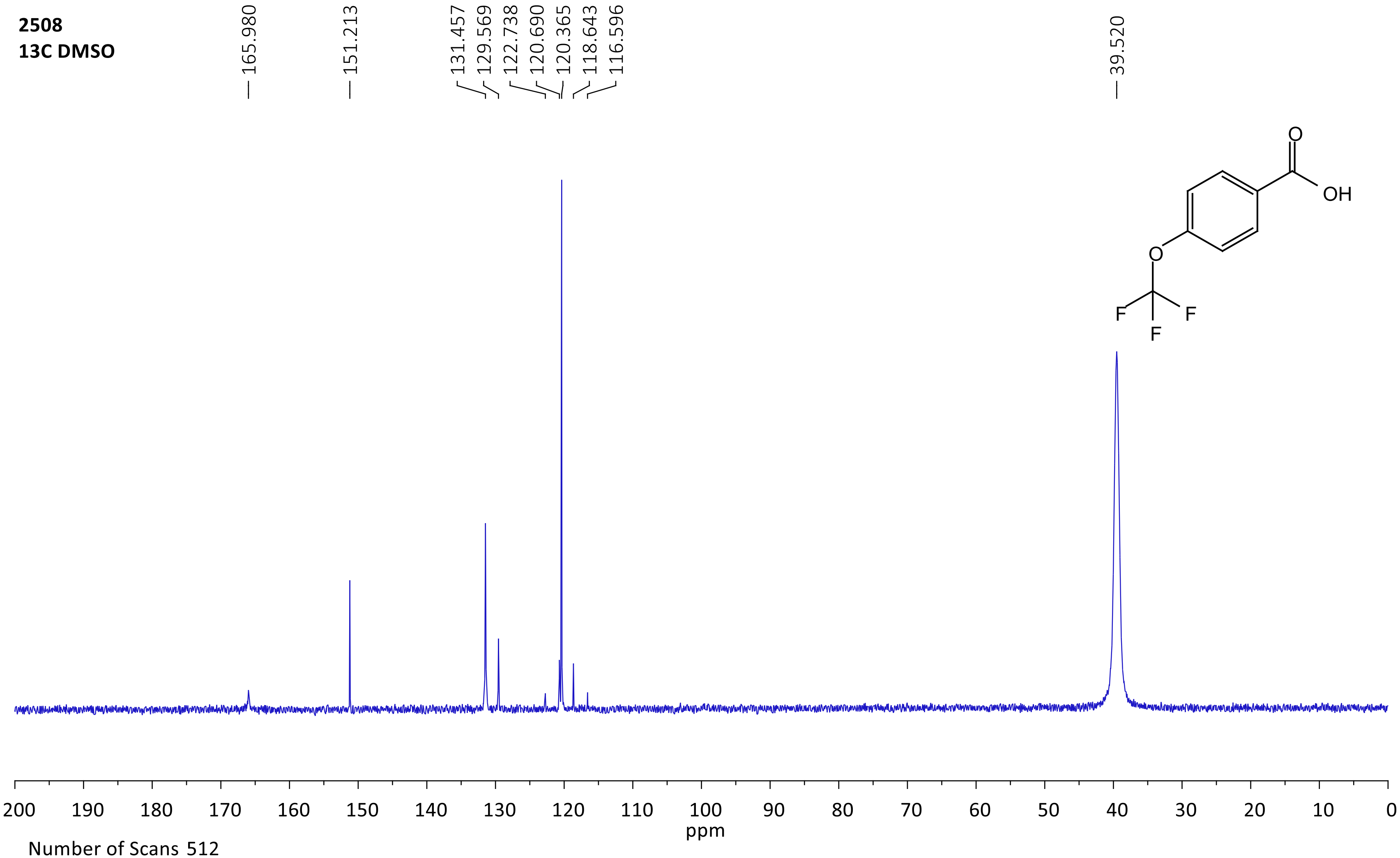

Number of Scans 512
Spectromet. Freq.125.76
Spectral Width 36057.7
Spectral Size 65536
freq. of 0 ppm: 125.7577890

Compound **4r**

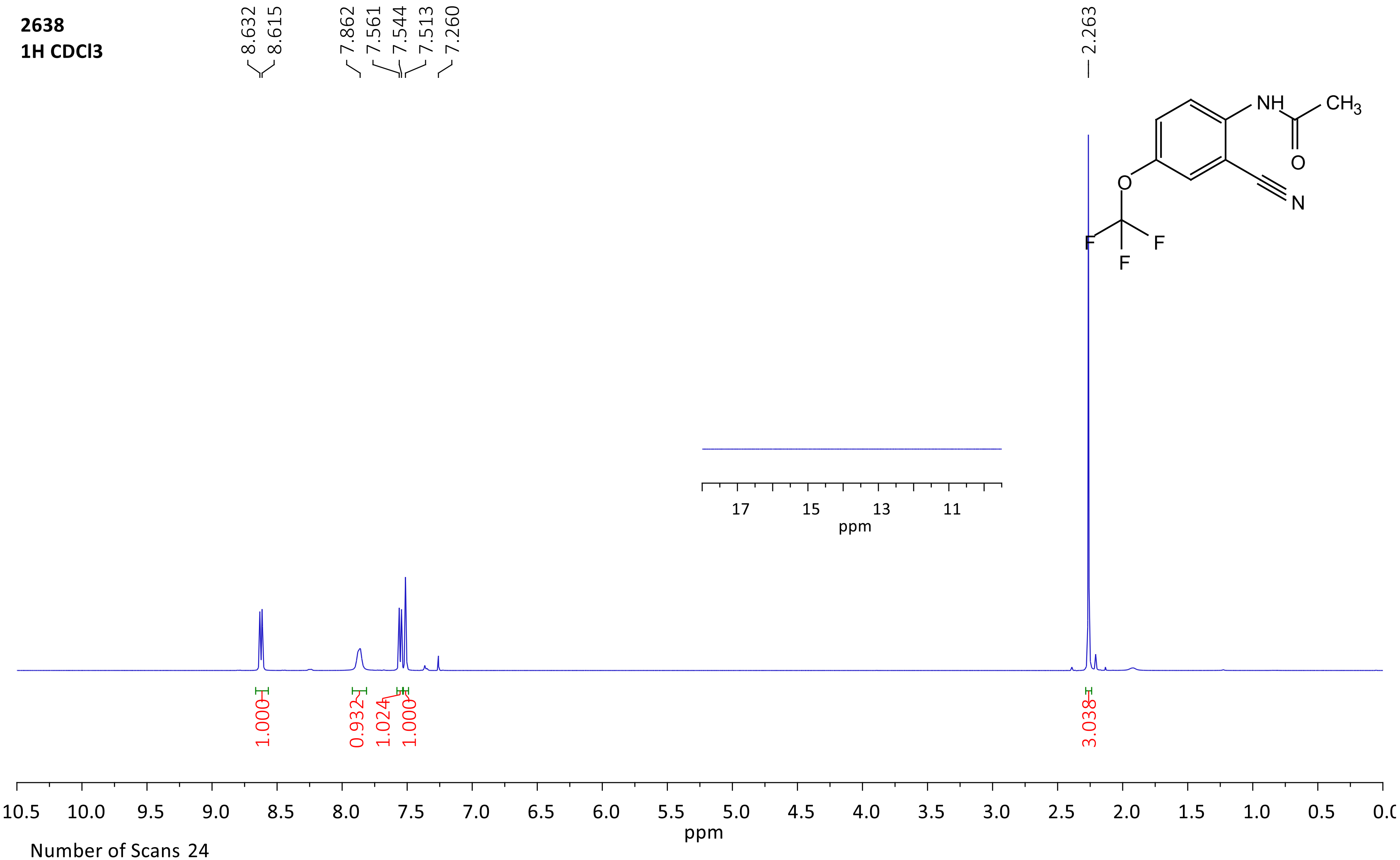

Number of Scans 24

Spectromet. Freq.500.13

Spectral Width 12335.5

Spectral Size 65536

freq. of 0 ppm: 500.1300236

Compound **4r**

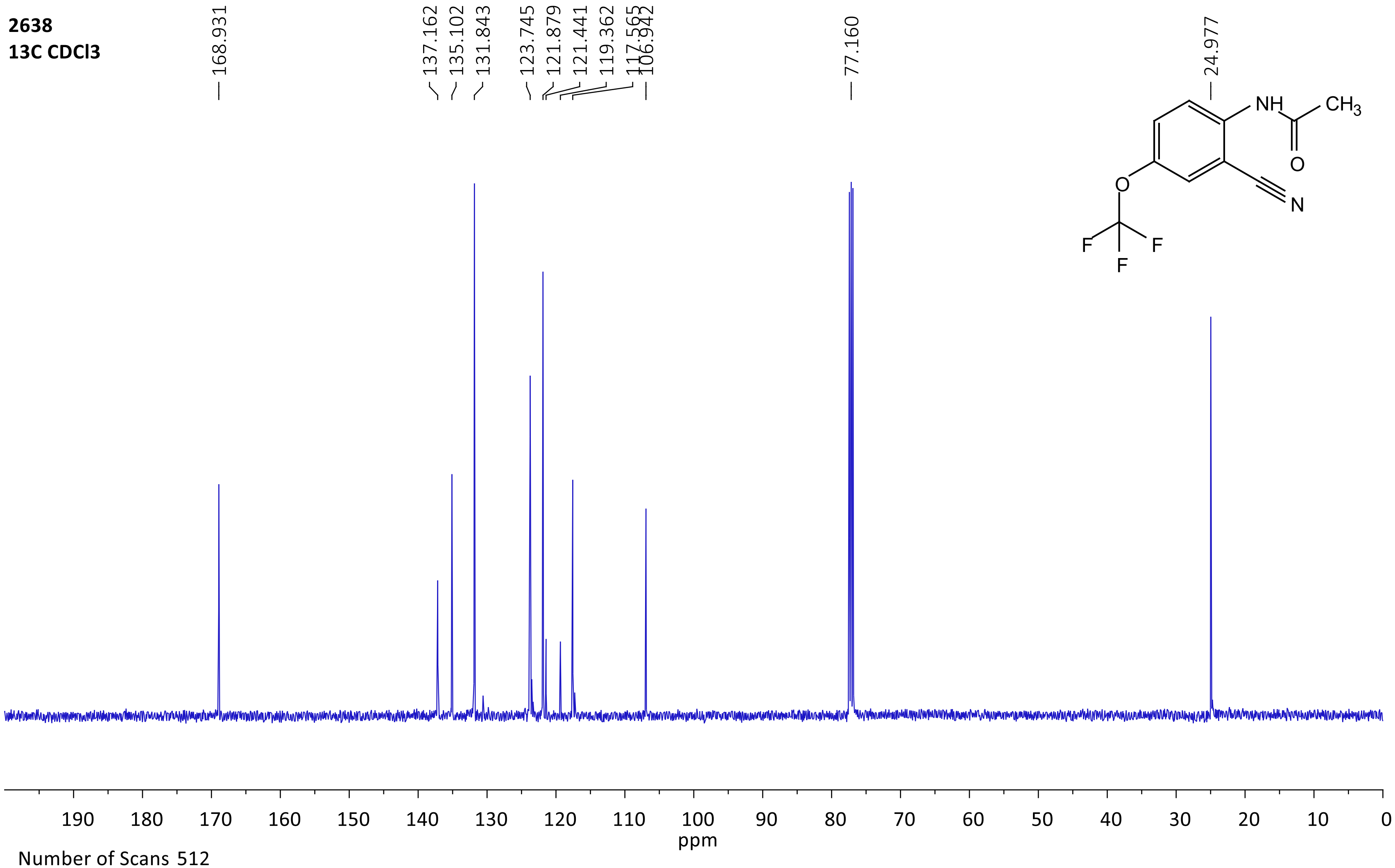

Number of Scans 512

Spectromet. Freq.125.76

Spectral Width 36057.7

Spectral Size 65536

freq. of 0 ppm: 125.7577890

Compound **4s**

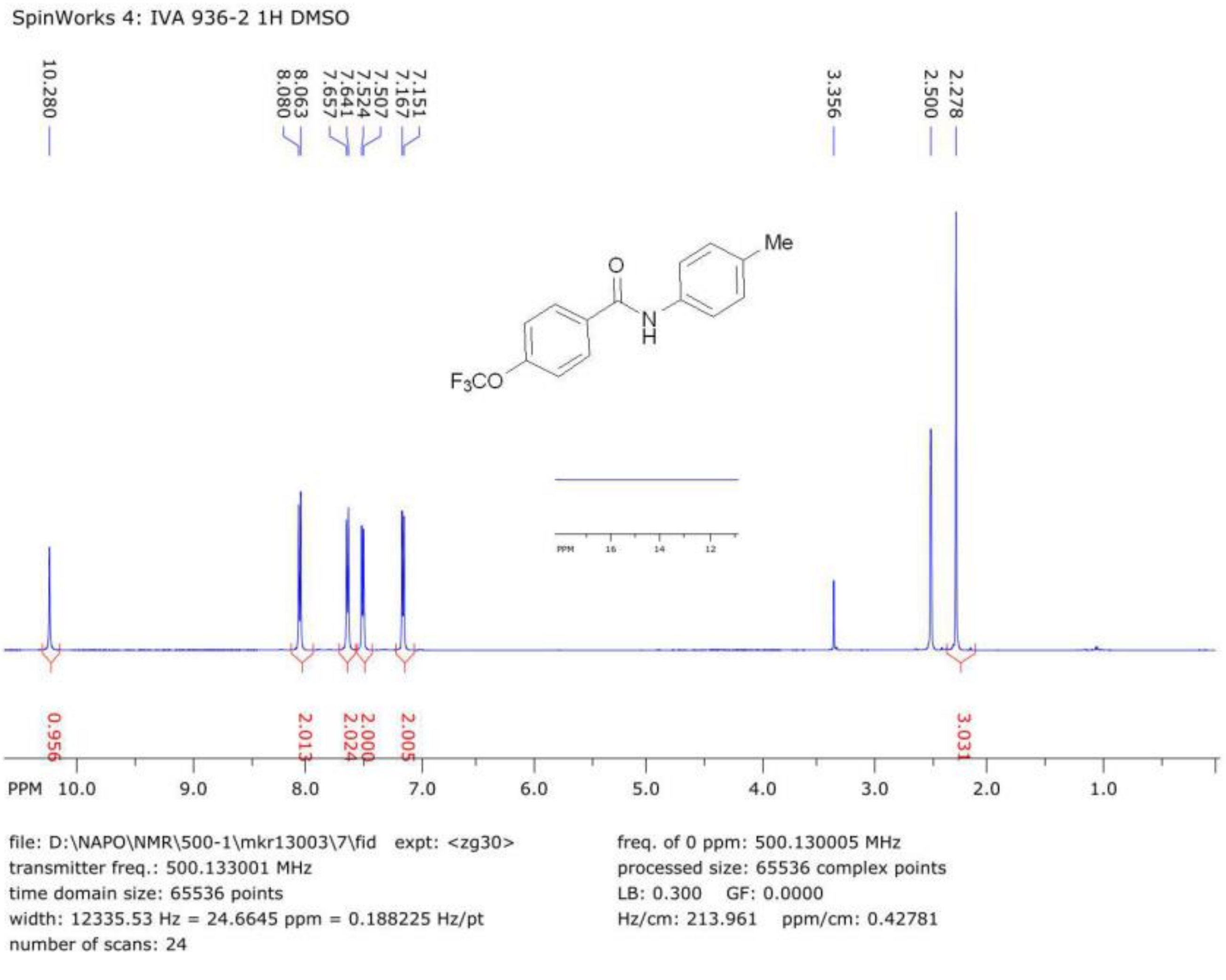

SpinWorks 4: IVA 936-2 1H DMSO
10.280
8.080
8.063
7.657
7.641
7.524
7.507
7.167
7.151
3.356
2.500
2.278
Me
O
N
H
F3CO
0.956
2.013
2.024
2.000
2.005
3.031
PPM 10.0 9.0 8.0 7.0 6.0 5.0 4.0 3.0 2.0 1.0
file: D:\NAPO\NMR\500-1\mkr13003\7\fid expt: <zg30>
transmitter freq.: 500.133001 MHz
time domain size: 65536 points
width: 12335.53 Hz = 24.6645 ppm = 0.188225 Hz/pt
number of scans: 24
freq. of 0 ppm: 500.130005 MHz
processed size: 65536 complex points
LB: 0.300 GF: 0.0000
Hz/cm: 213.961 ppm/cm: 0.42781

Compound **4s**

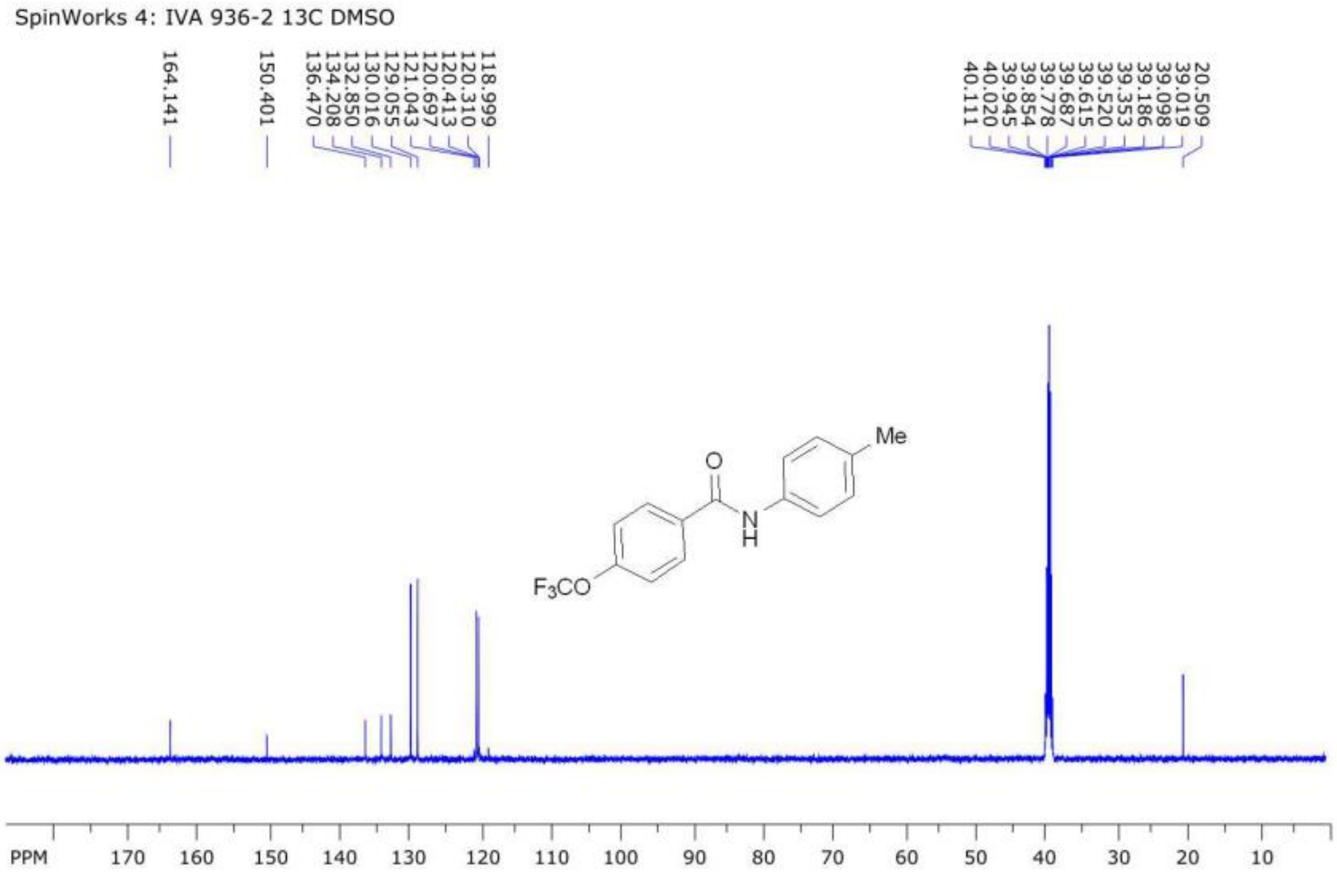

file: D:\NAPO\NMR\500-1\mkr13003\8\fid expt: <zgpg30>
transmitter freq.: 125.772879 MHz
time domain size: 65536 points
width: 36057.69 Hz = 286.6889 ppm = 0.550197 Hz/pt
number of scans: 256

freq. of 0 ppm: 125.757842 MHz
processed size: 32768 complex points
LB: 2.000 GF: 0.0000
Hz/cm: 941.329 ppm/cm: 7.48436

Compound **4t**

2186
1H CDCl3

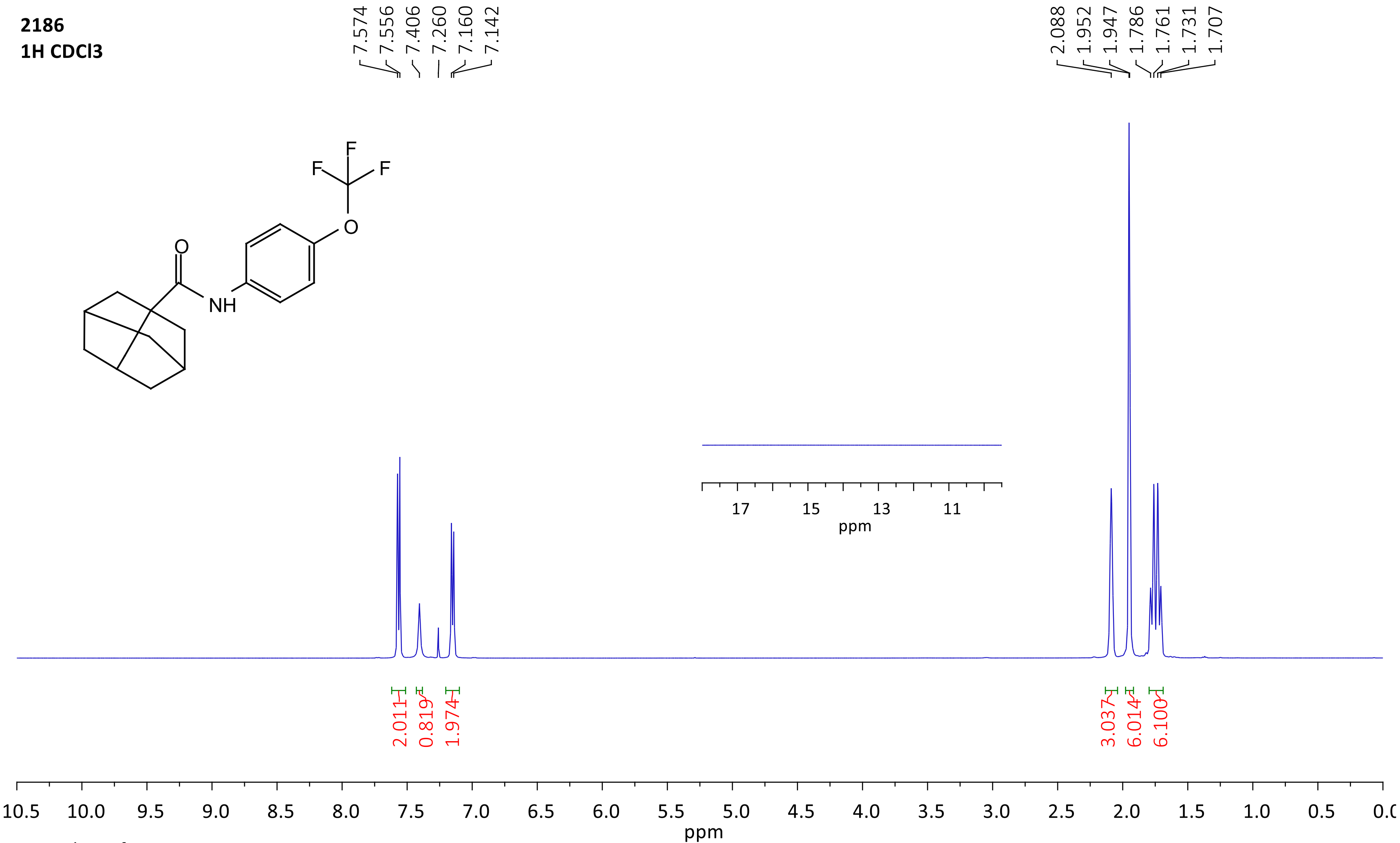

Number of Scans 24
Spectromet. Freq.500.13
Spectral Width 12335.5
Spectral Size 65536
freq. of 0 ppm: 500.1300236

Compound **4t**

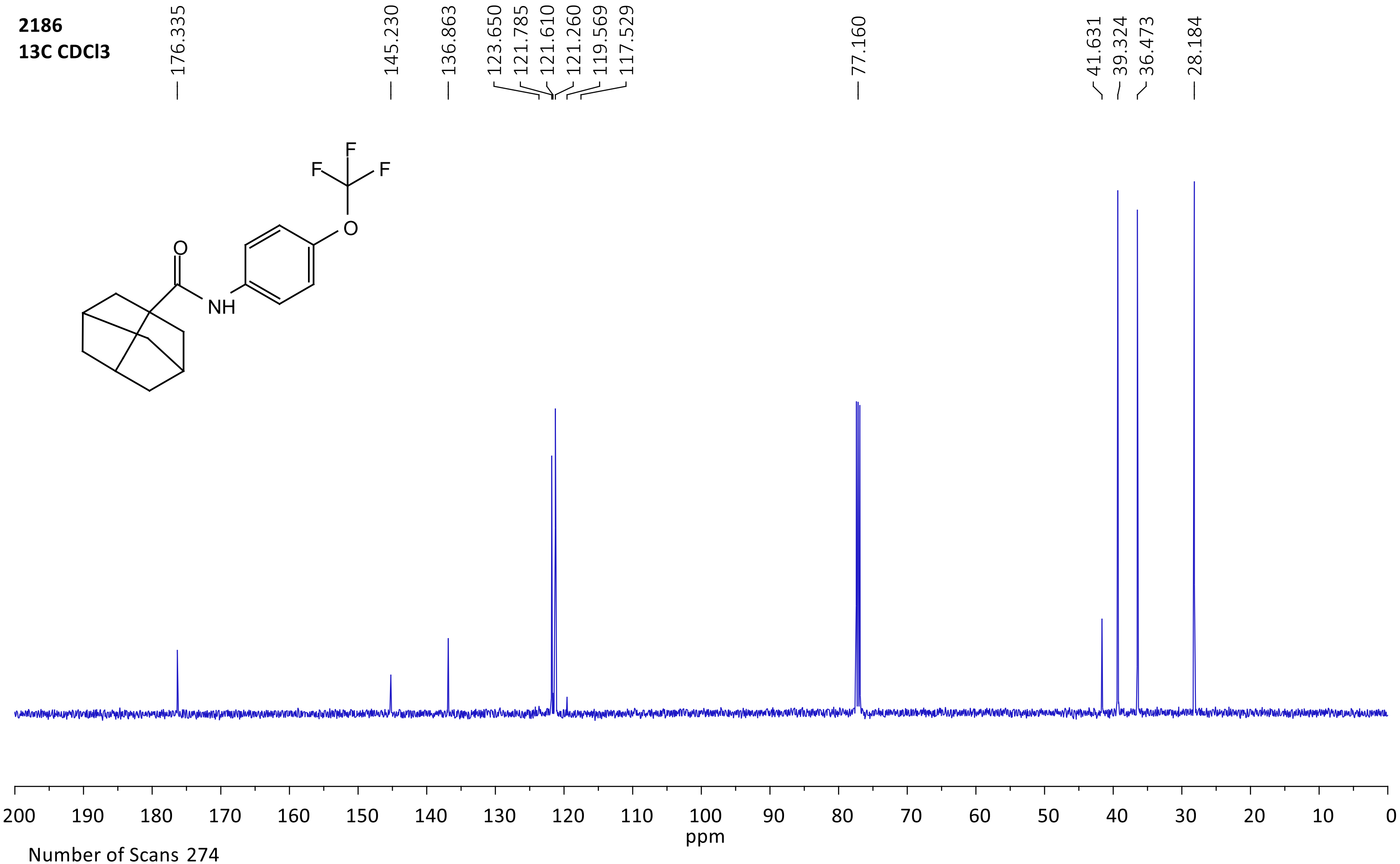

Number of Scans 274

Spectromet. Freq.125.76

Spectral Width 36057.7

Spectral Size 65536

freq. of 0 ppm: 125.7577890

Compound **4u**

SpinWorks 4: IVA 1419 1H CDCl3

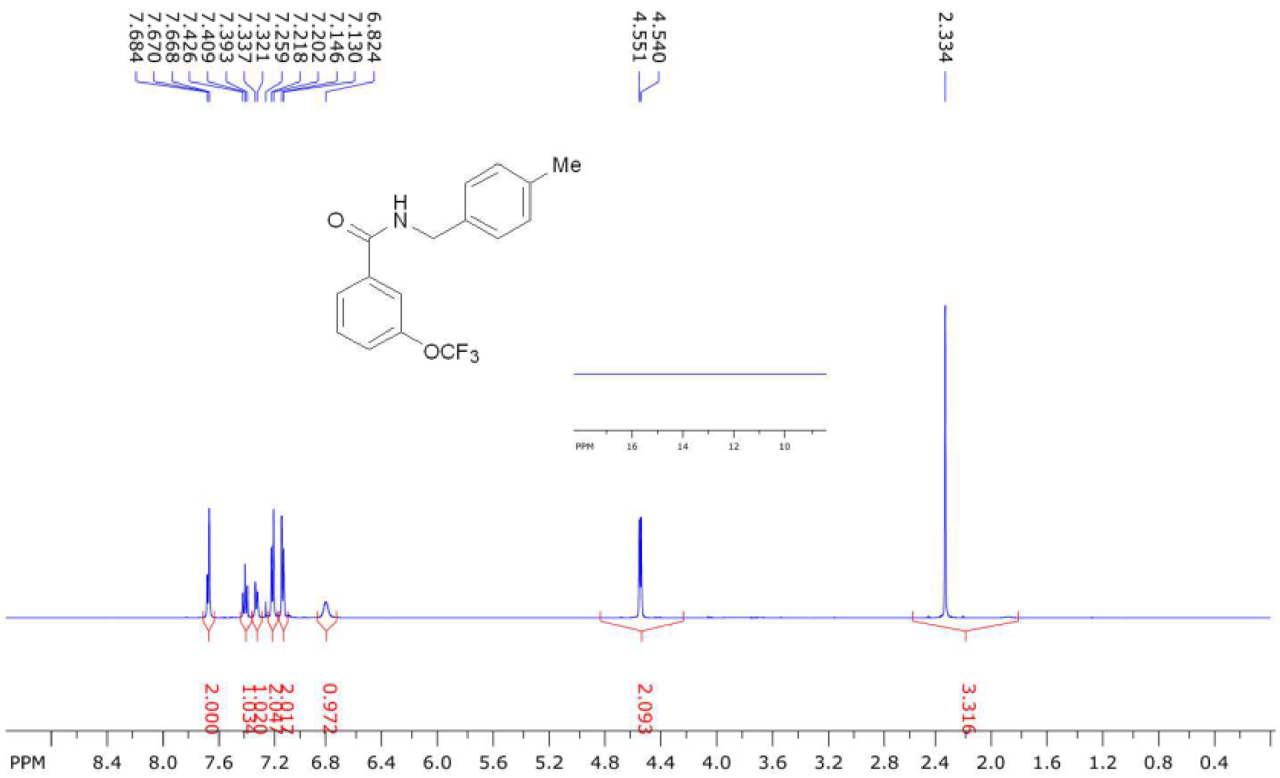

file: D:\NAPO\NMR\500-2\mkr11712\23\fid expt: <zg30>
transmitter freq.: 500.133001 MHz
time domain size: 65536 points
width: 12335.53 Hz = 24.6645 ppm = 0.188225 Hz/pt
number of scans: 24

freq. of 0 ppm: 500.130023 MHz
processed size: 65536 complex points
LB: 0.300 GF: 0.0000
Hz/cm: 183.395 ppm/cm: 0.36669

Compound **4u**

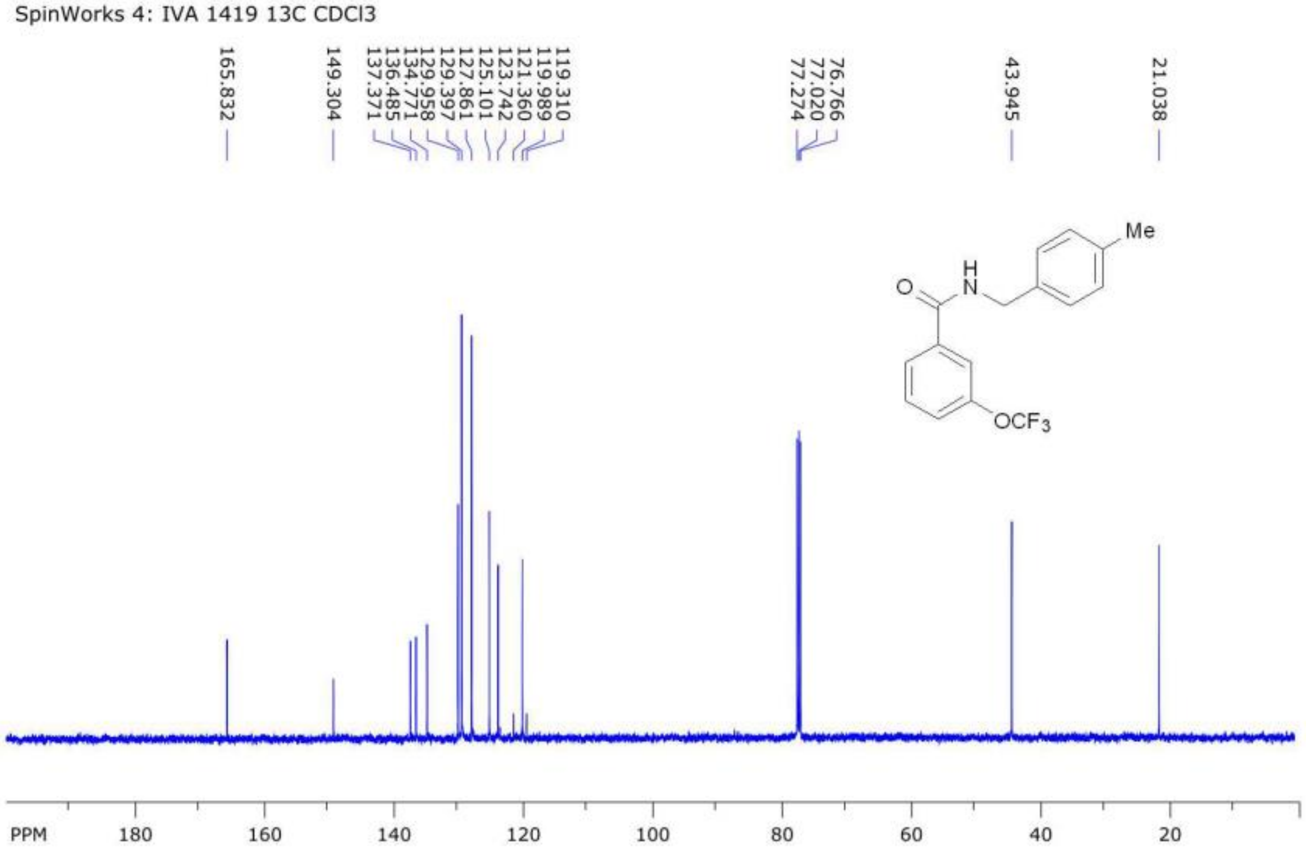

file: D:\NAPO\NMR\500-2\mkr11712\24\fid expt: <zgpg30>
transmitter freq.: 125.772879 MHz
time domain size: 65536 points
width: 36057.69 Hz = 286.6889 ppm = 0.550197 Hz/pt
number of scans: 256

freq. of 0 ppm: 125.757798 MHz
processed size: 32768 complex points
LB: 2.000 GF: 0.0000
Hz/cm: 1006.744 ppm/cm: 8.00446

Compound **4v**

SpinWorks 4: IVA 1994 1H DMSO

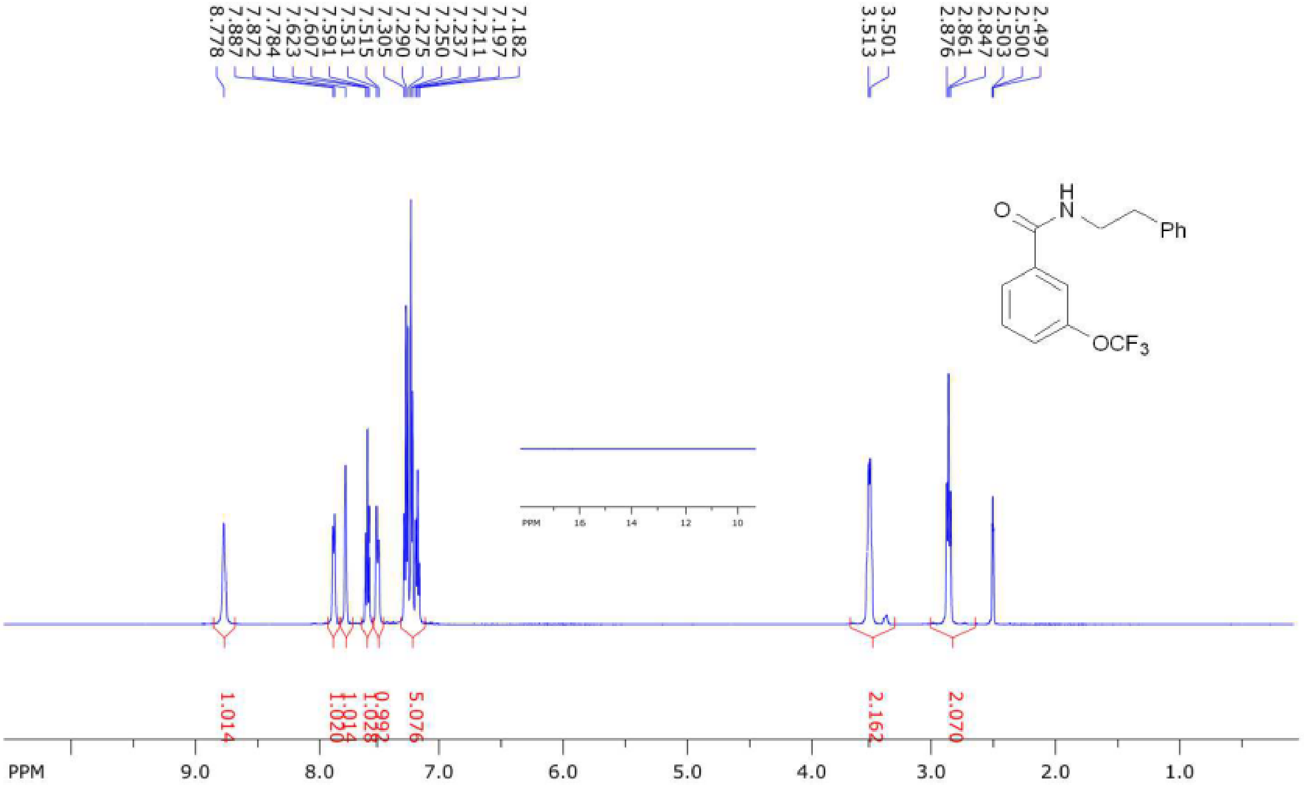

file: D:\NAPO\NMR\500-2\mkr10204\1\fid expt: <zg30>
transmitter freq.: 500.133001 MHz
time domain size: 65536 points
width: 12335.53 Hz = 24.6645 ppm = 0.188225 Hz/pt
number of scans: 24

freq. of 0 ppm: 500.130004 MHz
processed size: 65536 complex points
LB: 0.300 GF: 0.0000
Hz/cm: 210.686 ppm/cm: 0.42126

Compound **4v**

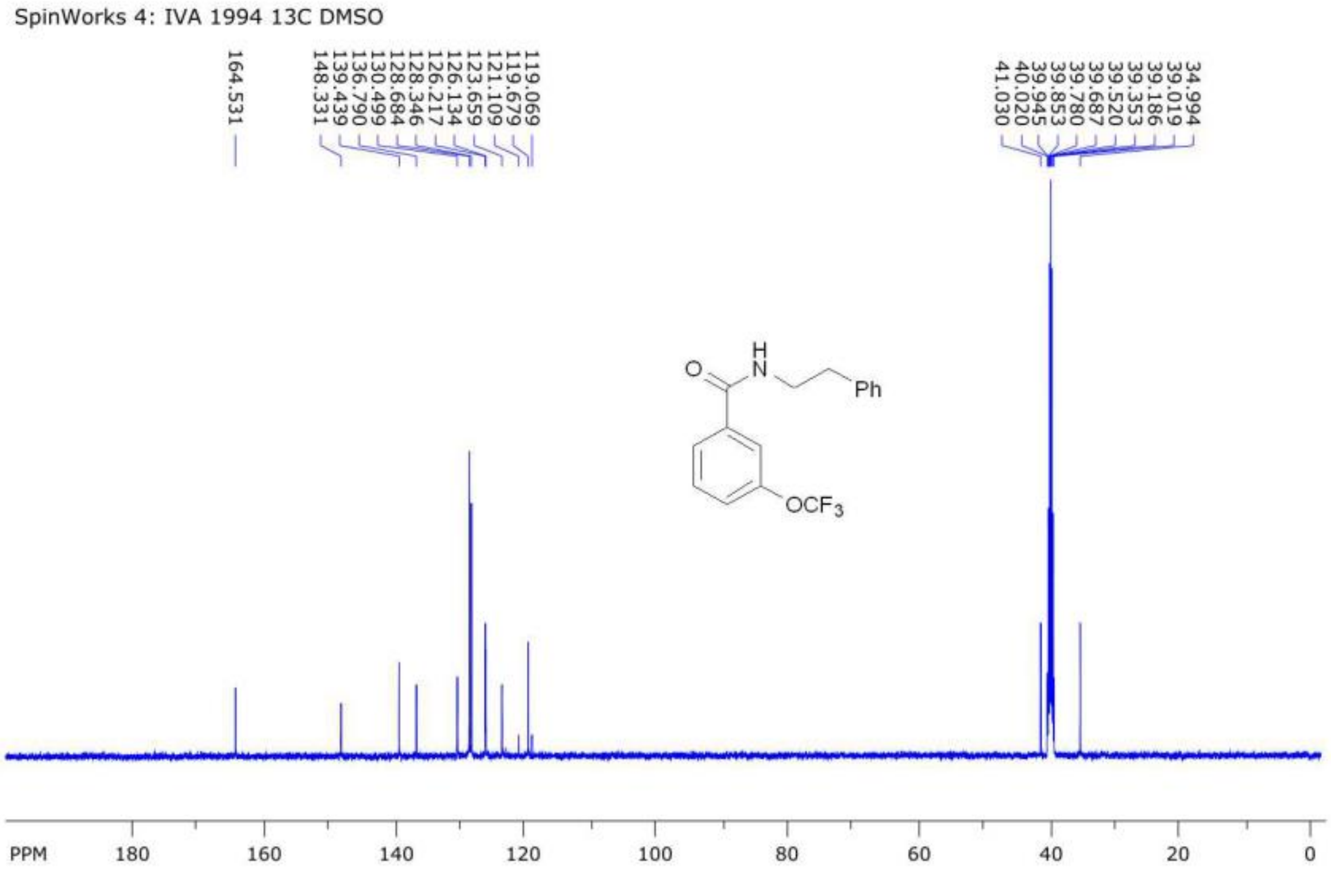

file: D:\NAPO\NMR\500-2\mkr10204\2\fid expt: <zgpg30>
transmitter freq.: 125.772879 MHz
time domain size: 65536 points
width: 36057.69 Hz = 286.6889 ppm = 0.550197 Hz/pt
number of scans: 256

freq. of 0 ppm: 125.757842 MHz
processed size: 32768 complex points
LB: 2.000 GF: 0.0000
Hz/cm: 1014.721 ppm/cm: 8.06788

Compound **4w**

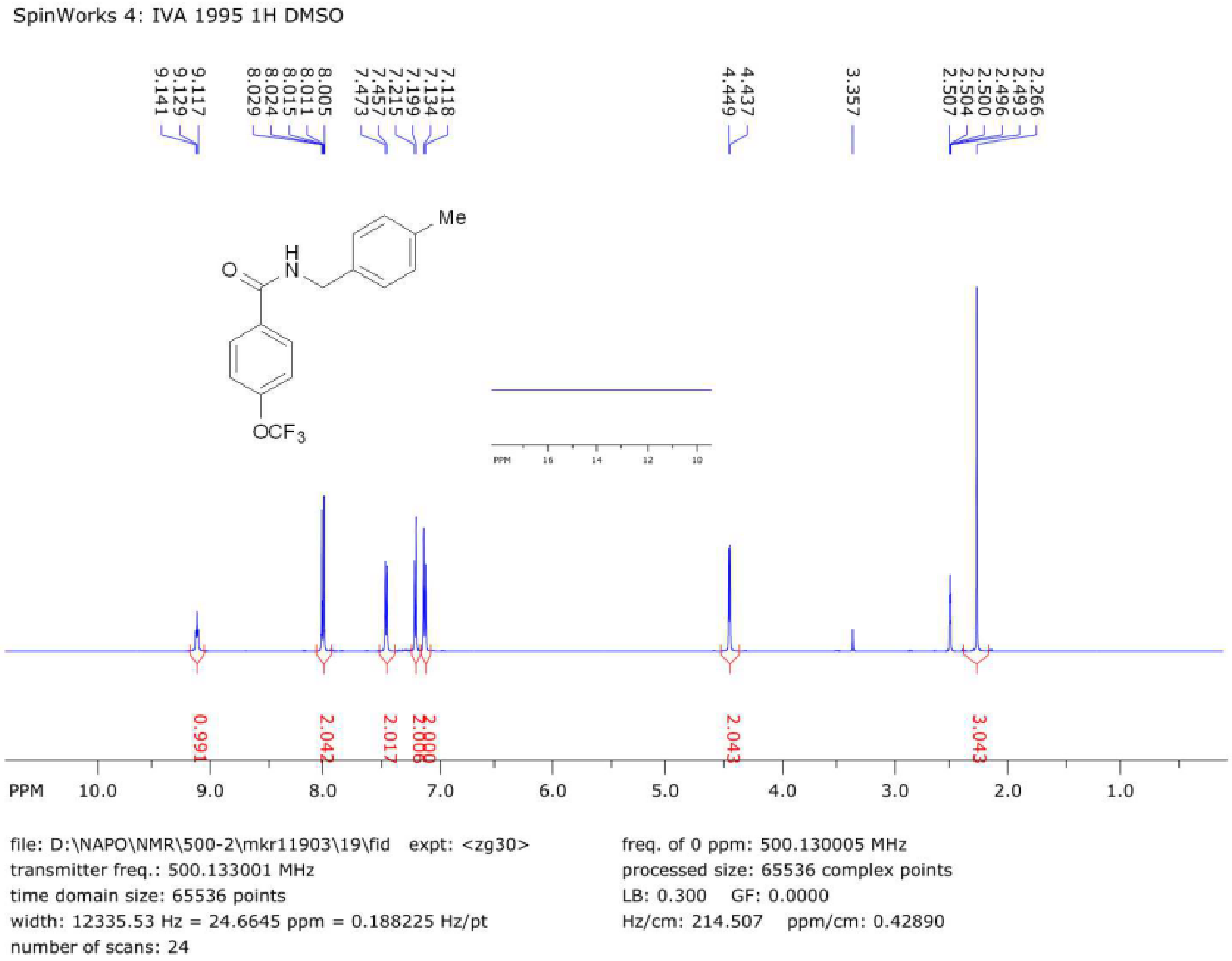
SpinWorks 4: IVA 1995 1H DMSO
9.141 9.129 9.117 8.029 8.024 8.015 8.011 8.005 7.473 7.457 7.215 7.199 7.134 7.118
4.449 4.437
3.357
2.507 2.504 2.500 2.496 2.493 2.266
Me
H
N
O
OCF3
0.991 2.042 2.017 2.880 2.043 3.043
PPM 10.0 9.0 8.0 7.0 6.0 5.0 4.0 3.0 2.0 1.0
file: D:\NAPO\NMR\500-2\mkr11903\19\fid expt: <zg30>
transmitter freq.: 500.133001 MHz
time domain size: 65536 points
width: 12335.53 Hz = 24.6645 ppm = 0.188225 Hz/pt
number of scans: 24
freq. of 0 ppm: 500.130005 MHz
processed size: 65536 complex points
LB: 0.300 GF: 0.0000
Hz/cm: 214.507 ppm/cm: 0.42890

Compound **4w**

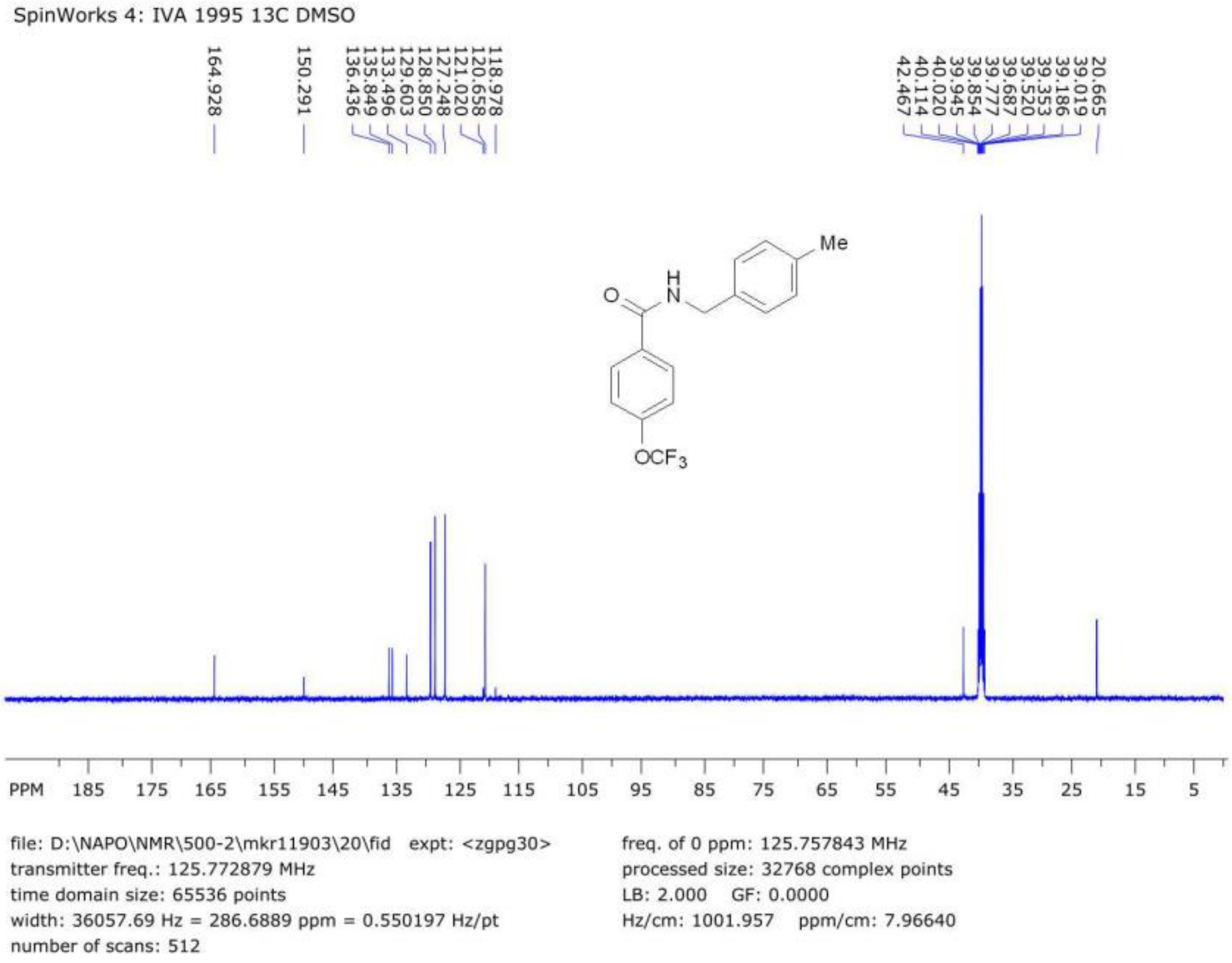
SpinWorks 4: IVA 1995 13C DMSO
164.928
150.291
136.436
135.849
133.496
129.603
128.850
127.248
121.020
120.658
118.978
42.467
40.114
40.020
39.945
39.854
39.777
39.687
39.520
39.353
39.186
39.019
20.665
Me
H
N
O
OCF3
PPM 185 175 165 155 145 135 125 115 105 95 85 75 65 55 45 35 25 15 5
file: D:\NAPO\NMR\500-2\mkr11903\20\fid expt: <zgpg30>
transmitter freq.: 125.772879 MHz
time domain size: 65536 points
width: 36057.69 Hz = 286.6889 ppm = 0.550197 Hz/pt
number of scans: 512
freq. of 0 ppm: 125.757843 MHz
processed size: 32768 complex points
LB: 2.000 GF: 0.0000
Hz/cm: 1001.957 ppm/cm: 7.96640

Compound **4x**

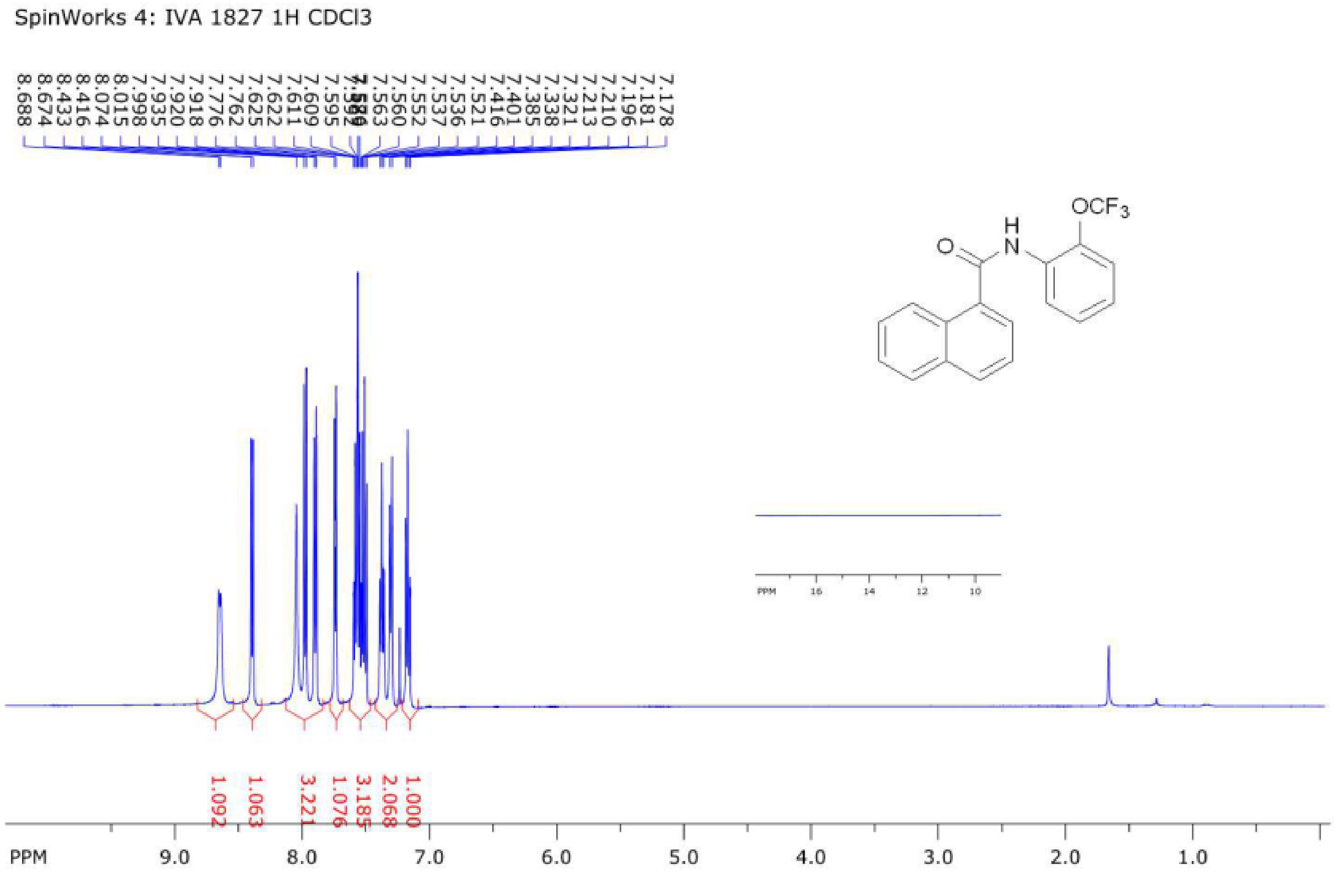

file: ...APO\NMR\500-2\mkr12206\15 1827\fid expt: <zg30>
transmitter freq.: 500.133001 MHz
time domain size: 65536 points
width: 12335.53 Hz = 24.6645 ppm = 0.188225 Hz/pt
number of scans: 24

freq. of 0 ppm: 500.130023 MHz
processed size: 65536 complex points
LB: 0.300 GF: 0.0000
Hz/cm: 208.503 ppm/cm: 0.41690

Compound **4x**

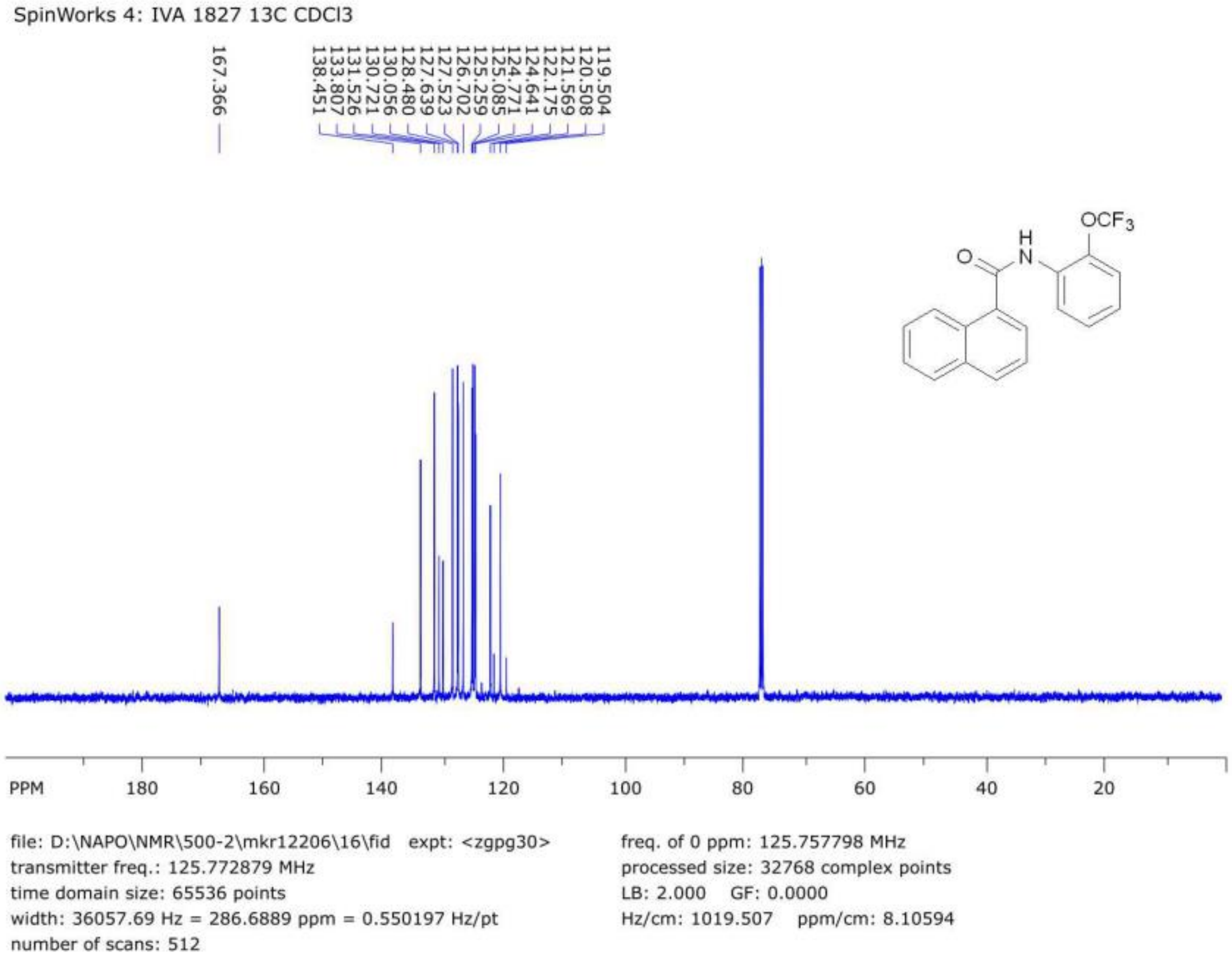
SpinWorks 4: IVA 1827 13C CDCl3
167.366
138.451
133.807
131.526
130.721
130.056
128.480
127.639
127.523
126.702
125.259
125.085
124.771
124.641
122.175
121.569
120.508
119.504
OCF3
H
N
O
PPM
180
160
140
120
100
80
60
40
20
file: D:\NAPO\NMR\500-2\mkr12206\16\fid expt: <zgpg30>
transmitter freq.: 125.772879 MHz
time domain size: 65536 points
width: 36057.69 Hz = 286.6889 ppm = 0.550197 Hz/pt
number of scans: 512
freq. of 0 ppm: 125.757798 MHz
processed size: 32768 complex points
LB: 2.000 GF: 0.0000
Hz/cm: 1019.507 ppm/cm: 8.10594

Compound **4y**

SpinWorks 4: IVA 1832 1H CDCl3

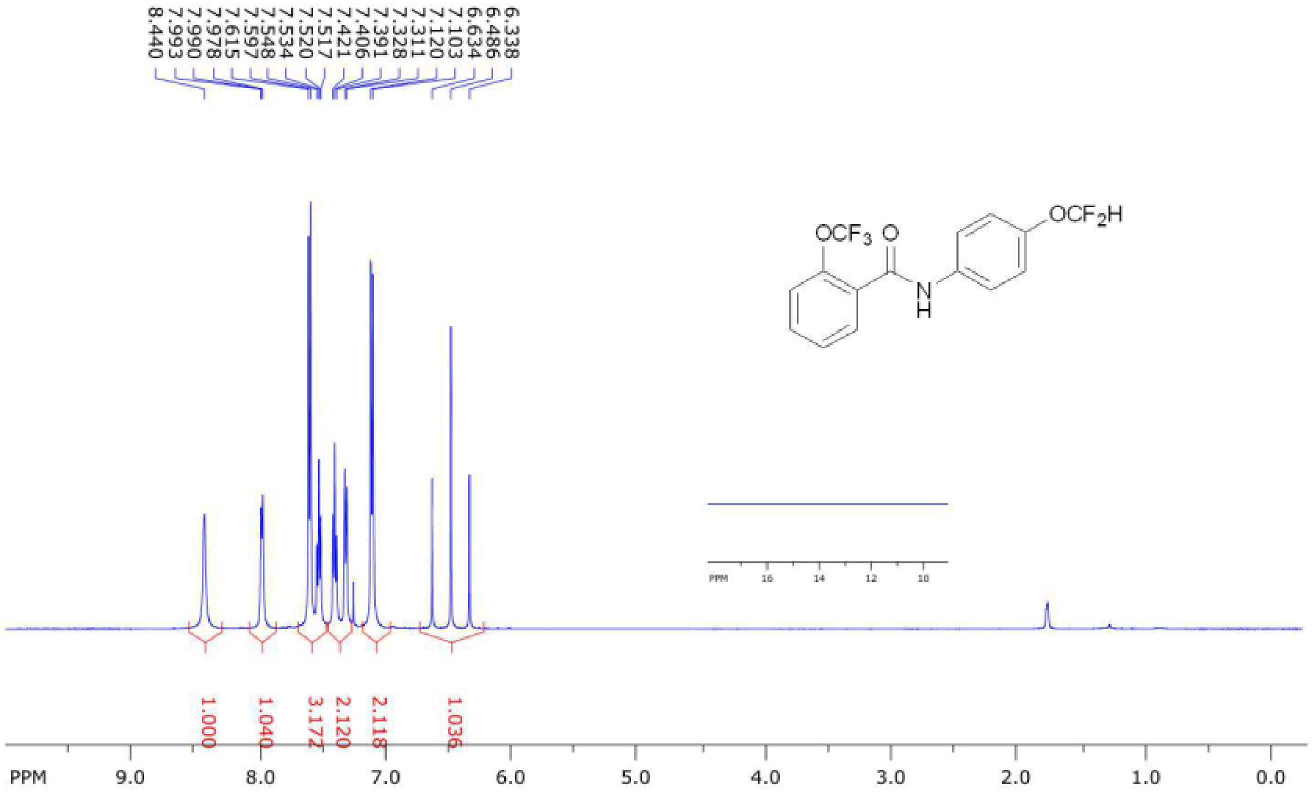

file: ...APO\NMR\500-2\mkr12206\17 1832\fid expt: <zg30>
transmitter freq.: 500.133001 MHz
time domain size: 65536 points
width: 12335.53 Hz = 24.6645 ppm = 0.188225 Hz/pt
number of scans: 24

freq. of 0 ppm: 500.130023 MHz
processed size: 65536 complex points
LB: 0.300 GF: 0.0000
Hz/cm: 205.774 ppm/cm: 0.41144

Compound **4y**

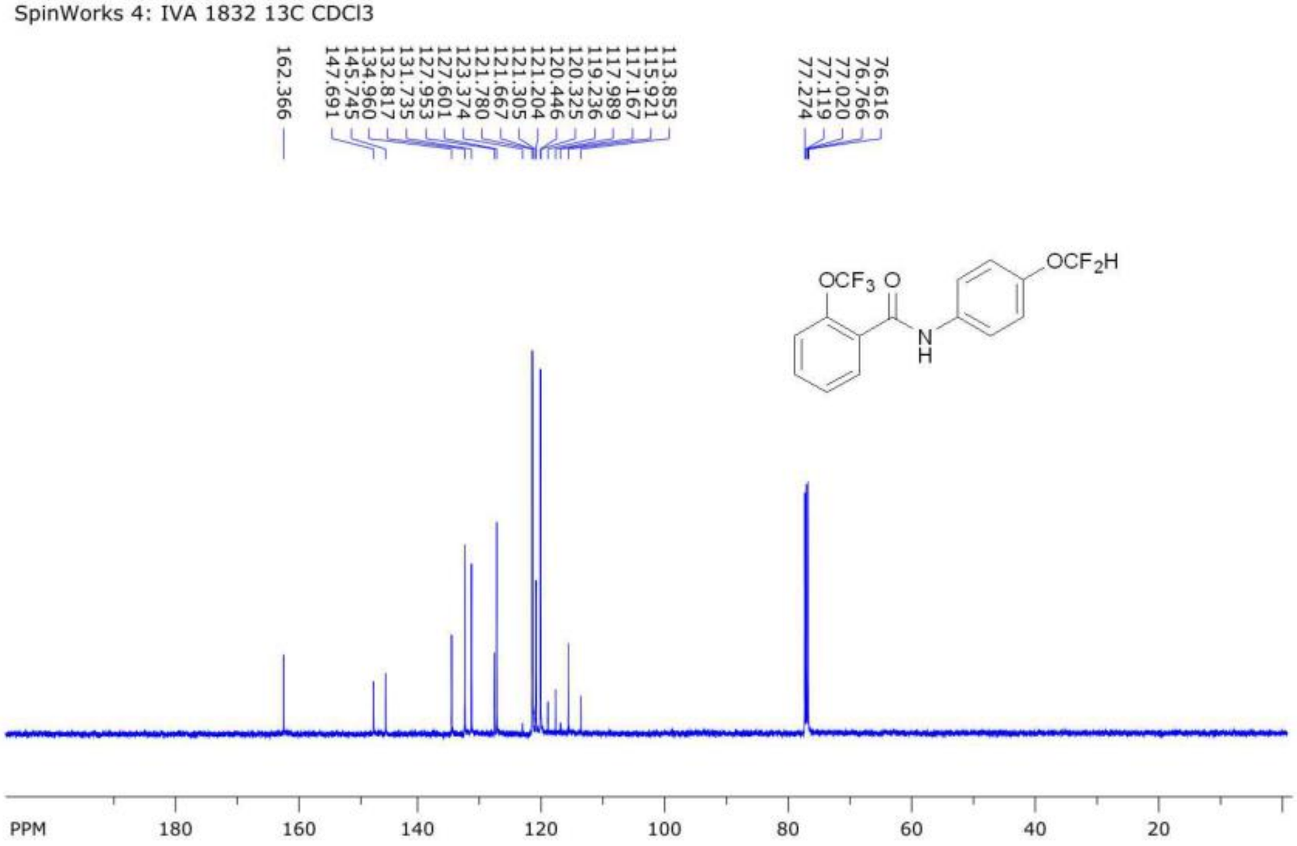

file: D:\NAPO\NMR\500-2\mkr12206\18\fid expt: <zgpg30>
transmitter freq.: 125.772879 MHz
time domain size: 65536 points
width: 36057.69 Hz = 286.6889 ppm = 0.550197 Hz/pt
number of scans: 512

freq. of 0 ppm: 125.757795 MHz
processed size: 32768 complex points
LB: 2.000 GF: 0.0000
Hz/cm: 1053.012 ppm/cm: 8.37233

Compound **4z**

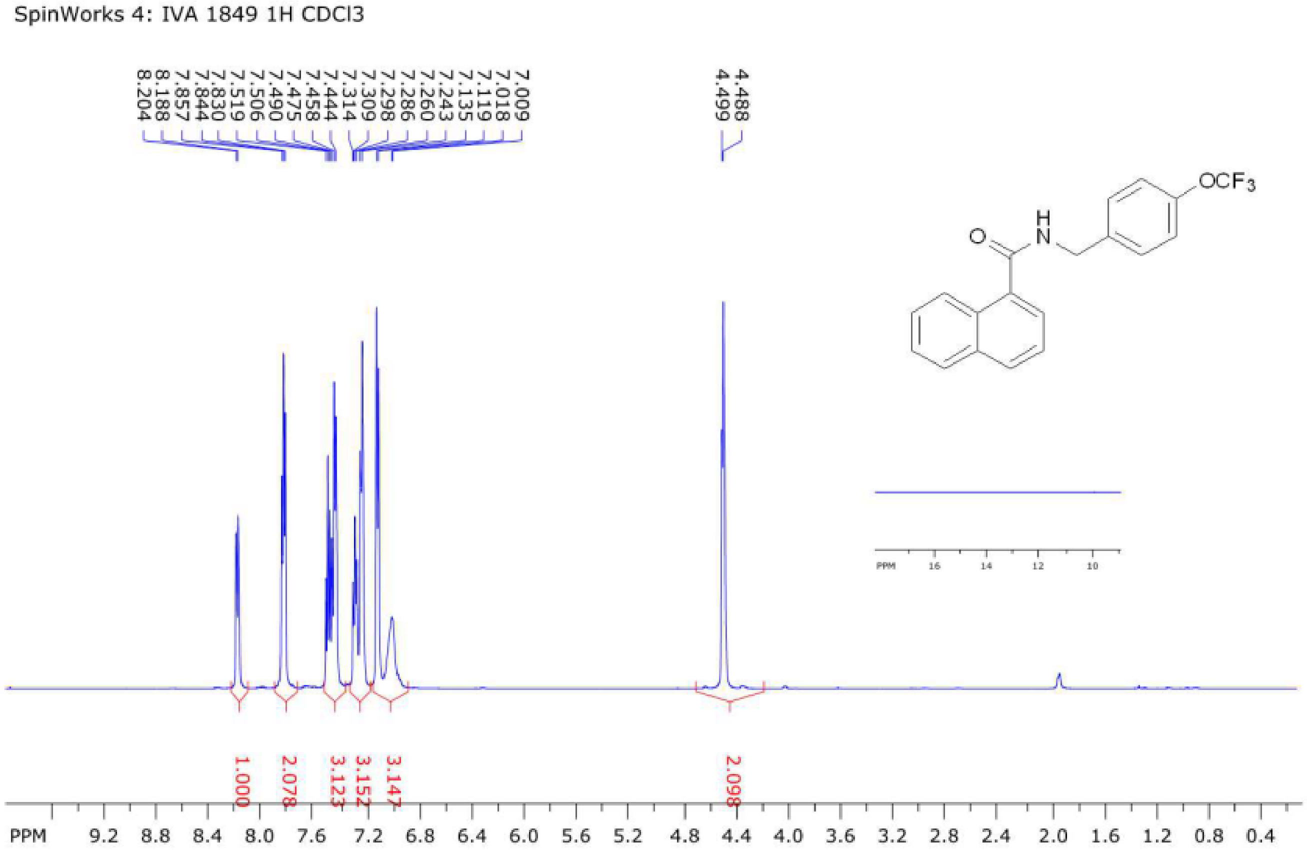

file: ...APO\NMR\500-2\mkr11306\21 1849\fid expt: <zg30>
transmitter freq.: 500.133001 MHz
time domain size: 65536 points
width: 12335.53 Hz = 24.6645 ppm = 0.188225 Hz/pt
number of scans: 24

freq. of 0 ppm: 500.130010 MHz
processed size: 65536 complex points
LB: 0.300 GF: 0.0000
Hz/cm: 197.041 ppm/cm: 0.39398

Compound **4z**

SpinWorks 4: IVA 1849 13C CDCL3

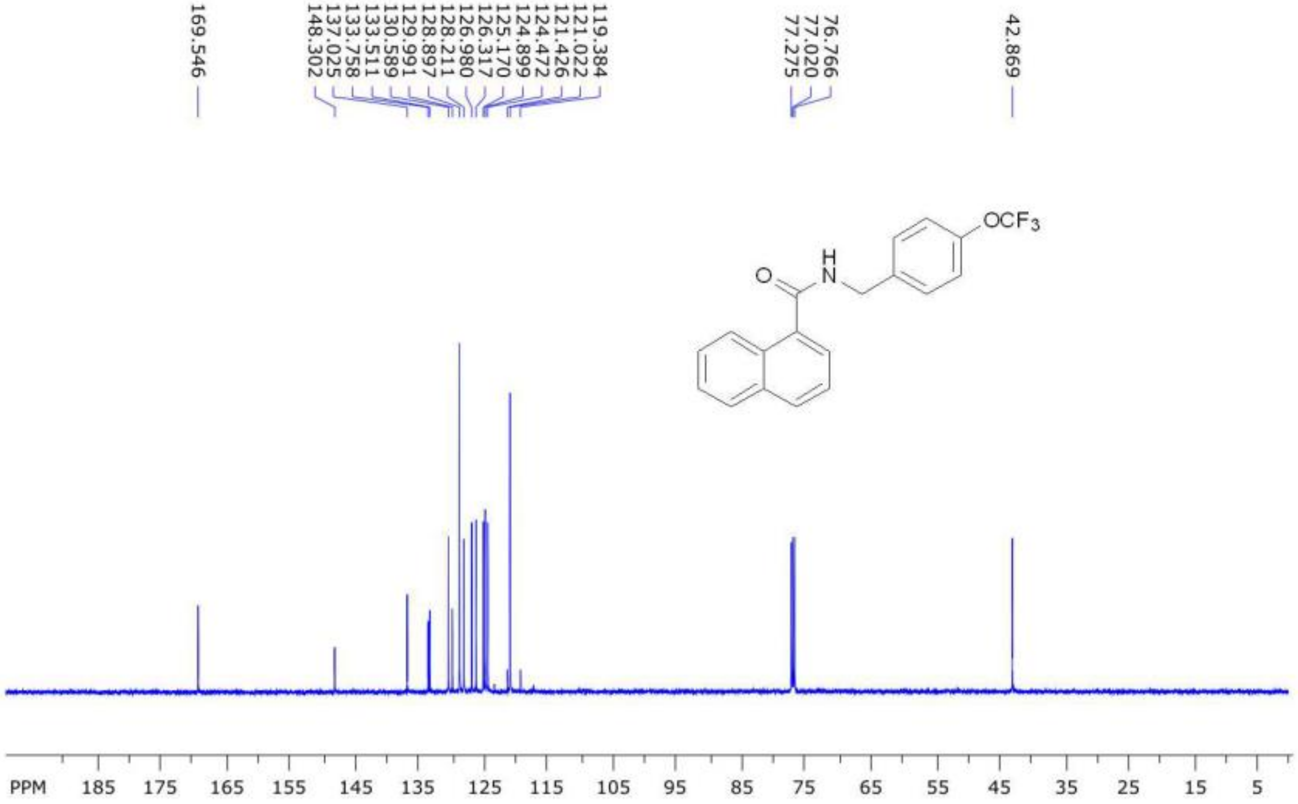

file: D:\NAPO\NMR\500-2\mkr11306\22\fid expt: <zgpg30>
transmitter freq.: 125.772879 MHz
time domain size: 65536 points
width: 36057.69 Hz = 286.6889 ppm = 0.550197 Hz/pt
number of scans: 512

freq. of 0 ppm: 125.757802 MHz
processed size: 32768 complex points
LB: 2.000 GF: 0.0000
Hz/cm: 1003.553 ppm/cm: 7.97909

Compound **4aa**

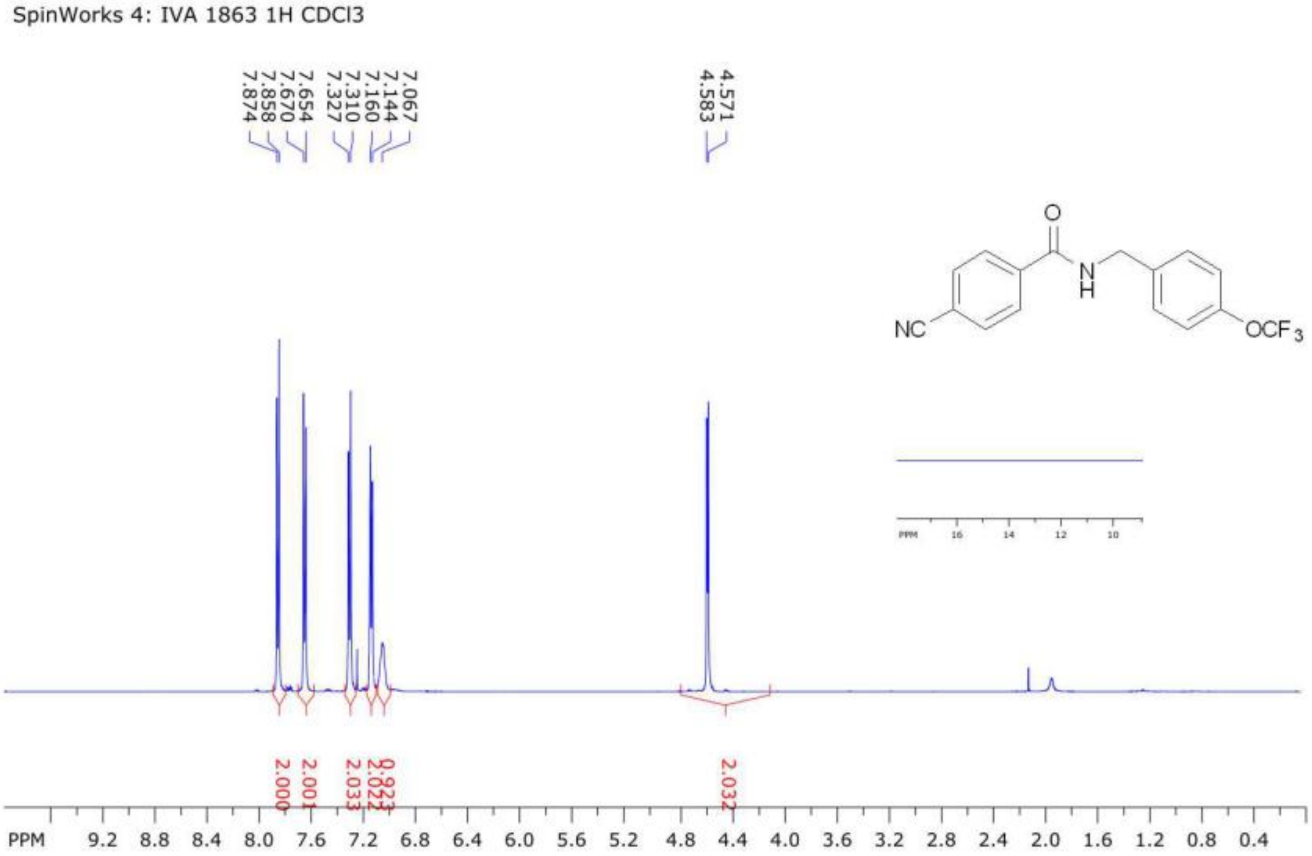

file: ...NMR\500-2\mkr12205\27 iva 1863\fid expt: <zg30>
transmitter freq.: 500.133001 MHz
time domain size: 65536 points
width: 12335.53 Hz = 24.6645 ppm = 0.188225 Hz/pt
number of scans: 24

freq. of 0 ppm: 500.130023 MHz
processed size: 65536 complex points
LB: 0.300 GF: 0.0000
Hz/cm: 198.678 ppm/cm: 0.39725

Compound **4aa**

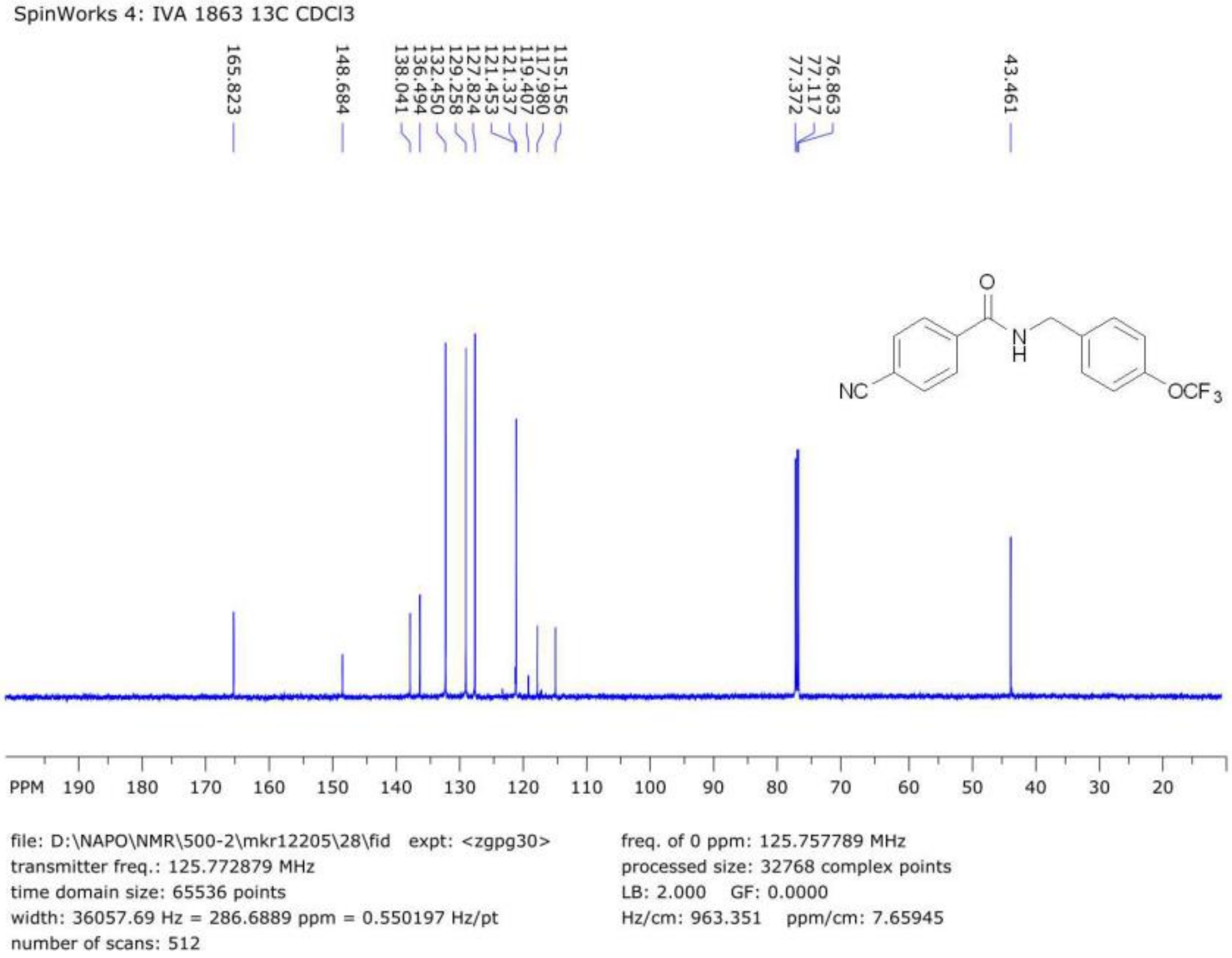

Compound **4ab**

SpinWorks 4: IVA 2351 1H DMSO

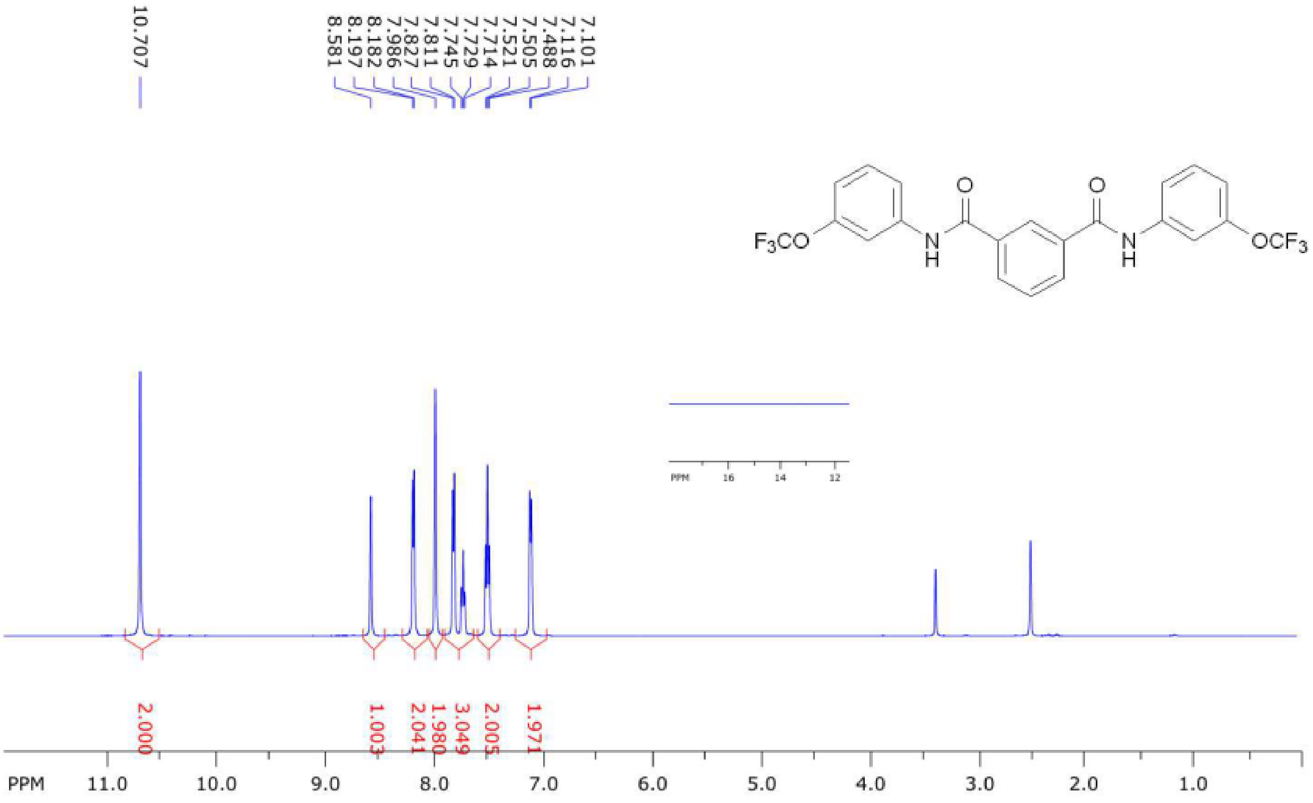

file: D:\NAPO\NMR\500-2\mkr11305\19\fid expt: <zg30>
transmitter freq.: 500.133001 MHz
time domain size: 65536 points
width: 12335.53 Hz = 24.6645 ppm = 0.188225 Hz/pt
number of scans: 24

freq. of 0 ppm: 500.130004 MHz
processed size: 65536 complex points
LB: 0.300 GF: 0.0000
Hz/cm: 238.523 ppm/cm: 0.47692

Compound **4ab**

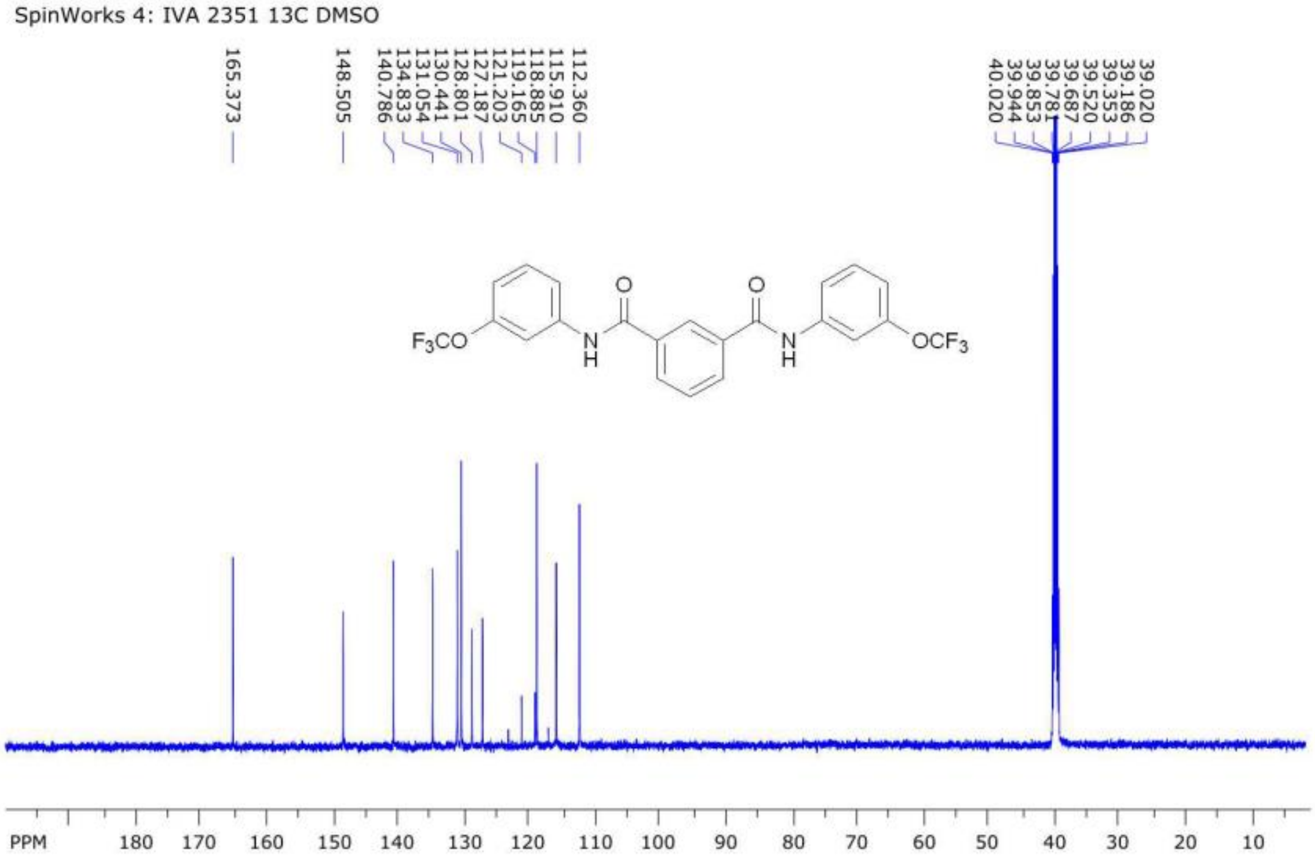

file: D:\NAPO\NMR\500-2\mkr11305\20\fid expt: <zgpg30>
transmitter freq.: 125.772879 MHz
time domain size: 65536 points
width: 36057.69 Hz = 286.6889 ppm = 0.550197 Hz/pt
number of scans: 512

freq. of 0 ppm: 125.757839 MHz
processed size: 32768 complex points
LB: 2.000 GF: 0.0000
Hz/cm: 1000.362 ppm/cm: 7.95372

Compound **4ac**

SpinWorks 4: IVA 2068 1H DMSO

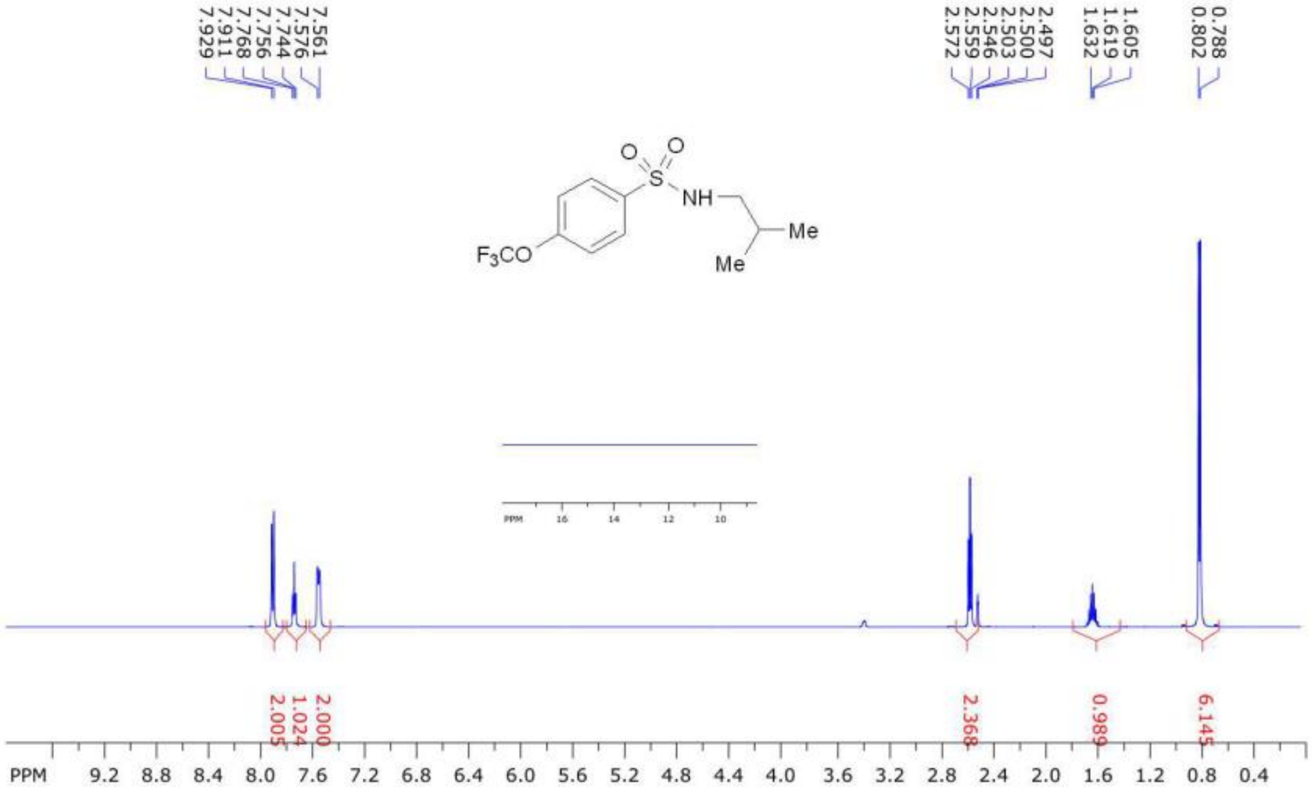

file: ...APO\NMR\500-2\mkr12207\25 2068\fid expt: <zg30>
transmitter freq.: 500.133001 MHz
time domain size: 65536 points
width: 12335.53 Hz = 24.6645 ppm = 0.188225 Hz/pt
number of scans: 24

freq. of 0 ppm: 500.130005 MHz
processed size: 65536 complex points
LB: 0.300 GF: 0.0000
Hz/cm: 199.224 ppm/cm: 0.39834

Compound **4ac**

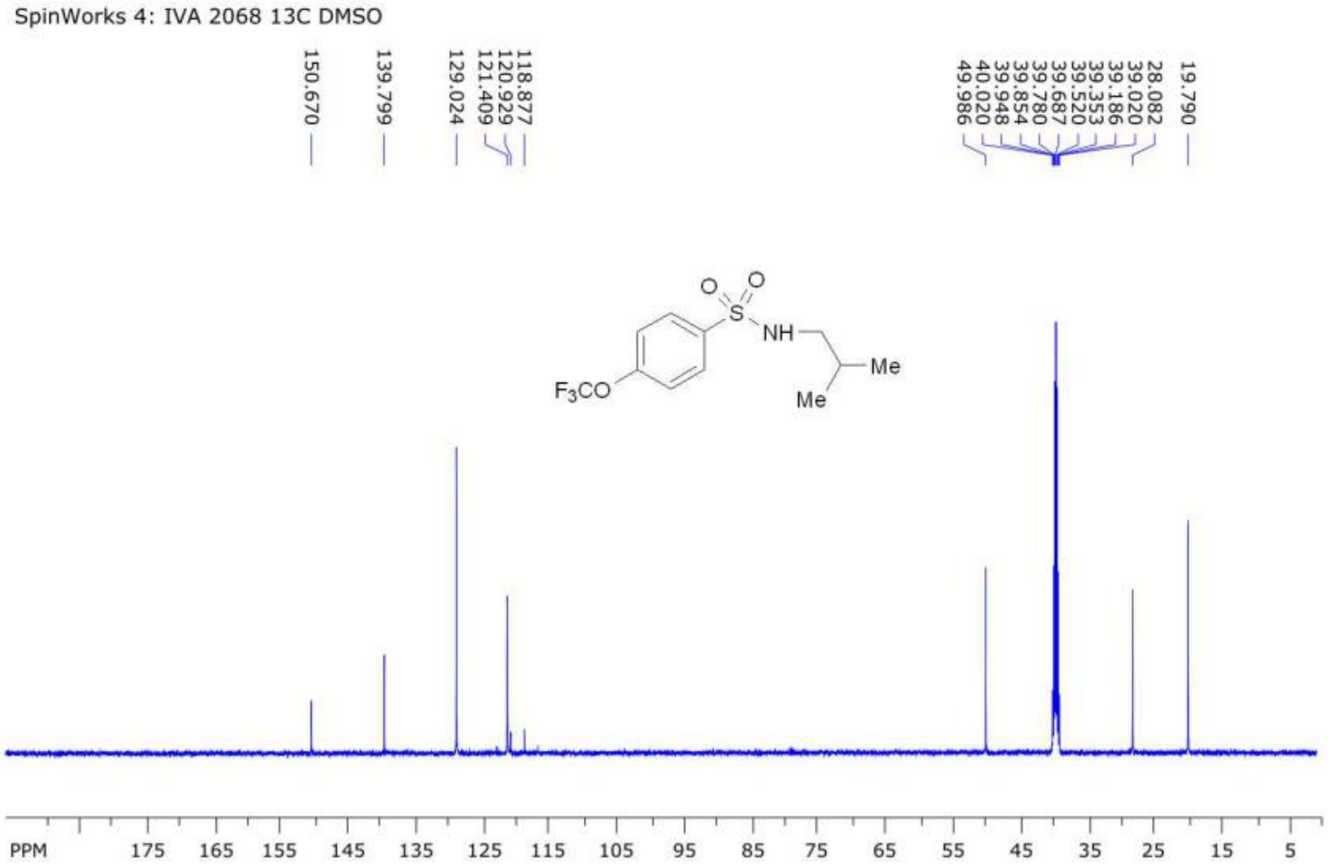

file: D:\NAPO\NMR\500-2\mkr12207\26\fid expt: <zgpg30>
transmitter freq.: 125.772879 MHz
time domain size: 65536 points
width: 36057.69 Hz = 286.6889 ppm = 0.550197 Hz/pt
number of scans: 512

freq. of 0 ppm: 125.757841 MHz
processed size: 32768 complex points
LB: 2.000 GF: 0.0000
Hz/cm: 984.407 ppm/cm: 7.82686

Compound **4ad**

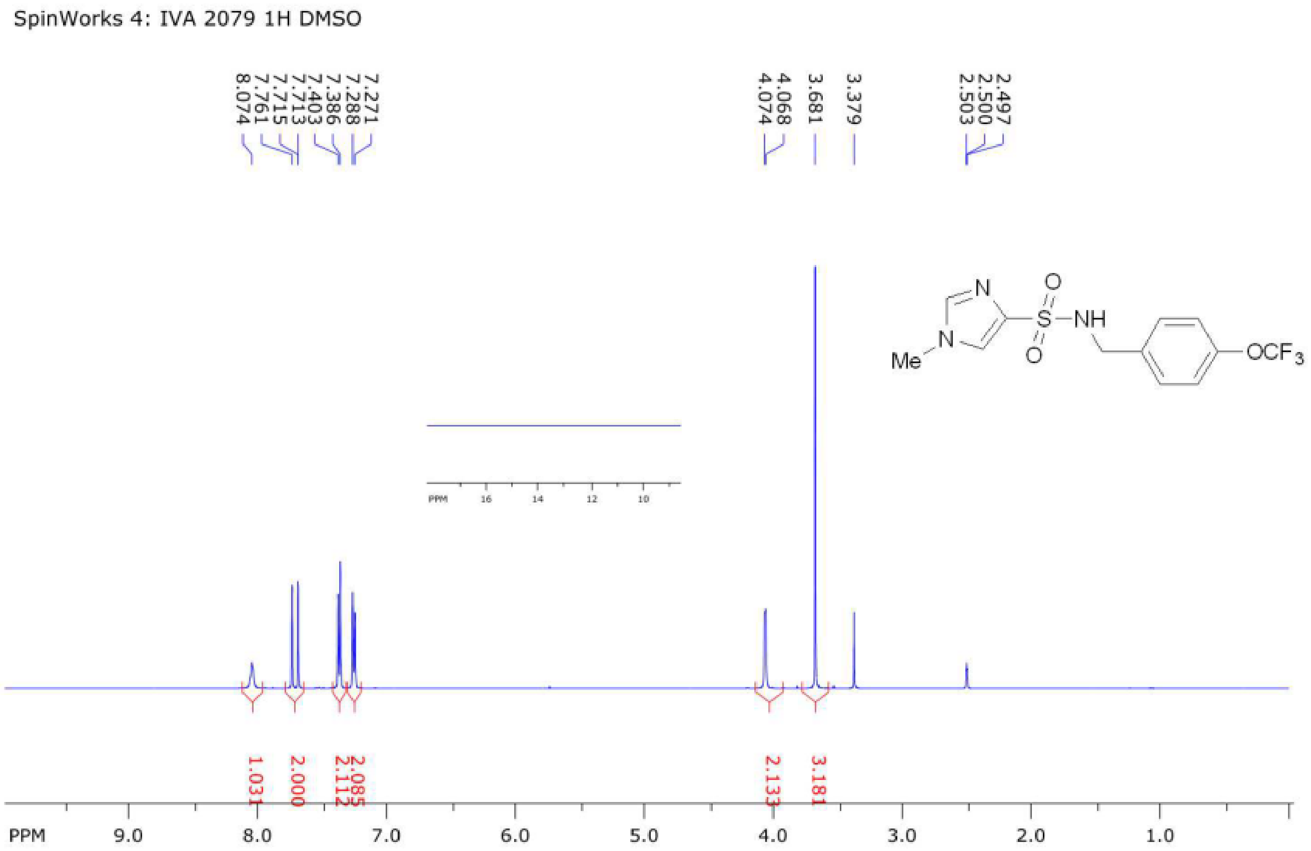

file: ...NAPO\NMR\500-2\mkr12207\5 2079\fid expt: <zg30>
transmitter freq.: 500.133001 MHz
time domain size: 65536 points
width: 12335.53 Hz = 24.6645 ppm = 0.188225 Hz/pt
number of scans: 24

freq. of 0 ppm: 500.130005 MHz
processed size: 65536 complex points
LB: 0.300 GF: 0.0000
Hz/cm: 200.316 ppm/cm: 0.40053

Compound **4ad**

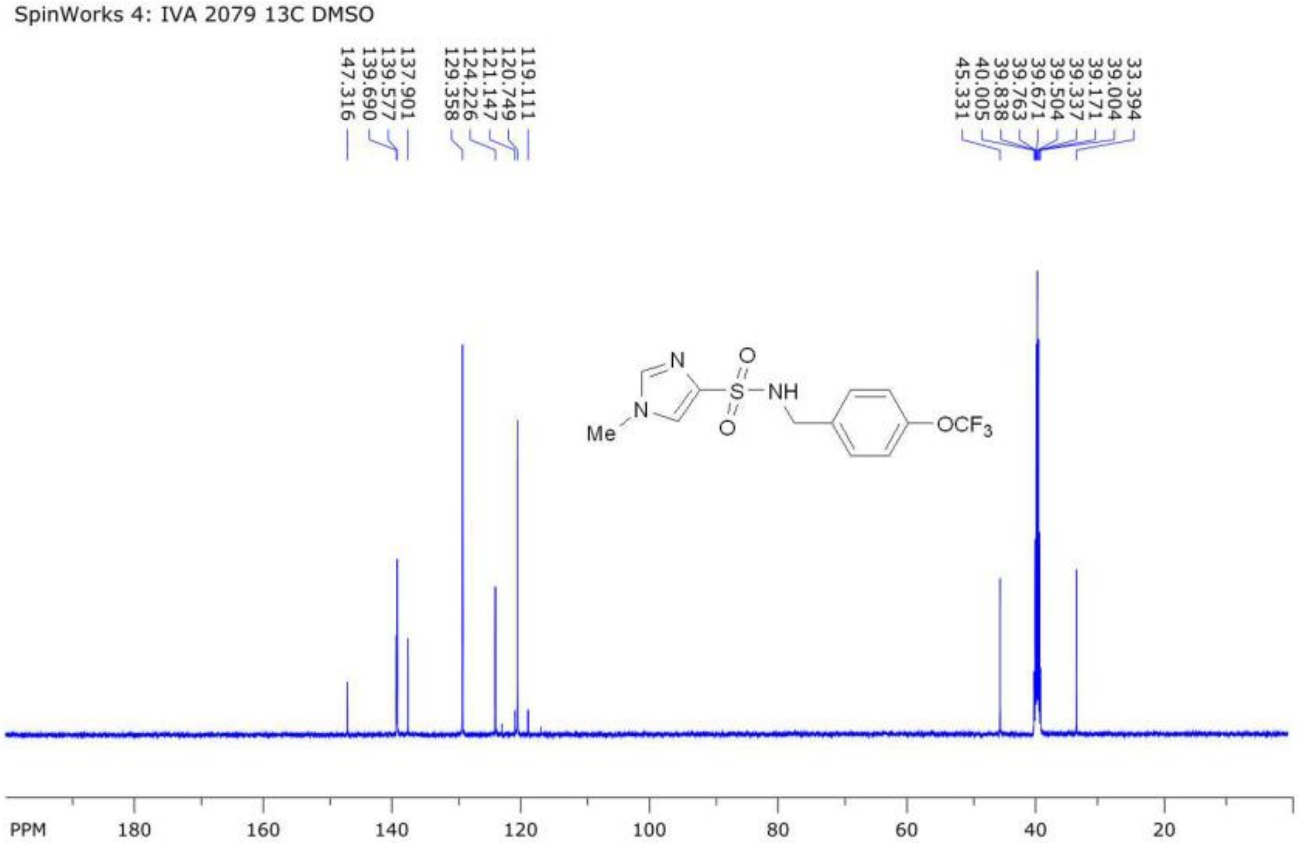

file: D:\NAPO\NMR\500-2\mkr12207\6\fid expt: <zgpg30>
transmitter freq.: 125.772879 MHz
time domain size: 65536 points
width: 36057.69 Hz = 286.6889 ppm = 0.550197 Hz/pt
number of scans: 512

freq. of 0 ppm: 125.757840 MHz
processed size: 32768 complex points
LB: 2.000 GF: 0.0000
Hz/cm: 1008.339 ppm/cm: 8.01714

Compound **4ae**

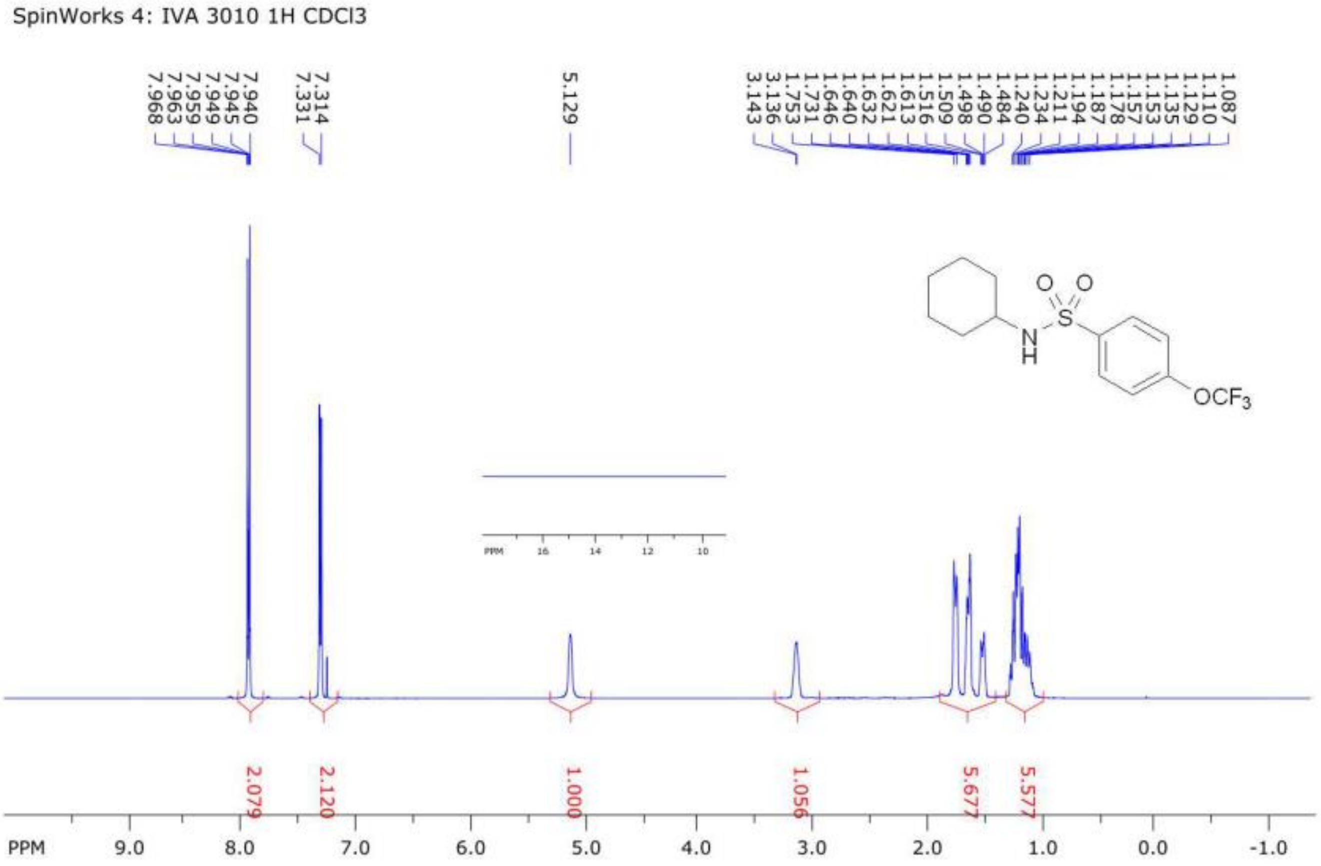

file: ...APO\NMR\500-2\mkr10307\11 3010\fid expt: <zg30>
transmitter freq.: 500.133001 MHz
time domain size: 65536 points
width: 12335.53 Hz = 24.6645 ppm = 0.188225 Hz/pt
number of scans: 24

freq. of 0 ppm: 500.130020 MHz
processed size: 65536 complex points
LB: 0.300 GF: 0.0000
Hz/cm: 229.790 ppm/cm: 0.45946

Compound **4ae**

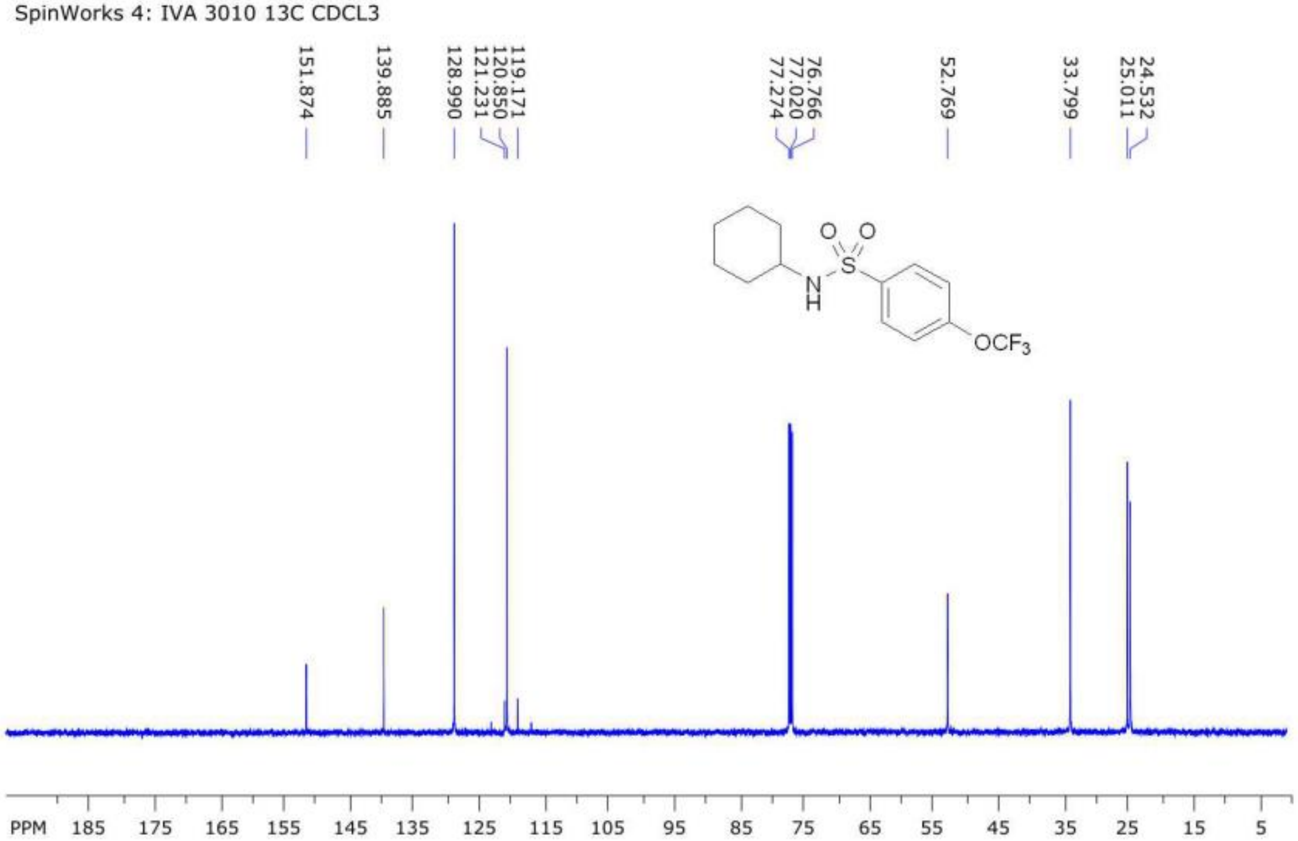

file: D:\NAPO\NMR\500-2\mkr10307\12\fid expt: <zgpg30>
transmitter freq.: 125.772879 MHz
time domain size: 65536 points
width: 36057.69 Hz = 286.6889 ppm = 0.550197 Hz/pt
number of scans: 512

freq. of 0 ppm: 125.757794 MHz
processed size: 32768 complex points
LB: 2.000 GF: 0.0000
Hz/cm: 995.575 ppm/cm: 7.91566

Compound **5**

IVA 1896
1H CDCl3

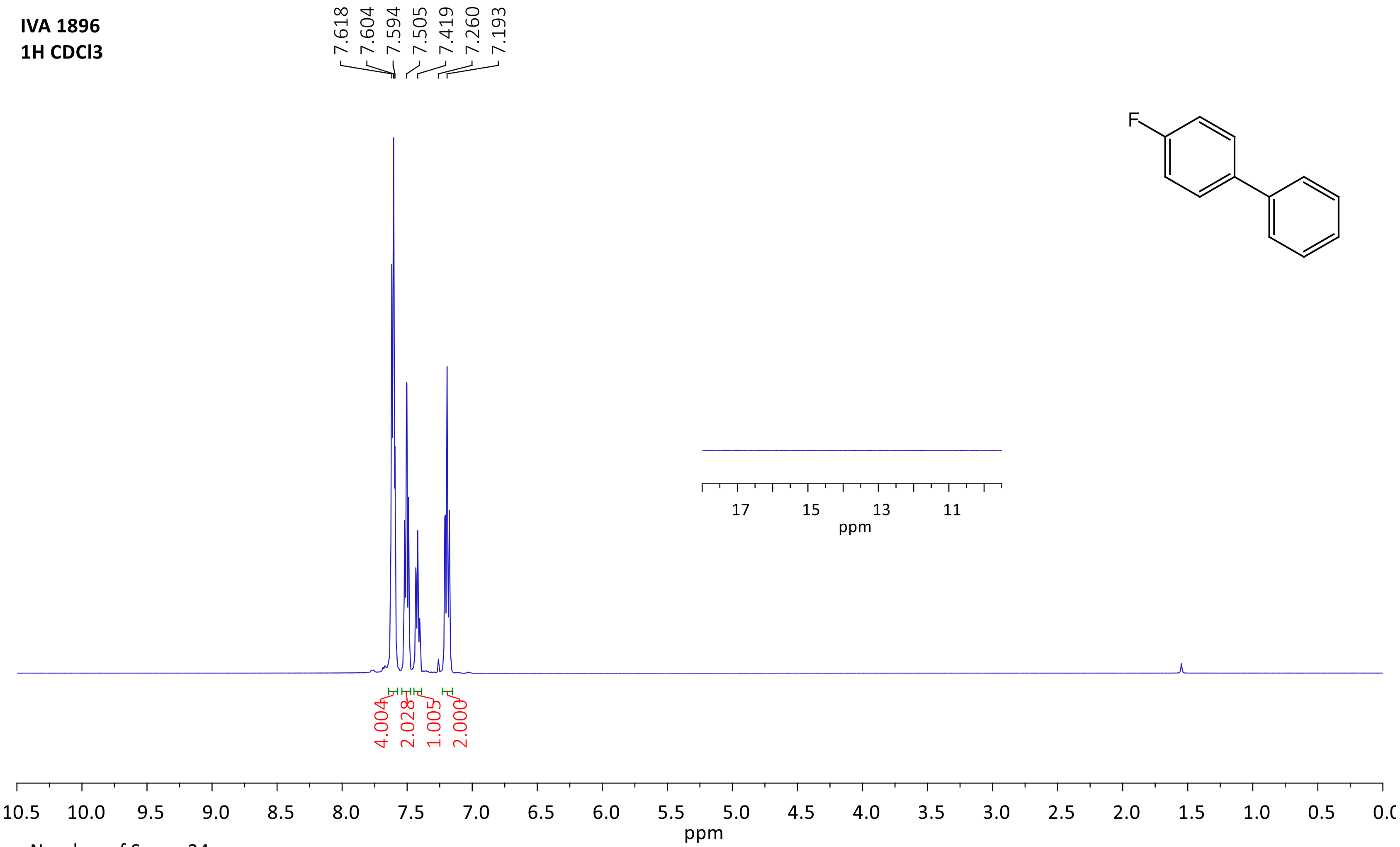

Number of Scans 24
Spectromet. Freq.500.13
Spectral Width 12335.5
Spectral Size 65536
freq. of 0 ppm: 500.1300236

Compound **5**

IVA 1896
13C CDCl3

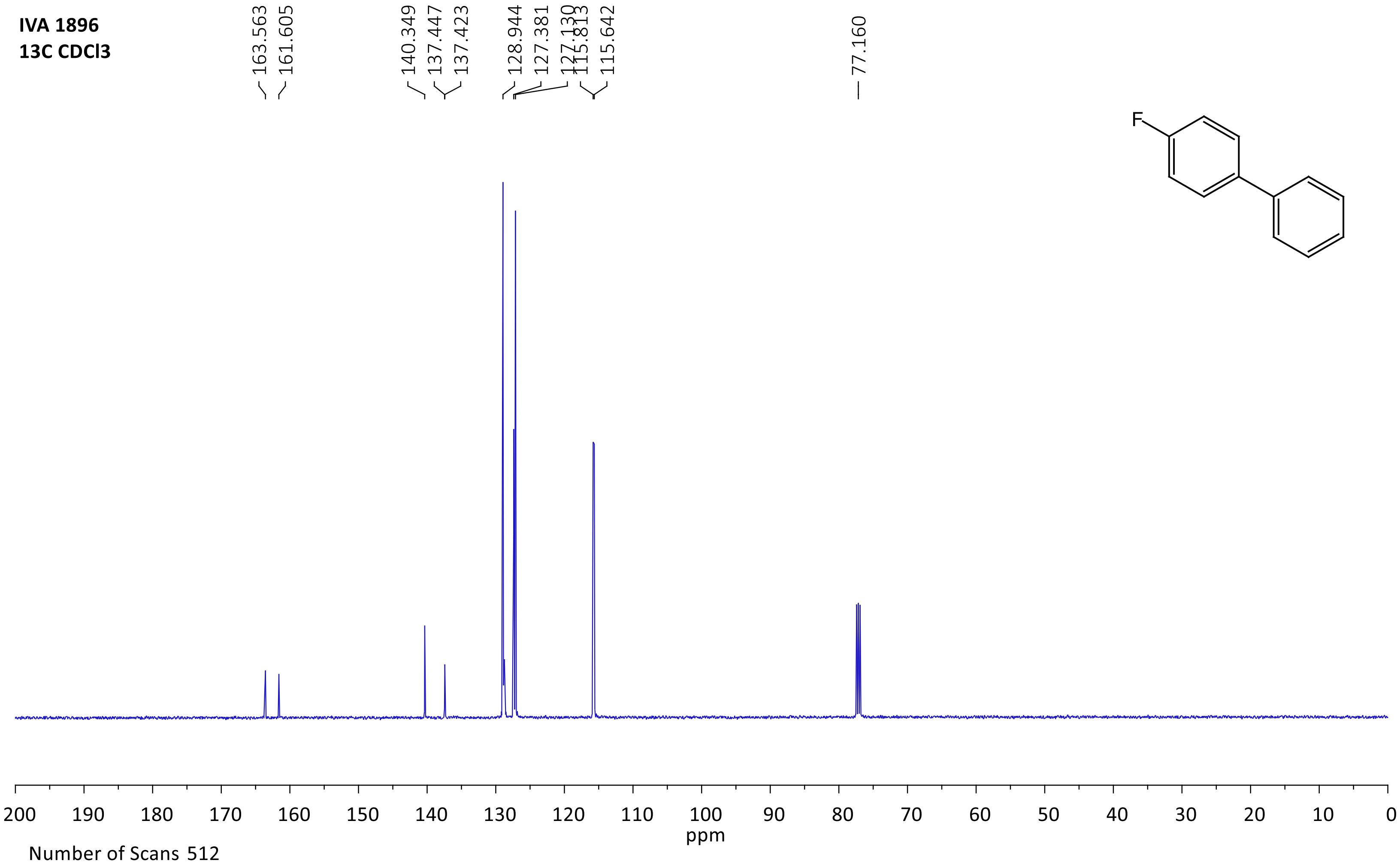

Number of Scans 512
Spectromet. Freq.125.76
Spectral Width 36057.7
Spectral Size 65536
freq. of 0 ppm: 125.7577890

(D) Mass spectra

Compound **4q**

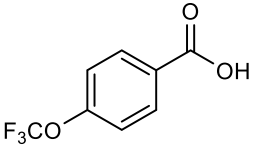

4-(trifluoromethoxy)benzoic acid **4q**

Chemical Formula: $C_8H_5F_3O_3$

Molecular Weight: 206.12

Sample Information

Sample Name	: Satenik_M
Sample ID	: IVA_2508
Analyzed	: 2019-06-28 09:57:23
Vial #	: 0
Injection Volume	: 0.50
$EndIf$Data File	: C:\GCMSsolution\Data\widma\2019\06_2019\Sa
Method File	: C:\GCMSsolution\System\Tune1\DI EI 1 metoda
Tuning File	: C:\GCMSsolution\System\Tune1\tuning12.06.20

Method

[Comment]

===== Analytical Line 1 =====

[GC-2010]

Column Oven Temp.	:30.0 °C
Injection Temp.	:150.00 °C
Injection Mode	:Split
Pressure	:12.8 kPa
Total Flow	:6.6 mL/min
Column Flow	:0.60 mL/min
Linear Velocity	:27.9 cm/sec
Purge Flow	:3.0 mL/min
Split Ratio	:5.0

[GCMS-QP2010 Ultra]

IonSourceTemp	:200.00 °C
Interface Temp.	:200.00 °C
Solvent Cut Time	:0.10 min
Detector Gain Mode	:Relative
Detector Gain	:1.24 kV +0.00 kV
Threshold	:0

[MS Table]

--Group 1 - Event 1--

Start Time	:0.10min
End Time	:25.00min
ACQ Mode	:Scan
Event Time	:0.10sec
Scan Speed	:20000
Start m/z	:35.00
End m/z	:1090.00

Chromatogram Satenik_M C:\GCMSsolution\Data\widma\2019\06_2019\Satenik_M_IVA_2508.QGD

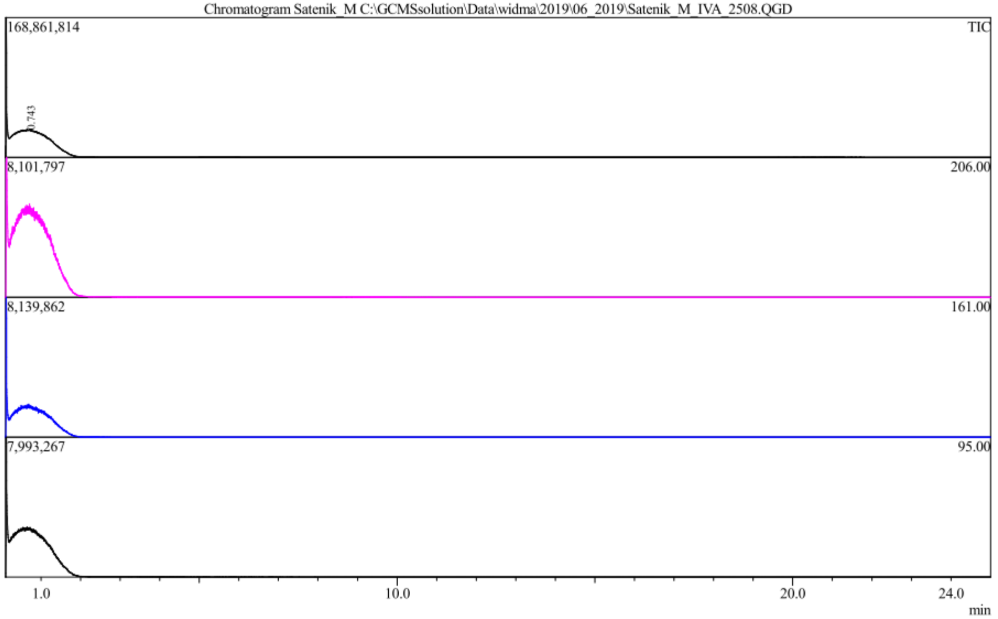

#	m/z	Abs. Int.	Rel. Int.
133	169.45	1406	0.02
134	170.90	897	0.01
135	171.90	405	0.01
136	172.90	62	0.00
137	173.90	285	0.00
138	174.90	195	0.00
139	175.95	989	0.01
140	176.95	5831	0.07
141	177.90	65563	0.84
142	178.85	12690	0.16
143	179.85	849	0.01
144	180.90	730	0.01
145	182.00	960	0.01
146	183.05	1274	0.02
147	183.95	910	0.01
148	184.95	1245	0.02
149	186.05	5287	0.07
150	187.05	3933	0.05
151	188.05	799937	10.25
152	188.95	7804431	100.00
153	189.95	1035742	13.27
154	191.95	4489	0.06
155	192.95	690	0.01
156	194.00	605	0.01
157	195.00	855	0.01
158	195.90	628	0.01
159	196.90	2434	0.03
160	197.90	610	0.01
161	198.90	740	0.01
162	200.90	145	0.00
163	201.90	482	0.01
164	203.05	1192	0.02
165	205.05	505696	6.48
166	205.95	5152459	66.02
167	206.95	704847	9.03
168	207.95	7752	0.10
169	208.95	3114	0.04
170	209.95	743	0.01
171	211.00	697	0.01
172	212.00	136	0.00
173	213.00	564	0.01
174	214.00	137	0.00
175	215.00	176	0.00
176	216.00	70	0.00
177	217.00	170	0.00
178	218.00	200	0.00
179	219.00	241	0.00
180	220.00	759	0.01
181	221.00	445	0.01
182	222.00	614	0.01
183	223.00	649	0.01
184	224.00	479	0.01
185	225.00	851	0.01
186	226.00	418	0.01
187	227.00	651	0.01
188	228.00	236	0.00
189	229.00	513	0.01
190	230.00	13	0.00
191	231.00	92	0.00
192	232.00	330	0.00
193	233.00	509	0.01
194	234.00	507	0.01
195	235.00	204	0.00
196	236.00	62	0.00
197	237.00	198	0.00
198	238.00	403	0.01
199	239.00	564	0.01
200	240.00	14	0.00
201	241.00	244	0.00
202	242.00	283	0.00
203	243.00	69	0.00
204	244.00	402	0.01
205	245.00	346	0.00
206	246.00	139	0.00
207	247.00	412	0.01
208	248.00	628	0.01
209	249.00	344	0.00
210	250.00	553	0.01
211	251.00	212	0.00
212	252.00	522	0.01
213	253.00	301	0.00
214	254.00	412	0.01
215	255.00	176	0.00
216	256.00	517	0.01
217	257.00	44	0.00
218	258.00	184	0.00

#	m/z	Abs. Int.	Rel. Int.
219	261.00	62	0.00
220	262.00	266	0.00
221	263.00	762	0.01
222	264.00	846	0.01
223	265.00	201	0.00
224	266.00	1095	0.01
225	267.00	409	0.01
226	268.00	650	0.01
227	269.00	166	0.00
228	270.00	226	0.00
229	271.00	150	0.00
230	273.00	25	0.00
231	274.00	117	0.00
232	275.00	22	0.00
233	276.00	301	0.00
234	277.00	180	0.00
235	278.00	434	0.01
236	279.00	551	0.01
237	280.00	501	0.01
238	281.00	696	0.01
239	282.00	55	0.00
240	283.00	512	0.01
241	284.00	450	0.01
242	285.00	700	0.01
243	286.00	62	0.00
244	287.00	637	0.01
245	288.00	259	0.00
246	289.00	696	0.01
247	290.00	117	0.00
248	291.00	333	0.00
249	292.00	5	0.00
250	293.00	361	0.00
251	295.00	338	0.00
252	296.00	287	0.00
253	298.00	6	0.00
254	302.00	168	0.00
255	303.00	116	0.00
256	304.00	264	0.00
257	305.00	507	0.01
258	306.00	266	0.00
259	307.00	277	0.00
260	309.00	405	0.01
261	310.00	131	0.00
262	311.00	181	0.00
263	312.00	248	0.00
264	313.00	227	0.00
265	315.00	112	0.00
266	316.00	4	0.00
267	317.00	263	0.00
268	318.00	143	0.00
269	320.00	65	0.00
270	321.00	2	0.00
271	322.00	312	0.00
272	323.00	94	0.00
273	324.00	303	0.00
274	325.00	119	0.00
275	326.00	124	0.00
276	327.00	393	0.01
277	328.00	261	0.00
278	329.00	176	0.00
279	330.00	437	0.01
280	331.00	127	0.00
281	332.00	106	0.00
282	333.00	414	0.01
283	334.00	122	0.00
284	335.00	162	0.00
285	337.00	59	0.00
286	338.00	308	0.00
287	340.00	203	0.00
288	341.00	93	0.00
289	342.00	79	0.00
290	343.00	340	0.00
291	344.00	533	0.01
292	346.00	327	0.00
293	347.00	183	0.00
294	348.00	374	0.00
295	349.00	226	0.00
296	350.00	68	0.00
297	351.00	198	0.00
298	352.00	181	0.00
299	353.00	155	0.00
300	357.00	55	0.00
301	362.00	137	0.00
302	363.00	32	0.00
303	364.00	305	0.00
304	365.00	264	0.00

#	m/z	Abs. Int.	Rel. Int.
305	366.00	126	0.00
306	367.00	235	0.00
307	368.00	92	0.00
308	369.00	580	0.01
309	370.00	209	0.00
310	371.00	191	0.00
311	372.00	345	0.00
312	373.00	282	0.00
313	381.00	23	0.00
314	383.00	233	0.00
315	384.00	139	0.00
316	385.00	143	0.00
317	386.00	240	0.00
318	387.00	108	0.00
319	388.00	226	0.00
320	389.00	123	0.00
321	390.00	72	0.00
322	391.00	108	0.00
323	392.00	215	0.00
324	402.00	550	0.01
325	403.00	27	0.00
326	404.00	62	0.00
327	405.00	39	0.00
328	406.00	674	0.01
329	407.00	21	0.00
330	408.00	200	0.00
331	409.00	108	0.00
332	410.00	72	0.00
333	411.00	17	0.00
334	413.00	121	0.00
335	420.00	38	0.00
336	422.00	12	0.00
337	423.00	111	0.00
338	424.00	158	0.00
339	426.00	196	0.00
340	427.00	275	0.00
341	428.00	132	0.00
342	429.00	34	0.00
343	430.00	188	0.00
344	431.00	229	0.00
345	432.00	167	0.00
346	433.00	100	0.00
347	435.00	23	0.00
348	441.00	18	0.00
349	444.00	147	0.00
350	445.00	167	0.00
351	446.00	39	0.00
352	447.00	151	0.00
353	448.00	42	0.00
354	449.00	135	0.00
355	450.00	72	0.00
356	451.00	21	0.00
357	452.00	47	0.00
358	453.00	83	0.00
359	456.00	19	0.00
360	458.00	11	0.00
361	462.00	15	0.00
362	464.00	100	0.00
363	465.00	53	0.00
364	466.00	154	0.00
365	467.00	92	0.00
366	468.00	169	0.00
367	469.00	85	0.00
368	471.00	93	0.00
369	472.00	99	0.00
370	474.00	101	0.00
371	478.00	29	0.00
372	479.00	149	0.00
373	484.00	7	0.00
374	485.00	192	0.00
375	486.00	68	0.00
376	487.00	187	0.00
377	488.00	177	0.00
378	489.00	16	0.00
379	490.00	36	0.00
380	491.00	113	0.00
381	493.00	107	0.00
382	502.00	52	0.00
383	503.00	75	0.00
384	505.00	225	0.00
385	506.00	208	0.00
386	507.00	82	0.00
387	508.00	213	0.00
388	509.00	85	0.00
389	510.00	154	0.00
390	512.00	48	0.00

Sample Information

Sample Name	: M.Satenik
Sample ID	: IVA_2638
Analyzed	: 2019-07-01 17:39:56
Vial #	: 3
Injection Volume	: 0.30
$EndIf$Data File	: C:\GCMSsolution\Data\widma\2019\07_2019\IV
Method File	: C:\GCMSsolution\Data\widma\metody\ewelina 0
Tuning File	: C:\GCMSsolution\System\Tune1\tuning12.06.20

Configuration Control

<<Column>>
Name : ZB5MSi
Serial # :
Thickness : 0.25um
Length : 30.0m
Inside Diameter : 0.25mm
Max Usable Temp : 360°C
Installation Date : 2018/06/05
Description :

Method

[Comment]

===== Analytical Line 1 =====

[GC-2010]

Column Oven Temp.	:50.0 °C
Injection Temp.	:250.00 °C
Injection Mode	:Splitless
Sampling Time	:1.00 min
Pressure	:70.0 kPa
Total Flow	:63.5 mL/min
Column Flow	:1.22 mL/min
Linear Velocity	:40.1 cm/sec
Purge Flow	:1.0 mL/min
Split Ratio	:50.0

Oven Temp. Program

Rate	Temperature(°C)	Hold Time(min)
-	50.0	3.00
30.00	150.0	5.00
25.00	250.0	25.00

[GCMS-QP2010 Ultra]

IonSourceTemp	:200.00 °C
Interface Temp.	:200.00 °C
Solvent Cut Time	:3.50 min
Detector Gain Mode	:Absolute
Detector Gain	:1.20 kV
Threshold	:0

[MS Table]
--Group 1 - Event 1--

Start Time	:3.50min
End Time	:40.33min
ACQ Mode	:Scan
Event Time	:0.10sec
Scan Speed	:20000
Start m/z	:35.00
End m/z	:1000.00

[Similarity Search Parameters]

File1(Min.SI:50)	:C:\GCMSsolution\library\NIST11.lib
File2(Min.SI:50)	:C:\GCMSsolution\library\NIST11s.lib
Search Depth	:No PreSearch
Max Hit#	:25
Delete the same Compounds	:OFF
Reverse Search	:OFF
--- Post-search ---	
Option (Match Case)	:OFF

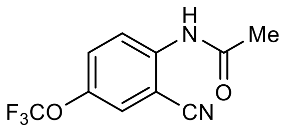

N-(2-cyano-4-(trifluoromethoxy)phenyl)acetamide **4r**

Chemical Formula: $C_{10}H_7F_3N_2O_2$
Molecular Weight: 244.17

Compound **4r**

Chromatogram M.Satenik C:\GCMSsolution\Data\widma\2019\07_2019\IVA_2638.qgd

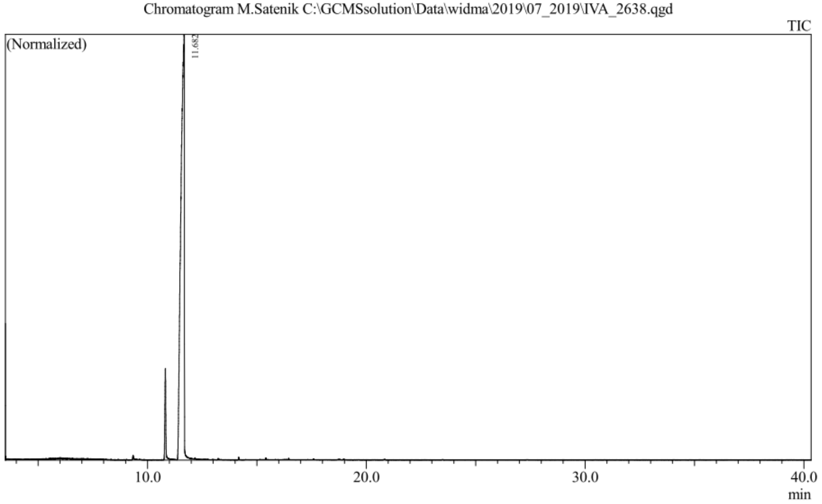

Spectrum

Line#:1 R.Time:11.682(Scan#:4910)
MassPeaks:563
RawMode:Single 11.682(4910) BasePeak:43(8228884)
BG Mode:None Group 1 - Event 1 Scan

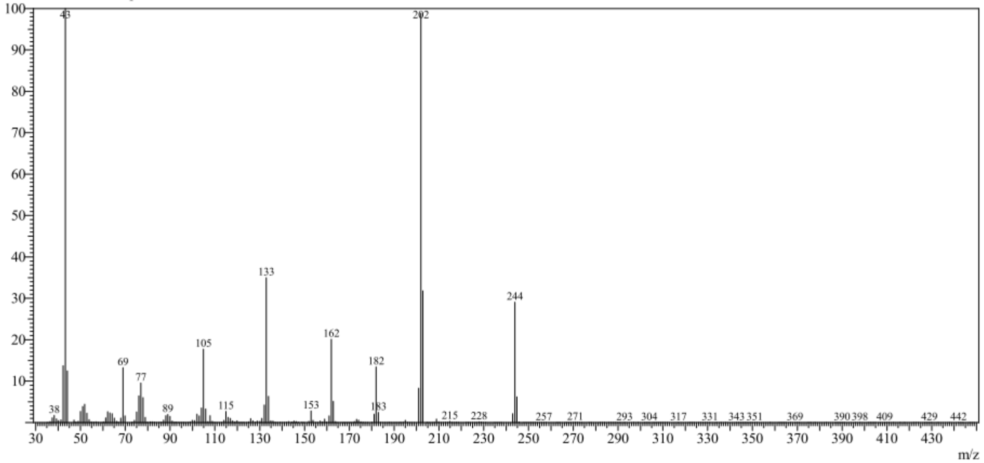

Mass Table
Line#:1 R.Time:11.682(Scan#:4910)
MassPeaks:563
RawMode:Single 11.682(4910) BasePeak:43(8228884)
BG Mode:None Group 1 - Event 1 Scan

#	m/z	Abs. Int.	Rel. Int.
1	35.35	1000	0.01
2	36.35	17416	0.21
3	37.35	95076	1.16
4	38.25	140065	1.70
5	39.25	75636	0.92
6	40.20	41355	0.50
7	41.25	55405	0.67
8	42.25	1129901	13.73
9	43.20	8228884	100.00
10	44.15	1025693	12.46
11	47.10	48351	0.59
12	48.05	9444	0.11
13	48.95	25623	0.31
14	49.95	219272	2.66
15	50.95	319976	3.89
16	51.90	361771	4.40
17	52.85	185167	2.25
18	53.85	56558	0.69
19	54.85	12486	0.15
20	55.85	4031	0.05
21	56.15	4352	0.05
22	57.05	14185	0.17
23	58.05	3899	0.05
24	59.25	450	0.01
25	60.25	15016	0.18
26	61.25	91607	1.11
27	62.15	215724	2.62
28	63.15	192054	2.33
29	64.15	175364	2.13
30	65.15	89170	1.08
31	66.15	24272	0.29
32	66.90	7913	0.10
33	67.95	90070	1.09
34	68.95	1091324	13.26
35	69.85	130463	1.59
36	73.05	11447	0.14
37	74.05	46398	0.56
38	75.05	212742	2.59
39	76.05	532394	6.47
40	76.95	783987	9.53
41	77.95	497889	6.05
42	78.95	100780	1.22
43	79.95	10811	0.13
44	81.10	13363	0.16
45	82.05	12466	0.15

#	m/z	Abs. Int.	Rel. Int.
46	83.05	11451	0.14
47	84.05	1833	0.02
48	85.05	2503	0.03
49	86.05	10744	0.13
50	87.05	48016	0.58
51	88.05	139257	1.69
52	88.95	163861	1.99
53	89.95	122548	1.49
54	90.95	27111	0.33
55	91.95	8183	0.10
56	92.85	10893	0.13
57	93.85	3616	0.04
58	94.95	2829	0.03
59	95.90	5318	0.06
60	96.85	1032	0.01
61	97.95	2516	0.03
62	98.95	16380	0.20
63	99.95	43183	0.52
64	100.95	40801	0.50
65	101.95	169400	2.06
66	102.95	134620	1.64
67	103.95	288047	3.50
68	104.90	1460361	17.75
69	105.85	268323	3.26
70	106.90	21747	0.26
71	107.90	137304	1.67
72	108.85	24356	0.30
73	109.85	4973	0.06
74	110.85	1449	0.02
75	111.85	994	0.01
76	113.05	3544	0.04
77	114.05	45721	0.56
78	114.95	221681	2.69
79	115.95	102748	1.25
80	116.95	83342	1.01
81	117.95	34126	0.41
82	118.90	14827	0.18
83	119.90	33823	0.41
84	120.85	7175	0.09
85	121.85	4571	0.06
86	122.85	2924	0.04
87	124.05	55	0.00
88	125.05	12737	0.15
89	126.00	76369	0.93
90	126.95	34445	0.42

#	m/z	Abs. Int.	Rel. Int.
91	128.05	12130	0.15
92	128.95	31168	0.38
93	130.05	24484	0.30
94	130.95	83186	1.01
95	132.05	350651	4.26
96	132.95	2879176	34.99
97	133.95	521936	6.34
98	134.95	35096	0.43
99	135.95	29245	0.36
100	136.95	4965	0.06
101	137.95	1624	0.02
102	139.15	2996	0.04
103	140.05	1753	0.02
104	140.95	1169	0.01
105	141.95	4552	0.06
106	142.95	17955	0.22
107	144.35	10446	0.13
108	145.30	29537	0.36
109	146.25	21081	0.26
110	147.25	6448	0.08
111	148.85	6739	0.08
112	149.75	1597	0.02
113	151.95	22598	0.27
114	152.95	228735	2.78
115	153.85	46394	0.56
116	154.85	184	0.00
117	155.95	2032	0.02
118	156.95	28120	0.34
119	157.95	16768	0.20
120	158.95	68097	0.83
121	159.85	10093	0.12
122	160.95	134672	1.64
123	161.95	1655886	20.12
124	162.85	423188	5.14
125	163.85	19271	0.23
126	164.85	627	0.01
127	166.40	1365	0.02
128	167.45	1051	0.01
129	168.40	752	0.01
130	169.40	468	0.01
131	170.40	484	0.01
132	171.25	241	0.00
133	172.25	17966	0.22
134	173.20	61555	0.75
135	174.15	48312	0.59

#	m/z	Abs. Int.	Rel. Int.
136	175.15	14710	0.18
137	176.15	5111	0.06
138	177.15	1840	0.02
139	178.20	418	0.01
140	180.05	3885	0.05
141	181.05	168808	2.05
142	181.95	1106350	13.44
143	182.95	199604	2.43
144	183.95	2820	0.03
145	184.95	167	0.00
146	185.95	1314	0.02
147	187.05	1399	0.02
148	187.95	3261	0.04
149	188.95	1011	0.01
150	190.00	883	0.01
151	191.00	473	0.01
152	192.00	378	0.00
153	192.95	3874	0.05
154	194.05	7001	0.09
155	195.00	44388	0.54
156	195.95	7325	0.09
157	197.00	46	0.00
158	197.90	600	0.01
159	198.95	209	0.00
160	200.95	685296	8.33
161	201.95	8137220	98.89
162	202.85	2618595	31.82
163	204.85	4226	0.05
164	205.85	51	0.00
165	206.90	31	0.00
166	207.95	5533	0.07
167	208.90	69948	0.85
168	209.85	12831	0.16
169	210.85	778	0.01
170	211.90	319	0.00
171	212.90	3775	0.05
172	213.95	3881	0.05
173	214.85	26579	0.32
174	215.85	6363	0.08
175	216.85	1085	0.01
176	217.85	2532	0.03
177	218.75	805	0.01
178	221.90	347	0.00
179	222.90	2561	0.03
180	223.85	1158	0.01
181	224.90	699	0.01
182	226.10	758	0.01
183	226.95	1606	0.02
184	227.90	15922	0.19
185	228.85	8687	0.11
186	229.85	932	0.01
187	230.90	497	0.01
188	234.90	3	0.00
189	235.90	49	0.00
190	236.90	109	0.00
191	237.90	193	0.00
192	242.95	174457	2.12
193	243.90	2391358	29.06
194	244.85	511904	6.22
195	245.85	11238	0.14
196	247.90	387	0.00
197	248.90	704	0.01
198	249.90	113	0.00
199	250.90	216	0.00
200	251.90	13	0.00
201	252.90	129	0.00
202	253.90	65	0.00
203	254.90	298	0.00
204	255.90	389	0.00
205	256.80	1146	0.01
206	257.80	502	0.01
207	262.80	510	0.01
208	263.80	113	0.00
209	266.80	138	0.00
210	267.80	197	0.00
211	268.80	379	0.00
212	269.95	823	0.01
213	270.90	3549	0.04
214	271.90	514	0.01
215	272.90	148	0.00
216	273.90	1	0.00
217	276.90	110	0.00
218	277.90	403	0.00
219	281.90	504	0.01
220	282.90	718	0.01
221	283.90	106	0.00

#	m/z	Abs. Int.	Rel. Int.
222	285.90	67	0.00
223	286.90	45	0.00
224	287.90	227	0.00
225	288.90	361	0.00
226	289.90	97	0.00
227	290.90	37	0.00
228	292.90	723	0.01
229	296.90	339	0.00
230	298.90	2	0.00
231	301.90	107	0.00
232	302.90	353	0.00
233	303.90	377	0.00
234	305.90	321	0.00
235	306.90	155	0.00
236	307.90	139	0.00
237	308.90	258	0.00
238	309.90	124	0.00
239	310.90	137	0.00
240	311.90	132	0.00
241	316.90	274	0.00
242	321.90	78	0.00
243	322.90	306	0.00
244	323.90	26	0.00
245	325.90	124	0.00
246	326.90	115	0.00
247	327.90	87	0.00
248	328.90	224	0.00
249	329.90	176	0.00
250	330.90	332	0.00
251	333.90	13	0.00
252	335.90	264	0.00
253	337.90	158	0.00
254	342.90	282	0.00
255	343.90	65	0.00
256	344.90	110	0.00
257	345.90	84	0.00
258	346.90	169	0.00
259	347.90	35	0.00
260	348.90	208	0.00
261	349.90	152	0.00
262	350.90	307	0.00
263	351.90	22	0.00
264	357.90	51	0.00
265	361.90	5	0.00
266	362.90	238	0.00
267	363.90	24	0.00
268	364.90	99	0.00
269	366.90	138	0.00
270	368.90	370	0.00
271	369.90	253	0.00
272	370.90	122	0.00
273	374.90	8	0.00
274	375.90	21	0.00
275	377.90	113	0.00
276	382.90	173	0.00
277	386.90	115	0.00
278	387.90	27	0.00
279	388.90	312	0.00
280	389.90	385	0.00
281	390.90	199	0.00
282	395.90	9	0.00
283	397.90	174	0.00
284	401.90	166	0.00
285	402.90	156	0.00
286	403.90	134	0.00
287	404.90	49	0.00
288	406.90	158	0.00
289	408.90	426	0.01
290	409.90	161	0.00
291	410.90	38	0.00
292	412.90	72	0.00
293	415.90	58	0.00
294	417.90	116	0.00
295	421.90	180	0.00
296	422.90	292	0.00
297	423.90	172	0.00
298	426.90	23	0.00
299	427.90	202	0.00
300	428.90	483	0.01
301	429.90	188	0.00
302	432.90	75	0.00
303	437.90	125	0.00
304	441.90	448	0.01
305	442.90	95	0.00
306	443.90	193	0.00
307	446.90	125	0.00

#	m/z	Abs. Int.	Rel. Int.
308	447.90	439	0.01
309	448.90	345	0.00
310	450.90	30	0.00
311	451.90	186	0.00
312	456.90	62	0.00
313	457.90	127	0.00
314	460.90	11	0.00
315	461.90	384	0.00
316	462.90	137	0.00
317	466.90	203	0.00
318	467.90	370	0.00
319	468.90	205	0.00
320	469.90	31	0.00
321	470.90	110	0.00
322	472.90	57	0.00
323	474.90	32	0.00
324	476.90	200	0.00
325	481.90	285	0.00
326	482.90	156	0.00
327	486.90	335	0.00
328	487.90	229	0.00
329	488.90	232	0.00
330	489.90	112	0.00
331	490.90	44	0.00
332	491.90	34	0.00
333	492.90	139	0.00
334	494.90	44	0.00
335	495.90	200	0.00
336	496.90	14	0.00
337	500.90	98	0.00
338	501.90	404	0.00
339	506.90	177	0.00
340	507.90	313	0.00
341	508.90	120	0.00
342	509.90	129	0.00
343	510.90	113	0.00
344	511.90	63	0.00
345	512.90	34	0.00
346	514.90	50	0.00
347	515.90	38	0.00
348	516.90	98	0.00
349	520.90	25	0.00
350	521.90	300	0.00
351	522.90	18	0.00
352	525.90	73	0.00
353	526.90	22	0.00
354	527.90	125	0.00
355	528.90	334	0.00
356	529.90	123	0.00
357	530.90	37	0.00
358	532.90	117	0.00
359	535.90	27	0.00
360	536.90	48	0.00
361	540.90	87	0.00
362	541.90	184	0.00
363	545.90	213	0.00
364	546.90	41	0.00
365	547.90	110	0.00
366	548.90	57	0.00
367	549.90	207	0.00
368	550.90	86	0.00
369	554.90	98	0.00
370	555.90	107	0.00
371	556.90	50	0.00
372	561.90	354	0.00
373	562.90	3	0.00
374	564.90	54	0.00
375	566.90	3	0.00
376	567.90	68	0.00
377	568.90	349	0.00
378	569.90	242	0.00
379	570.90	104	0.00
380	572.90	50	0.00
381	574.90	17	0.00
382	581.90	269	0.00
383	582.90	89	0.00
384	583.90	74	0.00
385	585.90	53	0.00
386	587.90	250	0.00
387	588.90	295	0.00
388	589.90	337	0.00
389	592.90	85	0.00
390	596.90	29	0.00
391	601.90	365	0.00
392	602.90	54	0.00
393	606.90	197	0.00

Method

[Comment]

==== Analytical Line 1 ====

[GC-2010]

Column Oven Temp.	:30.0 °C
Injection Temp.	:150.00 °C
Injection Mode	:Split
Pressure	:12.8 kPa
Total Flow	:6.6 mL/min
Column Flow	:0.60 mL/min
Linear Velocity	:27.9 cm/sec
Purge Flow	:3.0 mL/min
Split Ratio	:5.0

[GCMS-QP2010 Ultra]

IonSourceTemp	:200.00 °C
Interface Temp.	:200.00 °C
Solvent Cut Time	:0.10 min
Detector Gain Mode	:Relative
Detector Gain	:1.35 kV +0.00 kV
Threshold	:0

[MS Table]

--Group 1 - Event 1--

Start Time	:0.10min
End Time	:25.00min
ACQ Mode	:Scan
Event Time	:0.10sec
Scan Speed	:20000
Start m/z	:35.00
End m/z	:1090.00

Sample Information

Sample Name	: M.Satenik
Sample ID	: IVA_2186
Analyzed	: 2019-05-09 14:44:41
Vial #	: 1
Injection Volume	: 0.10
$EndIf$Data File	: C:\GCMSsolution\Data\widma\2019\05_2019\M
Method File	: C:\GCMSsolution\System\Tune1\DI EI 1 metoda
Tuning File	: C:\GCMSsolution\System\Tune1\tuning06.05.20

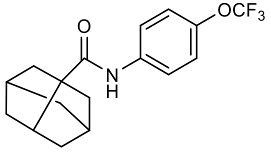

N-(4-(trifluoromethoxy)phenyl)adamantane-1-carboxamide **4t**

Chemical Formula: $C_{18}H_{20}F_{3}NO_{2}$

Molecular Weight: 339.36

Compound **4t**

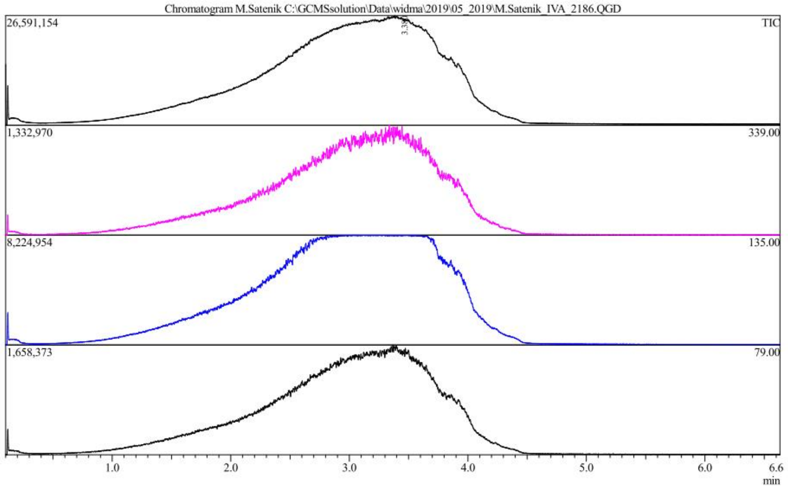

Spectrum

Line#:1 R.Time:3.380(Scan#:1969)
MassPeaks:592
RawMode:Single 3.380(1969) BasePeak:135(8204730)
BG Mode:None Group 1 - Event 1 Scan

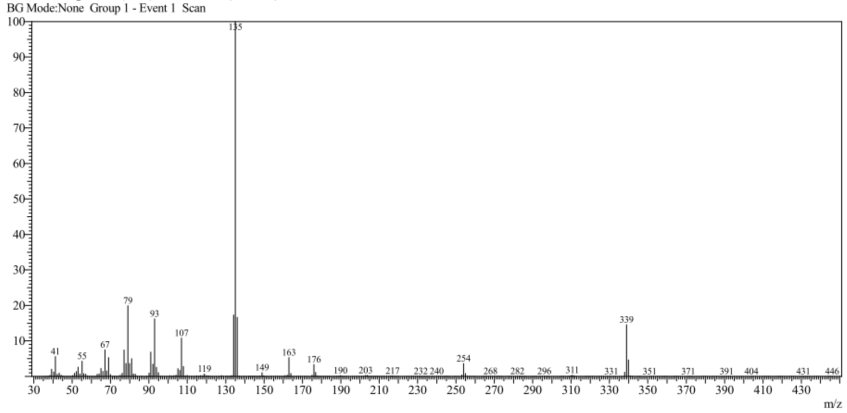

Mass Table
Line#:1 R.Time:3.380(Scan#:1969)
MassPeaks:592
RawMode:Single 3.380(1969) BasePeak:135(8204730)
BG Mode:None Group 1 - Event 1 Scan

#	m/z	Abs. Int.	Rel. Int.
1	35.00	280	0.00
2	36.30	1047	0.01
3	37.35	2592	0.03
4	38.35	25966	0.32
5	39.30	168164	2.05
6	40.35	102715	1.25
7	41.25	459894	5.61
8	42.25	51025	0.62
9	43.25	77971	0.95
10	44.25	27283	0.33
11	45.25	2900	0.04
12	46.70	2425	0.03
13	47.70	747	0.01
14	49.15	1435	0.02
15	50.15	19413	0.24
16	51.15	71166	0.87
17	52.15	111703	1.36
18	53.10	217103	2.65
19	54.10	60445	0.74
20	55.15	352755	4.30
21	56.05	65074	0.79
22	57.05	53557	0.65
23	58.05	15915	0.19
24	59.05	2282	0.03
25	60.05	2539	0.03
26	60.95	1658	0.02
27	62.05	7019	0.09
28	63.05	47210	0.58
29	64.05	58515	0.71
30	65.05	187026	2.28
31	66.05	116143	1.42
32	67.05	618538	7.54
33	67.95	125906	1.53
34	69.00	436318	5.32
35	69.95	44956	0.55
36	71.15	5313	0.06
37	72.15	3390	0.04
38	73.05	1825	0.02
39	74.05	5205	0.06
40	75.05	27755	0.34
41	76.05	75220	0.92
42	77.05	611115	7.45
43	78.05	298379	3.64
44	79.00	1635072	19.93
45	79.95	299665	3.65
46	81.00	410006	5.00
47	81.95	60091	0.73
48	82.95	54148	0.66
49	83.85	12089	0.15
50	85.05	4293	0.05
51	86.05	10094	0.12
52	86.95	2245	0.03
53	88.05	1907	0.02
54	89.05	11561	0.14
55	90.05	76559	0.93
56	91.00	564494	6.88
57	92.05	286119	3.49
58	93.00	1334790	16.27
59	93.95	212302	2.59
60	94.95	87377	1.06
61	95.95	11817	0.14
62	96.95	3456	0.04
63	98.05	1602	0.02
64	98.95	3928	0.05
65	100.05	5482	0.07
66	101.00	20847	0.25
67	102.00	8561	0.10
68	103.05	21013	0.26
69	104.05	30375	0.37
70	105.05	179724	2.19
71	106.05	139913	1.71
72	107.00	886867	10.81
73	107.95	230270	2.81
74	108.95	16807	0.20
75	110.00	22700	0.28
76	110.95	6220	0.08
77	111.95	3793	0.05
78	112.95	2530	0.03
79	114.05	4352	0.05
80	114.95	17111	0.21
81	116.05	10165	0.12
82	116.95	21672	0.26
83	118.05	17308	0.21
84	118.95	60533	0.74
85	119.95	21533	0.26
86	120.95	16140	0.20
87	121.95	5475	0.07
88	123.00	164	0.00
89	124.00	897	0.01
90	125.00	1253	0.02
91	125.95	1597	0.02
92	126.95	4801	0.06
93	127.90	19638	0.24
94	128.85	5141	0.06
95	130.05	8432	0.10
96	131.05	9815	0.12
97	132.15	12003	0.15
98	133.15	18527	0.23
99	134.15	1420945	17.32
100	135.05	8204730	100.00
101	136.05	1368002	16.67
102	137.05	9158	0.11
103	138.05	1620	0.02
104	139.25	1894	0.02
105	140.15	2961	0.04
106	141.15	2315	0.03
107	142.20	2985	0.04
108	143.15	6691	0.08
109	144.15	2611	0.03
110	144.95	3060	0.04
111	145.85	3136	0.04
112	146.95	3005	0.04
113	147.95	10028	0.12
114	148.90	86817	1.06
115	149.85	15067	0.18
116	150.85	1816	0.02
117	151.85	1256	0.02
118	153.05	2087	0.03
119	153.95	3140	0.04
120	155.05	2042	0.02
121	155.95	5906	0.07
122	156.95	2669	0.03
123	157.95	1888	0.02
124	159.00	379	0.00
125	160.05	3588	0.04
126	161.00	21948	0.27
127	162.05	28209	0.34
128	163.00	438351	5.34
129	163.95	70828	0.86
130	164.95	2437	0.03
131	165.95	1847	0.02
132	166.95	3365	0.04
133	167.90	3459	0.04
134	168.90	3135	0.04
135	169.85	2449	0.03
136	170.95	1856	0.02
137	171.85	1948	0.02
138	172.95	1401	0.02
139	174.95	28214	0.34
140	175.95	274598	3.35
141	176.85	92890	1.13
142	177.85	16874	0.21
143	178.85	220	0.00
144	179.50	1737	0.02
145	180.45	1104	0.01
146	181.40	529	0.01
147	182.40	1343	0.02
148	183.45	1083	0.01
149	184.35	1892	0.02
150	185.95	882	0.01
151	186.95	1416	0.02
152	187.85	7063	0.09
153	188.95	3569	0.04
154	189.85	18671	0.23
155	190.85	3160	0.04
156	191.90	446	0.01
157	193.10	1493	0.02
158	196.10	462	0.01
159	197.10	91	0.00
160	198.10	748	0.01
161	198.95	913	0.01
162	199.85	1372	0.02
163	200.90	2543	0.03
164	201.95	6914	0.08
165	202.95	25822	0.31
166	203.85	24058	0.29
167	204.85	5303	0.06
168	205.70	600	0.01
169	206.70	2277	0.03
170	207.70	804	0.01
171	208.70	69	0.00
172	209.70	373	0.00
173	210.80	626	0.01
174	211.85	4384	0.05
175	212.75	1949	0.02
176	213.85	5574	0.07
177	215.05	2426	0.03
178	216.05	8115	0.10
179	216.95	11939	0.15
180	217.95	4998	0.06
181	218.95	615	0.01
182	220.00	248	0.00
183	224.00	462	0.01
184	224.95	923	0.01
185	225.95	1889	0.02
186	226.90	3706	0.05
187	227.95	3615	0.04
188	228.85	1638	0.02
189	229.75	4601	0.06
190	230.75	2631	0.03
191	231.70	5458	0.07
192	232.65	1254	0.02
193	233.70	437	0.01
194	234.70	288	0.00
195	235.70	366	0.00
196	237.70	241	0.00
197	239.00	558	0.01
198	239.95	3670	0.04
199	240.95	2613	0.03
200	241.95	2686	0.03
201	243.55	1070	0.01
202	244.45	954	0.01
203	245.50	2957	0.04
204	246.50	455	0.01
205	249.50	174	0.00
206	251.05	1436	0.02
207	253.05	32312	0.39
208	253.95	297205	3.62
209	254.95	67232	0.82
210	255.95	5561	0.07
211	256.95	54	0.00
212	257.85	1510	0.02
213	258.80	2312	0.03
214	259.80	279	0.00
215	264.05	971	0.01
216	264.95	1035	0.01
217	265.95	2739	0.03
218	267.15	1601	0.02
219	268.05	6139	0.07
220	269.05	2021	0.02
221	270.05	2806	0.03
222	270.95	784	0.01
223	272.00	2994	0.04
224	273.00	489	0.01
225	274.00	141	0.00
226	277.00	82	0.00
227	278.00	410	0.00
228	279.00	903	0.01
229	280.10	1329	0.02
230	281.00	310	0.00
231	282.05	5006	0.06
232	282.95	953	0.01
233	283.55	1562	0.02
234	284.60	469	0.01
235	285.60	646	0.01
236	286.60	315	0.00
237	289.60	448	0.01
238	290.60	611	0.01
239	291.80	1281	0.02
240	293.75	1332	0.02
241	295.10	419	0.01
242	296.05	3137	0.04
243	297.30	1009	0.01
244	298.30	460	0.01
245	304.30	567	0.01
246	305.30	876	0.01
247	306.30	718	0.01
248	308.75	1095	0.01
249	309.75	14768	0.18
250	310.65	27750	0.34
251	311.65	13155	0.16
252	312.65	1052	0.01
253	317.70	7	0.00
254	319.00	522	0.01
255	319.95	2026	0.02
256	321.00	538	0.01
257	324.00	17	0.00
258	325.00	454	0.01
259	326.00	608	0.01
260	327.00	87	0.00
261	329.00	86	0.00
262	330.00	249	0.00
263	331.00	834	0.01
264	332.00	515	0.01
265	333.00	165	0.00
266	336.00	408	0.00
267	337.95	97312	1.19
268	338.95	1194509	14.56
269	339.85	380831	4.64
270	340.85	16822	0.21
271	341.85	472	0.01
272	344.90	595	0.01
273	345.90	494	0.01
274	350.90	1024	0.01
275	351.90	259	0.00
276	352.90	322	0.00
277	354.90	85	0.00
278	355.90	414	0.01
279	357.90	126	0.00
280	358.90	257	0.00
281	359.90	124	0.00
282	363.90	156	0.00
283	364.90	284	0.00
284	365.90	746	0.01
285	371.00	1126	0.01
286	372.00	221	0.00
287	373.00	345	0.00
288	379.00	427	0.01
289	380.00	340	0.00
290	384.00	596	0.01
291	386.00	662	0.01
292	389.00	231	0.00
293	390.00	82	0.00
294	391.00	817	0.01
295	392.00	369	0.00
296	393.00	187	0.00
297	399.00	420	0.01
298	404.00	943	0.01
299	405.00	9	0.00
300	406.00	865	0.01
301	410.00	52	0.00
302	411.00	648	0.01
303	412.00	507	0.01
304	413.00	360	0.00
305	418.00	178	0.00
306	419.00	391	0.00
307	420.00	137	0.00
308	424.00	243	0.00
309	425.00	386	0.00
310	426.00	512	0.01
311	430.00	135	0.00
312	431.00	824	0.01
313	432.00	331	0.00
314	433.00	195	0.00
315	434.00	186	0.00
316	437.00	9	0.00
317	438.00	135	0.00
318	439.00	139	0.00
319	444.00	466	0.01
320	445.00	214	0.00
321	446.00	630	0.01
322	450.00	366	0.00
323	451.00	733	0.01
324	452.00	194	0.00
325	453.00	294	0.00
326	458.00	314	0.00
327	459.00	156	0.00
328	464.00	188	0.00
329	465.00	226	0.00
330	466.00	648	0.01
331	470.00	225	0.00
332	471.00	730	0.01
333	472.00	112	0.00
334	473.00	352	0.00
335	478.00	299	0.00
336	479.00	50	0.00
337	480.00	29	0.00
338	484.00	407	0.00
339	485.00	23	0.00
340	486.00	585	0.01
341	490.00	394	0.00
342	491.00	655	0.01
343	492.00	212	0.00
344	493.00	138	0.00
345	497.00	16	0.00
346	498.00	352	0.00
347	504.00	437	0.01
348	505.00	49	0.00
349	506.00	485	0.01
350	510.00	214	0.00
351	511.00	804	0.01
352	512.00	43	0.00
353	518.00	293	0.00
354	519.00	156	0.00
355	524.00	365	0.00
356	525.00	280	0.00
357	526.00	438	0.01
358	530.00	406	0.00
359	531.00	850	0.01
360	532.00	163	0.00
361	538.00	7	0.00
362	539.00	39	0.00
363	544.00	361	0.00
364	545.00	186	0.00
365	546.00	435	0.01
366	550.00	611	0.01
367	551.00	505	0.01
368	552.00	352	0.00
369	554.00	6	0.00
370	557.00	27	0.00
371	559.00	222	0.00
372	564.00	575	0.01
373	565.00	353	0.00
374	566.00	162	0.00
375	570.00	735	0.01
376	571.00	268	0.00
377	572.00	443	0.01
378	575.00	33	0.00
379	577.00	195	0.00
380	579.00	319	0.00
381	584.00	226	0.00
382	585.00	494	0.01
383	586.00	261	0.00
384	590.00	776	0.01
385	591.00	300	0.00
386	592.00	274	0.00
387	593.00	78	0.00
388	597.00	137	0.00
389	598.00	105	0.00
390	599.00	120	0.00

Sample Information

Sample Name	: M.Satenik
Sample ID	: IVA_1419
Analyzed	: 2019-01-17 09:42:58
Vial #	: 12
Injection Volume	: 0.10
$EndIf$Data File	: C:\GCMSsolution\Data\widma\2019\01_2019\M
Method File	: C:\GCMSsolution\System\Tune1\DI EI 1 metoda
Tuning File	: C:\GCMSsolution\System\Tune1\tuning 07.01.20

Method

[Comment]

===== Analytical Line 1 =====

[GC-2010]

Column Oven Temp.	:30.0 °C
Injection Temp.	:150.00 °C
Injection Mode	:Split
Pressure	:12.8 kPa
Total Flow	:6.6 mL/min
Column Flow	:0.60 mL/min
Linear Velocity	:27.9 cm/sec
Purge Flow	:3.0 mL/min
Split Ratio	:5.0

[GCMS-QP2010 Ultra]

IonSourceTemp	:200.00 °C
Interface Temp.	:200.00 °C
Solvent Cut Time	:0.30 min
Detector Gain Mode	:Relative
Detector Gain	:1.29 kV +0.00 kV
Threshold	:0

[MS Table]
--Group 1 - Event 1--

Start Time	:0.30min
End Time	:25.00min
ACQ Mode	:Scan
Event Time	:0.10sec
Scan Speed	:20000
Start m/z	:35.00
End m/z	:1090.00

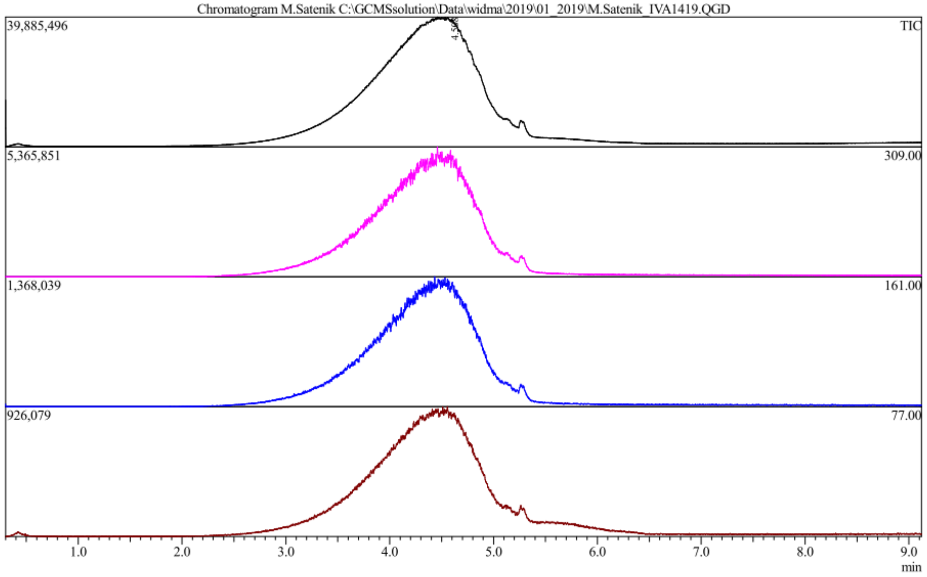

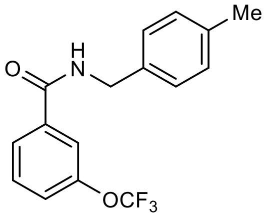

N-(4-methylbenzyl)-3-(trifluoromethoxy)benzamide **4u**
Chemical Formula: $C_{16}H_{14}F_3NO_2$
Molecular Weight: 309.29

Compound **4u**

Spectrum

Line#:1 R.Time:4.508(Scan#:2526)
MassPeaks:666
RawMode:Single 4.508(2526) BasePeak:189(7894720)
BG Mode:None Group 1 - Event 1 Scan

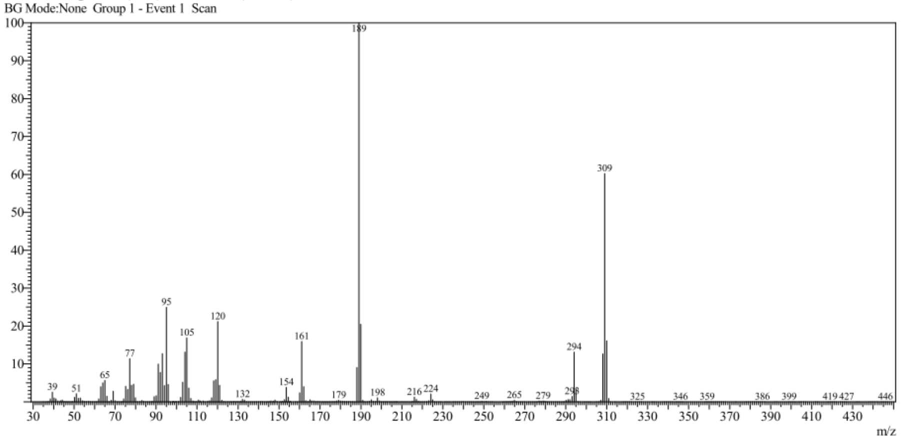

Mass Table
Line#:1 R.Time:4.508(Scan#:2526)
MassPeaks:666
RawMode:Single 4.508(2526) BasePeak:189(7894720)
BG Mode:None Group 1 - Event 1 Scan

#	m/z	Abs. Int.	Rel. Int.
1	35.00	437	0.01
2	36.35	1227	0.02
3	37.35	11095	0.14
4	38.35	61099	0.77
5	39.35	203024	2.57
6	40.25	78944	1.00
7	41.20	61245	0.78
8	42.15	17177	0.22
9	43.35	21533	0.27
10	44.25	39087	0.50
11	45.25	7193	0.09
12	46.30	3600	0.05
13	47.25	3262	0.04
14	48.10	1269	0.02
15	49.15	7732	0.10
16	50.15	94934	1.20
17	51.10	168838	2.14
18	52.05	77496	0.98
19	53.05	80569	1.02
20	54.05	21980	0.28
21	55.00	15105	0.19
22	55.95	9266	0.12
23	57.05	15402	0.20
24	57.95	4775	0.06
25	58.95	3044	0.04
26	60.05	3365	0.04
27	61.05	10584	0.13
28	62.05	60116	0.76
29	63.05	317696	4.02
30	64.05	393633	4.99
31	65.05	447623	5.67
32	65.95	117789	1.49
33	66.95	9633	0.12
34	68.15	35220	0.45
35	69.05	222466	2.82
36	70.05	23528	0.30
37	71.15	6023	0.08
38	72.15	1928	0.02
39	73.15	7140	0.09
40	74.15	62633	0.79
41	75.15	326402	4.13
42	76.15	260811	3.30
43	77.10	902310	11.43
44	78.05	343569	4.35

#	m/z	Abs. Int.	Rel. Int.
45	79.00	366450	4.64
46	79.95	85307	1.08
47	80.95	4751	0.06
48	82.15	10363	0.13
49	83.10	26385	0.33
50	84.05	12958	0.16
51	85.05	4878	0.06
52	86.05	5983	0.08
53	87.05	6351	0.08
54	88.05	12801	0.16
55	89.05	110646	1.40
56	90.05	129304	1.64
57	91.05	784823	9.94
58	92.05	613199	7.77
59	93.05	1003785	12.71
60	94.05	341360	4.32
61	95.05	1971283	24.97
62	95.95	361272	4.58
63	96.95	3302	0.04
64	97.95	6385	0.08
65	98.50	7124	0.09
66	99.45	3291	0.04
67	100.05	2691	0.03
68	101.05	15534	0.20
69	102.05	94525	1.20
70	103.05	411554	5.21
71	104.05	1037438	13.14
72	105.00	1331807	16.87
73	105.95	287748	3.64
74	106.95	69721	0.88
75	107.95	16911	0.21
76	108.70	4989	0.06
77	109.75	11610	0.15
78	110.75	39085	0.50
79	111.65	19614	0.25
80	112.95	18319	0.23
81	113.85	6635	0.08
82	115.15	14074	0.18
83	116.15	25605	0.32
84	117.15	84020	1.06
85	118.15	439650	5.57
86	119.15	463864	5.88
87	120.10	1673753	21.20
88	121.05	345411	4.38

#	m/z	Abs. Int.	Rel. Int.
89	122.05	34755	0.44
90	123.05	7791	0.10
91	124.05	1053	0.01
92	125.20	2660	0.03
93	126.25	3479	0.04
94	127.15	6908	0.09
95	128.15	9346	0.12
96	129.25	6116	0.08
97	130.15	9031	0.11
98	131.25	12338	0.16
99	132.20	49986	0.63
100	133.15	38733	0.49
101	134.15	6535	0.08
102	135.15	1063	0.01
103	136.20	68	0.00
104	137.20	327	0.00
105	138.00	585	0.01
106	139.05	5335	0.07
107	140.05	4658	0.06
108	141.05	11126	0.14
109	141.95	3783	0.05
110	143.05	1761	0.02
111	144.15	3514	0.04
112	145.15	4227	0.05
113	146.05	20442	0.26
114	147.15	12651	0.16
115	148.10	39289	0.50
116	149.05	8085	0.10
117	150.65	3742	0.05
118	151.65	14616	0.19
119	152.65	47089	0.60
120	153.60	299400	3.79
121	154.55	98973	1.25
122	155.55	15530	0.20
123	158.15	2240	0.03
124	159.15	1871	0.02
125	160.15	189864	2.40
126	161.05	1259571	15.95
127	162.05	315402	4.00
128	163.05	22404	0.28
129	164.15	8184	0.10
130	165.15	40922	0.52
131	166.05	20192	0.26
132	167.05	17377	0.22

#	m/z	Abs. Int.	Rel. Int.
133	168.05	10881	0.14
134	169.05	7502	0.10
135	170.05	3443	0.04
136	171.05	1156	0.01
137	171.55	1436	0.02
138	172.50	2305	0.03
139	173.45	1642	0.02
140	174.15	1290	0.02
141	175.05	4307	0.05
142	176.15	2721	0.03
143	177.15	6394	0.08
144	178.15	16654	0.21
145	179.05	31596	0.40
146	180.05	17423	0.22
147	181.05	16067	0.20
148	182.05	9851	0.12
149	183.10	7932	0.10
150	184.05	7410	0.09
151	185.00	2520	0.03
152	186.05	3223	0.04
153	187.05	2130	0.03
154	188.05	715781	9.07
155	189.05	7894720	100.00
156	189.95	1615628	20.46
157	190.95	34543	0.44
158	191.95	6629	0.08
159	193.15	5896	0.07
160	194.15	13877	0.18
161	195.10	40685	0.52
162	196.05	13173	0.17
163	197.15	13231	0.17
164	198.05	85554	1.08
165	199.05	18352	0.23
166	200.05	2667	0.03
167	201.05	6458	0.08
168	202.05	5563	0.07
169	202.90	2959	0.04
170	203.95	4753	0.06
171	204.95	3806	0.05
172	205.95	6834	0.09
173	206.90	10753	0.14
174	207.85	5510	0.07
175	209.05	1325	0.02
176	210.05	2614	0.03
177	211.05	4116	0.05
178	212.05	5140	0.07
179	212.95	2221	0.03
180	214.15	1106	0.01
181	215.15	15003	0.19
182	216.05	97416	1.23
183	217.05	50229	0.64
184	218.05	15880	0.20
185	219.05	1114	0.01
186	220.10	1121	0.01
187	221.15	1899	0.02
188	222.15	6137	0.08
189	223.15	21656	0.27
190	224.10	164544	2.08
191	225.05	44429	0.56
192	226.05	4377	0.06
193	227.05	1229	0.02
194	228.10	966	0.01
195	229.10	324	0.00
196	231.10	109	0.00
197	232.10	640	0.01
198	233.10	233	0.00
199	235.10	192	0.00
200	236.10	144	0.00
201	237.10	302	0.00
202	237.95	1561	0.02
203	238.95	2349	0.03
204	239.85	3301	0.04
205	240.90	666	0.01
206	241.90	2075	0.03
207	242.90	128	0.00
208	243.90	285	0.00
209	244.90	200	0.00
210	246.00	1192	0.02
211	247.05	3307	0.04
212	248.05	1908	0.02
213	249.00	16194	0.21
214	250.05	6715	0.09
215	251.05	9238	0.12
216	251.95	3947	0.05
217	252.95	1569	0.02
218	254.00	736	0.01

#	m/z	Abs. Int.	Rel. Int.
219	259.00	466	0.01
220	259.90	389	0.00
221	260.95	3169	0.04
222	261.95	2574	0.03
223	262.95	7624	0.10
224	263.95	13815	0.17
225	264.90	30913	0.39
226	265.85	11111	0.14
227	266.85	1936	0.02
228	267.90	530	0.01
229	268.90	128	0.00
230	269.90	72	0.00
231	271.90	222	0.00
232	272.90	203	0.00
233	273.90	595	0.01
234	274.90	851	0.01
235	275.95	6041	0.08
236	276.95	16680	0.21
237	277.95	14543	0.18
238	278.90	20022	0.25
239	279.85	14816	0.19
240	280.85	4492	0.06
241	281.85	2063	0.03
242	282.90	504	0.01
243	283.90	137	0.00
244	284.90	389	0.00
245	285.90	512	0.01
246	286.90	278	0.00
247	288.05	1590	0.02
248	289.05	3009	0.04
249	290.05	30996	0.39
250	291.05	47394	0.60
251	291.95	48808	0.62
252	293.05	114029	1.44
253	294.05	1035219	13.11
254	294.95	273493	3.46
255	295.95	10060	0.13
256	296.95	902	0.01
257	299.00	347	0.00
258	300.00	347	0.00
259	301.00	230	0.00
260	302.00	72	0.00
261	305.00	624	0.01
262	306.05	4198	0.05
263	307.05	30701	0.39
264	308.05	1000090	12.67
265	309.00	4757271	60.26
266	309.95	1273019	16.12
267	310.95	69682	0.88
268	311.95	2129	0.03
269	314.00	75	0.00
270	318.00	16	0.00
271	319.00	311	0.00
272	320.00	508	0.01
273	321.00	22	0.00
274	322.00	218	0.00
275	324.00	39	0.00
276	325.00	603	0.01
277	326.00	569	0.01
278	327.00	491	0.01
279	332.00	60	0.00
280	333.00	109	0.00
281	338.00	36	0.00
282	339.00	399	0.01
283	340.00	280	0.00
284	341.00	161	0.00
285	342.00	250	0.00
286	344.00	165	0.00
287	345.00	399	0.01
288	346.00	642	0.01
289	347.00	390	0.00
290	358.00	62	0.00
291	359.00	371	0.00
292	360.00	297	0.00
293	361.00	202	0.00
294	362.00	176	0.00
295	363.00	59	0.00
296	364.00	227	0.00
297	365.00	440	0.01
298	366.00	530	0.01
299	367.00	279	0.00
300	368.00	143	0.00
301	373.00	51	0.00
302	378.00	126	0.00
303	379.00	401	0.01
304	380.00	443	0.01

#	m/z	Abs. Int.	Rel. Int.
305	381.00	222	0.00
306	382.00	103	0.00
307	383.00	55	0.00
308	384.00	151	0.00
309	385.00	288	0.00
310	386.00	627	0.01
311	387.00	462	0.01
312	392.00	61	0.00
313	393.00	139	0.00
314	399.00	547	0.01
315	400.00	261	0.00
316	401.00	222	0.00
317	402.00	117	0.00
318	404.00	262	0.00
319	405.00	504	0.01
320	406.00	409	0.01
321	407.00	495	0.01
322	412.00	181	0.00
323	413.00	127	0.00
324	418.00	290	0.00
325	419.00	499	0.01
326	420.00	263	0.00
327	421.00	164	0.00
328	422.00	64	0.00
329	424.00	245	0.00
330	425.00	376	0.00
331	426.00	483	0.01
332	427.00	608	0.01
333	432.00	15	0.00
334	433.00	155	0.00
335	438.00	11	0.00
336	439.00	416	0.01
337	440.00	269	0.00
338	441.00	214	0.00
339	442.00	79	0.00
340	444.00	354	0.00
341	445.00	425	0.01
342	446.00	554	0.01
343	447.00	331	0.00
344	452.00	91	0.00
345	453.00	1	0.00
346	458.00	188	0.00
347	459.00	178	0.00
348	460.00	382	0.00
349	461.00	15	0.00
350	462.00	115	0.00
351	463.00	2	0.00
352	464.00	318	0.00
353	465.00	575	0.01
354	466.00	398	0.01
355	467.00	422	0.01
356	472.00	133	0.00
357	473.00	100	0.00
358	478.00	36	0.00
359	479.00	494	0.01
360	480.00	117	0.00
361	481.00	250	0.00
362	482.00	1	0.00
363	484.00	165	0.00
364	485.00	343	0.00
365	486.00	451	0.01
366	487.00	251	0.00
367	498.00	14	0.00
368	499.00	353	0.00
369	500.00	261	0.00
370	501.00	69	0.00
371	504.00	184	0.00
372	505.00	460	0.01
373	506.00	351	0.00
374	507.00	279	0.00
375	512.00	175	0.00
376	513.00	62	0.00
377	518.00	80	0.00
378	519.00	433	0.01
379	520.00	331	0.00
380	522.00	50	0.00
381	524.00	339	0.00
382	525.00	276	0.00
383	526.00	470	0.01
384	527.00	135	0.00
385	533.00	8	0.00
386	538.00	132	0.00
387	539.00	314	0.00
388	540.00	275	0.00
389	541.00	10	0.00
390	542.00	88	0.00

Elemental Composition Report

Single Mass Analysis

Tolerance = 5.0 PPM / DBE: min = -50.0, max = 80.0
Element prediction: Off
Number of isotope peaks used for i-FIT = 5

Monoisotopic Mass, Even Electron Ions
216 formula(e) evaluated with 2 results within limits (up to 50 closest results for each mass)
Elements Used:
C: 0-25 H: 0-25 N: 0-3 O: 0-3 F: 0-5

190125_IVA1419 16 (0.177) Cm (14:17-(2:8+32:72))
1: TOF MS ES+

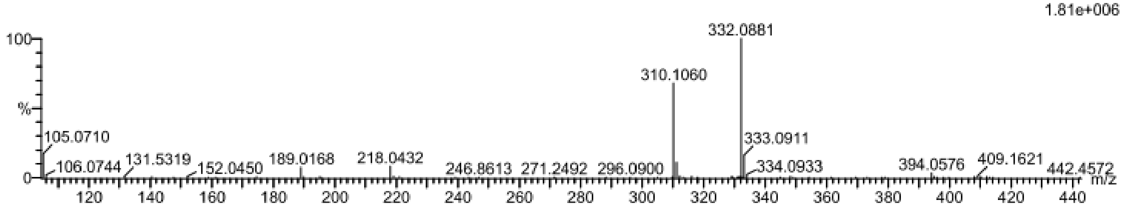

Minimum:				-50.0				
Maximum:		15.0	5.0	80.0				
Mass	Calc. Mass	mDa	PPM	DBE	i-FIT	Norm	Conf(%)	Formula
310.1060	310.1055	0.5	1.6	8.5	58.5	0.417	65.90	C16 H15 N O2 F3
	310.1066	-0.6	-1.9	4.5	59.1	1.076	34.10	C13 H16 N O3 F4

Method

[Comment]

==== Analytical Line 1 ====

[GC-2010]

Column Oven Temp.	:30.0 °C
Injection Temp.	:150.00 °C
Injection Mode	:Split
Pressure	:12.8 kPa
Total Flow	:6.6 mL/min
Column Flow	:0.60 mL/min
Linear Velocity	:27.9 cm/sec
Purge Flow	:3.0 mL/min
Split Ratio	:5.0

[GCMS-QP2010 Ultra]

IonSourceTemp	:200.00 °C
Interface Temp.	:200.00 °C
Solvent Cut Time	:0.30 min
Detector Gain Mode	:Relative
Detector Gain	:1.29 kV +0.00 kV
Threshold	:0

[MS Table]

--Group 1 - Event 1--

Start Time	:0.30min
End Time	:25.00min
ACQ Mode	:Scan
Event Time	:0.10sec
Scan Speed	:20000
Start m/z	:35.00
End m/z	:1090.00

Sample Information

Sample Name	: M.Satenik
Sample ID	: IVA_1427
Analyzed	: 2019-01-18 09:05:33
Vial #	: 12
Injection Volume	: 0.10
$EndIf$Data File	: C:\GCMSsolution\Data\widma\2019\01_2019\M
Method File	: C:\GCMSsolution\System\Tune1\DI EI 1 metoda
Tuning File	: C:\GCMSsolution\System\Tune1\tuning 07.01.2(

Chromatogram M.Satenik C:\GCMSsolution\Data\widma\2019\01_2019\M.Satenik_IVA1427.QGD

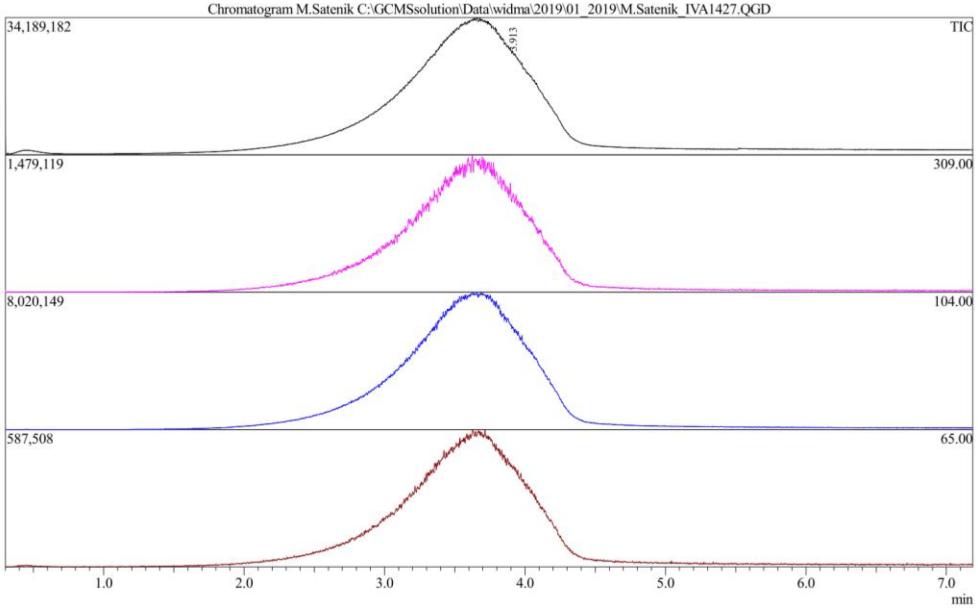

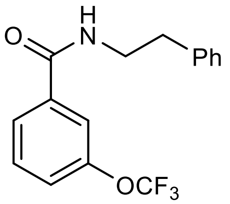

N-phenethyl-3-(trifluoromethoxy)benzamide
Chemical Formula: $C_{16}H_{14}F_3NO_2$
Molecular Weight: 309.29

Compound **4v**

Spectrum

Line#:1 R.Time:3.913(Scan#:2169)
MassPeaks:641
RawMode:Single 3.913(2169) BasePeak:189(6010310)
BG Mode:None Group 1 - Event 1 Scan

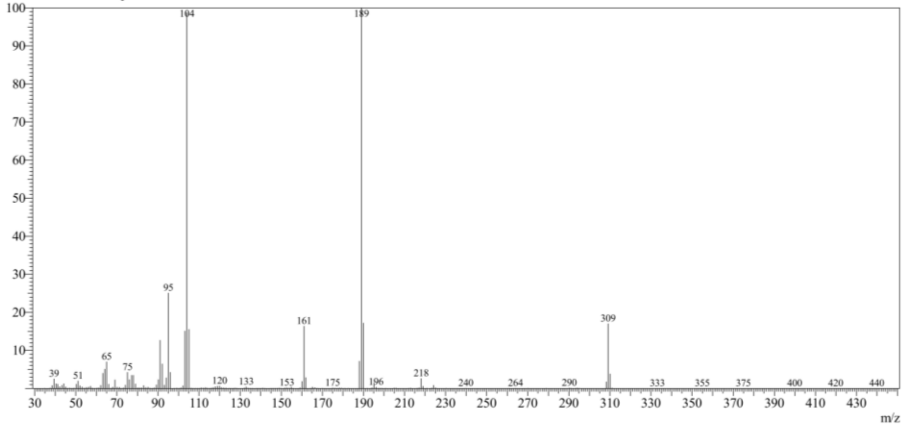

Mass Table
Line#:1 R.Time:3.913(Scan#:2169)
MassPeaks:641
RawMode:Single 3.913(2169) BasePeak:189(6010310)
BG Mode:None Group 1 - Event 1 Scan

#	m/z	Abs. Int.	Rel. Int.
1	34.90	2367	0.04
2	36.45	1724	0.03
3	37.45	8690	0.14
4	38.45	47097	0.78
5	39.40	152086	2.53
6	40.35	75875	1.26
7	41.20	68839	1.15
8	42.15	27615	0.46
9	43.25	50479	0.84
10	44.15	74287	1.24
11	45.15	23576	0.39
12	46.10	2860	0.05
13	47.05	6774	0.11
14	48.05	3396	0.06
15	49.15	7662	0.13
16	50.15	67134	1.12
17	51.10	117270	1.95
18	52.05	44002	0.73
19	53.05	25775	0.43
20	54.10	6908	0.11
21	55.15	19538	0.33
22	56.15	20184	0.34
23	57.15	37545	0.62
24	58.05	8890	0.15
25	59.05	3380	0.06
26	60.15	5827	0.10
27	61.15	10094	0.17
28	62.15	48638	0.81
29	63.15	241122	4.01
30	64.15	305892	5.09
31	65.05	419050	6.97
32	66.05	69324	1.15
33	67.05	6206	0.10
34	68.15	22696	0.38
35	69.10	134574	2.24
36	70.05	20359	0.34
37	71.25	17996	0.30
38	72.15	3617	0.06
39	73.15	10738	0.18
40	74.15	52123	0.87
41	75.15	255351	4.25
42	76.05	141660	2.36
43	77.15	209752	3.49
44	78.05	209211	3.48
45	79.05	72724	1.21
46	80.05	9171	0.15
47	81.05	9659	0.16
48	82.05	11744	0.20
49	83.05	45518	0.76
50	83.95	12770	0.21
51	85.05	22600	0.38
52	85.95	6134	0.10
53	87.15	5227	0.09
54	88.15	12137	0.20
55	89.15	59006	0.98
56	90.15	138343	2.30
57	91.10	762321	12.68
58	92.05	386512	6.43
59	93.05	52551	0.87
60	94.05	169701	2.82
61	95.05	1505956	25.06
62	95.95	254337	4.23
63	96.95	1668	0.03
64	97.95	4694	0.08
65	99.05	6383	0.11
66	100.15	3846	0.06
67	101.15	10609	0.18
68	102.15	41555	0.69
69	103.15	906884	15.09
70	104.10	5935017	98.75
71	105.05	932795	15.52
72	106.05	8151	0.14
73	107.05	3438	0.06
74	108.05	3039	0.05
75	109.05	4771	0.08
76	110.05	4359	0.07
77	111.05	11699	0.19
78	111.95	8175	0.14
79	113.05	15755	0.26
80	113.95	6405	0.11
81	114.95	5110	0.09
82	115.95	5708	0.09
83	116.95	14672	0.24
84	117.95	30085	0.50
85	118.95	34856	0.58
86	119.95	39323	0.65
87	120.85	16076	0.27
88	121.85	10041	0.17
89	123.15	7191	0.12
90	124.55	2250	0.04
91	125.45	2995	0.05
92	126.55	4019	0.07
93	127.55	6306	0.10
94	128.45	4206	0.07
95	129.50	895	0.01
96	130.50	466	0.01
97	131.95	7260	0.12
98	132.90	25844	0.43
99	133.85	6862	0.11
100	134.85	2316	0.04
101	135.90	2017	0.03
102	137.00	1029	0.02
103	137.95	1850	0.03
104	139.00	4447	0.07
105	140.05	4112	0.07
106	141.00	7000	0.12
107	141.95	1803	0.03
108	143.00	920	0.02
109	143.90	1162	0.02
110	144.85	3373	0.06
111	145.95	3092	0.05
112	146.95	2906	0.05
113	147.90	1851	0.03
114	148.95	6053	0.10
115	149.85	1913	0.03
116	150.75	1252	0.02
117	151.75	7104	0.12
118	152.75	10582	0.18
119	153.75	7777	0.13
120	154.75	21897	0.36
121	155.65	5282	0.09
122	156.70	753	0.01
123	158.05	1710	0.03
124	159.05	12233	0.20
125	160.05	112254	1.87
126	161.05	983070	16.36
127	161.95	172921	2.88
128	162.95	14	0.00
129	164.15	3414	0.06
130	165.15	20520	0.34
131	166.05	9256	0.15
132	166.75	8023	0.13
133	167.75	5197	0.09
134	168.75	1134	0.02
135	170.05	3895	0.06
136	170.95	1405	0.02
137	172.00	120	0.00
138	172.90	1268	0.02
139	173.95	1333	0.02
140	174.95	6053	0.10
141	175.85	2267	0.04
142	176.45	1859	0.03
143	177.45	3754	0.06
144	178.35	5815	0.10
145	179.35	5833	0.10
146	180.90	3705	0.06
147	181.85	1499	0.02
148	182.70	2411	0.04
149	183.70	434	0.01
150	184.70	652	0.01
151	186.05	1196	0.02
152	188.05	431086	7.17
153	189.05	6010310	100.00
154	189.95	1033357	17.19
155	191.95	4093	0.07
156	193.15	1398	0.02
157	194.15	10443	0.17
158	195.10	91108	1.52
159	196.05	22051	0.37
160	197.05	2224	0.04
161	197.95	4333	0.07
162	198.95	2645	0.04
163	200.00	891	0.01
164	201.00	3067	0.05
165	201.95	1754	0.03
166	202.90	624	0.01
167	203.85	1324	0.02
168	204.95	5690	0.09
169	205.85	7473	0.12
170	206.85	1475	0.02
171	207.90	866	0.01
172	208.90	89	0.00
173	209.90	125	0.00
174	211.00	453	0.01
175	211.95	2655	0.04
176	213.00	5672	0.09
177	213.95	1279	0.02
178	215.00	769	0.01
179	216.05	3199	0.05
180	217.05	9620	0.16
181	218.05	157443	2.62
182	218.95	35682	0.59
183	219.95	1189	0.02
184	220.95	1990	0.03
185	222.25	1028	0.02
186	223.25	6872	0.11
187	224.15	50304	0.84
188	225.15	9989	0.17
189	226.15	545	0.01
190	227.20	579	0.01
191	228.20	516	0.01
192	229.20	943	0.02
193	230.20	3947	0.07
194	231.20	669	0.01
195	232.20	784	0.01
196	233.20	597	0.01
197	234.20	373	0.01
198	235.20	719	0.01
199	236.20	494	0.01
200	237.20	723	0.01
201	238.20	422	0.01
202	238.80	882	0.01
203	239.85	3641	0.06
204	240.75	1761	0.03
205	241.80	579	0.01
206	242.80	555	0.01
207	245.80	5	0.00
208	246.80	640	0.01
209	247.80	363	0.01
210	248.80	355	0.01
211	249.80	232	0.00
212	250.80	541	0.01
213	252.80	675	0.01
214	253.80	525	0.01
215	254.80	603	0.01
216	255.80	1014	0.02
217	256.80	1133	0.02
218	257.80	778	0.01
219	258.80	631	0.01
220	259.90	743	0.01
221	260.85	2830	0.05
222	261.85	1405	0.02
223	262.90	20	0.00
224	264.05	3147	0.05
225	265.10	824	0.01
226	266.10	119	0.00
227	267.10	1370	0.02
228	268.10	137	0.00
229	270.10	3619	0.06
230	272.10	452	0.01
231	273.10	310	0.01
232	274.10	885	0.01
233	275.10	758	0.01
234	277.10	428	0.01
235	278.00	680	0.01
236	279.05	2751	0.05
237	279.95	1655	0.03
238	281.00	953	0.02
239	282.00	847	0.01
240	283.00	120	0.00
241	287.00	74	0.00
242	288.00	873	0.01
243	289.10	785	0.01
244	290.15	7380	0.12
245	291.05	2191	0.04
246	292.10	859	0.01
247	293.25	1959	0.03
248	294.15	5960	0.10
249	295.15	1873	0.03
250	296.20	240	0.00
251	298.20	61	0.00
252	299.20	452	0.01
253	300.20	750	0.01
254	301.20	389	0.01
255	303.20	48	0.00
256	306.10	516	0.01
257	308.15	103057	1.71
258	309.10	1021423	16.99
259	310.05	228967	3.81
260	311.05	7719	0.13
261	312.05	1512	0.03
262	313.10	417	0.01
263	314.10	500	0.01
264	315.10	319	0.01
265	316.10	47	0.00
266	318.10	394	0.01
267	319.10	1073	0.02
268	320.10	343	0.01
269	321.10	559	0.01
270	322.10	20	0.00
271	327.10	295	0.00
272	328.10	57	0.00
273	332.10	21	0.00
274	333.10	336	0.01
275	334.10	283	0.00
276	335.10	265	0.00
277	336.10	89	0.00
278	338.10	293	0.00
279	339.10	388	0.01
280	340.10	581	0.01
281	341.10	517	0.01
282	342.10	104	0.00
283	343.10	97	0.00
284	347.10	67	0.00
285	348.10	33	0.00
286	352.10	50	0.00
287	353.10	119	0.00
288	354.10	473	0.01
289	354.95	1535	0.03
290	355.90	624	0.01
291	356.90	52	0.00
292	357.90	98	0.00
293	358.90	271	0.00
294	359.90	464	0.01
295	360.90	476	0.01
296	361.90	203	0.00
297	366.90	253	0.00
298	367.90	166	0.00
299	372.90	220	0.00
300	373.90	258	0.00
301	374.90	529	0.01
302	375.90	243	0.00
303	376.90	155	0.00
304	377.90	56	0.00
305	378.90	124	0.00
306	379.90	644	0.01
307	380.90	350	0.01
308	381.90	180	0.00
309	386.90	392	0.01
310	387.90	133	0.00
311	392.90	266	0.00
312	393.90	479	0.01
313	394.90	372	0.01
314	395.90	394	0.01
315	397.90	30	0.00
316	398.90	256	0.00
317	399.90	686	0.01
318	400.90	472	0.01
319	401.90	113	0.00
320	406.90	562	0.01
321	407.90	115	0.00
322	412.90	327	0.01
323	413.90	630	0.01
324	414.90	492	0.01
325	415.90	189	0.00
326	418.90	253	0.00
327	419.90	729	0.01
328	420.90	412	0.01
329	421.90	245	0.00
330	426.90	744	0.01
331	427.90	43	0.00
332	432.90	530	0.01
333	433.90	523	0.01
334	434.90	523	0.01
335	438.90	258	0.00
336	439.90	869	0.01
337	440.90	526	0.01
338	441.90	194	0.00
339	446.90	508	0.01
340	447.90	62	0.00
341	452.90	536	0.01
342	453.90	425	0.01
343	454.90	555	0.01
344	458.90	313	0.01
345	459.90	954	0.02
346	460.90	346	0.01
347	461.90	33	0.00
348	466.90	480	0.01
349	467.90	84	0.00
350	472.90	695	0.01
351	473.90	381	0.01
352	474.90	307	0.01
353	478.90	655	0.01
354	479.90	1012	0.02
355	480.90	224	0.00
356	486.90	367	0.01
357	487.90	141	0.00
358	492.90	870	0.01
359	493.90	530	0.01
360	494.90	142	0.00
361	498.90	566	0.01
362	499.90	1025	0.02
363	500.90	503	0.01
364	505.90	58	0.00
365	506.90	81	0.00
366	507.90	364	0.01
367	511.90	264	0.00
368	512.90	654	0.01
369	513.90	389	0.01
370	514.90	247	0.00
371	517.90	49	0.00
372	518.90	739	0.01
373	519.90	801	0.01
374	520.90	262	0.00
375	525.90	88	0.00
376	526.90	315	0.01
377	531.90	340	0.01
378	532.90	740	0.01
379	533.90	587	0.01
380	538.90	880	0.01
381	539.90	578	0.01
382	540.90	326	0.01
383	545.90	229	0.00
384	546.90	169	0.00
385	547.90	11	0.00
386	551.90	468	0.01
387	552.90	765	0.01

Sample Information

Sample Name	: M.Satenik
Sample ID	: IVA_1382
Analyzed	: 2019-01-10 12:40:17
Vial #	: 12
Injection Volume	: 6.00
$EndIf$Data File	: C:\GCMSsolution\Data\widma\2019\01_2019\M
Method File	: C:\GCMSsolution\System\Tune1\DI EI 1 metoda
Tuning File	: C:\GCMSsolution\System\Tune1\tuning 07.01.2(

Method

[Comment]

═══ Analytical Line 1 ═══

[GC-2010]

Column Oven Temp.	:30.0 °C
Injection Temp.	:150.00 °C
Injection Mode	:Split
Pressure	:12.8 kPa
Total Flow	:6.6 mL/min
Column Flow	:0.60 mL/min
Linear Velocity	:27.9 cm/sec
Purge Flow	:3.0 mL/min
Split Ratio	:5.0

[GCMS-QP2010 Ultra]

IonSourceTemp	:200.00 °C
Interface Temp.	:200.00 °C
Solvent Cut Time	:0.30 min
Detector Gain Mode	:Relative
Detector Gain	:1.29 kV +0.00 kV
Threshold	:0

[MS Table]
--Group 1 - Event 1--

Start Time	:0.30min
End Time	:25.00min
ACQ Mode	:Scan
Event Time	:0.10sec
Scan Speed	:20000
Start m/z	:35.00
End m/z	:1090.00

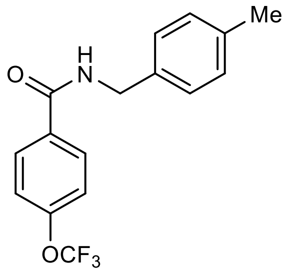

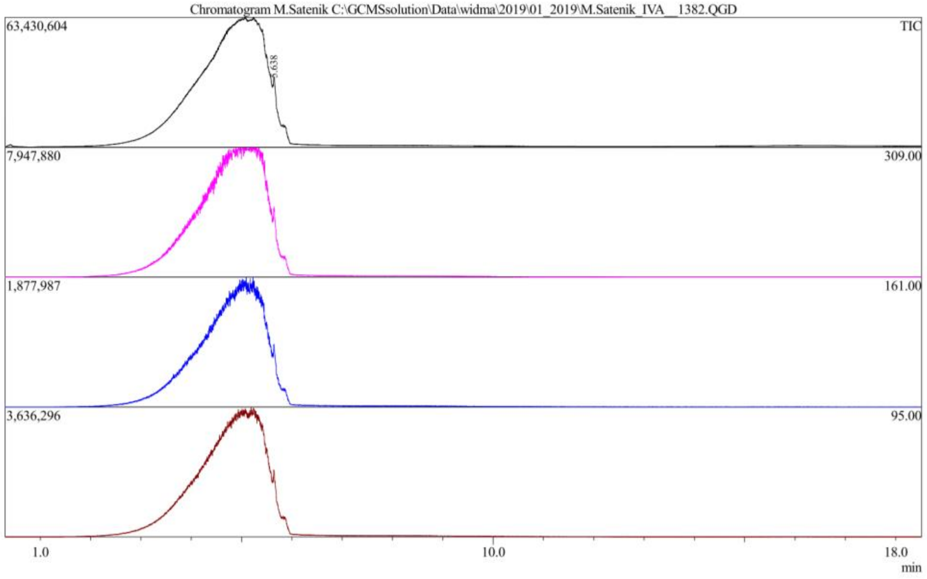

N-(4-methylbenzyl)-4-(trifluoromethoxy)benzamide
Chemical Formula: $C_{16}H_{14}F_3NO_2$
Molecular Weight: 309.29

Compound **4w**

Spectrum

Line#:1 R.Time:5.638(Scan#:3204)
MassPeaks:603
RawMode:Single 5.638(3204) BasePeak:189(7989574)
BG Mode:None Group 1 - Event 1 Scan

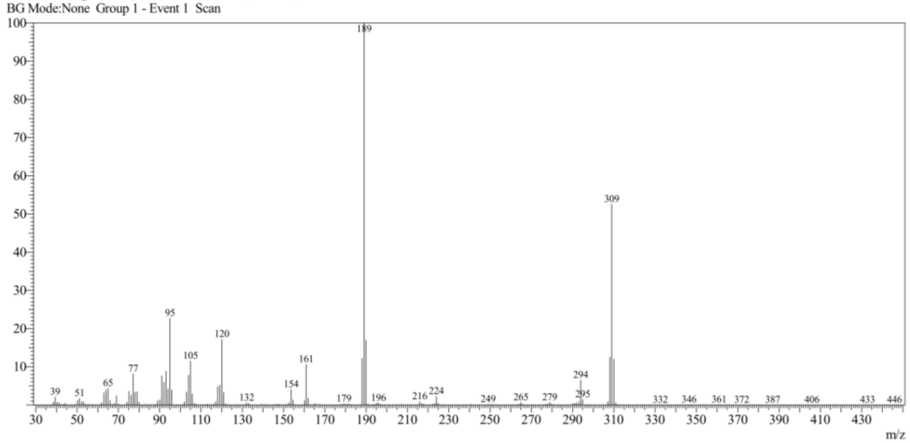

Mass Table
Line#:1 R.Time:5.638(Scan#:3204)
MassPeaks:603
RawMode:Single 5.638(3204) BasePeak:189(7989574)
BG Mode:None Group 1 - Event 1 Scan

#	m/z	Abs. Int.	Rel. Int.
1	35.00	154	0.00
2	36.45	1878	0.02
3	37.45	9844	0.12
4	38.45	61133	0.77
5	39.35	168609	2.11
6	40.35	56416	0.71
7	41.25	48373	0.61
8	42.25	10016	0.13
9	43.25	13935	0.17
10	44.20	31341	0.39
11	45.15	5737	0.07
12	46.35	1960	0.02
13	47.25	4102	0.05
14	48.20	868	0.01
15	49.25	9159	0.11
16	50.25	97050	1.21
17	51.15	140636	1.76
18	52.15	65998	0.83
19	53.10	66754	0.84
20	54.05	18961	0.24
21	55.30	9235	0.12
22	56.25	8086	0.10
23	57.25	9103	0.11
24	58.00	3216	0.04
25	58.95	2452	0.03
26	60.05	2794	0.03
27	61.05	7932	0.10
28	62.05	48656	0.61
29	63.05	257056	3.22
30	64.05	308719	3.86
31	65.00	346874	4.34
32	65.95	88468	1.11
33	66.95	9434	0.12
34	68.15	33077	0.41
35	69.10	194660	2.44
36	70.05	16759	0.21
37	71.15	1574	0.02
38	72.15	1931	0.02
39	73.15	7081	0.09
40	74.15	63760	0.80
41	75.15	287962	3.60
42	76.15	203427	2.55
43	77.10	654892	8.20
44	78.05	268900	3.37

#	m/z	Abs. Int.	Rel. Int.
45	79.00	275731	3.45
46	79.95	61778	0.77
47	80.95	2908	0.04
48	82.15	6800	0.09
49	83.15	15020	0.19
50	84.05	4714	0.06
51	84.95	4213	0.05
52	86.05	4100	0.05
53	87.05	5408	0.07
54	88.05	12680	0.16
55	89.05	92942	1.16
56	90.05	108241	1.35
57	91.05	609193	7.62
58	92.05	478567	5.99
59	93.05	704392	8.82
60	94.05	333460	4.17
61	95.05	1810465	22.66
62	95.95	307973	3.85
63	98.20	3551	0.04
64	99.15	2877	0.04
65	99.90	1275	0.02
66	100.95	7675	0.10
67	101.95	65741	0.82
68	102.95	274056	3.43
69	103.95	624210	7.81
70	104.95	919405	11.51
71	105.85	230425	2.88
72	106.85	37310	0.47
73	107.85	10242	0.13
74	108.85	1843	0.02
75	110.05	3761	0.05
76	111.05	8953	0.11
77	112.05	9596	0.12
78	113.00	19048	0.24
79	113.95	6355	0.08
80	115.15	8465	0.11
81	116.15	17756	0.22
82	117.15	69342	0.87
83	118.15	384816	4.82
84	119.15	415777	5.20
85	120.10	1371871	17.17
86	121.05	260381	3.26
87	122.05	23718	0.30
88	123.05	5741	0.07

#	m/z	Abs. Int.	Rel. Int.
89	124.10	631	0.01
90	125.20	862	0.01
91	126.25	2586	0.03
92	127.25	4627	0.06
93	128.15	6257	0.08
94	129.15	5799	0.07
95	130.15	3922	0.05
96	131.25	11206	0.14
97	132.20	41420	0.52
98	133.15	27067	0.34
99	134.15	3537	0.04
100	135.20	249	0.00
101	138.10	661	0.01
102	139.15	8433	0.11
103	140.00	2733	0.03
104	140.95	9794	0.12
105	141.95	3415	0.04
106	142.95	1287	0.02
107	145.05	3049	0.04
108	146.05	12865	0.16
109	146.95	9734	0.12
110	148.00	13947	0.17
111	148.95	4357	0.05
112	149.95	1475	0.02
113	150.65	3660	0.05
114	151.65	13191	0.17
115	152.65	47526	0.59
116	153.65	324744	4.06
117	154.55	104262	1.30
118	155.55	5941	0.07
119	156.55	1397	0.02
120	158.05	880	0.01
121	159.05	3060	0.04
122	160.05	92310	1.16
123	161.00	850894	10.65
124	161.95	142184	1.78
125	162.95	3154	0.04
126	164.05	3510	0.04
127	165.05	29318	0.37
128	165.95	16686	0.21
129	167.10	11467	0.14
130	168.05	6501	0.08
131	168.95	5990	0.07
132	169.95	3279	0.04

#	m/z	Abs. Int.	Rel. Int.
133	170.95	1466	0.02
134	171.80	689	0.01
135	172.75	1431	0.02
136	173.85	1764	0.02
137	174.85	2146	0.03
138	175.85	4871	0.06
139	177.15	2549	0.03
140	178.15	13132	0.16
141	179.15	23593	0.30
142	180.05	12674	0.16
143	181.10	17737	0.22
144	181.70	9610	0.12
145	182.70	9582	0.12
146	183.65	3444	0.04
147	184.65	2004	0.03
148	186.05	5958	0.07
149	188.05	970965	12.15
150	189.00	7989574	100.00
151	189.95	1359606	17.02
152	190.95	18230	0.23
153	191.95	6333	0.08
154	193.05	4768	0.06
155	194.05	12631	0.16
156	195.05	37274	0.47
157	196.00	39060	0.49
158	196.95	13374	0.17
159	198.25	701	0.01
160	199.20	2462	0.03
161	200.25	1431	0.02
162	201.20	3715	0.05
163	202.15	947	0.01
164	202.95	3537	0.04
165	203.95	5992	0.07
166	204.95	4069	0.05
167	205.95	4509	0.06
168	206.90	7690	0.10
169	207.85	4761	0.06
170	208.85	3278	0.04
171	209.40	3096	0.04
172	210.35	3087	0.04
173	211.00	942	0.01
174	212.05	2645	0.03
175	212.95	2999	0.04
176	214.05	4179	0.05
177	215.05	9343	0.12
178	215.95	71043	0.89
179	216.95	27234	0.34
180	217.95	14692	0.18
181	218.95	1995	0.02
182	219.70	1395	0.02
183	221.15	2178	0.03
184	222.15	13105	0.16
185	223.15	31093	0.39
186	224.05	183037	2.29
187	225.05	34910	0.44
188	226.05	2638	0.03
189	227.05	2208	0.03
190	228.10	666	0.01
191	231.10	466	0.01
192	231.70	542	0.01
193	232.65	1539	0.02
194	233.70	82	0.00
195	234.70	810	0.01
196	238.05	1852	0.02
197	239.05	2689	0.03
198	240.05	4656	0.06
199	241.00	7223	0.09
200	241.95	1947	0.02
201	245.00	279	0.00
202	246.00	622	0.01
203	247.15	1555	0.02
204	248.15	1986	0.02
205	249.15	11958	0.15
206	250.05	5767	0.07
207	250.95	7294	0.09
208	251.85	2905	0.04
209	252.85	1654	0.02
210	253.90	666	0.01
211	256.90	16	0.00
212	258.95	3249	0.04
213	260.00	786	0.01
214	260.95	2062	0.03
215	262.05	2182	0.03
216	263.05	6715	0.08
217	264.05	16317	0.20
218	265.00	50290	0.63

#	m/z	Abs. Int.	Rel. Int.
219	265.95	15439	0.19
220	266.95	2091	0.03
221	267.95	1170	0.01
222	271.00	129	0.00
223	272.00	628	0.01
224	272.90	891	0.01
225	273.85	1245	0.02
226	274.95	2044	0.03
227	275.95	5191	0.06
228	276.95	11950	0.15
229	277.95	20943	0.26
230	278.90	54713	0.68
231	279.85	24459	0.31
232	280.85	6157	0.08
233	281.85	1205	0.02
234	285.90	1261	0.02
235	286.60	503	0.01
236	287.60	993	0.01
237	289.05	4245	0.05
238	290.05	28360	0.35
239	291.05	33535	0.42
240	292.00	47479	0.59
241	293.05	67000	0.84
242	293.95	519280	6.50
243	294.95	116527	1.46
244	295.95	6467	0.08
245	298.00	419	0.01
246	299.00	633	0.01
247	300.00	118	0.00
248	303.00	251	0.00
249	305.05	848	0.01
250	306.05	4954	0.06
251	307.05	72551	0.91
252	308.05	997026	12.48
253	309.00	4196799	52.53
254	309.95	960302	12.02
255	310.95	44723	0.56
256	311.95	4505	0.06
257	312.95	432	0.01
258	314.00	288	0.00
259	315.00	296	0.00
260	318.00	16	0.00
261	319.00	391	0.00
262	320.00	275	0.00
263	325.00	497	0.01
264	326.00	680	0.01
265	326.85	1305	0.02
266	327.80	8	0.00
267	330.80	130	0.00
268	331.40	375	0.00
269	332.45	1395	0.02
270	333.40	253	0.00
271	334.40	556	0.01
272	339.40	811	0.01
273	345.40	703	0.01
274	346.40	2856	0.04
275	351.45	1671	0.02
276	352.40	425	0.01
277	353.40	867	0.01
278	358.40	590	0.01
279	359.40	11	0.00
280	360.95	3353	0.04
281	361.90	407	0.01
282	365.90	749	0.01
283	366.90	113	0.00
284	370.90	614	0.01
285	371.90	782	0.01
286	372.90	218	0.00
287	373.90	528	0.01
288	378.90	490	0.01
289	380.90	429	0.01
290	383.90	408	0.01
291	384.90	394	0.00
292	385.90	501	0.01
293	386.90	554	0.01
294	387.90	97	0.00
295	390.90	65	0.00
296	391.90	533	0.01
297	392.90	575	0.01
298	393.90	303	0.00
299	397.90	213	0.00
300	398.90	565	0.01
301	399.90	330	0.00
302	404.90	35	0.00
303	405.90	755	0.01
304	406.90	752	0.01

#	m/z	Abs. Int.	Rel. Int.
305	410.90	191	0.00
306	411.90	701	0.01
307	412.90	765	0.01
308	417.90	329	0.00
309	418.90	207	0.00
310	419.90	518	0.01
311	424.90	606	0.01
312	425.90	222	0.00
313	426.90	763	0.01
314	430.90	99	0.00
315	431.90	697	0.01
316	432.90	845	0.01
317	433.90	95	0.00
318	434.90	283	0.00
319	437.90	65	0.00
320	438.90	191	0.00
321	439.90	216	0.00
322	445.90	656	0.01
323	446.90	565	0.01
324	450.90	345	0.00
325	451.70	131	0.00
326	452.70	1743	0.02
327	457.70	9	0.00
328	458.70	417	0.01
329	459.70	445	0.01
330	464.70	201	0.00
331	465.70	891	0.01
332	466.70	572	0.01
333	471.60	1452	0.02
334	472.95	1104	0.01
335	478.00	31	0.00
336	479.00	874	0.01
337	485.00	510	0.01
338	486.00	766	0.01
339	487.00	365	0.00
340	491.00	363	0.00
341	492.05	1515	0.02
342	493.10	248	0.00
343	498.10	312	0.00
344	499.10	536	0.01
345	501.10	171	0.00
346	505.10	702	0.01
347	506.10	690	0.01
348	510.10	494	0.01
349	511.10	230	0.00
350	512.10	1022	0.01
351	513.10	271	0.00
352	518.10	716	0.01
353	519.10	238	0.00
354	520.10	361	0.00
355	525.10	1026	0.01
356	526.10	471	0.01
357	527.10	498	0.01
358	531.10	576	0.01
359	532.10	737	0.01
360	533.10	330	0.00
361	538.10	413	0.01
362	539.10	377	0.00
363	544.10	43	0.00
364	545.10	728	0.01
365	546.10	541	0.01
366	547.10	151	0.00
367	550.10	232	0.00
368	551.10	465	0.01
369	552.10	300	0.00
370	553.10	712	0.01
371	558.10	519	0.01
372	559.10	34	0.00
373	564.10	515	0.01
374	565.10	24	0.00
375	566.10	764	0.01
376	567.10	559	0.01
377	571.10	313	0.00
378	572.10	767	0.01
379	573.10	565	0.01
380	578.10	258	0.00
381	579.10	185	0.00
382	584.10	15	0.00
383	585.10	274	0.00
384	586.10	812	0.01
385	591.60	1466	0.02
386	592.60	264	0.00
387	593.60	457	0.01
388	598.60	983	0.01
389	603.60	15	0.00
390	604.60	18	0.00

Method

[Comment]

===== Analytical Line 1 =====

[GC-2010]

Column Oven Temp.	:30.0 °C
Injection Temp.	:150.00 °C
Injection Mode	:Split
Pressure	:12.8 kPa
Total Flow	:6.6 mL/min
Column Flow	:0.60 mL/min
Linear Velocity	:27.7 cm/sec
Purge Flow	:3.0 mL/min
Split Ratio	:5.0

[GCMS-QP2010 Ultra]

IonSourceTemp	:200.00 °C
Interface Temp.	:200.00 °C
Solvent Cut Time	:0.10 min
Detector Gain Mode	:Relative
Detector Gain	:1.36 kV +0.00 kV
Threshold	:0

[MS Table]

--Group 1 - Event 1--

Start Time	:0.10min
End Time	:25.00min
ACQ Mode	:Scan
Event Time	:0.10sec
Scan Speed	:20000
Start m/z	:35.00
End m/z	:1090.00

Sample Information

Sample Name	: M_Satenik
Sample ID	: IVA_1827
Analyzed	: 2019-07-23 09:53:38
Vial #	: 12
Injection Volume	: 1.00
$EndIf$Data File	: C:\GCMSsolution\Data\widma\2019\07_2019\M
Method File	: C:\GCMSsolution\System\Tune1\DI EI 1 metoda
Tuning File	: C:\GCMSsolution\System\Tune1\tuning22.07.20

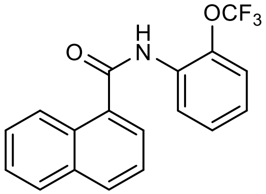

N-(2-(trifluoromethoxy)phenyl)-1-naphthamide **4x**

Chemical Formula: $C_{18}H_{12}F_3NO_2$

Molecular Weight: 331.29

Compound **4x**

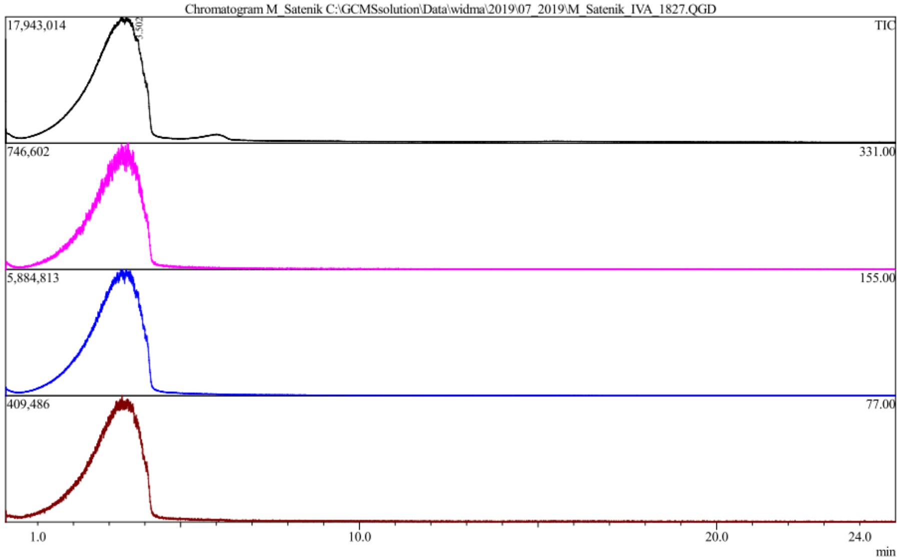

Spectrum

Line#:1 R.Time:3.502(Scan#:2042)
MassPeaks:582
RawMode:Single 3.502(2042) BasePeak:155(5576914)
BG Mode:None Group 1 - Event 1 Scan

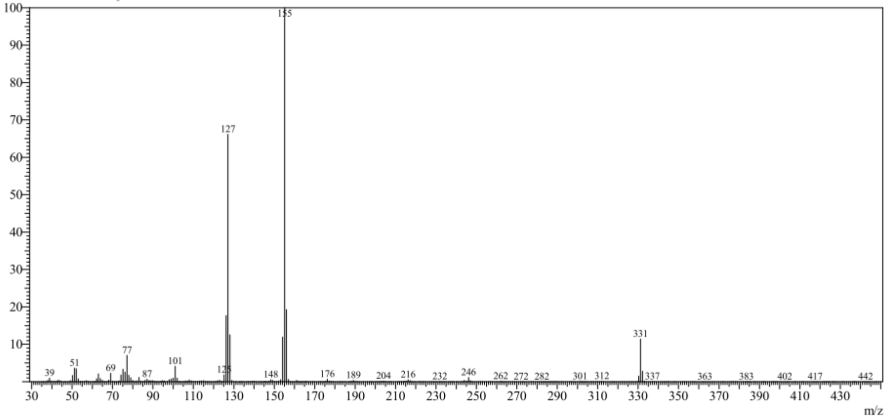

Mass Table
Line#:1 R.Time:3.502(Scan#:2042)
MassPeaks:582
RawMode:Single 3.502(2042) BasePeak:155(5576914)
BG Mode:None Group 1 - Event 1 Scan

#	m/z	Abs. Int.	Rel. Int.
1	35.00	177	0.00
2	35.90	43	0.00
3	36.95	4181	0.07
4	37.95	19332	0.35
5	38.85	52965	0.95
6	39.85	14481	0.26
7	41.15	9608	0.17
8	42.25	11189	0.20
9	43.15	22049	0.40
10	44.15	16858	0.30
11	45.15	1371	0.02
12	46.60	1819	0.03
13	48.15	1198	0.02
14	49.15	16300	0.29
15	50.15	94131	1.69
16	51.15	204409	3.67
17	52.05	193401	3.47
18	53.05	41926	0.75
19	54.05	5383	0.10
20	54.95	6456	0.12
21	56.05	5033	0.09
22	57.00	15497	0.28
23	57.95	5680	0.10
24	58.95	961	0.02
25	60.15	1255	0.02
26	61.15	7554	0.14
27	62.15	42836	0.77
28	63.05	117793	2.11
29	64.05	46887	0.84
30	65.05	18462	0.33
31	66.05	3758	0.07
32	67.15	2256	0.04
33	68.15	24696	0.44
34	69.05	125854	2.26
35	70.05	10544	0.19
36	71.15	3463	0.06
37	72.15	1595	0.03
38	73.15	13400	0.24
39	74.15	100823	1.81
40	75.15	185563	3.33
41	76.15	142583	2.56
42	77.10	392346	7.04
43	78.05	96799	1.74
44	79.05	60985	1.09
45	80.05	18385	0.33
46	81.15	8690	0.16
47	82.15	13327	0.24
48	83.10	62736	1.12
49	84.05	9034	0.16
50	85.15	6099	0.11
51	86.15	19114	0.34
52	87.10	35511	0.64
53	88.05	16709	0.30
54	88.65	13026	0.23
55	89.65	21058	0.38
56	90.55	10891	0.20
57	91.55	7367	0.13
58	92.55	4649	0.08
59	93.65	6465	0.12
60	94.60	17517	0.31
61	95.55	12633	0.23
62	97.05	5235	0.09
63	98.05	28350	0.51
64	99.05	39513	0.71
65	100.05	54885	0.98
66	101.05	228661	4.10
67	101.95	53660	0.96
68	102.95	5501	0.10
69	103.95	732	0.01
70	105.05	1743	0.03
71	106.05	12082	0.22
72	107.05	11032	0.20
73	108.00	27151	0.49
74	108.95	12026	0.22
75	109.95	3223	0.06
76	111.05	4048	0.07
77	112.05	2829	0.05
78	113.05	10392	0.19
79	113.95	11854	0.21
80	115.00	15210	0.27
81	115.95	2572	0.05
82	116.75	2327	0.04
83	117.65	3544	0.06
84	118.90	1555	0.03
85	120.15	714	0.01
86	121.15	5200	0.09
87	122.15	17851	0.32
88	123.05	19814	0.36
89	124.15	8932	0.16
90	125.15	104414	1.87
91	126.15	983970	17.64
92	127.10	3690663	66.18
93	128.05	698696	12.53
94	129.05	20054	0.36
95	130.05	175	0.00
96	131.00	2315	0.04
97	132.95	4712	0.08
98	133.85	2020	0.04
99	134.90	481	0.01
100	136.95	3282	0.06
101	137.95	2646	0.05
102	138.95	6572	0.12
103	139.90	9460	0.17
104	140.85	3962	0.07
105	141.85	1423	0.03
106	142.90	451	0.01
107	143.55	860	0.02
108	144.55	1328	0.02
109	145.45	6472	0.12
110	146.20	2411	0.04
111	147.25	5925	0.11
112	148.25	31599	0.57
113	149.15	19355	0.35
114	150.15	2957	0.05
115	151.15	3976	0.07
116	152.15	11181	0.20
117	153.15	30442	0.55
118	154.15	664638	11.92
119	155.10	5576914	100.00
120	156.05	1076136	19.30
121	157.05	33773	0.61
122	158.05	1843	0.03
123	159.05	924	0.02
124	160.05	1483	0.03
125	161.05	18850	0.34
126	161.95	4215	0.08
127	162.55	3435	0.06
128	163.45	3989	0.07
129	164.45	2042	0.04
130	165.55	9131	0.16
131	166.45	4440	0.08
132	167.95	1094	0.02
133	169.00	2167	0.04
134	169.95	924	0.02
135	172.15	919	0.02
136	173.10	1902	0.03
137	174.20	129	0.00
138	175.25	6731	0.12
139	176.20	37727	0.68
140	177.15	9899	0.18
141	178.15	5750	0.10
142	179.15	1961	0.04
143	182.30	1135	0.02
144	183.15	1110	0.02
145	184.05	787	0.01
146	185.10	1449	0.03
147	187.15	2197	0.04
148	188.15	6635	0.12
149	189.10	18540	0.33
150	190.05	8114	0.15
151	191.05	3785	0.07
152	192.05	956	0.02
153	194.10	675	0.01
154	196.10	8	0.00
155	197.45	1048	0.02
156	198.45	828	0.01
157	200.15	989	0.02
158	201.15	1585	0.03
159	202.15	3862	0.07
160	203.15	5522	0.10
161	204.10	10528	0.19
162	205.05	5668	0.10
163	206.60	701	0.01
164	207.60	253	0.00
165	211.60	53	0.00
166	212.25	1200	0.02
167	213.15	1752	0.03
168	214.25	9481	0.17
169	215.25	8408	0.15
170	216.20	29243	0.52
171	217.15	17555	0.31
172	218.15	4669	0.08
173	219.60	836	0.01
174	221.60	166	0.00
175	222.30	568	0.01
176	223.25	1280	0.02
177	223.90	401	0.01
178	224.90	1046	0.02
179	227.20	1499	0.03
180	228.20	443	0.01
181	229.20	259	0.00
182	230.20	352	0.01
183	231.90	2597	0.05
184	232.50	1376	0.02
185	233.50	1446	0.03
186	234.45	1091	0.02
187	236.50	32	0.00
188	237.50	599	0.01
189	238.50	268	0.00
190	240.50	45	0.00
191	242.10	627	0.01
192	243.15	3075	0.06
193	244.05	19159	0.34
194	245.15	9937	0.18
195	246.15	62821	1.13
196	247.05	17597	0.32
197	248.05	602	0.01
198	251.10	599	0.01
199	252.10	648	0.01
200	253.10	467	0.01
201	256.10	81	0.00
202	257.10	678	0.01
203	258.10	840	0.02
204	260.10	542	0.01
205	262.00	6517	0.12
206	262.95	2792	0.05
207	264.00	550	0.01
208	265.00	373	0.01
209	266.00	158	0.00
210	268.00	340	0.01
211	269.00	33	0.00
212	270.00	110	0.00
213	272.10	1094	0.02
214	273.10	49	0.00
215	277.10	778	0.01
216	278.10	620	0.01
217	280.10	173	0.00
218	281.10	69	0.00
219	282.30	1226	0.02
220	283.30	768	0.01
221	284.30	126	0.00
222	285.30	74	0.00
223	286.30	86	0.00
224	287.30	514	0.01
225	288.30	94	0.00
226	289.30	325	0.01
227	290.30	589	0.01
228	292.30	227	0.00
229	296.30	544	0.01
230	297.30	832	0.01
231	298.30	40	0.00
232	301.20	2959	0.05
233	302.25	2039	0.04
234	303.15	977	0.02
235	303.90	1204	0.02
236	304.90	520	0.01
237	306.90	31	0.00
238	307.90	674	0.01
239	309.05	821	0.01
240	309.95	3321	0.06
241	311.15	739	0.01
242	312.05	5182	0.09
243	313.05	482	0.01
244	317.10	460	0.01
245	318.10	784	0.01
246	322.10	308	0.01
247	323.10	910	0.02
248	325.10	250	0.00
249	327.10	192	0.00
250	329.25	156	0.00
251	330.25	84199	1.51
252	331.15	635353	11.39
253	332.15	155572	2.79
254	333.15	8630	0.15
255	337.00	2829	0.05
256	338.00	454	0.01
257	342.00	900	0.02
258	343.00	501	0.01
259	344.00	24	0.00
260	345.00	323	0.01
261	348.00	30	0.00
262	352.00	150	0.00
263	353.00	25	0.00
264	357.00	484	0.01
265	358.00	671	0.01
266	362.00	513	0.01
267	363.00	735	0.01
268	365.00	920	0.02
269	367.00	402	0.01
270	372.00	367	0.01
271	376.00	254	0.00
272	377.00	550	0.01
273	378.00	485	0.01
274	379.00	85	0.00
275	382.00	663	0.01
276	383.50	958	0.02
277	384.50	46	0.00
278	385.50	47	0.00
279	387.50	651	0.01
280	391.50	106	0.00
281	392.50	29	0.00
282	396.50	362	0.01
283	397.50	564	0.01
284	398.50	266	0.00
285	400.50	144	0.00
286	402.50	890	0.02
287	403.50	422	0.01
288	404.50	250	0.00
289	407.50	450	0.01
290	411.50	404	0.01
291	416.50	317	0.01
292	417.50	744	0.01
293	418.50	258	0.00
294	421.50	426	0.01
295	422.50	471	0.01
296	423.50	421	0.01
297	424.50	421	0.01
298	426.50	114	0.00
299	427.50	2	0.00
300	431.50	250	0.00
301	436.50	51	0.00
302	437.50	909	0.02
303	441.50	257	0.00
304	442.50	962	0.02
305	443.50	63	0.00
306	444.50	471	0.01
307	447.50	90	0.00
308	451.50	416	0.01
309	456.50	635	0.01
310	457.50	630	0.01
311	461.50	505	0.01
312	462.50	846	0.02
313	463.50	197	0.00
314	464.50	327	0.01
315	467.50	306	0.01
316	468.50	2	0.00
317	472.50	29	0.00
318	476.50	317	0.01
319	477.50	775	0.01
320	481.50	722	0.01
321	482.50	646	0.01
322	484.50	330	0.01
323	486.50	49	0.00
324	487.50	170	0.00
325	491.50	184	0.00
326	492.50	42	0.00
327	496.50	596	0.01
328	497.50	436	0.01
329	501.50	832	0.01
330	502.50	425	0.01
331	503.50	232	0.00
332	504.50	122	0.00
333	507.50	150	0.00
334	511.50	38	0.00
335	512.50	116	0.00
336	516.50	493	0.01
337	517.50	612	0.01
338	521.50	355	0.01
339	522.50	770	0.01
340	526.50	197	0.00
341	527.50	91	0.00
342	531.50	226	0.00
343	536.50	609	0.01
344	537.50	325	0.01
345	541.50	706	0.01
346	542.50	536	0.01
347	543.50	36	0.00
348	544.50	414	0.01
349	546.50	6	0.00
350	547.50	207	0.00
351	551.50	286	0.01
352	556.50	640	0.01
353	557.50	389	0.01
354	561.50	465	0.01
355	562.50	986	0.02
356	563.50	6	0.00
357	564.50	360	0.01
358	566.50	250	0.00
359	571.50	186	0.00
360	575.50	106	0.00
361	576.50	295	0.01
362	577.50	675	0.01
363	581.50	117	0.00
364	582.50	717	0.01
365	583.50	59	0.00
366	584.50	289	0.01
367	586.50	193	0.00
368	591.50	344	0.01
369	596.50	601	0.01
370	597.50	484	0.01
371	601.50	481	0.01
372	602.50	672	0.01
373	603.50	201	0.00
374	604.50	192	0.00
375	606.50	210	0.00
376	611.50	17	0.00
377	615.50	104	0.00
378	616.50	769	0.01
379	617.50	420	0.01
380	621.50	296	0.01
381	622.50	759	0.01
382	624.50	134	0.00
383	626.50	98	0.00
384	631.50	107	0.00
385	636.50	928	0.02
386	637.50	112	0.00
387	641.50	711	0.01
388	642.50	294	0.01
389	643.50	420	0.01
390	646.50	258	0.00

Single Mass Analysis

Tolerance = 5.0 PPM / DBE: min = -50.0, max = 80.0
Element prediction: Off
Number of isotope peaks used for i-FIT = 6

Monoisotopic Mass, Even Electron Ions
130 formula(e) evaluated with 2 results within limits (up to 50 closest results for each mass)
Elements Used:
C: 0-20 H: 0-25 N: 0-2 O: 0-2 F: 0-8
190716_1827 17 (0.197) Cm (14:20-(30:72+2:8)x2.000)
1: TOF MS AP+

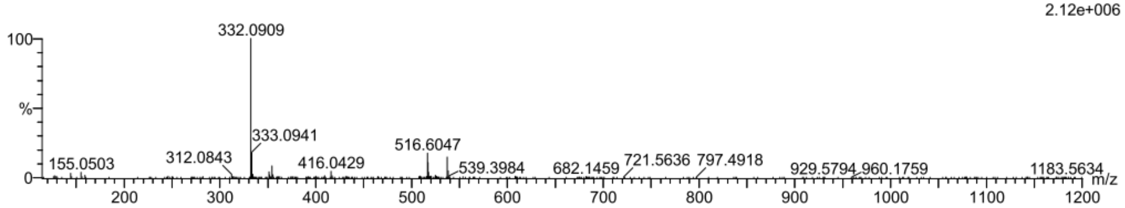

Minimum:				-50.0				
Maximum:		15.0	5.0	80.0				
Mass	Calc. Mass	mDa	PPM	DBE	i-FIT	Norm	Conf(%)	Formula
332.0909	332.0898	1.1	3.3	11.5	314.8	3.165	4.22	C18 H13 N O2 F3
	332.0897	1.2	3.6	0.5	311.7	0.043	95.78	C10 H14 N O2 F8

Sample Information

Sample Name	: M_Satenik
Sample ID	: IVA_1832
Analyzed	: 2019-07-22 12:24:38
Vial #	: 12
Injection Volume	: 1.00
$EndIf$Data File	: C:\GCMSsolution\Data\widma\2019\07_2019\M
Method File	: C:\GCMSsolution\System\Tune1\DI EI 1 metoda
Tuning File	: C:\GCMSsolution\System\Tune1\tuning05.07.20

Method

[Comment]

===== Analytical Line 1 =====

[GC-2010]

Column Oven Temp.	:30.0 °C
Injection Temp.	:150.00 °C
Injection Mode	:Split
Pressure	:12.8 kPa
Total Flow	:6.6 mL/min
Column Flow	:0.60 mL/min
Linear Velocity	:27.7 cm/sec
Purge Flow	:3.0 mL/min
Split Ratio	:5.0

[GCMS-QP2010 Ultra]

IonSourceTemp	:200.00 °C
Interface Temp.	:200.00 °C
Solvent Cut Time	:0.10 min
Detector Gain Mode	:Relative
Detector Gain	:1.23 kV +0.00 kV
Threshold	:0

[MS Table]

--Group 1 - Event 1--

Start Time	:0.10min
End Time	:25.00min
ACQ Mode	:Scan
Event Time	:0.10sec
Scan Speed	:20000
Start m/z	:35.00
End m/z	:1090.00

Chromatogram M_Satenik C:\GCMSsolution\Data\widma\2019\07_2019\M_Satenik_IVA_1832.QGD

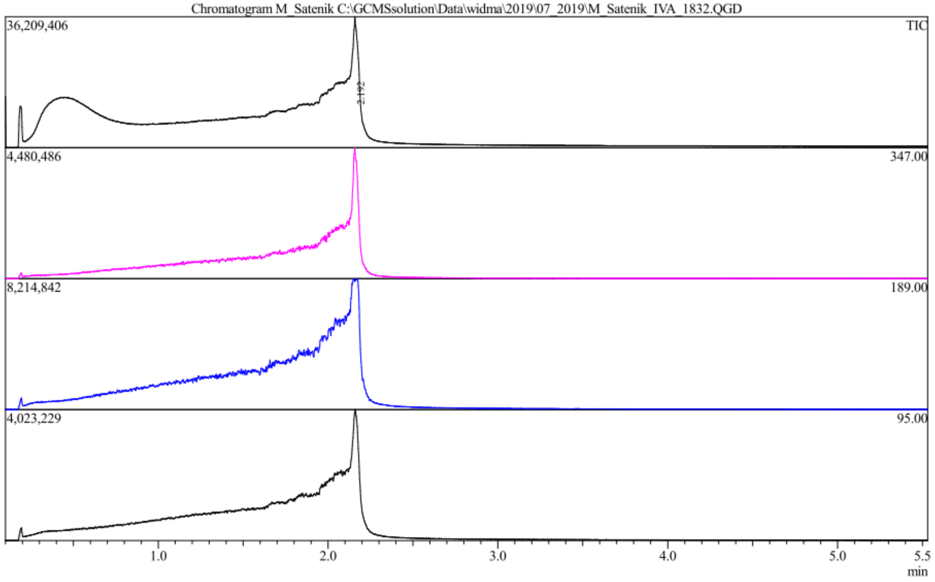

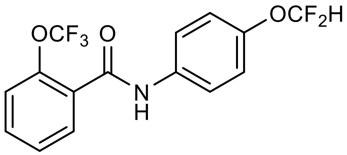

N-(4-(difluoromethoxy)phenyl)-2-(trifluoromethoxy)benzamide **4y**

Chemical Formula: $C_{15}H_{10}F_5NO_3$

Molecular Weight: 347.24

Compound **4y**

Spectrum

Line#:1 R.Time:2.192(Scan#:1256)
MassPeaks:694
RawMode:Single 2.192(1256) BasePeak:189(3516417)
BG Mode:None Group 1 - Event 1 Scan

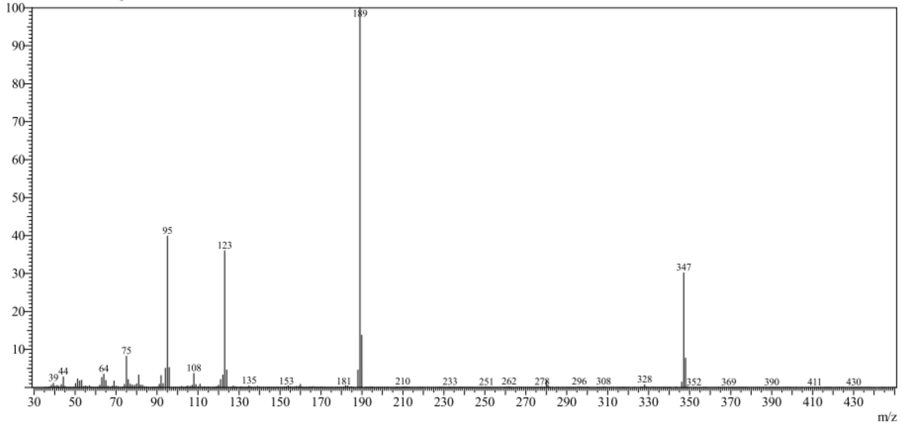

Mass Table
Line#:1 R.Time:2.192(Scan#:1256)
MassPeaks:694
RawMode:Single 2.192(1256) BasePeak:189(3516417)
BG Mode:None Group 1 - Event 1 Scan

#	m/z	Abs. Int.	Rel. Int.
1	35.35	966	0.03
2	36.35	2806	0.08
3	37.35	6249	0.18
4	38.35	20500	0.58
5	39.35	39194	1.11
6	40.25	12145	0.35
7	41.25	18053	0.51
8	42.15	9457	0.27
9	43.25	28276	0.80
10	44.20	98321	2.80
11	45.15	11182	0.32
12	46.20	267	0.01
13	47.25	1635	0.05
14	48.15	1018	0.03
15	49.25	3313	0.09
16	50.25	38512	1.10
17	51.15	79234	2.25
18	52.15	61396	1.75
19	53.15	67964	1.93
20	54.15	11439	0.33
21	55.15	15126	0.43
22	56.05	8354	0.24
23	57.10	16650	0.47
24	58.05	4915	0.14
25	59.05	1867	0.05
26	60.05	2872	0.08
27	61.05	4373	0.12
28	62.05	21055	0.60
29	63.05	92364	2.63
30	64.00	124617	3.54
31	64.95	64436	1.83
32	65.95	13229	0.38
33	67.05	5899	0.17
34	68.05	13643	0.39
35	69.00	61926	1.76
36	69.95	13605	0.39
37	70.95	8940	0.25
38	71.95	2352	0.07
39	73.05	5787	0.16
40	74.05	30313	0.86
41	75.00	290064	8.25
42	75.95	73822	2.10
43	76.95	31123	0.89
44	77.95	22625	0.64
45	79.05	21201	0.60
46	80.05	34435	0.98
47	81.00	114849	3.27
48	81.95	22082	0.63
49	82.95	20103	0.57
50	83.85	7418	0.21
51	84.85	5320	0.15
52	85.75	2660	0.08
53	87.05	1751	0.05
54	88.05	3417	0.10
55	89.05	4968	0.14
56	90.05	7337	0.21
57	91.05	30174	0.86
58	91.95	111321	3.17
59	92.95	36554	1.04
60	94.05	178686	5.08
61	95.00	1402413	39.88
62	95.95	183980	5.23
63	96.95	66	0.00
64	97.95	4412	0.13
65	99.05	2898	0.08
66	99.95	1584	0.05
67	101.05	4494	0.13
68	101.95	10334	0.29
69	103.00	3421	0.10
70	104.05	8699	0.25
71	104.95	14405	0.41
72	106.05	10896	0.31
73	107.05	21616	0.61
74	108.00	129823	3.69
75	108.95	28077	0.80
76	110.05	9203	0.26
77	110.95	30994	0.88
78	111.95	6243	0.18
79	112.95	5718	0.16
80	113.85	2955	0.08
81	114.85	5600	0.16
82	115.85	2746	0.08
83	117.05	2718	0.08
84	118.05	2478	0.07
85	119.05	9147	0.26
86	120.05	21301	0.61
87	121.05	73222	2.08
88	122.05	116269	3.31
89	123.00	1266039	36.00
90	123.95	162594	4.62
91	124.95	2178	0.06
92	126.25	4674	0.13
93	127.20	13586	0.39
94	128.15	9293	0.26
95	129.15	3388	0.10
96	130.15	1557	0.04
97	131.10	5262	0.15
98	132.05	2888	0.08
99	133.05	2712	0.08
100	134.05	4884	0.14
101	134.95	14437	0.41
102	135.95	3460	0.10
103	137.05	1735	0.05
104	138.05	3059	0.09
105	138.95	11269	0.32
106	139.95	5512	0.16
107	140.95	2713	0.08
108	141.95	2283	0.06
109	143.20	2857	0.08
110	144.15	1345	0.04
111	144.95	2004	0.06
112	146.00	1764	0.05
113	146.95	2921	0.08
114	148.15	1263	0.04
115	149.05	4077	0.12
116	150.15	2518	0.07
117	151.15	2991	0.09
118	152.15	6009	0.17
119	153.15	7568	0.22
120	154.05	14918	0.42
121	155.05	7489	0.21
122	156.05	3712	0.11
123	157.05	5458	0.16
124	157.95	11480	0.33
125	159.05	10657	0.30
126	159.95	29442	0.84
127	160.95	3948	0.11
128	161.95	1056	0.03
129	163.05	1281	0.04
130	164.10	3920	0.11
131	165.05	6926	0.20
132	166.10	8358	0.24
133	167.05	7265	0.21
134	168.00	3781	0.11
135	168.95	4638	0.13
136	170.00	5284	0.15
137	170.95	3521	0.10
138	171.95	3238	0.09
139	173.00	595	0.02
140	173.80	702	0.02
141	174.85	1346	0.04
142	175.80	575	0.02
143	177.15	1071	0.03
144	178.05	1896	0.05
145	179.05	2266	0.06
146	180.15	2468	0.07
147	181.15	5606	0.16
148	182.10	16133	0.46
149	183.05	13788	0.39
150	184.05	5506	0.16
151	184.90	5486	0.16
152	185.85	1246	0.04
153	188.05	161265	4.59
154	189.00	3516417	100.00
155	189.95	485494	13.81
156	191.95	3763	0.11
157	192.95	3049	0.09
158	194.05	6435	0.18
159	195.00	6004	0.17
160	195.95	1911	0.05
161	197.15	953	0.03
162	198.15	2502	0.07
163	199.05	1009	0.03
164	199.95	1805	0.05
165	200.95	1138	0.03
166	201.90	2565	0.07
167	202.85	1168	0.03
168	203.90	502	0.01
169	204.90	721	0.02
170	205.85	758	0.02
171	206.85	3159	0.09
172	207.75	1897	0.05
173	208.85	1523	0.04
174	209.85	5844	0.17
175	210.75	5972	0.17
176	211.75	6475	0.18
177	212.75	3530	0.10
178	213.75	1402	0.04
179	214.80	295	0.01
180	215.80	397	0.01
181	217.10	1056	0.03
182	218.05	946	0.03
183	218.70	633	0.02
184	219.75	1073	0.03
185	220.70	526	0.01
186	222.10	1647	0.05
187	223.10	693	0.02
188	224.10	549	0.02
189	225.10	185	0.01
190	226.10	801	0.02
191	227.25	1266	0.04
192	228.25	2061	0.06
193	229.15	1317	0.04
194	230.15	1224	0.03
195	231.05	856	0.02
196	232.05	2139	0.06
197	233.00	4082	0.12
198	233.95	2766	0.08
199	234.95	1192	0.03
200	235.60	528	0.02
201	236.55	1023	0.03
202	237.60	1569	0.04
203	238.60	1383	0.04
204	239.65	1011	0.03
205	240.60	574	0.02
206	241.60	710	0.02
207	242.60	106	0.00
208	243.20	303	0.01
209	244.15	1318	0.04
210	245.20	588	0.02
211	246.20	297	0.01
212	247.20	93	0.00
213	248.20	584	0.02
214	248.80	506	0.01
215	249.85	1768	0.05
216	250.80	2169	0.06
217	251.75	1132	0.03
218	252.80	662	0.02
219	253.80	247	0.01
220	254.80	374	0.01
221	255.80	538	0.02
222	256.80	469	0.01
223	257.80	327	0.01
224	258.80	137	0.00
225	259.85	2069	0.06
226	260.85	4187	0.12
227	261.80	5677	0.16
228	262.75	1284	0.04
229	263.80	655	0.02
230	265.15	985	0.03
231	266.05	1664	0.05
232	267.15	3157	0.09
233	268.05	1369	0.04
234	269.10	181	0.01
235	270.10	360	0.01
236	271.10	383	0.01
237	272.10	477	0.01
238	273.10	233	0.01
239	274.10	59	0.00
240	275.10	117	0.00
241	276.05	1113	0.03
242	277.05	1939	0.06
243	277.95	3414	0.10
244	279.05	2899	0.08
245	280.05	66660	1.90
246	280.95	17129	0.49
247	281.95	1295	0.04
248	283.00	328	0.01
249	285.00	344	0.01
250	286.00	460	0.01
251	288.00	491	0.01
252	290.00	434	0.01
253	291.00	84	0.00
254	292.00	462	0.01
255	293.00	184	0.01
256	294.00	512	0.01
257	295.15	1172	0.03
258	296.05	7173	0.20
259	297.05	2021	0.06
260	297.95	2097	0.06
261	299.00	1053	0.03
262	300.00	3170	0.09
263	300.95	850	0.02
264	305.00	135	0.00
265	306.00	51	0.00
266	307.00	112	0.00
267	308.00	5695	0.16
268	308.95	2005	0.06
269	310.00	368	0.01
270	311.00	503	0.01
271	312.00	899	0.03
272	313.00	627	0.02
273	314.00	194	0.01
274	315.00	144	0.00
275	316.00	141	0.00
276	317.00	443	0.01
277	317.95	2330	0.07
278	319.00	349	0.01
279	320.00	193	0.01
280	323.00	374	0.01
281	326.05	3636	0.10
282	327.05	2608	0.07
283	328.00	25460	0.72
284	328.95	6635	0.19
285	329.95	638	0.02
286	331.35	1443	0.04
287	332.30	197	0.01
288	333.30	396	0.01
289	334.30	436	0.01
290	335.30	87	0.00
291	336.30	132	0.00
292	337.30	228	0.01
293	338.30	28	0.00
294	339.30	39	0.00
295	340.30	36	0.00
296	343.30	285	0.01
297	344.00	242	0.01
298	346.05	49599	1.41
299	347.05	1063004	30.23
300	347.95	272113	7.74
301	348.95	8532	0.24
302	349.95	415	0.01
303	352.00	333	0.01
304	353.00	166	0.00
305	355.00	202	0.01
306	357.00	277	0.01
307	358.00	177	0.01
308	364.00	104	0.00
309	365.00	11	0.00
310	366.00	36	0.00
311	367.00	4	0.00
312	368.00	212	0.01
313	369.00	581	0.02
314	370.00	178	0.01
315	371.00	332	0.01
316	372.00	341	0.01
317	373.00	153	0.00
318	374.00	111	0.00
319	375.00	195	0.01
320	376.00	363	0.01
321	377.00	377	0.01
322	378.00	241	0.01
323	383.00	32	0.00
324	384.00	237	0.01
325	386.00	113	0.00
326	387.00	38	0.00
327	389.00	256	0.01
328	390.00	544	0.02
329	391.00	245	0.01
330	392.00	428	0.01
331	393.00	81	0.00
332	394.00	11	0.00
333	396.00	105	0.00
334	397.00	137	0.00
335	398.00	276	0.01
336	399.00	99	0.00
337	402.00	131	0.00
338	405.00	6	0.00
339	408.00	17	0.00
340	409.00	57	0.00
341	410.00	237	0.01
342	411.00	315	0.01
343	412.00	174	0.00
344	413.00	102	0.00
345	414.00	74	0.00
346	416.00	143	0.00
347	417.00	251	0.01
348	418.00	26	0.00
349	419.00	24	0.00
350	422.00	19	0.00
351	423.00	117	0.00
352	424.00	17	0.00
353	425.00	50	0.00
354	429.00	233	0.01
355	430.00	240	0.01
356	431.00	212	0.01
357	432.00	194	0.01
358	433.00	70	0.00
359	434.00	256	0.01
360	436.00	237	0.01
361	437.00	116	0.00
362	438.00	143	0.00
363	444.00	51	0.00
364	445.00	100	0.00
365	447.00	117	0.00
366	449.00	138	0.00
367	450.00	333	0.01
368	451.00	204	0.01
369	452.00	244	0.01
370	453.00	184	0.01
371	455.00	10	0.00
372	456.00	150	0.00
373	458.00	21	0.00
374	462.00	39	0.00
375	464.00	109	0.00
376	469.00	108	0.00
377	470.00	149	0.00
378	471.00	101	0.00
379	472.00	265	0.01
380	473.00	24	0.00
381	474.00	200	0.01
382	476.00	77	0.00
383	477.00	141	0.00
384	478.00	114	0.00
385	484.00	3	0.00
386	489.00	242	0.01
387	490.00	176	0.01
388	491.00	236	0.01
389	492.00	205	0.01
390	493.00	275	0.01
391	495.00	5	0.00
392	497.00	60	0.00
393	498.00	34	0.00

Single Mass Analysis
Tolerance = 5.0 PPM / DBE: min = -50.0, max = 80.0
Element prediction: Off
Number of isotope peaks used for i-FIT = 6

Monoisotopic Mass, Even Electron Ions
136 formula(e) evaluated with 4 results within limits (up to 50 closest results for each mass)
Elements Used:
C: 0-20 H: 0-15 N: 0-2 O: 0-4 F: 0-8
190716_1832 16 (0.177) Cm (15:19-(31:72+2:9)x2.000)
1: TOF MS AP+

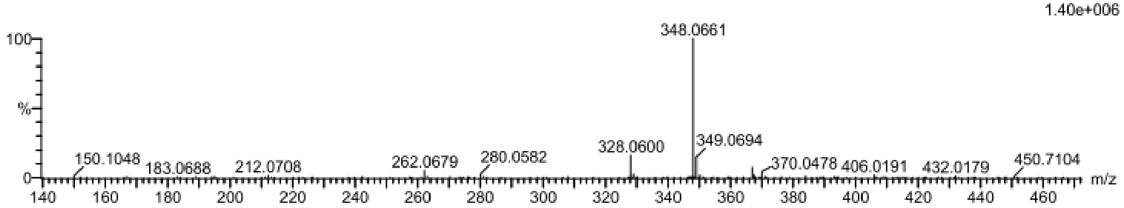

Minimum:				-50.0				
Maximum:		15.0	5.0	80.0				
Mass	Calc. Mass	mDa	PPM	DBE	i-FIT	Norm	Conf(%)	Formula
348.0661	348.0659	0.2	0.6	8.5	132.1	2.534	7.93	C15 H11 N O3 F5
	348.0671	-1.0	-2.9	4.5	129.6	0.083	92.06	C12 H12 N O4 F6
	348.0672	-1.1	-3.2	15.5	142.9	13.377	0.00	C20 H11 N O4 F
	348.0648	1.3	3.7	12.5	138.9	9.392	0.01	C18 H10 N O2 F4

Sample Information

Sample Name : M_Satenik
Sample ID : IVA_1849
Analyzed : 2019-07-18 08:21:19

Vial # : 12
Injection Volume : 1.00
$EndIf$Data File : C:\GCMSsolution\Data\widma\2019\07_2019\M
Method File : C:\GCMSsolution\System\Tune1\DI EI 1 metoda
Tuning File : C:\GCMSsolution\System\Tune1\tuning05.07.20

Method

[Comment]

===== Analytical Line 1 =====

[GC-2010]
Column Oven Temp. :30.0 °C
Injection Temp. :150.00 °C
Injection Mode :Split
Pressure :12.8 kPa
Total Flow :6.6 mL/min
Column Flow :0.60 mL/min
Linear Velocity :27.7 cm/sec
Purge Flow :3.0 mL/min
Split Ratio :5.0

[GCMS-QP2010 Ultra]
IonSourceTemp :200.00 °C
Interface Temp. :200.00 °C
Solvent Cut Time :0.10 min
Detector Gain Mode :Relative
Detector Gain :1.23 kV +0.00 kV
Threshold :0

[MS Table]
--Group 1 - Event 1--
Start Time :0.10min
End Time :25.00min
ACQ Mode :Scan
Event Time :0.10sec
Scan Speed :20000
Start m/z :35.00
End m/z :1090.00

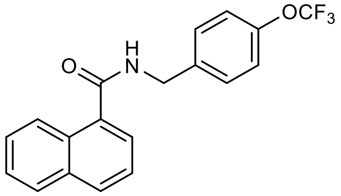

N-(4-(trifluoromethoxy)benzyl)-1-naphthamide **4z**

Chemical Formula: $C_{19}H_{14}F_3NO_2$
Molecular Weight: 345.32

Compound **4z**

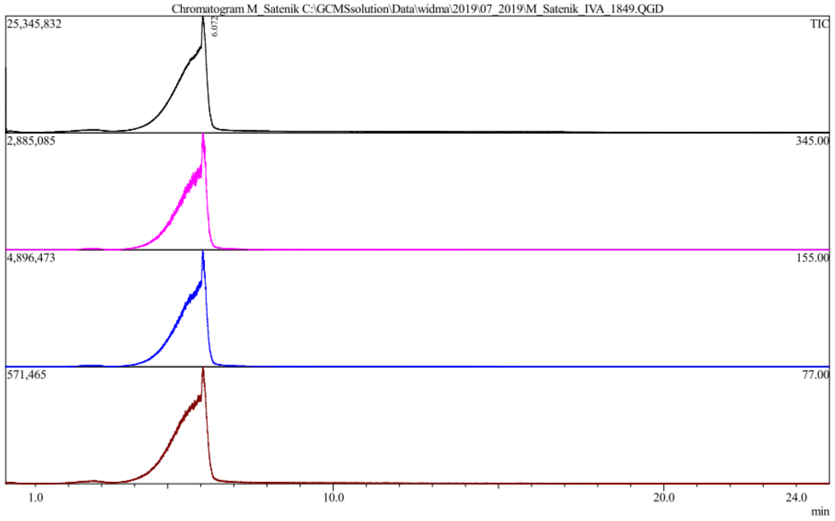

Spectrum

Line#:1 R.Time:6.072(Scan#:3584)
MassPeaks:711
RawMode:Single 6.072(3584) BasePeak:155(4460908)
BG Mode:None Group 1 - Event 1 Scan

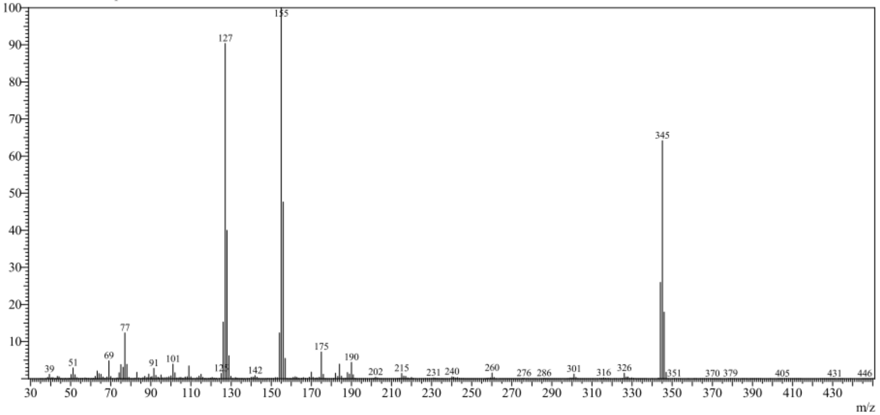

Mass Table
Line#:1 R.Time:6.072(Scan#:3584)
MassPeaks:711
RawMode:Single 6.072(3584) BasePeak:155(4460908)
BG Mode:None Group 1 - Event 1 Scan

#	m/z	Abs. Int.	Rel. Int.
1	35.00	332	0.01
2	36.00	236	0.01
3	37.45	2458	0.06
4	38.45	16305	0.37
5	39.35	54215	1.22
6	40.35	13574	0.30
7	41.25	14171	0.32
8	42.35	6403	0.14
9	43.35	29059	0.65
10	44.25	25291	0.57
11	45.25	3732	0.08
12	46.25	819	0.02
13	47.30	2257	0.05
14	48.20	372	0.01
15	49.25	4690	0.11
16	50.25	51104	1.15
17	51.20	129213	2.90
18	52.15	46963	1.05
19	53.15	12807	0.29
20	54.15	3159	0.07
21	55.15	6448	0.14
22	56.10	4492	0.10
23	57.15	10832	0.24
24	58.05	6108	0.14
25	59.05	6638	0.15
26	60.25	1382	0.03
27	61.25	6475	0.15
28	62.25	28936	0.65
29	63.20	93998	2.11
30	64.15	61694	1.38
31	65.15	52391	1.17
32	66.15	22604	0.51
33	67.15	8743	0.20
34	68.05	20203	0.45
35	69.05	215373	4.83
36	69.95	25975	0.58
37	71.00	3782	0.08
38	72.15	1156	0.03
39	73.15	10036	0.22
40	74.15	71204	1.60
41	75.10	170430	3.82
42	76.15	137855	3.09
43	77.05	552289	12.38
44	78.05	171782	3.85
45	79.05	16829	0.38
46	80.05	2013	0.05
47	81.05	2438	0.05
48	82.05	7475	0.17
49	83.00	77362	1.73
50	83.95	11712	0.26
51	85.05	4290	0.10
52	86.05	13726	0.31
53	87.00	28991	0.65
54	88.05	17302	0.39
55	89.00	57137	1.28
56	89.95	17424	0.39
57	90.55	27125	0.61
58	91.55	124919	2.80
59	92.45	41258	0.92
60	93.45	18281	0.41
61	94.05	9959	0.22
62	95.00	44170	0.99
63	95.95	13825	0.31
64	97.05	12008	0.27
65	98.05	15170	0.34
66	99.05	23061	0.52
67	100.05	34017	0.76
68	101.05	174854	3.92
69	101.95	71178	1.60
70	102.95	9413	0.21
71	104.05	13617	0.31
72	104.95	16196	0.36
73	105.95	9083	0.20
74	107.05	20608	0.46
75	108.05	23192	0.52
76	109.00	155523	3.49
77	109.95	25114	0.56
78	110.95	3328	0.07
79	112.05	2377	0.05
80	113.05	14384	0.32
81	114.05	30669	0.69
82	115.00	54042	1.21
83	115.95	14938	0.33
84	116.95	2827	0.06
85	117.60	1001	0.02
86	118.65	3813	0.09
87	119.65	7641	0.17
88	120.60	15417	0.35
89	121.55	8097	0.18
90	122.55	5225	0.12
91	124.05	3607	0.08
92	125.05	65236	1.46
93	126.05	682875	15.31
94	127.05	4031318	90.37
95	127.95	1784860	40.01
96	128.95	276516	6.20
97	129.95	28097	0.63
98	130.95	3603	0.08
99	132.10	13080	0.29
100	133.05	7005	0.16
101	134.05	2826	0.06
102	135.05	1535	0.03
103	135.70	421	0.01
104	136.65	1297	0.03
105	138.05	2156	0.05
106	139.05	6360	0.14
107	140.05	18303	0.41
108	141.05	20758	0.47
109	141.95	36382	0.82
110	142.95	18747	0.42
111	143.95	1963	0.04
112	145.05	1154	0.03
113	146.05	1187	0.03
114	147.15	858	0.02
115	148.15	2413	0.05
116	149.05	1852	0.04
117	150.15	2187	0.05
118	151.15	3566	0.08
119	152.15	14207	0.32
120	153.15	14465	0.32
121	154.15	550575	12.34
122	155.05	4460908	100.00
123	156.05	2124591	47.63
124	157.05	244645	5.48
125	158.05	6362	0.14
126	159.05	1484	0.03
127	159.95	1357	0.03
128	160.95	14743	0.33
129	161.95	24828	0.56
130	162.85	16778	0.38
131	163.85	4047	0.09
132	165.15	4904	0.11
133	166.05	12217	0.27
134	167.05	4044	0.09
135	168.05	1798	0.04
136	169.05	17592	0.39
137	170.00	79311	1.78
138	170.95	15019	0.34
139	172.05	7597	0.17
140	172.95	11092	0.25
141	174.05	19341	0.43
142	175.00	323042	7.24
143	175.95	53320	1.20
144	176.95	1632	0.04
145	177.95	1282	0.03
146	178.95	786	0.02
147	180.05	1041	0.02
148	181.15	4451	0.10
149	182.10	66598	1.49
150	183.15	28352	0.64
151	184.10	174617	3.91
152	185.05	31047	0.70
153	186.05	1371	0.03
154	187.05	8018	0.18
155	188.05	74930	1.68
156	189.05	55883	1.25
157	190.05	196548	4.41
158	190.95	46779	1.05
159	191.95	4299	0.10
160	192.95	1008	0.02
161	194.00	156	0.00
162	195.00	680	0.02
163	196.00	461	0.01
164	197.00	800	0.02
165	198.00	414	0.01
166	199.15	910	0.02
167	200.15	3229	0.07
168	201.15	5951	0.13
169	202.10	17389	0.39
170	203.05	9730	0.22
171	204.05	2798	0.06
172	205.10	1806	0.04
173	206.10	71	0.00
174	207.10	414	0.01
175	208.10	545	0.01
176	209.10	263	0.01
177	210.10	150	0.00
178	211.10	490	0.01
179	212.15	1135	0.03
180	213.15	4013	0.09
181	214.15	7193	0.16
182	215.10	63466	1.42
183	216.05	33098	0.74
184	217.05	29220	0.66
185	218.05	9689	0.22
186	219.00	3156	0.07
187	220.00	15470	0.35
188	220.95	4860	0.11
189	222.00	403	0.01
190	224.00	216	0.00
191	225.00	596	0.01
192	225.90	492	0.01
193	226.95	1531	0.03
194	227.95	2820	0.06
195	228.85	2056	0.05
196	229.95	5907	0.13
197	230.95	11073	0.25
198	231.85	7266	0.16
199	232.85	4194	0.09
200	233.85	2204	0.05
201	234.95	1581	0.04
202	235.90	618	0.01
203	236.90	117	0.00
204	237.90	20	0.00
205	239.15	2539	0.06
206	240.15	23274	0.52
207	241.05	17716	0.40
208	242.05	10316	0.23
209	242.95	14021	0.31
210	243.95	4508	0.10
211	244.95	2476	0.06
212	245.95	1892	0.04
213	246.85	1007	0.02
214	247.90	411	0.01
215	248.90	709	0.02
216	249.90	780	0.02
217	250.90	514	0.01
218	251.90	712	0.02
219	252.90	318	0.01
220	253.90	615	0.01
221	254.90	332	0.01
222	256.25	1104	0.02
223	257.25	1584	0.04
224	258.15	6721	0.15
225	259.25	10409	0.23
226	260.15	68017	1.52
227	261.15	16233	0.36
228	262.15	1222	0.03
229	264.20	151	0.00
230	265.20	603	0.01
231	266.20	337	0.01
232	267.20	255	0.01
233	268.20	173	0.00
234	269.20	194	0.00
235	270.20	115	0.00
236	271.20	278	0.01
237	272.20	291	0.01
238	273.20	80	0.00
239	274.10	584	0.01
240	275.15	1782	0.04
241	276.05	6036	0.14
242	277.05	821	0.02
243	278.10	2206	0.05
244	279.10	472	0.01
245	280.10	187	0.00
246	281.10	600	0.01
247	282.10	63	0.00
248	284.10	7	0.00
249	285.10	296	0.01
250	286.15	5093	0.11
251	287.05	2561	0.06
252	288.05	1580	0.04
253	289.05	842	0.02
254	290.10	596	0.01
255	291.10	334	0.01
256	292.10	335	0.01
257	293.10	275	0.01
258	294.10	293	0.01
259	295.10	134	0.00
260	296.10	122	0.00
261	297.15	1295	0.03
262	298.15	1435	0.03
263	299.15	10425	0.23
264	300.15	10243	0.23
265	301.05	56278	1.26
266	302.05	15481	0.35
267	303.05	1579	0.04
268	304.10	200	0.00
269	305.10	194	0.00
270	306.10	512	0.01
271	307.10	613	0.01
272	308.10	272	0.01
273	309.10	84	0.00
274	310.10	654	0.01
275	310.90	428	0.01
276	311.90	1013	0.02
277	312.90	62	0.00
278	313.95	4203	0.09
279	314.95	5225	0.12
280	315.85	9992	0.22
281	316.85	4903	0.11
282	318.05	6133	0.14
283	319.05	996	0.02
284	321.10	45	0.00
285	322.10	26	0.00
286	324.15	768	0.02
287	325.15	5443	0.12
288	326.10	65663	1.47
289	327.05	20803	0.47
290	327.95	20309	0.46
291	328.95	6426	0.14
292	329.85	9357	0.21
293	330.85	4690	0.11
294	331.85	932	0.02
295	332.90	396	0.01
296	333.90	224	0.01
297	334.90	206	0.00
298	335.90	21	0.00
299	336.90	28	0.00
300	339.90	41	0.00
301	343.05	7498	0.17
302	344.05	1157356	25.94
303	345.05	2862717	64.17
304	345.95	802489	17.99
305	346.95	76608	1.72
306	347.95	848	0.02
307	350.05	590	0.01
308	351.05	3656	0.08
309	351.95	1083	0.02
310	353.00	452	0.01
311	354.00	301	0.01
312	358.00	117	0.00
313	361.00	30	0.00
314	362.00	16	0.00
315	364.00	70	0.00
316	365.00	129	0.00
317	366.00	146	0.00
318	367.00	118	0.00
319	368.00	121	0.00
320	369.00	186	0.00
321	370.00	296	0.01
322	371.00	225	0.01
323	372.00	253	0.01
324	373.00	289	0.01
325	374.00	4	0.00
326	378.00	616	0.01
327	379.00	1861	0.04
328	380.00	437	0.01
329	381.00	343	0.01
330	385.00	142	0.00
331	386.00	139	0.00
332	387.00	154	0.00
333	389.00	105	0.00
334	390.00	364	0.01
335	391.00	214	0.00
336	392.00	232	0.01
337	393.00	177	0.00
338	394.00	126	0.00
339	399.00	48	0.00
340	403.00	8	0.00
341	404.00	1	0.00
342	405.00	256	0.01
343	406.00	147	0.00
344	407.00	245	0.01
345	408.00	197	0.00
346	409.00	165	0.00
347	410.00	195	0.00
348	411.00	184	0.00
349	412.00	269	0.01
350	413.00	84	0.00
351	414.00	182	0.00
352	424.00	245	0.01
353	425.00	73	0.00
354	426.00	207	0.00
355	427.00	109	0.00
356	428.00	124	0.00
357	429.00	207	0.00
358	430.00	36	0.00
359	431.00	446	0.01
360	433.00	196	0.00
361	434.00	37	0.00
362	435.00	37	0.00
363	439.00	64	0.00
364	445.00	5	0.00
365	446.00	265	0.01
366	447.00	28	0.00
367	448.00	7	0.00
368	449.00	17	0.00
369	450.00	206	0.00
370	451.00	222	0.00
371	452.00	198	0.00
372	453.00	352	0.01
373	465.00	168	0.00
374	466.00	212	0.00
375	467.00	24	0.00
376	468.00	189	0.00
377	469.00	118	0.00
378	470.00	139	0.00
379	471.00	267	0.01
380	472.00	303	0.01
381	473.00	262	0.01
382	474.00	43	0.00
383	477.00	113	0.00
384	485.00	155	0.00
385	486.00	214	0.00
386	487.00	66	0.00
387	488.00	160	0.00
388	489.00	63	0.00
389	490.00	244	0.01
390	491.00	201	0.00
391	492.00	272	0.01
392	493.00	48	0.00
393	494.00	131	0.00

Elemental Composition Report

Single Mass Analysis

Tolerance = 5.0 PPM / DBE: min = -50.0, max = 80.0
Element prediction: Off
Number of isotope peaks used for i-FIT = 6

Monoisotopic Mass, Even Electron Ions
234 formula(e) evaluated with 2 results within limits (up to 50 closest results for each mass)
Elements Used:
C: 0-25 H: 0-30 N: 0-5 O: 0-5 F: 0-10
190708_1849 16 (0.177) Cm (11:42-(49:72+2:8)x2.000)
1: TOF MS AP+

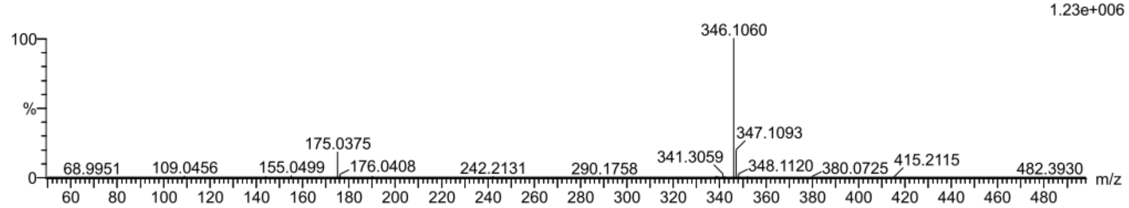

Minimum:				-50.0				
Maximum:		15.0	5.0	80.0				
Mass	Calc. Mass	mDa	PPM	DBE	i-FIT	Norm	Conf(%)	Formula
346.1060	346.1055	0.5	1.4	11.5	5389.7	0.001	99.90	C19 H15 N O2 F3
	346.1043	1.7	4.9	15.5	5396.6	6.860	0.10	C22 H14 N O F2

Method

[Comment]

===== Analytical Line 1 =====

[GC-2010]

Column Oven Temp.	:30.0 °C
Injection Temp.	:150.00 °C
Injection Mode	:Split
Pressure	:12.8 kPa
Total Flow	:6.6 mL/min
Column Flow	:0.60 mL/min
Linear Velocity	:27.9 cm/sec
Purge Flow	:3.0 mL/min
Split Ratio	:5.0

[GCMS-QP2010 Ultra]

IonSourceTemp	:200.00 °C
Interface Temp.	:200.00 °C
Solvent Cut Time	:0.10 min
Detector Gain Mode	:Relative
Detector Gain	:1.35 kV +0.00 kV
Threshold	:0

[MS Table]

--Group 1 - Event 1--

Start Time	:0.10min
End Time	:25.00min
ACQ Mode	:Scan
Event Time	:0.10sec
Scan Speed	:20000
Start m/z	:35.00
End m/z	:1090.00

Sample Information

Sample Name	: M.Satenik
Sample ID	: IVA_1863
Analyzed	: 2019-05-13 11:20:19
Vial #	: 1
Injection Volume	: 0.10
$EndIf$Data File	: C:\GCMSsolution\Data\widma\2019\05_2019\M
Method File	: C:\GCMSsolution\System\Tune1\DI EI 1 metoda
Tuning File	: C:\GCMSsolution\System\Tune1\tuning06.05.20

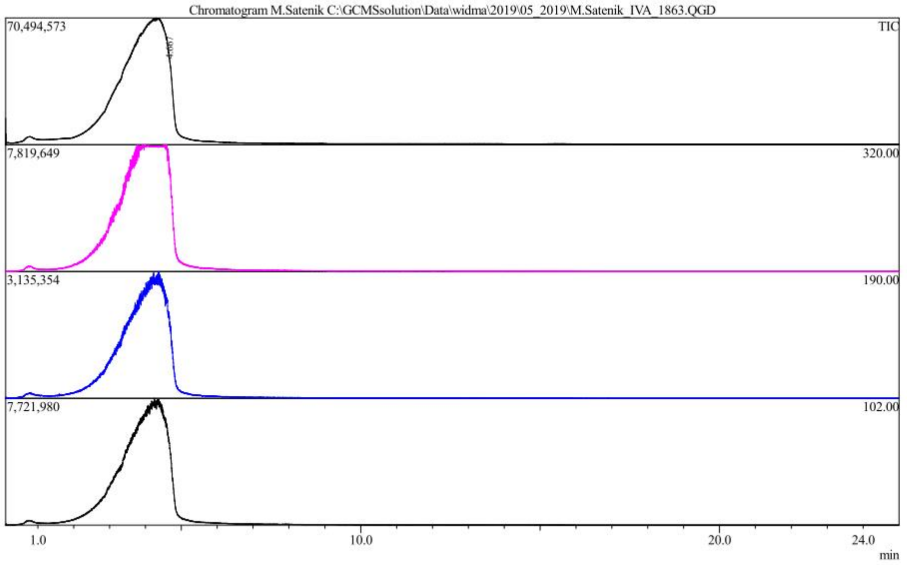

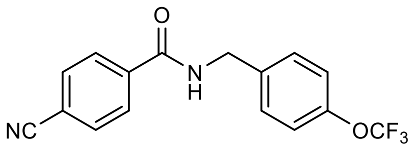

4-cyano-*N*-(4-(trifluoromethoxy)benzyl)benzamide **4aa**

Chemical Formula: $C_{16}H_{11}F_3N_2O_2$

Molecular Weight: 320.27

Compound **4aa**

Spectrum

Line#:1 R.Time:4.667(Scan#:2741)
MassPeaks:612
RawMode:Single 4.667(2741) BasePeak:130(8182651)
BG Mode:None Group 1 - Event 1 Scan

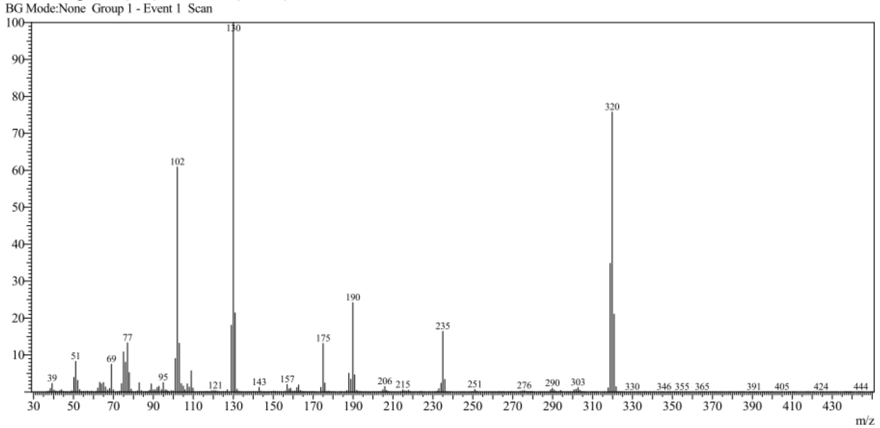

Mass Table
Line#:1 R.Time:4.667(Scan#:2741)
MassPeaks:612
RawMode:Single 4.667(2741) BasePeak:130(8182651)
BG Mode:None Group 1 - Event 1 Scan

#	m/z	Abs. Int.	Rel. Int.
1	36.35	2225	0.03
2	37.35	20263	0.25
3	38.35	78344	0.96
4	39.30	192620	2.35
5	40.25	42264	0.52
6	41.25	20614	0.25
7	42.10	10296	0.13
8	43.15	28389	0.35
9	44.05	50277	0.61
10	45.05	16279	0.20
11	46.05	7129	0.09
12	47.05	4275	0.05
13	48.15	1188	0.01
14	49.15	28546	0.35
15	50.15	320300	3.91
16	51.10	679653	8.31
17	52.05	257414	3.15
18	53.05	53363	0.65
19	54.05	13160	0.16
20	55.05	15228	0.19
21	56.15	15920	0.19
22	57.10	21479	0.26
23	58.00	9007	0.11
24	59.05	17553	0.21
25	59.95	3788	0.05
26	61.15	14449	0.18
27	62.15	87857	1.07
28	63.15	217376	2.66
29	64.05	182792	2.23
30	65.05	208389	2.55
31	66.05	110302	1.35
32	67.05	43782	0.54
33	68.05	75773	0.93
34	69.00	615881	7.53
35	69.95	48718	0.60
36	71.05	674	0.01
37	72.05	4067	0.05
38	73.05	21504	0.26
39	74.05	186504	2.28
40	75.05	889316	10.87
41	76.05	658891	8.05
42	77.00	1090134	13.32
43	77.95	430396	5.26
44	78.95	65109	0.80
45	79.95	7149	0.09

#	m/z	Abs. Int.	Rel. Int.
46	81.05	6900	0.08
47	82.05	24079	0.29
48	82.95	205919	2.52
49	83.95	31267	0.38
50	85.05	5245	0.06
51	86.05	9957	0.12
52	87.05	11357	0.14
53	88.05	37127	0.45
54	88.95	179561	2.19
55	89.95	50385	0.62
56	90.95	49699	0.61
57	91.95	103628	1.27
58	92.85	126164	1.54
59	94.05	48257	0.59
60	94.95	210612	2.57
61	95.95	49804	0.61
62	96.85	39926	0.49
63	97.75	12286	0.15
64	99.05	29649	0.36
65	100.05	25018	0.31
66	101.05	743796	9.09
67	101.95	4984025	60.91
68	102.95	1081012	13.21
69	103.95	187567	2.29
70	104.95	131497	1.61
71	105.90	49205	0.60
72	106.95	179206	2.19
73	107.95	109464	1.34
74	108.95	471195	5.76
75	109.85	88434	1.08
76	110.85	2970	0.04
77	112.05	6323	0.08
78	113.05	9066	0.11
79	113.95	10220	0.12
80	115.05	8501	0.10
81	116.05	11300	0.14
82	116.95	15304	0.19
83	117.95	11979	0.15
84	119.05	24976	0.31
85	120.05	27779	0.34
86	121.00	31378	0.38
87	121.95	18503	0.23
88	122.95	4948	0.06
89	123.95	5975	0.07
90	125.05	8126	0.10

#	m/z	Abs. Int.	Rel. Int.
91	126.05	10495	0.13
92	127.05	46440	0.57
93	129.05	1479945	18.09
94	130.05	8182651	100.00
95	130.95	1752284	21.41
96	131.95	65554	0.80
97	132.95	16537	0.20
98	133.95	2452	0.03
99	135.00	1266	0.02
100	135.90	78	0.00
101	136.85	2204	0.03
102	137.95	2749	0.03
103	138.90	5067	0.06
104	139.95	8121	0.10
105	140.85	4608	0.06
106	141.95	11646	0.14
107	142.95	101451	1.24
108	143.85	18691	0.23
109	145.15	6483	0.08
110	146.05	1283	0.02
111	147.15	1551	0.02
112	148.15	3206	0.04
113	149.15	8768	0.11
114	150.10	19872	0.24
115	151.05	13835	0.17
116	152.05	11226	0.14
117	153.05	4308	0.05
118	154.05	2540	0.03
119	155.05	2254	0.03
120	156.05	17978	0.22
121	156.95	165822	2.03
122	157.95	69851	0.85
123	158.80	84792	1.04
124	159.75	18043	0.22
125	160.85	24030	0.29
126	161.85	99095	1.21
127	162.80	155049	1.89
128	163.75	34862	0.43
129	164.85	13090	0.16
130	165.75	7197	0.09
131	166.75	5385	0.07
132	167.80	1319	0.02
133	168.75	2584	0.03
134	169.85	10984	0.13
135	170.75	5228	0.06

#	m/z	Abs. Int.	Rel. Int.
136	171.95	1364	0.02
137	173.95	103633	1.27
138	174.95	1072377	13.11
139	175.85	200879	2.45
140	176.85	16800	0.21
141	177.85	17155	0.21
142	178.85	6631	0.08
143	179.95	11087	0.14
144	180.95	2034	0.02
145	183.35	1462	0.02
146	184.35	2268	0.03
147	185.95	55	0.00
148	186.95	31125	0.38
149	187.95	413165	5.05
150	188.95	282757	3.46
151	189.90	1979342	24.19
152	190.85	381280	4.66
153	191.85	39767	0.49
154	192.85	12267	0.15
155	193.85	2677	0.03
156	194.25	3532	0.04
157	195.25	2138	0.03
158	196.25	3102	0.04
159	197.30	1359	0.02
160	198.30	397	0.00
161	199.80	4044	0.05
162	200.85	2987	0.04
163	201.90	558	0.01
164	202.95	6648	0.08
165	203.95	4859	0.06
166	204.95	49680	0.61
167	205.90	121752	1.49
168	206.85	36484	0.45
169	207.85	13677	0.17
170	208.85	2377	0.03
171	209.85	1672	0.02
172	210.90	576	0.01
173	212.00	1131	0.01
174	213.05	1264	0.02
175	214.05	8644	0.11
176	215.00	49861	0.61
177	215.95	25834	0.32
178	216.85	17650	0.22
179	217.85	39438	0.48
180	218.75	10187	0.12
181	219.75	839	0.01
182	220.90	3943	0.05
183	221.95	2258	0.03
184	222.95	6823	0.08
185	223.90	14342	0.18
186	224.85	4564	0.06
187	225.90	758	0.01
188	227.90	535	0.01
189	228.90	442	0.01
190	229.90	332	0.00
191	231.05	3401	0.04
192	232.05	19508	0.24
193	232.95	71714	0.88
194	234.05	195566	2.39
195	234.95	1343018	16.41
196	235.95	278787	3.41
197	236.95	11372	0.14
198	237.95	2509	0.03
199	238.95	1363	0.02
200	240.00	757	0.01
201	244.00	455	0.01
202	245.00	827	0.01
203	246.00	211	0.00
204	247.95	1051	0.01
205	248.95	5898	0.07
206	249.95	10161	0.12
207	250.95	54739	0.67
208	251.85	19206	0.23
209	252.85	6162	0.08
210	253.90	286	0.00
211	255.90	93	0.00
212	256.90	336	0.00
213	257.90	33	0.00
214	258.90	990	0.01
215	259.90	644	0.01
216	260.90	9	0.00
217	262.55	4758	0.06
218	263.50	5081	0.06
219	264.45	4777	0.06
220	265.45	2455	0.03
221	267.30	1196	0.01

#	m/z	Abs. Int.	Rel. Int.
222	268.35	1102	0.01
223	269.35	2784	0.03
224	270.50	1109	0.01
225	271.60	1417	0.02
226	272.75	674	0.01
227	273.75	13550	0.17
228	274.75	25309	0.31
229	275.70	30111	0.37
230	276.65	12888	0.16
231	277.60	3333	0.04
232	278.65	10050	0.12
233	279.60	12090	0.15
234	280.55	2016	0.02
235	283.60	56	0.00
236	284.60	685	0.01
237	285.70	751	0.01
238	286.70	1899	0.02
239	287.95	3297	0.04
240	288.95	47922	0.59
241	289.90	85349	1.04
242	290.85	44296	0.54
243	291.85	14069	0.17
244	292.95	12157	0.15
245	293.90	31868	0.39
246	294.85	7635	0.09
247	295.90	191	0.00
248	296.90	226	0.00
249	297.60	563	0.01
250	298.65	3130	0.04
251	299.65	1249	0.02
252	300.65	47393	0.58
253	301.65	59262	0.72
254	302.65	98136	1.20
255	303.55	36974	0.45
256	304.55	14240	0.17
257	305.55	5803	0.07
258	306.60	134	0.00
259	309.60	1311	0.02
260	310.60	158	0.00
261	311.60	593	0.01
262	312.60	147	0.00
263	315.80	91	0.00
264	317.85	90972	1.11
265	318.85	2845918	34.78
266	319.80	6197178	75.74
267	320.75	1723056	21.06
268	321.75	119644	1.46
269	322.75	394	0.00
270	323.75	333	0.00
271	324.80	561	0.01
272	325.80	354	0.00
273	328.80	299	0.00
274	329.80	971	0.01
275	330.80	16	0.00
276	331.80	810	0.01
277	332.80	101	0.00
278	333.80	381	0.00
279	335.80	154	0.00
280	336.80	426	0.01
281	338.80	101	0.00
282	340.80	387	0.00
283	343.80	421	0.01
284	344.80	197	0.00
285	345.80	1033	0.01
286	347.80	21	0.00
287	348.80	218	0.00
288	349.80	834	0.01
289	350.80	404	0.00
290	351.80	565	0.01
291	352.80	472	0.01
292	354.80	727	0.01
293	356.80	200	0.00
294	363.80	507	0.01
295	364.80	673	0.01
296	365.80	279	0.00
297	366.80	74	0.00
298	368.80	566	0.01
299	369.80	641	0.01
300	370.80	455	0.01
301	371.80	547	0.01
302	373.80	263	0.00
303	378.80	955	0.01
304	383.80	369	0.00
305	384.80	629	0.01
306	385.80	184	0.00
307	388.80	353	0.00

#	m/z	Abs. Int.	Rel. Int.
308	389.80	332	0.00
309	390.80	973	0.01
310	391.80	131	0.00
311	392.80	227	0.00
312	395.80	118	0.00
313	397.80	355	0.00
314	399.80	522	0.01
315	404.80	1068	0.01
316	408.35	1471	0.02
317	410.40	778	0.01
318	411.40	701	0.01
319	417.40	247	0.00
320	418.40	129	0.00
321	420.40	54	0.00
322	424.40	760	0.01
323	425.40	141	0.00
324	427.40	91	0.00
325	429.40	522	0.01
326	430.40	318	0.00
327	431.40	107	0.00
328	432.40	397	0.00
329	436.40	51	0.00
330	437.40	332	0.00
331	438.40	74	0.00
332	439.40	188	0.00
333	443.40	60	0.00
334	444.40	988	0.01
335	445.40	454	0.01
336	449.40	159	0.00
337	450.40	243	0.00
338	451.40	525	0.01
339	452.40	274	0.00
340	453.40	219	0.00
341	456.40	303	0.00
342	458.40	257	0.00
343	463.40	438	0.01
344	464.40	726	0.01
345	465.40	747	0.01
346	469.40	347	0.00
347	470.40	275	0.00
348	471.40	943	0.01
349	472.40	313	0.00
350	476.40	68	0.00
351	477.40	143	0.00
352	483.40	573	0.01
353	484.40	441	0.01
354	485.40	491	0.01
355	489.40	624	0.01
356	490.40	678	0.01
357	491.40	755	0.01
358	492.40	446	0.01
359	496.40	196	0.00
360	497.40	355	0.00
361	498.40	36	0.00
362	503.40	376	0.00
363	504.40	1057	0.01
364	505.40	241	0.00
365	508.40	234	0.00
366	509.40	772	0.01
367	510.40	495	0.01
368	511.40	773	0.01
369	516.40	566	0.01
370	517.40	6	0.00
371	523.40	157	0.00
372	524.40	1133	0.01
373	528.40	287	0.00
374	529.40	634	0.01
375	530.40	855	0.01
376	531.40	507	0.01
377	535.40	293	0.00
378	536.40	62	0.00
379	537.40	104	0.00
380	539.40	37	0.00
381	542.40	197	0.00
382	543.40	118	0.00
383	544.40	1139	0.01
384	548.40	339	0.00
385	549.40	1040	0.01
386	550.40	140	0.00
387	551.40	714	0.01
388	555.40	348	0.00
389	556.40	118	0.00
390	563.40	535	0.01
391	564.40	261	0.00
392	565.40	99	0.00
393	568.40	543	0.01

Method

[Comment]

==== Analytical Line 1 ====

[GC-2010]	
Column Oven Temp.	:30.0 °C
Injection Temp.	:150.00 °C
Injection Mode	:Split
Pressure	:12.8 kPa
Total Flow	:6.6 mL/min
Column Flow	:0.60 mL/min
Linear Velocity	:27.9 cm/sec
Purge Flow	:3.0 mL/min
Split Ratio	:5.0

[GCMS-QP2010 Ultra]	
IonSourceTemp	:200.00 °C
Interface Temp.	:200.00 °C
Solvent Cut Time	:0.10 min
Detector Gain Mode	:Relative
Detector Gain	:1.35 kV +0.00 kV
Threshold	:0

[MS Table]	
--Group 1 - Event 1--	
Start Time	:0.10min
End Time	:25.00min
ACQ Mode	:Scan
Event Time	:0.10sec
Scan Speed	:20000
Start m/z	:35.00
End m/z	:1090.00

Sample Information

Sample Name	: M.Satenik
Sample ID	: IVA2351pr
Analyzed	: 2019-05-06 15:55:50
Vial #	: 12
Injection Volume	: 0.10
$EndIf$Data File	: C:\GCMSsolution\Data\widma\2019\05_2019\M
Method File	: C:\GCMSsolution\System\Tune1\DI EI 1 metoda
Tuning File	: C:\GCMSsolution\System\Tune1\tuning06.05.20

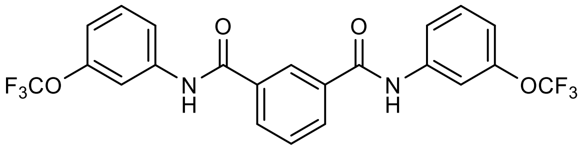

N^1,N^3-bis(3-(trifluoromethoxy)phenyl)isophthalamide **4ab**

Chemical Formula: $C_{22}H_{14}F_6N_2O_4$

Molecular Weight: 484.35

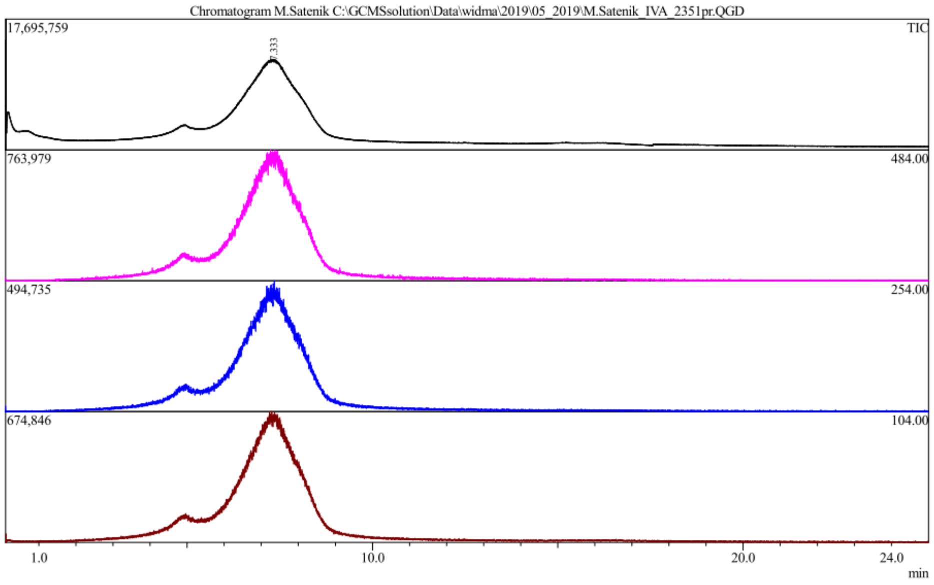

Compound **4ab**

Spectrum

Line#:1 R.Time:7.333(Scan#:4341)
MassPeaks:698
RawMode:Single 7.333(4341) BasePeak:308(2289274)
BG Mode:None Group 1 - Event 1 Scan

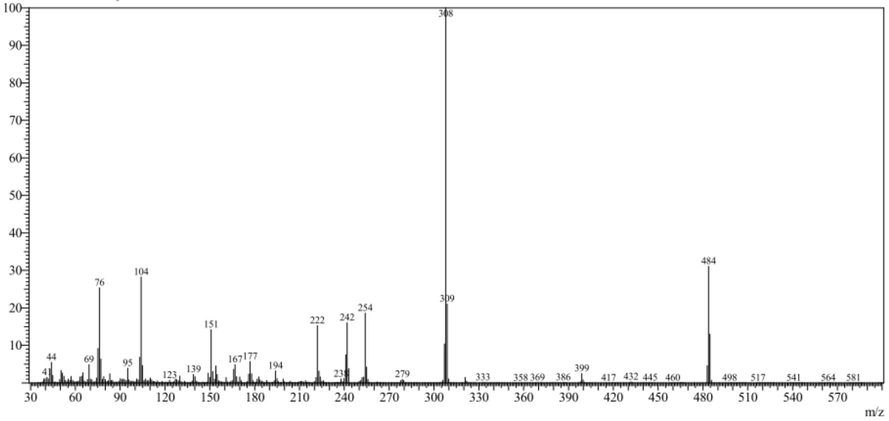

Mass Table
Line#:1 R.Time:7.333(Scan#:4341)
MassPeaks:698
RawMode:Single 7.333(4341) BasePeak:308(2289274)
BG Mode:None Group 1 - Event 1 Scan

#	m/z	Abs. Int.	Rel. Int.
1	35.00	949	0.04
2	35.95	1336	0.06
3	36.95	1895	0.08
4	37.95	7407	0.32
5	38.95	24434	1.07
6	39.85	25692	1.12
7	40.95	34445	1.50
8	41.85	23970	1.05
9	42.95	84658	3.70
10	43.95	125988	5.50
11	44.85	46305	2.02
12	45.85	5992	0.26
13	46.85	5045	0.22
14	47.85	2169	0.09
15	48.45	1116	0.05
16	49.45	20799	0.91
17	50.40	76976	3.36
18	51.35	60677	2.65
19	52.35	40341	1.76
20	53.35	16127	0.70
21	54.25	7377	0.32
22	55.15	23245	1.02
23	56.25	16997	0.74
24	57.20	39789	1.74
25	58.15	16145	0.71
26	59.15	7321	0.32
27	60.10	8512	0.37
28	61.15	7597	0.33
29	62.15	12103	0.53
30	63.15	36738	1.60
31	64.15	40561	1.77
32	65.05	62387	2.73
33	66.05	11984	0.52
34	67.05	5700	0.25
35	68.05	19972	0.87
36	69.05	111418	4.87
37	69.95	22906	1.00
38	71.05	19738	0.86
39	72.05	6140	0.27
40	73.15	10133	0.44
41	74.15	30571	1.34
42	75.15	210697	9.20
43	76.10	580906	25.38
44	77.05	146617	6.40
45	78.05	21324	0.93
46	79.05	37920	1.66
47	80.05	20184	0.88
48	81.05	10025	0.44
49	82.05	13182	0.58
50	83.05	55738	2.43
51	83.95	15261	0.67
52	84.80	14914	0.65
53	85.75	5322	0.23
54	87.05	7102	0.31
55	87.95	4771	0.21
56	89.05	10985	0.48
57	90.05	25662	1.12
58	91.05	20623	0.90
59	91.95	24817	1.08
60	92.95	19427	0.85
61	94.05	15490	0.68
62	95.05	90521	3.95
63	95.95	19144	0.84
64	97.00	9775	0.43
65	98.00	11993	0.52
66	98.95	8091	0.35
67	100.05	7989	0.35
68	100.95	24420	1.07
69	102.05	19793	0.86
70	103.05	156964	6.86
71	103.95	646500	28.24
72	104.95	107123	4.68
73	105.95	15235	0.67
74	107.05	25239	1.10
75	108.05	14235	0.62
76	109.15	15956	0.70
77	110.15	30642	1.34
78	111.05	19335	0.84
79	112.05	9891	0.43
80	113.05	9196	0.40
81	114.05	3856	0.17
82	115.00	11847	0.52
83	115.95	4440	0.19
84	116.95	3383	0.15
85	117.85	9410	0.41
86	118.85	4275	0.19
87	119.85	3492	0.15
88	120.90	5710	0.25
89	121.95	3446	0.15
90	122.95	16308	0.71
91	123.85	4478	0.20
92	124.95	5913	0.26
93	125.95	8705	0.38
94	126.95	23722	1.04
95	127.85	20875	0.91
96	128.95	14079	0.61
97	129.95	41803	1.83
98	130.85	8578	0.37
99	132.05	3298	0.14
100	133.05	10488	0.46
101	133.95	4001	0.17
102	135.15	2756	0.12
103	136.15	2045	0.09
104	137.05	4122	0.18
105	138.15	11747	0.51
106	139.10	52336	2.29
107	140.05	37845	1.65
108	141.05	10642	0.46
109	142.05	3973	0.17
110	143.05	2488	0.11
111	144.25	1464	0.06
112	145.25	5493	0.24
113	146.15	3382	0.15
114	147.15	2313	0.10
115	147.95	4821	0.21
116	148.90	59636	2.61
117	149.95	33893	1.48
118	150.90	325764	14.23
119	151.85	70027	3.06
120	153.05	22984	1.00
121	154.00	103275	4.51
122	154.95	51989	2.27
123	155.95	13000	0.57
124	156.95	9387	0.41
125	157.95	4784	0.21
126	158.85	6143	0.27
127	159.85	4025	0.18
128	160.85	32356	1.41
129	161.75	7135	0.31
130	162.95	2426	0.11
131	163.95	7606	0.33
132	164.95	15066	0.66
133	165.95	82735	3.61
134	166.85	111841	4.89
135	167.85	38883	1.70
136	168.85	6996	0.31
137	170.00	36507	1.59
138	170.95	15546	0.68
139	171.95	2237	0.10
140	173.00	450	0.02
141	174.05	738	0.03
142	175.05	9777	0.43
143	176.05	54723	2.39
144	176.95	131046	5.72
145	177.95	56326	2.46
146	178.95	13020	0.57
147	179.70	3068	0.13
148	180.75	5077	0.22
149	181.75	22692	0.99
150	182.70	37254	1.63
151	183.65	18300	0.80
152	184.85	11094	0.48
153	185.75	6172	0.27
154	187.15	2625	0.11
155	188.05	15210	0.66
156	189.05	4787	0.21
157	190.05	420	0.02
158	191.05	2558	0.11
159	192.05	3498	0.15
160	193.05	13530	0.59
161	194.00	73760	3.22
162	194.95	26083	1.14
163	195.95	8288	0.36
164	197.05	2332	0.10
165	198.05	3140	0.14
166	199.00	23939	1.05
167	199.95	8281	0.36
168	200.95	988	0.04
169	202.05	3329	0.15
170	202.95	5013	0.22
171	204.05	9102	0.40
172	204.95	2358	0.10
173	205.95	790	0.03
174	206.95	3314	0.14
175	208.05	1100	0.05
176	209.05	3779	0.17
177	210.05	8820	0.39
178	211.05	8937	0.39
179	212.00	9321	0.41
180	213.05	3851	0.17
181	214.00	14126	0.62
182	214.95	4435	0.19
183	215.75	5074	0.22
184	216.65	2766	0.12
185	217.70	240	0.01
186	219.05	1318	0.06
187	220.05	8323	0.36
188	221.05	32338	1.41
189	221.95	349432	15.26
190	222.95	72833	3.18
191	223.95	37427	1.63
192	224.95	11357	0.50
193	225.95	13834	0.60
194	226.85	3530	0.15
195	227.85	1302	0.06
196	229.10	1368	0.06
197	230.10	570	0.02
198	232.00	1158	0.05
199	233.00	303	0.01
200	234.05	1461	0.06
201	235.00	4458	0.19
202	235.95	2194	0.10
203	236.95	4083	0.18
204	237.90	24798	1.08
205	238.85	8525	0.37
206	239.95	28435	1.24
207	240.95	171990	7.51
208	241.90	367996	16.07
209	242.85	87761	3.83
210	243.85	1957	0.09
211	244.85	262	0.01
212	246.20	1091	0.05
213	247.20	127	0.01
214	248.20	373	0.02
215	248.95	624	0.03
216	249.95	9421	0.41
217	250.95	14715	0.64
218	251.95	32328	1.41
219	252.95	35024	1.53
220	253.95	426741	18.64
221	254.85	96966	4.24
222	255.85	21777	0.95
223	256.85	3802	0.17
224	257.90	242	0.01
225	258.90	317	0.01
226	259.90	372	0.02
227	261.15	2816	0.12
228	262.05	5860	0.26
229	263.05	3245	0.14
230	264.05	1074	0.05
231	264.70	713	0.03
232	265.65	869	0.04
233	266.70	1606	0.07
234	267.70	386	0.02
235	269.90	1278	0.06
236	270.90	188	0.01
237	271.90	463	0.02
238	272.90	386	0.02
239	273.90	66	0.00
240	274.90	133	0.01
241	275.90	636	0.03
242	276.85	188	0.01
243	277.85	15442	0.67
244	278.80	21931	0.96
245	279.75	15235	0.67
246	280.75	3037	0.13
247	281.75	1901	0.08
248	282.90	258	0.01
249	283.85	1082	0.05
250	284.85	1727	0.08
251	285.85	905	0.04
252	287.25	985	0.04
253	288.30	1407	0.06
254	289.30	169	0.01
255	290.30	106	0.00
256	292.30	24	0.00
257	293.30	116	0.01
258	294.30	266	0.01
259	295.30	505	0.02
260	296.30	184	0.01
261	297.30	120	0.01
262	298.30	155	0.01
263	299.30	435	0.02
264	300.30	538	0.02
265	301.30	339	0.01
266	302.30	216	0.01
267	303.30	221	0.01
268	303.90	211	0.01
269	304.95	5001	0.22
270	305.95	15794	0.69
271	306.95	238476	10.42
272	307.85	2289274	100.00
273	308.85	482861	21.09
274	309.85	23962	1.05
275	310.85	2330	0.10
276	311.85	441	0.02
277	312.95	1685	0.07
278	313.90	84	0.00
279	314.90	48	0.00
280	317.90	151	0.01
281	318.90	130	0.01
282	319.85	1362	0.06
283	320.85	34351	1.50
284	321.75	10256	0.45
285	322.75	2226	0.10
286	323.80	173	0.01
287	324.80	794	0.03
288	325.80	255	0.01
289	327.30	943	0.04
290	330.30	478	0.02
291	332.75	3250	0.14
292	333.75	1713	0.07
293	334.80	115	0.01
294	336.80	182	0.01
295	337.80	362	0.02
296	338.80	267	0.01
297	339.80	22	0.00
298	340.80	535	0.02
299	341.50	542	0.02
300	342.50	1169	0.05
301	343.95	927	0.04
302	345.00	635	0.03
303	346.00	102	0.00
304	347.00	400	0.02
305	348.00	286	0.01
306	349.00	49	0.00
307	351.00	370	0.02
308	353.00	267	0.01
309	354.00	331	0.01
310	355.00	341	0.01
311	356.00	26	0.00
312	358.00	480	0.02
313	359.00	442	0.02
314	360.00	380	0.02
315	361.00	187	0.01
316	362.00	326	0.01
317	363.00	423	0.02
318	364.00	188	0.01
319	365.00	231	0.01
320	366.00	283	0.01
321	367.20	482	0.02
322	368.15	1123	0.05
323	369.20	1218	0.05
324	370.20	411	0.02
325	371.30	1097	0.05
326	372.30	698	0.03
327	373.30	45	0.00
328	375.30	129	0.01
329	376.30	288	0.01
330	378.80	2138	0.09
331	379.80	529	0.02
332	381.20	839	0.04
333	382.20	129	0.01
334	383.20	111	0.00
335	384.20	63	0.00
336	385.20	280	0.01
337	386.55	2601	0.11
338	387.40	742	0.03
339	388.45	1090	0.05
340	396.95	845	0.04
341	397.95	6719	0.29
342	398.85	57806	2.53
343	399.85	18498	0.81
344	400.85	3698	0.16
345	402.90	308	0.01
346	403.90	526	0.02
347	404.90	137	0.01
348	405.90	332	0.01
349	407.90	134	0.01
350	414.90	383	0.02
351	416.90	420	0.02
352	418.90	69	0.00
353	419.90	225	0.01
354	420.90	144	0.01
355	421.90	205	0.01
356	422.90	220	0.01
357	423.90	51	0.00
358	424.90	466	0.02
359	426.90	208	0.01
360	427.90	84	0.00
361	430.85	1709	0.07
362	431.85	6969	0.30
363	432.75	3066	0.13
364	436.80	216	0.01
365	438.80	8	0.00
366	439.80	8	0.00
367	440.80	255	0.01
368	441.80	169	0.01
369	442.80	260	0.01
370	443.80	142	0.01
371	444.75	1675	0.07
372	445.80	551	0.02
373	446.80	371	0.02
374	447.80	136	0.01
375	457.80	160	0.01
376	458.80	99	0.00
377	459.80	277	0.01
378	460.80	137	0.01
379	461.80	228	0.01
380	462.90	1129	0.05
381	463.70	434	0.02
382	464.70	3304	0.14
383	465.65	1162	0.05
384	466.35	1079	0.05
385	467.30	1648	0.07
386	474.30	285	0.01
387	479.30	87	0.00
388	482.85	105109	4.59
389	483.80	711670	31.09
390	484.75	297273	12.99
391	485.75	16339	0.71
392	487.75	59	0.00
393	490.80	35	0.00

Elemental Composition Report

Single Mass Analysis

Tolerance = 5.0 PPM / DBE: min = -50.0, max = 80.0
Element prediction: Off
Number of isotope peaks used for i-FIT = 6

Monoisotopic Mass, Odd and Even Electron Ions
536 formula(e) evaluated with 13 results within limits (up to 50 closest results for each mass)
Elements Used:
C: 0-25 H: 0-25 N: 0-5 O: 0-5 F: 0-10
190514_IVA_2351 16 (0.177) Cm (11:22-(25:72+1:8)x2.000)
1: TOF MS AP+

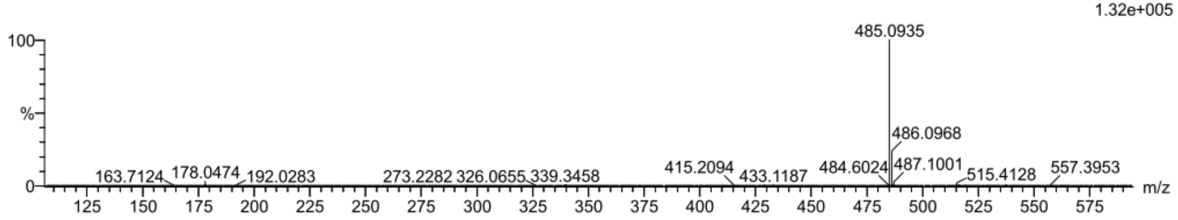

Minimum:				-50.0				
Maximum:		15.0	5.0	80.0				
Mass	Calc. Mass	mDa	PPM	DBE	i-FIT	Norm	Conf(%)	Formula
485.0935	485.0936	-0.1	-0.2	13.5	1372.6	7.621	0.05	C22 H15 N2 O4 F6
	485.0936	-0.1	-0.2	21.0	1375.9	10.893	0.00	C25 H13 N5 O4 F2
	485.0934	0.1	0.2	10.0	1369.5	4.523	1.09	C17 H14 N5 O4 F7
	485.0925	1.0	2.1	17.5	1375.0	9.960	0.00	C25 H14 N2 O3 F5
	485.0945	-1.0	-2.1	6.0	1371.5	6.530	0.15	C14 H15 N5 O5 F8
	485.0947	-1.2	-2.5	9.5	1371.3	6.261	0.19	C19 H16 N2 O5 F7
	485.0947	-1.2	-2.5	17.0	1374.2	9.254	0.01	C22 H14 N5 O5 F3
	485.0923	1.2	2.5	14.0	1370.5	5.534	0.40	C20 H13 N5 O3 F6
	485.0923	1.2	2.5	6.5	1369.3	4.315	1.34	C17 H15 N2 O3 F10
	485.0950	-1.5	-3.1	11.0	1365.1	0.061	94.08	C20 H13 N3 F10
	485.0952	-1.7	-3.5	14.5	1373.6	8.658	0.02	C25 H14 F9
	485.0912	2.3	4.7	10.5	1368.6	3.624	2.67	C20 H14 N2 O2 F9
	485.0911	2.4	4.9	18.0	1373.6	8.630	0.02	C23 H12 N5 O2 F5

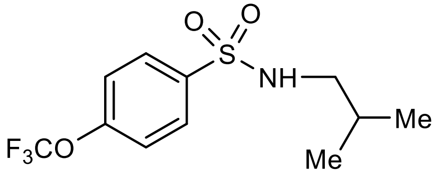

N-isobutyl-4-(trifluoromethoxy)benzenesulfonamide **4ac**

Chemical Formula: $C_{11}H_{14}F_3NO_3S$

Molecular Weight: 297.29

Compound **4ac**

Sample Information

Sample Name	: M.Satenik
Sample ID	: IVA_ 2068
Analyzed	: 2019-07-01 18:25:31
Vial #	: 4
Injection Volume	: 0.30
$EndIf$Data File	: C:\GCMSsolution\Data\widma\2019\07_2019\IV
Method File	: C:\GCMSsolution\Data\widma\metody\ewelina 0
Tuning File	: C:\GCMSsolution\System\Tune1\tuning12.06.20

Configuration Control

<<Column>>
Name : ZB5MSi
Serial # :
Thickness : 0.25um
Length : 30.0m
Inside Diameter : 0.25mm
Max Usable Temp : 360°C
Installation Date : 2018/06/05
Description :

Method

[Comment]

==== Analytical Line 1 ====

[GC-2010]

Column Oven Temp.	:50.0 °C
Injection Temp.	:250.00 °C
Injection Mode	:Splitless
Sampling Time	:1.00 min
Pressure	:70.0 kPa
Total Flow	:63.5 mL/min
Column Flow	:1.22 mL/min
Linear Velocity	:40.1 cm/sec
Purge Flow	:1.0 mL/min
Split Ratio	:50.0

Oven Temp. Program

Rate	Temperature(°C)	Hold Time(min)
-	50.0	3.00
30.00	150.0	5.00
25.00	250.0	25.00

[GCMS-QP2010 Ultra]

IonSourceTemp	:200.00 °C
Interface Temp.	:200.00 °C
Solvent Cut Time	:3.50 min
Detector Gain Mode	:Absolute
Detector Gain	:1.20 kV
Threshold	:0

[MS Table]
--Group 1 - Event 1--

Start Time	:3.50min
End Time	:40.33min
ACQ Mode	:Scan
Event Time	:0.10sec
Scan Speed	:20000
Start m/z	:35.00
End m/z	:1000.00

[Similarity Search Parameters]

File1(Min.SI:50)	:C:\GCMSsolution\library\NIST11.lib
File2(Min.SI:50)	:C:\GCMSsolution\library\NIST11s.lib
Search Depth	:No PreSearch
Max Hit#	:25
Delete the same Compounds	:OFF
Reverse Search	:OFF
--- Post-search ---	
Option (Match Case)	:OFF

Chromatogram M.Satenik C:\GCMSsolution\Data\widma\2019\07_2019\IVA_2068.qgd

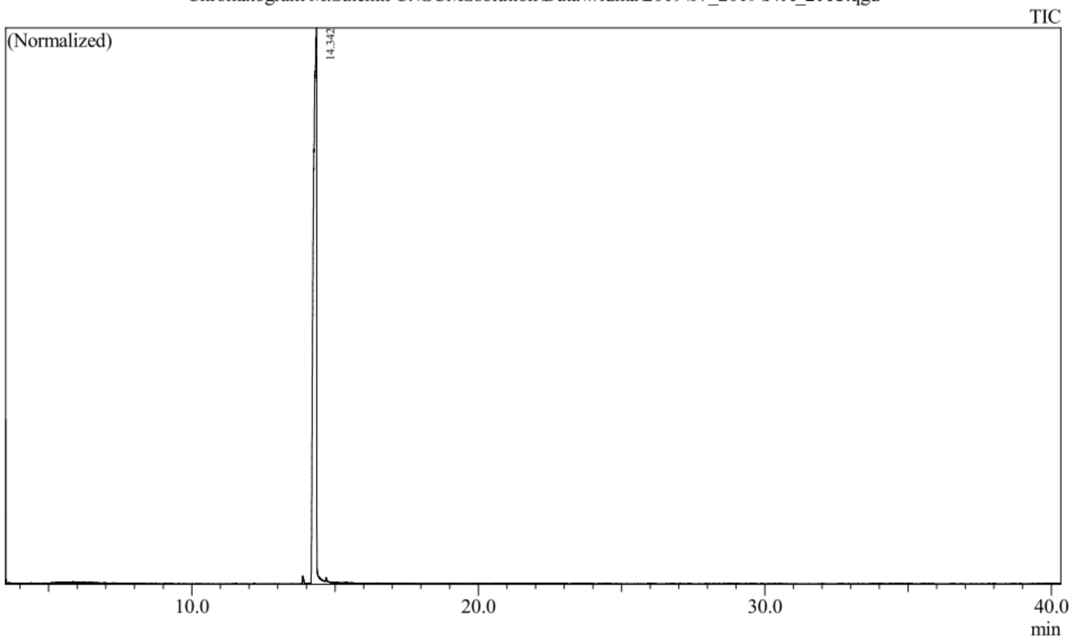

Spectrum

Line#:1 R.Time:14.342(Scan#:6506)
MassPeaks:599
RawMode:Single 14.342(6506) BasePeak:225(8151529)
BG Mode:None Group 1 - Event 1 Scan

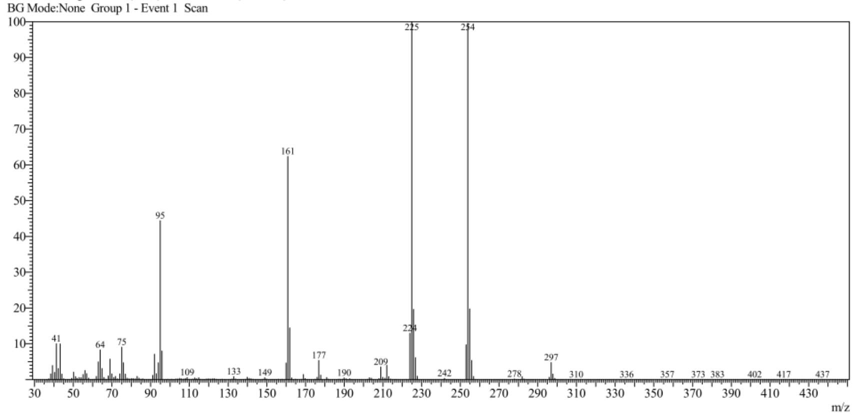

Mass Table
Line#:1 R.Time:14.342(Scan#:6506)
MassPeaks:599
RawMode:Single 14.342(6506) BasePeak:225(8151529)
BG Mode:None Group 1 - Event 1 Scan

#	m/z	Abs. Int.	Rel. Int.
1	36.35	3135	0.04
2	37.35	21180	0.26
3	38.35	129563	1.59
4	39.30	322554	3.96
5	40.35	163436	2.00
6	41.30	818087	10.04
7	42.20	251518	3.09
8	43.25	812770	9.97
9	44.15	127586	1.57
10	45.15	18301	0.22
11	46.15	6730	0.08
12	47.25	6971	0.09
13	48.15	6358	0.08
14	49.25	27827	0.34
15	50.15	168014	2.06
16	51.15	69183	0.85
17	52.00	31591	0.39
18	53.05	48911	0.60
19	54.05	44956	0.55
20	55.05	117849	1.45
21	56.05	209908	2.58
22	56.95	134684	1.65
23	57.95	32262	0.40
24	58.95	4866	0.06
25	59.95	2654	0.03
26	60.95	10372	0.13
27	61.95	73710	0.90
28	62.95	403837	4.95
29	63.95	677597	8.31
30	64.85	253014	3.10
31	65.85	50529	0.62
32	66.85	11349	0.14
33	68.05	81388	1.00
34	69.00	466666	5.72
35	69.95	129074	1.58
36	70.95	50806	0.62
37	71.90	74966	0.92
38	72.85	18259	0.22
39	74.05	131286	1.61
40	75.00	737877	9.05
41	75.95	384657	4.72
42	76.95	128144	1.57
43	77.95	32468	0.40
44	78.95	20189	0.25
45	80.05	23001	0.28

#	m/z	Abs. Int.	Rel. Int.
46	80.95	23987	0.29
47	82.05	17224	0.21
48	83.00	73678	0.90
49	83.95	33202	0.41
50	84.95	9004	0.11
51	85.85	18610	0.23
52	86.75	4879	0.06
53	87.95	3455	0.04
54	89.05	5188	0.06
55	90.05	4242	0.05
56	91.05	96613	1.19
57	91.95	578804	7.10
58	92.95	134573	1.65
59	93.95	382689	4.69
60	94.90	3619336	44.40
61	95.85	650023	7.97
62	97.85	4923	0.06
63	98.80	7592	0.09
64	99.95	2506	0.03
65	100.90	10456	0.13
66	101.95	5086	0.06
67	102.95	12353	0.15
68	103.95	15637	0.19
69	104.95	30809	0.38
70	105.85	17082	0.21
71	106.95	15831	0.19
72	107.95	26129	0.32
73	108.90	47454	0.58
74	109.85	10971	0.13
75	110.85	8961	0.11
76	111.85	13245	0.16
77	112.85	36811	0.45
78	113.75	17624	0.22
79	114.85	43449	0.53
80	115.75	10454	0.13
81	116.95	5848	0.07
82	117.95	3529	0.04
83	118.95	12172	0.15
84	119.90	21726	0.27
85	120.85	15235	0.19
86	121.95	17167	0.21
87	122.85	21852	0.27
88	123.85	11845	0.15
89	124.85	3982	0.05
90	125.95	2533	0.03

#	m/z	Abs. Int.	Rel. Int.
91	126.85	8000	0.10
92	127.85	2931	0.04
93	128.85	1727	0.02
94	129.95	4069	0.05
95	130.95	4282	0.05
96	131.95	14154	0.17
97	132.90	68807	0.84
98	133.85	15489	0.19
99	134.85	1623	0.02
100	135.95	2032	0.02
101	136.90	5350	0.07
102	137.85	2693	0.03
103	138.95	8570	0.11
104	139.90	51431	0.63
105	140.85	29704	0.36
106	141.85	21025	0.26
107	142.85	7842	0.10
108	143.85	1026	0.01
109	144.70	1667	0.02
110	145.75	932	0.01
111	146.95	1357	0.02
112	147.95	11372	0.14
113	148.95	44553	0.55
114	149.85	13811	0.17
115	150.85	896	0.01
116	151.90	447	0.01
117	152.85	4042	0.05
118	153.90	236	0.00
119	154.95	4029	0.05
120	155.95	11489	0.14
121	156.85	6001	0.07
122	157.95	11853	0.15
123	159.95	378693	4.65
124	160.90	5083257	62.36
125	161.85	1176777	14.44
126	162.85	40758	0.50
127	163.85	6091	0.07
128	164.70	8903	0.11
129	165.65	2504	0.03
130	167.95	11627	0.14
131	168.90	116142	1.42
132	169.85	24254	0.30
133	170.85	5397	0.07
134	171.85	1136	0.01
135	172.90	541	0.01

#	m/z	Abs. Int.	Rel. Int.
136	173.90	239	0.00
137	174.95	8537	0.10
138	175.95	51839	0.64
139	176.90	435866	5.35
140	177.85	103089	1.26
141	178.85	4947	0.06
142	179.95	5043	0.06
143	180.95	47313	0.58
144	181.85	8869	0.11
145	182.80	2252	0.03
146	184.05	1163	0.01
147	184.95	6190	0.08
148	186.05	1551	0.02
149	187.05	9783	0.12
150	187.95	11294	0.14
151	189.05	9583	0.12
152	189.95	40212	0.49
153	190.95	17814	0.22
154	191.90	6939	0.09
155	192.85	20279	0.25
156	193.85	4312	0.05
157	194.90	383	0.00
158	195.90	811	0.01
159	197.10	1098	0.01
160	198.10	385	0.00
161	199.10	297	0.00
162	201.05	1261	0.02
163	202.15	9251	0.11
164	203.10	45171	0.55
165	204.05	35992	0.44
166	205.05	5134	0.06
167	206.05	399	0.00
168	207.95	23315	0.29
169	208.90	285421	3.50
170	209.85	52346	0.64
171	211.05	33816	0.41
172	212.00	324622	3.98
173	212.95	68674	0.84
174	213.95	12584	0.15
175	214.95	3449	0.04
176	216.05	7119	0.09
177	216.95	4026	0.05
178	217.95	1146	0.01
179	220.95	754	0.01
180	221.95	4247	0.05
181	223.95	1058468	12.98
182	224.95	8151529	100.00
183	225.85	1599256	19.62
184	226.85	502423	6.16
185	227.85	79142	0.97
186	228.85	200	0.00
187	229.85	1347	0.02
188	230.90	153	0.00
189	231.75	1133	0.01
190	232.85	1163	0.01
191	233.85	2015	0.02
192	234.70	677	0.01
193	235.70	962	0.01
194	236.90	561	0.01
195	237.95	2909	0.04
196	238.90	306	0.00
197	239.90	9	0.00
198	240.95	3100	0.04
199	241.85	31240	0.38
200	242.85	5716	0.07
201	243.85	1156	0.01
202	244.90	167	0.00
203	245.90	211	0.00
204	246.90	92	0.00
205	247.90	991	0.01
206	248.90	707	0.01
207	249.90	221	0.00
208	250.95	61	0.00
209	252.95	790905	9.70
210	253.85	8116406	99.57
211	254.85	1611929	19.77
212	255.85	437390	5.37
213	256.85	72397	0.89
214	257.85	3758	0.05
215	258.85	1088	0.01
216	260.90	24	0.00
217	261.90	336	0.00
218	262.90	401	0.00
219	263.90	580	0.01
220	264.90	553	0.01
221	265.90	355	0.00

#	m/z	Abs. Int.	Rel. Int.
222	266.90	264	0.00
223	267.90	390	0.00
224	268.90	666	0.01
225	269.90	63	0.00
226	270.90	75	0.00
227	271.90	60	0.00
228	272.90	95	0.00
229	276.95	896	0.01
230	277.90	15197	0.19
231	278.85	3703	0.05
232	279.95	5526	0.07
233	280.95	4032	0.05
234	281.95	67939	0.83
235	282.85	13812	0.17
236	283.85	4055	0.05
237	284.90	317	0.00
238	285.90	18	0.00
239	286.90	205	0.00
240	287.90	27	0.00
241	292.90	212	0.00
242	295.95	52820	0.65
243	296.90	391375	4.80
244	297.85	125730	1.54
245	298.85	30936	0.38
246	299.85	5442	0.07
247	300.85	1270	0.02
248	301.90	742	0.01
249	302.90	114	0.00
250	305.90	165	0.00
251	306.90	11	0.00
252	307.90	240	0.00
253	309.90	1100	0.01
254	310.90	86	0.00
255	311.90	20	0.00
256	312.90	29	0.00
257	315.90	49	0.00
258	316.90	240	0.00
259	320.90	160	0.00
260	321.90	529	0.01
261	322.90	43	0.00
262	323.90	3	0.00
263	324.90	129	0.00
264	325.90	173	0.00
265	326.90	36	0.00
266	327.90	32	0.00
267	330.90	216	0.00
268	331.90	147	0.00
269	335.90	303	0.00
270	337.90	139	0.00
271	340.90	185	0.00
272	341.90	220	0.00
273	342.90	245	0.00
274	344.90	237	0.00
275	345.90	133	0.00
276	346.90	55	0.00
277	347.90	109	0.00
278	355.90	22	0.00
279	356.90	328	0.00
280	360.90	61	0.00
281	361.90	209	0.00
282	362.90	89	0.00
283	363.90	197	0.00
284	364.90	172	0.00
285	365.90	125	0.00
286	366.90	63	0.00
287	367.90	103	0.00
288	371.90	107	0.00
289	372.90	236	0.00
290	376.90	204	0.00
291	377.90	111	0.00
292	380.90	65	0.00
293	381.90	144	0.00
294	382.90	425	0.01
295	383.90	4	0.00
296	384.90	350	0.00
297	386.90	200	0.00
298	391.90	83	0.00
299	392.90	11	0.00
300	395.90	119	0.00
301	396.90	213	0.00
302	397.90	186	0.00
303	400.90	9	0.00
304	402.10	1358	0.02
305	403.10	237	0.00
306	404.10	220	0.00
307	405.10	221	0.00

#	m/z	Abs. Int.	Rel. Int.
308	407.10	229	0.00
309	408.10	27	0.00
310	409.10	1	0.00
311	411.10	212	0.00
312	412.10	34	0.00
313	416.10	180	0.00
314	417.10	449	0.01
315	418.10	36	0.00
316	420.10	19	0.00
317	421.10	51	0.00
318	422.10	177	0.00
319	423.10	328	0.00
320	424.10	132	0.00
321	425.10	260	0.00
322	427.10	136	0.00
323	431.10	39	0.00
324	432.10	210	0.00
325	437.10	526	0.01
326	438.10	198	0.00
327	441.10	85	0.00
328	442.10	420	0.01
329	443.10	194	0.00
330	444.10	76	0.00
331	446.10	18	0.00
332	447.10	143	0.00
333	451.10	156	0.00
334	452.10	202	0.00
335	456.10	69	0.00
336	456.70	395	0.00
337	457.70	1395	0.02
338	458.70	267	0.00
339	461.70	292	0.00
340	462.70	389	0.00
341	463.70	68	0.00
342	464.70	16	0.00
343	465.70	42	0.00
344	466.70	181	0.00
345	467.70	146	0.00
346	471.70	112	0.00
347	475.70	131	0.00
348	476.70	424	0.01
349	480.70	43	0.00
350	481.70	381	0.00
351	482.70	145	0.00
352	483.70	56	0.00
353	485.70	200	0.00
354	486.70	293	0.00
355	489.70	76	0.00
356	495.70	169	0.00
357	496.70	159	0.00
358	500.70	185	0.00
359	501.70	85	0.00
360	502.70	328	0.00
361	503.70	33	0.00
362	504.70	4	0.00
363	507.70	142	0.00
364	508.70	66	0.00
365	510.70	260	0.00
366	515.70	99	0.00
367	516.70	246	0.00
368	519.70	37	0.00
369	521.70	850	0.01
370	522.70	137	0.00
371	524.70	184	0.00
372	525.70	16	0.00
373	526.70	58	0.00
374	530.70	63	0.00
375	531.70	98	0.00
376	535.70	4	0.00
377	536.70	290	0.00
378	540.70	199	0.00
379	541.70	122	0.00
380	542.70	113	0.00
381	543.70	66	0.00
382	544.70	323	0.00
383	545.70	38	0.00
384	551.70	105	0.00
385	556.70	347	0.00
386	559.70	78	0.00
387	560.70	95	0.00
388	561.70	159	0.00
389	562.70	245	0.00
390	563.70	86	0.00
391	564.70	328	0.00
392	567.70	131	0.00
393	570.70	81	0.00

Sample Information

Sample Name	: M.Satenik
Sample ID	: IVA_ 2079
Analyzed	: 2019-05-13 16:00:58
Vial #	: 1
Injection Volume	: 0.10
$EndIf$Data File	: C:\GCMSsolution\Data\widma\2019\05_2019\M
Method File	: C:\GCMSsolution\System\Tune1\DI EI 1 metoda
Tuning File	: C:\GCMSsolution\System\Tune1\tuning06.05.20

Method

[Comment]

═══ Analytical Line 1 ═══

[GC-2010]

Column Oven Temp.	:30.0 °C
Injection Temp.	:150.00 °C
Injection Mode	:Split
Pressure	:12.8 kPa
Total Flow	:6.6 mL/min
Column Flow	:0.60 mL/min
Linear Velocity	:27.9 cm/sec
Purge Flow	:3.0 mL/min
Split Ratio	:5.0

[GCMS-QP2010 Ultra]

IonSourceTemp	:200.00 °C
Interface Temp.	:200.00 °C
Solvent Cut Time	:0.10 min
Detector Gain Mode	:Relative
Detector Gain	:1.35 kV +0.00 kV
Threshold	:0

[MS Table]

--Group 1 - Event 1--

Start Time	:0.10min
End Time	:25.00min
ACQ Mode	:Scan
Event Time	:0.10sec
Scan Speed	:20000
Start m/z	:35.00
End m/z	:1090.00

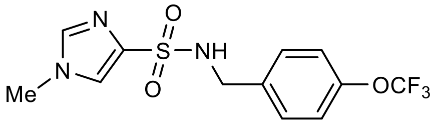

1-methyl-*N*-(4-(trifluoromethoxy)benzyl)-1*H*-imidazole-4-sulfonamide **4ad**

Chemical Formula: $C_{12}H_{12}F_3N_3O_3S$

Molecular Weight: 335.30

Compound **4ad**

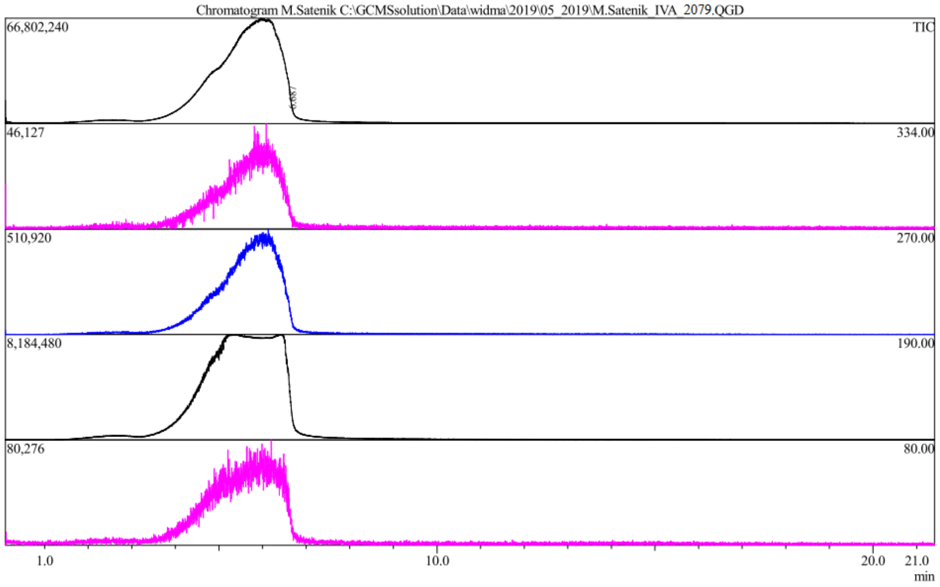

Spectrum

Line#:1 R.Time:6.687(Scan#:3953)
MassPeaks:596
RawMode:Single 6.687(3953) BasePeak:82(2089623)
BG Mode:None Group 1 - Event 1 Scan

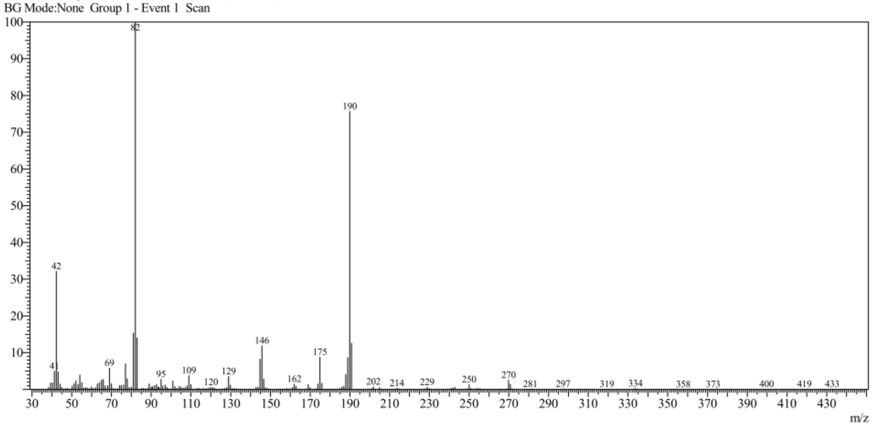

Spectrum

Line#:1 R.Time:6.687(Scan#:3953)
MassPeaks:596
RawMode:Single 6.687(3953) BasePeak:82(2089623)
BG Mode:None Group 1 - Event 1 Scan

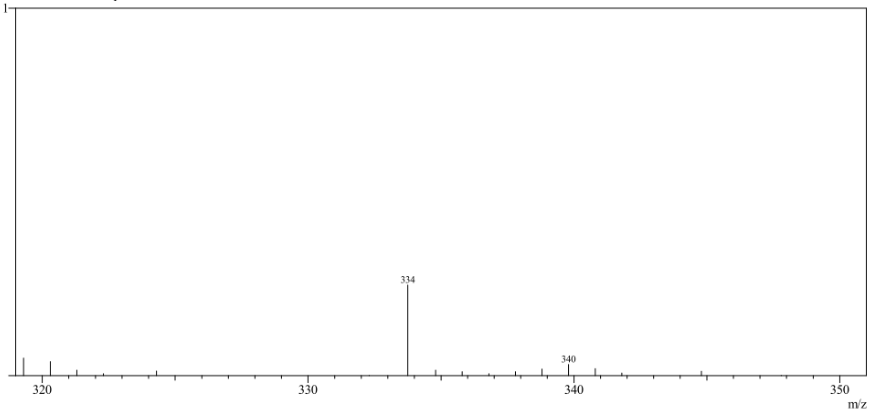

Mass Table
Line#:1 R.Time:6.687(Scan#:3953)
MassPeaks:596
RawMode:Single 6.687(3953) BasePeak:82(2089623)
BG Mode:None Group 1 - Event 1 Scan

#	m/z	Abs. Int.	Rel. Int.
1	35.00	303	0.01
2	36.00	337	0.02
3	37.20	592	0.03
4	38.25	11828	0.57
5	39.25	36214	1.73
6	40.25	36919	1.77
7	41.25	103836	4.97
8	42.25	671663	32.14
9	43.15	99509	4.76
10	44.10	29582	1.42
11	45.05	10994	0.53
12	45.90	2506	0.12
13	46.95	5077	0.24
14	47.90	5175	0.25
15	48.85	1905	0.09
16	49.15	1320	0.06
17	50.15	18500	0.89
18	51.15	32999	1.58
19	52.10	48107	2.30
20	53.15	28008	1.34
21	54.10	82560	3.95
22	55.05	39112	1.87
23	56.05	8901	0.43
24	56.95	8242	0.39
25	57.85	9139	0.44
26	58.95	5051	0.24
27	59.95	13923	0.67
28	60.85	4518	0.22
29	61.95	9436	0.45
30	62.95	31388	1.50
31	63.95	38665	1.85
32	64.95	53125	2.54
33	65.90	56095	2.68
34	66.85	21033	1.01
35	68.05	21453	1.03
36	69.00	121566	5.82
37	69.95	31854	1.52
38	70.95	6281	0.30
39	72.00	2743	0.13
40	73.05	4680	0.22
41	74.05	22633	1.08
42	74.95	24142	1.16
43	76.05	24528	1.17
44	77.00	145756	6.98
45	77.95	61401	2.94
46	78.95	10686	0.51
47	80.05	13225	0.63
48	81.05	320404	15.33
49	82.00	2089623	100.00
50	82.95	293744	14.06
51	83.95	768	0.04
52	85.30	5668	0.27
53	86.25	4434	0.21
54	87.00	1931	0.09
55	88.05	6199	0.30
56	89.00	31125	1.49
57	89.95	13909	0.67
58	90.65	15941	0.76
59	91.65	21719	1.04
60	92.65	28593	1.37
61	93.55	14472	0.69
62	93.95	11428	0.55
63	94.95	57685	2.76
64	95.85	20495	0.98
65	97.00	25379	1.21
66	97.95	12757	0.61
67	98.95	4241	0.20
68	99.95	4223	0.20
69	100.90	48277	2.31
70	101.85	16060	0.77
71	102.85	6592	0.32
72	104.05	17888	0.86
73	104.95	12561	0.60
74	106.05	8909	0.43
75	107.05	11378	0.54
76	108.05	19966	0.96
77	108.95	77979	3.73
78	109.95	27430	1.31
79	110.95	3022	0.14
80	111.95	1863	0.09
81	112.95	4886	0.23
82	113.90	5928	0.28
83	114.85	2324	0.11
84	115.95	5171	0.25
85	116.95	3395	0.16
86	117.95	5177	0.25
87	119.05	8247	0.39
88	119.95	12351	0.59
89	120.95	11731	0.56
90	121.95	5993	0.29
91	123.00	1032	0.05
92	124.85	2874	0.14
93	125.95	3067	0.15
94	126.95	6517	0.31
95	127.95	11375	0.54
96	128.90	73380	3.51
97	129.85	24450	1.17
98	130.85	5416	0.26
99	131.85	4637	0.22
100	132.85	3177	0.15
101	133.85	2355	0.11
102	135.10	1767	0.08
103	136.10	354	0.02
104	137.10	485	0.02
105	138.10	574	0.03
106	138.80	696	0.03
107	139.80	1881	0.09
108	140.80	1276	0.06
109	141.75	2669	0.13
110	142.75	12683	0.61
111	143.75	12521	0.60
112	144.75	172209	8.24
113	145.70	248577	11.90
114	146.65	59101	2.83
115	147.65	10207	0.49
116	148.65	4857	0.23
117	149.70	961	0.05
118	150.70	479	0.02
119	152.70	1522	0.07
120	153.60	719	0.03
121	154.60	1947	0.09
122	155.55	1317	0.06
123	156.25	2298	0.11
124	157.15	1804	0.09
125	158.20	4662	0.22
126	159.15	4176	0.20
127	159.95	1511	0.07
128	160.95	10955	0.52
129	161.95	29560	1.41
130	162.85	17339	0.83
131	163.85	2094	0.10
132	164.90	678	0.03
133	166.90	187	0.01
134	168.05	3994	0.19
135	169.00	27850	1.33
136	169.95	9878	0.47
137	170.95	1554	0.07
138	171.95	1717	0.08
139	172.95	3649	0.17
140	173.95	31669	1.52
141	174.90	182980	8.76
142	175.85	34676	1.66
143	176.85	753	0.04
144	177.50	1729	0.08
145	178.50	434	0.02
146	179.50	950	0.05
147	180.50	873	0.04
148	181.50	1281	0.06
149	182.50	86	0.00
150	183.95	2640	0.13
151	184.95	4724	0.23
152	185.90	12343	0.59
153	186.95	18445	0.88
154	187.95	85122	4.07
155	188.95	180531	8.64
156	189.90	1580845	75.65
157	190.85	262970	12.58
158	192.85	893	0.04
159	193.90	1191	0.06
160	194.90	735	0.04
161	195.90	91	0.00
162	196.90	114	0.01
163	197.90	541	0.03
164	198.95	2138	0.10
165	199.95	2699	0.13
166	200.95	5489	0.26
167	201.85	15206	0.73
168	202.85	1911	0.09
169	203.95	1170	0.06
170	204.90	10775	0.52
171	205.90	288	0.01
172	206.90	834	0.04
173	207.90	396	0.02
174	208.90	694	0.03
175	211.90	608	0.03
176	212.75	1167	0.06
177	213.70	6758	0.32
178	214.65	3336	0.16
179	215.70	363	0.02
180	216.70	100	0.00
181	217.70	693	0.03
182	218.70	474	0.02
183	219.70	1031	0.05
184	220.70	1101	0.05
185	221.70	979	0.05
186	222.70	583	0.03
187	223.70	148	0.01
188	224.70	296	0.01
189	225.70	591	0.03
190	226.95	1703	0.08
191	227.95	4266	0.20
192	228.95	12225	0.59
193	229.85	4527	0.22
194	232.90	269	0.01
195	233.90	887	0.04
196	234.90	255	0.01
197	236.70	421	0.02
198	237.75	1795	0.09
199	238.95	1918	0.09
200	239.90	420	0.02
201	240.95	6384	0.31
202	241.85	8948	0.43
203	242.85	12163	0.58
204	243.85	1448	0.07
205	244.90	304	0.01
206	247.90	26	0.00
207	249.05	4051	0.19
208	249.95	27164	1.30
209	250.95	4894	0.23
210	252.05	2491	0.12
211	252.70	964	0.05
212	253.75	4482	0.21
213	254.70	5255	0.25
214	255.65	4060	0.19
215	256.65	1246	0.06
216	257.70	907	0.04
217	258.70	301	0.01
218	259.70	609	0.03
219	260.70	968	0.05
220	263.70	126	0.01
221	264.70	369	0.02
222	265.70	358	0.02
223	266.70	135	0.01
224	267.70	973	0.05
225	268.85	3607	0.17
226	269.85	51999	2.49
227	270.75	28133	1.35
228	271.75	4270	0.20
229	272.80	608	0.03
230	273.80	639	0.03
231	277.80	826	0.04
232	278.80	276	0.01
233	279.80	607	0.03
234	280.80	653	0.03
235	281.80	282	0.01
236	284.80	141	0.01
237	287.80	282	0.01
238	288.80	38	0.00
239	291.80	381	0.02
240	292.80	602	0.03
241	293.80	559	0.03
242	294.80	33	0.00
243	296.35	1582	0.08
244	297.30	2931	0.14
245	298.30	906	0.04
246	299.30	318	0.02
247	300.30	730	0.03
248	301.30	301	0.01
249	302.30	145	0.01
250	304.30	132	0.01
251	305.30	132	0.01
252	308.30	261	0.01
253	309.30	99	0.00
254	312.30	252	0.01
255	313.30	616	0.03
256	317.30	689	0.03
257	318.30	276	0.01
258	319.30	1003	0.05
259	320.30	803	0.04
260	321.30	306	0.01
261	322.30	112	0.01
262	324.30	264	0.01
263	332.30	14	0.00
264	333.75	5154	0.25
265	334.80	315	0.02
266	335.80	227	0.01
267	336.80	114	0.01
268	337.80	227	0.01
269	338.80	369	0.02
270	339.80	632	0.03
271	340.80	400	0.02
272	341.80	151	0.01
273	344.80	253	0.01
274	347.80	15	0.00
275	352.80	117	0.01
276	353.80	246	0.01
277	354.80	215	0.01
278	357.80	525	0.03
279	358.80	479	0.02
280	359.80	433	0.02
281	360.80	364	0.02
282	361.80	245	0.01
283	364.80	130	0.01
284	368.80	23	0.00
285	372.80	361	0.02

Sample Information

Sample Name	: M.Satenik
Sample ID	: IVA_3010
Analyzed	: 2019-07-30 18:45:43
Vial #	: 6
Injection Volume	: 0.50
$EndIf$Data File	: C:\GCMSsolution\Data\widma\2019\07_2019\IV
Method File	: C:\GCMSsolution\Data\widma\metody\ewelina 0
Tuning File	: C:\GCMSsolution\System\Tune1\tuning26.07.20

Configuration Control

<<Column>>
Name : ZB5MSi
Serial # :
Thickness : 0.25um
Length : 30.0m
Inside Diameter : 0.25mm
Max Usable Temp : 360°C
Installation Date : 2018/06/05
Description :

Method

[Comment]

===== Analytical Line 1 =====

[GC-2010]

Column Oven Temp.	:50.0 °C
Injection Temp.	:250.00 °C
Injection Mode	:Splitless
Sampling Time	:1.00 min
Pressure	:70.0 kPa
Total Flow	:63.5 mL/min
Column Flow	:1.22 mL/min
Linear Velocity	:40.1 cm/sec
Purge Flow	:1.0 mL/min
Split Ratio	:50.0

Oven Temp. Program

Rate	Temperature(°C)	Hold Time(min)
-	50.0	3.00
30.00	150.0	5.00
25.00	250.0	25.00

[GCMS-QP2010 Ultra]

IonSourceTemp	:200.00 °C
Interface Temp.	:200.00 °C
Solvent Cut Time	:3.50 min
Detector Gain Mode	:Relative
Detector Gain	:1.40 kV +0.00 kV
Threshold	:0

[MS Table]
--Group 1 - Event 1--

Start Time	:4.50min
End Time	:40.33min
ACQ Mode	:Scan
Event Time	:0.10sec
Scan Speed	:20000
Start m/z	:35.00
End m/z	:1000.00

[Similarity Search Parameters]

File1(Min.SI:50)	:C:\GCMSsolution\library\NIST11.lib
File2(Min.SI:50)	:C:\GCMSsolution\library\NIST11s.lib
Search Depth	:No PreSearch
Max Hit#	:25
Delete the same Compounds	:OFF
Reverse Search	:OFF
--- Post-search ---	
Option (Match Case)	:OFF

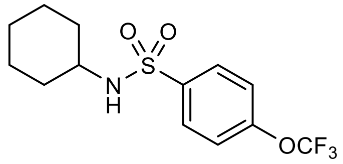

N-cyclohexyl-4-(trifluoromethoxy)benzenesulfonamide **4ae**

Chemical Formula: $C_{13}H_{16}F_3NO_3S$

Molecular Weight: 323.33

Compound **4ae**

Chromatogram M.Satenik C:\GCMSsolution\Data\widma\2019\07_2019\IVA_3010.qgd

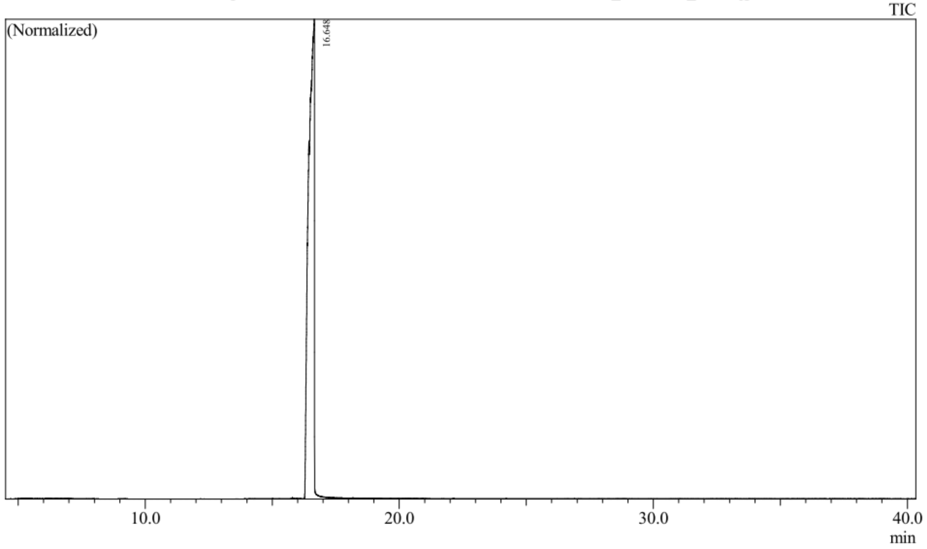

Spectrum

Line#:1 R.Time:16.648(Scan#:7290)
MassPeaks:608
RawMode:Single 16.648(7290) BasePeak:225(8164427)
BG Mode:None Group 1 - Event 1 Scan

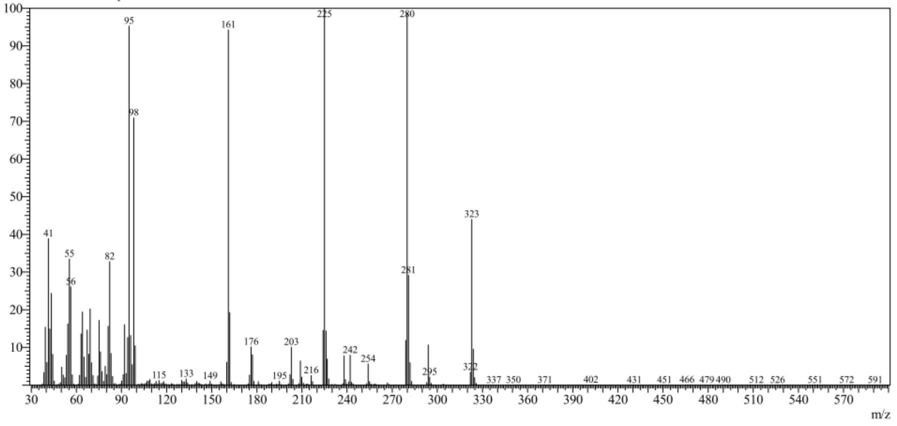

Mass Table
Line#:1 R.Time:16.648(Scan#:7290)
MassPeaks:608
RawMode:Single 16.648(7290) BasePeak:225(8164427)
BG Mode:None Group 1 - Event 1 Scan

#	m/z	Abs. Int.	Rel. Int.
1	35.45	1914	0.02
2	36.45	3498	0.04
3	37.45	30563	0.37
4	38.45	270851	3.32
5	39.40	1255830	15.38
6	40.45	492298	6.03
7	41.40	3173119	38.87
8	42.35	1216846	14.90
9	43.35	1986850	24.34
10	44.25	669505	8.20
11	45.25	86729	1.06
12	46.25	4791	0.06
13	47.35	16457	0.20
14	48.25	20774	0.25
15	49.35	51339	0.63
16	50.30	391624	4.80
17	51.25	218364	2.67
18	52.35	161351	1.98
19	53.35	648041	7.94
20	54.35	1324180	16.22
21	55.30	2735427	33.50
22	56.25	2128768	26.07
23	57.25	223164	2.73
24	58.25	25884	0.32
25	59.25	9704	0.12
26	60.10	5712	0.07
27	61.15	26672	0.33
28	62.15	215227	2.64
29	63.15	1111527	13.61
30	64.10	1584100	19.40
31	65.05	609317	7.46
32	66.15	168263	2.06
33	67.15	1193977	14.62
34	68.15	671385	8.22
35	69.10	1653327	20.25
36	70.05	487954	5.98
37	71.05	211817	2.59
38	72.05	26936	0.33
39	73.15	14272	0.17
40	74.15	201504	2.47
41	75.15	1404021	17.20
42	76.05	720895	8.83
43	77.05	299346	3.67
44	78.15	82346	1.01
45	79.15	410197	5.02
46	80.15	229745	2.81
47	81.15	1274859	15.61
48	82.15	2677139	32.79
49	83.05	685279	8.39
50	84.05	191042	2.34
51	85.05	27368	0.34
52	86.05	33936	0.42
53	86.95	8447	0.10
54	88.15	14406	0.18
55	89.15	27845	0.34
56	90.15	92788	1.14
57	91.15	229540	2.81
58	92.05	1309434	16.04
59	93.05	251070	3.08
60	94.15	1031180	12.63
61	95.05	7777561	95.26
62	96.05	1075689	13.18
63	97.15	443352	5.43
64	98.15	5791591	70.94
65	99.05	853367	10.45
66	101.00	25836	0.32
67	101.95	12549	0.15
68	103.05	34174	0.42
69	103.95	28097	0.34
70	105.05	25495	0.31
71	106.05	41504	0.51
72	107.05	85392	1.05
73	108.05	94416	1.16
74	109.00	123424	1.51
75	109.95	27850	0.34
76	111.05	21167	0.26
77	112.05	33951	0.42
78	113.00	80976	0.99
79	114.00	30352	0.37
80	115.00	103073	1.26
81	115.95	40327	0.49
82	117.15	46817	0.57
83	118.05	76822	0.94
84	119.05	28287	0.35
85	120.05	13305	0.16
86	121.05	15514	0.19
87	122.15	10868	0.13
88	123.10	35343	0.43
89	124.05	22240	0.27
90	125.05	11227	0.14
91	126.00	4660	0.06
92	127.05	18019	0.22
93	128.05	15444	0.19
94	129.05	17040	0.21
95	130.05	108763	1.33
96	130.95	68961	0.84
97	132.05	71445	0.88
98	133.00	136838	1.68
99	133.95	46607	0.57
100	134.95	10400	0.13
101	136.05	2948	0.04
102	137.00	12991	0.16
103	138.05	3684	0.05
104	139.05	34847	0.43
105	140.00	83609	1.02
106	140.95	50652	0.62
107	141.85	34942	0.43
108	142.75	18460	0.23
109	143.75	11732	0.14
110	145.15	9551	0.12
111	146.10	28881	0.35
112	147.05	8766	0.11
113	148.05	19953	0.24
114	148.95	85900	1.05
115	149.95	25166	0.31
116	150.95	3018	0.04
117	151.95	2657	0.03
118	152.90	8998	0.11
119	154.15	2957	0.04
120	155.15	13512	0.17
121	156.05	74583	0.91
122	157.05	28164	0.34
123	157.95	20053	0.25
124	159.05	7380	0.09
125	160.05	495120	6.06
126	161.00	7696194	94.26
127	161.95	1573685	19.27
128	162.95	56294	0.69
129	163.95	1293	0.02
130	165.00	14725	0.18
131	165.95	1730	0.02
132	166.80	3096	0.04

#	m/z	Abs. Int.	Rel. Int.
133	168.15	1530	0.02
134	169.10	5021	0.06
135	170.05	3789	0.05
136	171.05	1027	0.01
137	172.15	2068	0.03
138	173.15	6332	0.08
139	174.15	27224	0.33
140	175.15	220015	2.69
141	176.10	830782	10.18
142	177.05	656240	8.04
143	178.05	84130	1.03
144	179.05	4684	0.06
145	180.05	9308	0.11
146	180.95	76165	0.93
147	181.95	13959	0.17
148	182.90	7550	0.09
149	183.85	2677	0.03
150	184.85	1650	0.02
151	186.00	280	0.00
152	187.05	3711	0.05
153	188.05	22552	0.28
154	189.05	26552	0.33
155	190.00	59474	0.73
156	190.95	10898	0.13
157	192.05	11599	0.14
158	193.00	25951	0.32
159	194.05	16124	0.20
160	195.00	88461	1.08
161	195.95	15076	0.18
162	196.95	4706	0.06
163	197.95	863	0.01
164	199.05	3370	0.04
165	200.05	3364	0.04
166	201.05	27935	0.34
167	202.05	231153	2.83
168	203.00	822594	10.08
169	203.95	127426	1.56
170	205.95	1480	0.02
171	206.95	3205	0.04
172	207.95	36316	0.44
173	208.95	525059	6.43
174	209.85	176793	2.17
175	210.85	47425	0.58
176	211.85	7342	0.09
177	213.05	23581	0.29
178	213.95	22667	0.28
179	215.05	12180	0.15
180	216.05	207662	2.54
181	216.95	77097	0.94
182	217.95	7056	0.09
183	219.95	925	0.01
184	221.05	2562	0.03
185	222.05	7307	0.09
186	224.05	1187358	14.54
187	224.95	8164427	100.00
188	225.95	1172690	14.36
189	226.80	565739	6.93
190	227.75	134722	1.65
191	228.75	5450	0.07
192	230.00	19698	0.24
193	230.95	4838	0.06
194	232.05	17140	0.21
195	233.05	4163	0.05
196	233.95	5970	0.07
197	234.95	572	0.01
198	237.05	36484	0.45
199	238.05	635196	7.78
200	238.95	123504	1.51
201	239.95	30199	0.37
202	241.05	73910	0.91
203	241.95	646142	7.91
204	242.95	63888	0.78
205	243.95	27259	0.33
206	244.95	2941	0.04
207	246.00	185	0.00
208	247.00	217	0.00
209	248.05	1570	0.02
210	249.00	381	0.00
211	249.90	265	0.00
212	250.95	979	0.01
213	251.95	3567	0.04
214	252.95	32030	0.39
215	253.95	455853	5.58
216	254.85	80394	0.98
217	255.85	24731	0.30
218	256.85	9460	0.12

#	m/z	Abs. Int.	Rel. Int.
219	258.05	22636	0.28
220	259.00	25350	0.31
221	259.95	5766	0.07
222	260.95	1252	0.02
223	264.90	41	0.00
224	265.95	4806	0.06
225	266.90	47695	0.58
226	267.85	15466	0.19
227	268.85	5033	0.06
228	269.85	536	0.01
229	270.60	909	0.01
230	271.60	362	0.00
231	272.60	129	0.00
232	273.60	152	0.00
233	274.60	23	0.00
234	275.95	1173	0.01
235	276.95	3007	0.04
236	278.95	968683	11.86
237	279.95	8073694	98.89
238	280.85	2377719	29.12
239	281.85	485522	5.95
240	282.85	83970	1.03
241	283.85	2958	0.04
242	284.85	1519	0.02
243	286.90	20	0.00
244	287.90	224	0.00
245	288.90	360	0.00
246	289.85	3152	0.04
247	290.95	551	0.01
248	292.95	56243	0.69
249	293.95	870526	10.66
250	294.85	177266	2.17
251	295.85	40221	0.49
252	296.85	6786	0.08
253	297.85	727	0.01
254	303.05	890	0.01
255	303.95	24861	0.30
256	304.95	5187	0.06
257	305.95	2947	0.04
258	306.95	462	0.01
259	308.15	2226	0.03
260	309.10	176	0.00
261	310.10	384	0.00
262	311.10	109	0.00
263	312.10	306	0.00
264	314.10	29	0.00
265	315.10	60	0.00
266	316.10	201	0.00
267	317.10	95	0.00
268	318.10	10	0.00
269	319.95	1848	0.02
270	321.95	285094	3.49
271	322.90	3593749	44.02
272	323.85	778911	9.54
273	324.85	165992	2.03
274	325.85	40303	0.49
275	326.85	827	0.01
276	327.85	768	0.01
277	328.90	71	0.00
278	329.90	190	0.00
279	330.90	161	0.00
280	331.90	129	0.00
281	332.90	297	0.00
282	333.90	106	0.00
283	335.50	1183	0.01
284	337.50	150	0.00
285	346.50	59	0.00
286	347.50	22	0.00
287	348.50	116	0.00
288	350.50	192	0.00
289	351.50	135	0.00
290	352.50	6	0.00
291	353.50	106	0.00
292	354.50	186	0.00
293	355.50	91	0.00
294	369.50	146	0.00
295	370.50	69	0.00
296	371.50	284	0.00
297	372.50	160	0.00
298	373.50	118	0.00
299	374.50	148	0.00
300	376.50	96	0.00
301	377.50	29	0.00
302	379.50	425	0.01
303	386.50	146	0.00
304	388.50	67	0.00

#	m/z	Abs. Int.	Rel. Int.
305	389.50	9	0.00
306	390.50	42	0.00
307	391.50	267	0.00
308	392.50	223	0.00
309	393.50	163	0.00
310	394.50	120	0.00
311	395.50	38	0.00
312	396.50	120	0.00
313	398.50	138	0.00
314	400.50	32	0.00
315	402.00	3373	0.04
316	403.00	411	0.01
317	404.00	78	0.00
318	405.00	137	0.00
319	407.00	111	0.00
320	408.00	89	0.00
321	409.00	36	0.00
322	410.00	203	0.00
323	411.00	158	0.00
324	412.00	167	0.00
325	413.00	77	0.00
326	414.00	203	0.00
327	415.00	62	0.00
328	416.00	183	0.00
329	417.00	111	0.00
330	419.00	10	0.00
331	427.00	85	0.00
332	429.00	100	0.00
333	430.00	70	0.00
334	431.00	356	0.00
335	432.00	286	0.00
336	434.00	170	0.00
337	435.00	66	0.00
338	436.00	255	0.00
339	447.00	167	0.00
340	449.00	55	0.00
341	450.00	110	0.00
342	451.00	346	0.00
343	453.00	264	0.00
344	454.00	57	0.00
345	455.00	88	0.00
346	456.00	214	0.00
347	457.00	20	0.00
348	465.05	918	0.01
349	466.00	3230	0.04
350	466.95	2951	0.04
351	468.05	1685	0.02
352	469.00	96	0.00
353	470.00	146	0.00
354	471.00	343	0.00
355	472.00	89	0.00
356	473.00	84	0.00
357	475.00	233	0.00
358	477.00	7	0.00
359	479.10	1120	0.01
360	484.10	769	0.01
361	489.10	110	0.00
362	490.10	197	0.00
363	491.10	192	0.00
364	492.10	135	0.00
365	493.10	130	0.00
366	494.10	39	0.00
367	495.10	62	0.00
368	496.10	51	0.00
369	497.10	104	0.00
370	498.10	21	0.00
371	502.10	2	0.00
372	507.10	93	0.00
373	508.10	75	0.00
374	509.10	4	0.00
375	510.10	132	0.00
376	511.10	25	0.00
377	512.10	331	0.00
378	513.10	36	0.00
379	515.10	171	0.00
380	517.10	20	0.00
381	526.10	284	0.00
382	527.10	40	0.00
383	529.10	149	0.00
384	530.10	75	0.00
385	531.10	131	0.00
386	532.10	153	0.00
387	533.10	12	0.00
388	534.10	228	0.00
389	538.10	11	0.00
390	549.10	137	0.00

Elemental Composition Report

Single Mass Analysis
Tolerance = 5.0 PPM / DBE: min = -50.0, max = 80.0
Element prediction: Off
Number of isotope peaks used for i-FIT = 6

Monoisotopic Mass, Odd and Even Electron Ions
1751 formula(e) evaluated with 26 results within limits (up to 50 closest results for each mass)
Elements Used:
C: 0-15 H: 0-25 N: 0-5 O: 0-5 F: 0-8 S: 0-2
190716_3010 17 (0.197) Cm (16:20-(27:72+2:13)x2.000)
1: TOF MS AP+

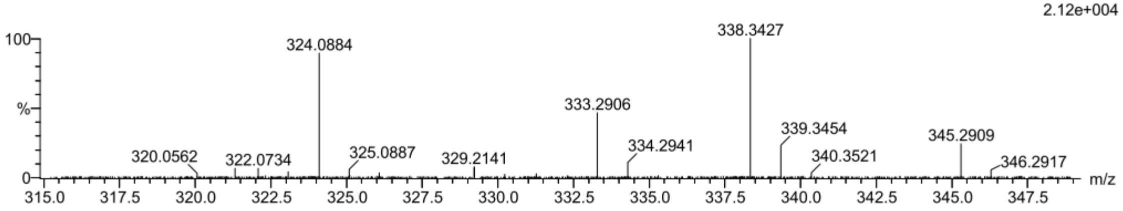

Minimum:				-50.0				
Maximum:		15.0	5.0	80.0				
Mass	Calc. Mass	mDa	PPM	DBE	i-FIT	Norm	Conf(%)	Formula
324.0884	324.0884	0.0	0.0	6.5	936.6	9.605	0.01	C11 H11 N5 O F5
	324.0881	0.3	0.9	4.5	935.2	8.262	0.03	C13 H17 N O3 F3 S
	324.0880	0.4	1.2	-6.5	927.2	0.203	81.65	C5 H18 N O3 F8 S
	324.0879	0.5	1.5	1.0	932.2	5.184	0.56	C8 H16 N4 O3 F4 S
	324.0889	-0.5	-1.5	-7.0	938.0	10.998	0.00	C3 H19 N4 O F7 S2
	324.0879	0.5	1.5	0.5	939.0	12.031	0.00	C11 H19 N F5 S2
	324.0879	0.5	1.5	8.0	940.2	13.190	0.00	C14 H17 N4 F S2
	324.0890	-0.6	-1.9	4.0	939.4	12.465	0.00	C11 H18 N4 O F2 S2
	324.0890	-0.6	-1.9	-3.5	938.2	11.254	0.00	C8 H20 N O F6 S2
	324.0877	0.7	2.2	-1.0	938.9	11.918	0.00	C10 H22 O5 F2 S2
	324.0891	-0.7	-2.2	-3.0	929.1	2.110	12.12	C5 H17 N4 O4 F5 S
	324.0877	0.7	2.2	-3.0	938.1	11.171	0.00	C6 H18 N4 F6 S2
	324.0892	-0.8	-2.5	8.0	936.1	9.147	0.01	C13 H16 N4 O4 S
	324.0875	0.9	2.8	-12.0	937.8	10.817	0.00	C2 H23 O5 F7 S2
	324.0875	0.9	2.8	-4.5	938.4	11.471	0.00	C5 H21 N3 O5 F3 S2
	324.0893	-0.9	-2.8	0.5	933.2	6.266	0.19	C10 H18 N O4 F4 S
	324.0895	-1.1	-3.4	2.5	933.6	6.660	0.13	C8 H12 N5 O2 F6
	324.0873	1.1	3.4	3.0	937.5	10.562	0.00	C11 H12 N2 F8
	324.0872	1.2	3.7	10.5	939.1	12.152	0.00	C14 H10 N5 F4
	324.0897	-1.3	-4.0	6.0	939.0	11.979	0.00	C13 H13 N2 O2 F5
	324.0870	1.4	4.3	1.5	936.3	9.278	0.01	C10 H15 N O5 F5
	324.0870	1.4	4.3	9.0	938.5	11.572	0.00	C13 H13 N4 O5 F
	324.0900	-1.6	-4.9	-11.0	939.6	12.609	0.00	H20 N4 O2 F8 S2
	324.0868	1.6	4.9	5.0	934.8	7.794	0.04	C11 H15 N4 O2 F3 S
	324.0868	1.6	4.9	-2.0	930.4	3.441	3.20	C5 H14 N4 O5 F6
	324.0868	1.6	4.9	-2.5	930.9	3.892	2.04	C8 H17 N O2 F7 S

Sample Information

Sample Name	: Satenik_M
Sample ID	: IVA_1896
Analyzed	: 2019-03-07 13:20:55
Vial #	: 12
Injection Volume	: 0.10
$EndIf$Data File	: C:\GCMSsolution\Data\widma\2019\03_2019\Sa
Method File	: C:\GCMSsolution\System\Tune1\DI EI 1 metoda
Tuning File	: C:\GCMSsolution\System\Tune1\tuning28.02.20

Method

[Comment]

═══ Analytical Line 1 ═══

[GC-2010]

Column Oven Temp.	:30.0 °C
Injection Temp.	:150.00 °C
Injection Mode	:Split
Pressure	:12.8 kPa
Total Flow	:6.6 mL/min
Column Flow	:0.60 mL/min
Linear Velocity	:27.9 cm/sec
Purge Flow	:3.0 mL/min
Split Ratio	:5.0

[GCMS-QP2010 Ultra]

IonSourceTemp	:200.00 °C
Interface Temp.	:200.00 °C
Solvent Cut Time	:0.10 min
Detector Gain Mode	:Absolute
Detector Gain	:1.27 kV
Threshold	:0

[MS Table]

--Group 1 - Event 1--

Start Time	:0.10min
End Time	:25.00min
ACQ Mode	:Scan
Event Time	:0.10sec
Scan Speed	:20000
Start m/z	:35.00
End m/z	:1090.00

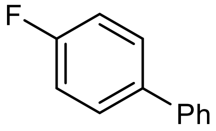

4-fluoro-1,1'-biphenyl

Chemical Formula: $C_{190}H_{158}F_{42}N_{16}O_{31}S_3$

Molecular Weight: 4055.55

Compound **5**

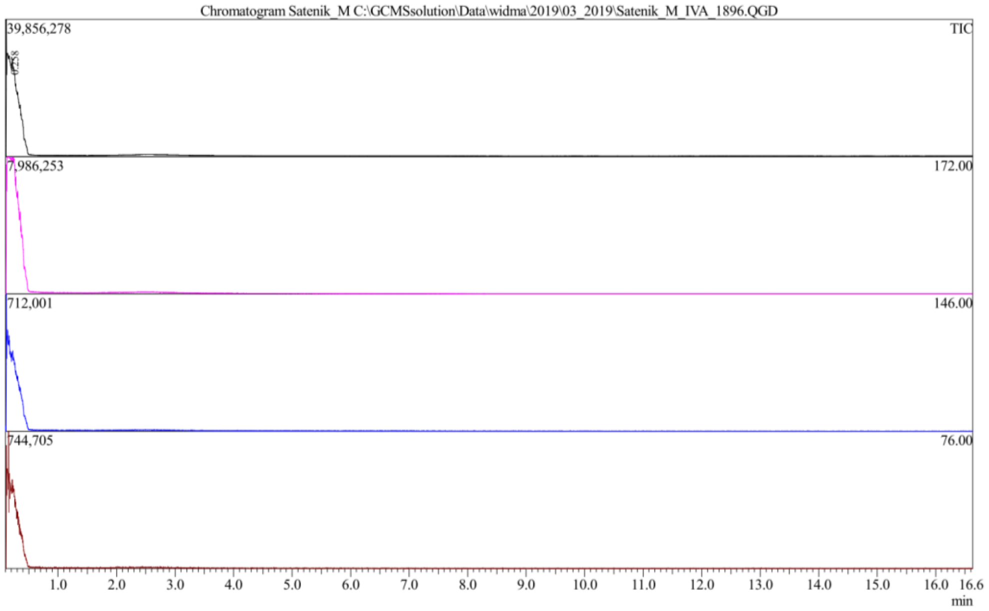

Spectrum

Line#:1 R.Time:0.258(Scan#:96)
MassPeaks:638
RawMode:Single 0.258(96) BasePeak:172(7461971)
BG Mode:None Group 1 - Event 1 Scan

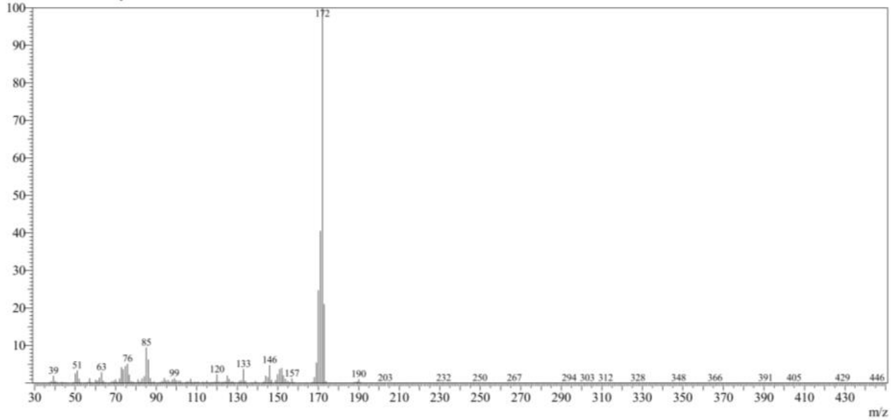

Mass Table
Line#:1 R.Time:0.258(Scan#:96)
MassPeaks:638
RawMode:Single 0.258(96) BasePeak:172(7461971)
BG Mode:None Group 1 - Event 1 Scan

#	m/z	Abs. Int.	Rel. Int.	#	m/z	Abs. Int.	Rel. Int.	#	m/z	Abs. Int.	Rel. Int.
1	35.20	306	0.00	45	78.75	25711	0.34	89	123.05	24575	0.33
2	36.25	1810	0.02	46	80.05	15148	0.20	90	124.15	37856	0.51
3	37.25	12277	0.16	47	81.00	73153	0.98	91	125.05	149218	2.00
4	38.25	34993	0.47	48	82.15	25756	0.35	92	126.05	81419	1.09
5	39.25	141126	1.89	49	83.05	88646	1.19	93	127.05	30328	0.41
6	40.15	37740	0.51	50	84.15	133070	1.78	94	127.90	24715	0.33
7	41.20	16057	0.22	51	85.10	703130	9.42	95	128.85	12797	0.17
8	42.35	5431	0.07	52	86.05	467604	6.27	96	130.05	7012	0.09
9	43.30	17369	0.23	53	87.05	99671	1.34	97	131.05	41578	0.56
10	44.25	11825	0.16	54	88.05	15629	0.21	98	132.05	55476	0.74
11	45.25	6885	0.09	55	88.95	30125	0.40	99	133.00	277362	3.72
12	46.25	2838	0.04	56	89.95	5690	0.08	100	133.95	44770	0.60
13	47.00	762	0.01	57	91.05	9922	0.13	101	134.95	2544	0.03
14	48.05	1418	0.02	58	92.05	22347	0.30	102	136.05	1515	0.02
15	49.05	15589	0.21	59	93.05	40237	0.54	103	137.05	7228	0.10
16	50.05	191380	2.56	60	94.00	96990	1.30	104	138.05	8009	0.11
17	51.00	248181	3.33	61	94.95	49085	0.66	105	139.00	40691	0.55
18	51.95	84504	1.13	62	95.95	56570	0.76	106	139.95	9237	0.12
19	52.95	7111	0.10	63	96.90	20338	0.27	107	140.95	2988	0.04
20	54.15	2358	0.03	64	97.95	66688	0.89	108	142.05	4207	0.06
21	55.15	10958	0.15	65	98.95	93581	1.25	109	143.05	30142	0.40
22	56.15	19854	0.27	66	99.85	54700	0.73	110	144.05	142580	1.91
23	57.10	94629	1.27	67	101.00	46106	0.62	111	145.05	114870	1.54
24	58.05	13199	0.18	68	101.95	42508	0.57	112	146.00	356910	4.78
25	59.05	11004	0.15	69	102.95	7018	0.09	113	146.95	64364	0.86
26	60.05	64292	0.86	70	103.95	8078	0.11	114	147.90	2297	0.03
27	61.05	48342	0.65	71	104.95	31161	0.42	115	148.95	56500	0.76
28	62.05	106991	1.43	72	105.95	29945	0.40	116	149.95	174260	2.34
29	63.00	212745	2.85	73	106.95	78058	1.05	117	150.95	274843	3.68
30	63.95	43040	0.58	74	107.85	16369	0.22	118	151.90	299754	4.02
31	64.95	13813	0.19	75	109.05	26639	0.36	119	152.85	151489	2.03
32	65.95	4825	0.06	76	110.05	23224	0.31	120	153.85	78840	1.06
33	66.95	6668	0.09	77	111.05	21037	0.28	121	154.85	40837	0.55
34	67.95	29722	0.40	78	112.05	6338	0.08	122	156.05	16656	0.22
35	68.95	42279	0.57	79	112.95	27144	0.36	123	157.05	91219	1.22
36	69.95	73257	0.98	80	114.05	12102	0.16	124	157.95	17732	0.24
37	70.85	22913	0.31	81	115.00	43063	0.58	125	158.95	879	0.01
38	71.85	87703	1.18	82	115.95	13027	0.17	126	160.00	229	0.00
39	72.85	318408	4.27	83	117.05	13855	0.19	127	161.00	209	0.00
40	73.75	269788	3.62	84	117.95	18384	0.25	128	162.15	1220	0.02
41	74.85	337423	4.52	85	119.05	29004	0.39	129	163.15	1265	0.02
42	75.80	380959	5.11	86	120.00	168597	2.26	130	164.15	3636	0.05
43	76.75	166260	2.23	87	120.95	32763	0.44	131	165.05	1123	0.02
44	77.75	37820	0.51	88	122.15	18921	0.25	132	166.05	1321	0.02

#	m/z	Abs. Int.	Rel. Int.	#	m/z	Abs. Int.	Rel. Int.	#	m/z	Abs. Int.	Rel. Int.
133	167.05	17239	0.23	219	273.80	133	0.00	305	414.80	29	0.00
134	168.05	116437	1.56	220	274.80	65	0.00	306	423.80	52	0.00
135	169.05	407296	5.46	221	275.80	161	0.00	307	424.80	43	0.00
136	170.05	1844687	24.72	222	276.80	23	0.00	308	425.80	52	0.00
137	171.05	3023838	40.52	223	280.80	208	0.00	309	426.80	176	0.00
138	172.00	7461971	100.00	224	281.80	17	0.00	310	427.80	111	0.00
139	172.95	1566652	21.00	225	282.80	130	0.00	311	428.80	227	0.00
140	173.95	36537	0.49	226	284.80	65	0.00	312	429.80	194	0.00
141	175.95	454	0.01	227	285.80	92	0.00	313	430.80	147	0.00
142	176.50	913	0.01	228	286.80	293	0.00	314	431.80	111	0.00
143	177.85	956	0.01	229	287.80	339	0.00	315	432.80	92	0.00
144	178.90	516	0.01	230	288.80	149	0.00	316	433.80	119	0.00
145	179.90	473	0.01	231	289.80	268	0.00	317	434.80	35	0.00
146	180.90	518	0.01	232	290.80	163	0.00	318	443.80	41	0.00
147	181.90	351	0.00	233	291.80	226	0.00	319	445.80	293	0.00
148	182.90	235	0.00	234	292.80	25	0.00	320	446.80	168	0.00
149	183.90	230	0.00	235	293.80	528	0.01	321	447.80	151	0.00
150	184.90	380	0.01	236	294.80	22	0.00	322	448.80	53	0.00
151	186.05	1096	0.01	237	295.80	96	0.00	323	449.80	288	0.00
152	187.05	3743	0.05	238	296.80	1	0.00	324	450.80	152	0.00
153	188.05	16444	0.22	239	297.80	76	0.00	325	451.80	217	0.00
154	189.05	27746	0.37	240	302.80	308	0.00	326	452.80	115	0.00
155	189.95	76710	1.03	241	304.80	186	0.00	327	454.80	13	0.00
156	190.95	17009	0.23	242	305.80	293	0.00	328	455.80	102	0.00
157	191.95	455	0.01	243	307.80	298	0.00	329	464.80	130	0.00
158	193.10	878	0.01	244	308.80	94	0.00	330	465.80	63	0.00
159	194.10	244	0.00	245	309.80	297	0.00	331	466.80	78	0.00
160	195.10	374	0.01	246	310.80	98	0.00	332	467.80	247	0.00
161	196.10	38	0.00	247	311.80	400	0.01	333	468.80	186	0.00
162	197.10	189	0.00	248	312.80	14	0.00	334	469.80	184	0.00
163	198.10	234	0.00	249	313.80	10	0.00	335	470.80	111	0.00
164	200.10	175	0.00	250	314.80	176	0.00	336	471.80	132	0.00
165	201.10	640	0.01	251	319.80	74	0.00	337	472.80	99	0.00
166	202.10	470	0.01	252	320.80	288	0.00	338	473.80	78	0.00
167	203.10	789	0.01	253	325.80	39	0.00	339	474.80	122	0.00
168	205.10	296	0.00	254	326.80	48	0.00	340	484.80	250	0.00
169	206.10	340	0.00	255	327.80	317	0.00	341	486.80	195	0.00
170	207.10	394	0.01	256	328.80	73	0.00	342	487.80	32	0.00
171	208.10	306	0.00	257	329.80	258	0.00	343	488.80	333	0.00
172	209.10	532	0.01	258	330.80	51	0.00	344	489.80	135	0.00
173	210.10	582	0.01	259	331.80	228	0.00	345	490.80	321	0.00
174	211.10	183	0.00	260	332.80	42	0.00	346	491.80	25	0.00
175	212.10	441	0.01	261	333.80	315	0.00	347	492.80	54	0.00
176	213.10	241	0.00	262	335.80	35	0.00	348	493.80	93	0.00
177	214.10	115	0.00	263	343.80	22	0.00	349	494.80	80	0.00
178	215.10	507	0.01	264	345.80	269	0.00	350	504.80	180	0.00
179	216.10	98	0.00	265	346.80	127	0.00	351	505.80	221	0.00
180	217.10	296	0.00	266	347.80	296	0.00	352	506.80	158	0.00
181	218.10	129	0.00	267	348.80	125	0.00	353	507.80	28	0.00
182	219.10	184	0.00	268	349.80	80	0.00	354	508.80	116	0.00
183	222.10	30	0.00	269	350.80	277	0.00	355	509.80	140	0.00
184	223.10	111	0.00	270	351.80	197	0.00	356	510.80	106	0.00
185	224.10	120	0.00	271	352.80	153	0.00	357	511.80	288	0.00
186	225.10	203	0.00	272	354.80	67	0.00	358	512.80	50	0.00
187	226.10	188	0.00	273	364.80	46	0.00	359	524.80	156	0.00
188	227.10	699	0.01	274	365.80	329	0.00	360	525.80	94	0.00
189	228.10	358	0.00	275	366.80	164	0.00	361	526.80	181	0.00
190	229.10	671	0.01	276	367.80	80	0.00	362	527.80	65	0.00
191	230.10	344	0.00	277	368.80	197	0.00	363	528.80	353	0.00
192	230.80	348	0.00	278	369.80	149	0.00	364	529.80	126	0.00
193	231.85	931	0.01	279	370.80	149	0.00	365	530.80	152	0.00
194	232.80	591	0.01	280	371.80	66	0.00	366	531.80	145	0.00
195	233.80	506	0.01	281	372.80	283	0.00	367	532.80	73	0.00
196	234.80	277	0.00	282	373.80	35	0.00	368	533.80	11	0.00
197	235.80	354	0.00	283	374.80	34	0.00	369	543.80	114	0.00
198	244.80	282	0.00	284	377.80	22	0.00	370	544.80	41	0.00
199	245.80	229	0.00	285	383.80	176	0.00	371	545.80	110	0.00
200	246.80	287	0.00	286	385.80	58	0.00	372	547.80	193	0.00
201	248.80	524	0.01	287	386.80	145	0.00	373	548.80	316	0.00
202	249.80	791	0.01	288	387.80	194	0.00	374	549.80	242	0.00
203	250.80	749	0.01	289	388.80	151	0.00	375	550.80	16	0.00
204	251.80	213	0.00	290	389.80	127	0.00	376	551.80	166	0.00
205	252.80	85	0.00	291	390.80	259	0.00	377	552.80	165	0.00
206	253.80	439	0.01	292	391.80	134	0.00	378	554.80	24	0.00
207	254.80	26	0.00	293	392.80	30	0.00	379	563.80	72	0.00
208	255.80	547	0.01	294	393.80	30	0.00	380	565.80	116	0.00
209	256.80	82	0.00	295	404.80	155	0.00	381	566.80	231	0.00
210	262.80	161	0.00	296	405.80	130	0.00	382	567.80	111	0.00
211	263.80	490	0.01	297	406.80	197	0.00	383	568.80	162	0.00
212	266.80	640	0.01	298	407.80	71	0.00	384	569.80	284	0.00
213	267.80	551	0.01	299	408.80	331	0.00	385	570.80	91	0.00
214	268.80	251	0.00	300	409.80	58	0.00	386	571.80	103	0.00
215	269.80	504	0.01	301	410.80	334	0.00	387	573.80	45	0.00
216	270.80	397	0.01	302	411.80	75	0.00	388	580.80	8	0.00
217	271.80	206	0.00	303	412.80	123	0.00	389	584.80	263	0.00
218	272.80	559	0.01	304	413.80	85	0.00	390	585.80	47	0.00

Single Mass Analysis

Tolerance = 5.0 PPM / DBE: min = -50.0, max = 80.0
Element prediction: Off
Number of isotope peaks used for i-FIT = 5

Monoisotopic Mass, Odd and Even Electron Ions
16 formula(e) evaluated with 2 results within limits (up to 50 closest results for each mass)
Elements Used:
C: 0-25 H: 0-20 F: 0-8

190311_IVA_1896_APCI_ 16 (0.177) Cm (12:18-(2:8+39:69)x2.000)
1: TOF MS AP+

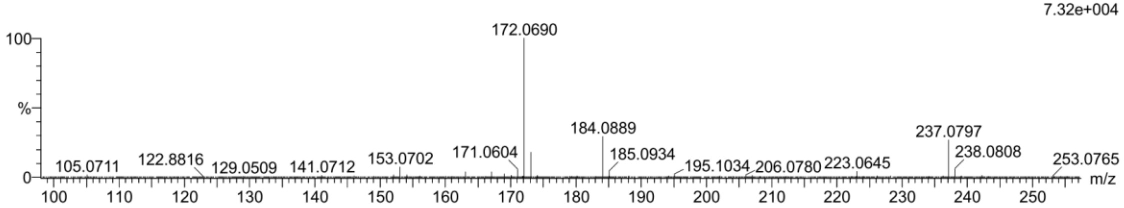

Minimum:				-50.0				
Maximum:		15.0	5.0	80.0				

Mass	Calc. Mass	mDa	PPM	DBE	i-FIT	Norm	Conf(%)	Formula
172.0690	172.0688	0.2	1.2	8.0	1009.9	0.001	99.86	C12 H9 F
	172.0687	0.3	1.7	-3.0	1016.5	6.564	0.14	C4 H10 F6

(E) Computational study

General: Density functional calculations were performed at B3LYP/def2-SVP level of theory using Gaussian 16 software.[1] Tight criteria were used for geometry optimization together with ultrafine integration grid. In stationary points second vibrational frequencies were always calculated, one imaginary frequency was found for transition states and positive definitive Hessian for all other structures. Solvent effects by were included by means of continuum solvation models: PCM and SMD. For transition states and radical species unrestricted Kohn-Sham formalism was used, in all cases leading to small spin contamination values. Special attention was given to discussion of the ionization energies and electron affinities. Here we recalculated all geometries using larger basis set def2-TZVPP.[2] Since different correlation energies between neutral/ionized species has to treated carefully we performed DLPNO-CCSD(T)/def2-TZVPP[3] single point calculations in Orca 5.0 software,[4] using TightPNO settings.[5]
Cartesian coordinates of stationary points are attached.

Literature:

1. Frisch, M. J.; Trucks, G. W.; Schlegel, H. B.; Scuseria, G. E.; Robb, M. A.; Cheeseman, J. R.; Scalmani, G.; Barone, V.; Petersson, G. A.; Nakatsuji, H.; Li, X.; Caricato, M.; Marenich, A. V.; Bloino, J;. Janesko, B. G.; Gomperts, R.; Mennucci, B.; Hratchian, H. P.; Ortiz, J. V.; Izmaylov, A. F.; Sonnenberg, J. L.; Williams-Young, D.; Ding, F.; Lipparini, F.; Egidi, F.; Goings, J.; Peng, B.; Petrone, A.; Henderson, T.; Ranasinghe, D.; Zakrzewski, V. G.; Gao, J.; Rega, N.; Zheng, G.; Liang, W.; Hada, M.; Ehara, M.; Toyota, K.; Fukuda, R.; Hasegawa, J.; Ishida, M.; Nakajima, T.; Honda, Y.; Kitao, O.; Nakai, H.; Vreven, T.; Throssell, K.; Montgomery Jr., J. A. Peralta, J. E.; Ogliaro, F.; Bearpark, M. J.; Heyd, J. J.; Brothers, E. N.; Kudin, K. N.; Staroverov, V. N.; Keith, T. A.; Kobayashi, R.; Normand, J.; Raghavachari, K.; Rendell, A. P.; Burant, J. C.; Iyengar, S. S.; Tomasi, J.; Cossi, M.; Millam, J. M.; Klene, M.; Adamo, C.; Cammi, R.; Ochterski, J. W.; Martin, R. L.; Morokuma, K.; Farkas, O.; Foresman, J. B.; Fox, D. J.; Gaussian, Inc., Wallingford CT, 2016.
2. Weigend, F.; Ahlrichs, R. Balanced basis sets of split valence, triple zeta valence and quadruple zeta valence quality for H to Rn: Design and assessment of accuracy. *Phys. Chem. Chem. Phys.* **2005**, *7*, 3297- 3305.
3. Guo, Y.; Riplinger, C.; Becker, U.; Liakos, D. G.; Minenkov, Y.; Cavallo, L.; Neese, F. An improved linear scaling perturbative triples correction for the domain based local pair-natural orbital based singles and doubles coupled cluster method [DLPNO-CCSD(T)]. *J. Chem. Phys.,* **2018**, *148(1)*, 011101.
4. (a) Neese, F. The ORCA program system" Wiley Interdisciplinary Reviews: Computational Molecular Science, **2012**, *2*, 73-78. (b) Neese, F. Software update: the ORCA program system, version 4.0" Wiley Interdisciplinary Reviews: Computational Molecular Science, **2017**, *8*, e1327.
5. Liakos, D. G.; Sparta, M.; Kesharwani, M. K.; Martin, J. M. L.; Neese, F. *J. Chem. Theory Comput.*, **2015**, *11*, 1525-1539.

Cartesian coordinates

OCF3 minus B3LYP/def2TZVPP in toluene solvent

8	-0.454617000	1.331193000	0.000000000
6	-0.061896000	0.180376000	0.000000000
9	-0.454617000	-0.640019000	1.103519000
9	-0.454617000	-0.640019000	-1.103519000
9	1.354601000	-0.023495000	0.000000000

OCF3 radical B3LYP/def2TZVPP in toluene solvent

8	-0.446598000	1.317112000	0.000000000
6	-0.028102000	0.029502000	0.000000000
9	1.308907000	0.089277000	0.000000000
9	-0.446598000	-0.639856000	1.077154000
9	-0.446598000	-0.639856000	-1.077154000

pyridium cation B3LYP/def2TZVPP in toluene solvent

6	0.000000000	0.000000000	-3.485939000
6	0.983633000	0.697273000	-2.792869000
6	0.990959000	0.701346000	-1.403842000
6	0.000000000	0.000000000	-0.728737000
6	-0.990959000	-0.701346000	-1.403842000
6	-0.983633000	-0.697273000	-2.792869000
1	0.000000000	0.000000000	-4.566971000
1	1.753996000	1.231721000	-3.330565000
1	1.768052000	1.220297000	-0.859562000
1	-1.768052000	-1.220297000	-0.859562000
1	-1.753996000	-1.231721000	-3.330565000
7	0.000000000	0.000000000	0.725067000
6	0.000000000	1.175257000	1.394127000
6	0.000000000	-1.175257000	1.394127000
6	0.007554000	1.198985000	2.771216000
1	-0.023143000	2.067525000	0.789393000
6	-0.007554000	-1.198985000	2.771216000
1	0.023143000	-2.067525000	0.789393000
1	0.005515000	2.151323000	3.279286000
1	-0.005515000	-2.151323000	3.279286000
6	0.000000000	0.000000000	3.474007000
1	0.000000000	0.000000000	4.554833000

pyridium radical B3LYP/def2TZVPP in toluene solvent

6	0.000000000	0.000000000	-3.522216000
6	0.589840000	1.043343000	-2.814796000

6	0.596624000	1.048902000	-1.427814000
6	0.000000000	0.000000000	-0.710413000
6	-0.596624000	-1.048902000	-1.427814000
6	-0.589840000	-1.043343000	-2.814796000
1	0.000000000	0.000000000	-4.603379000
1	1.065112000	1.857699000	-3.346003000
1	1.096068000	1.850704000	-0.904217000
1	-1.096068000	-1.850704000	-0.904217000
1	-1.065112000	-1.857699000	-3.346003000
7	0.000000000	0.000000000	0.695818000
6	0.001965000	1.197266000	1.423920000
6	-0.001965000	-1.197266000	1.423920000
6	0.000000000	1.199614000	2.786167000
1	-0.052778000	2.102018000	0.844575000
6	0.000000000	-1.199614000	2.786167000
1	0.052778000	-2.102018000	0.844575000
1	-0.018325000	2.157940000	3.287175000
1	0.018325000	-2.157940000	3.287175000
6	0.000000000	0.000000000	3.525300000
1	0.000000000	0.000000000	4.603845000

pyridium+OCF3- reactant complex B3LYP/def2SVP in toluene solvent as model for environment

6	-3.303885000	2.612441000	-0.018650000
6	-3.923330000	1.445860000	0.440328000
6	-3.222685000	0.238684000	0.454682000
6	-1.899069000	0.209499000	-0.003435000
6	-1.258243000	1.372197000	-0.446666000
6	-1.975234000	2.571522000	-0.450738000
1	-3.853905000	3.556242000	-0.026459000
1	-4.953028000	1.473325000	0.803445000
1	-3.700380000	-0.660222000	0.848266000
1	-0.206634000	1.359946000	-0.745817000
1	-1.479385000	3.481910000	-0.794240000
7	-1.176710000	-1.054638000	-0.011317000
6	-1.832307000	-2.200173000	-0.350099000
6	0.144833000	-1.080224000	0.302577000
6	-1.181410000	-3.419167000	-0.345863000
1	-2.875147000	-2.095006000	-0.643911000
6	0.841188000	-2.284373000	0.296691000
1	0.660093000	-0.121038000	0.503479000
1	-1.736605000	-4.316345000	-0.622680000
1	1.905956000	-2.240064000	0.527862000
6	0.178925000	-3.469702000	-0.013792000
1	0.712479000	-4.422703000	-0.014396000

8	1.789603000	1.264056000	0.083715000
6	2.981878000	0.926716000	0.026190000
9	3.246544000	-0.408921000	0.466984000
9	3.565272000	0.934202000	-1.237025000
9	3.870711000	1.665195000	0.795219000

pyridium+OCF3- TS1 B3LYP/def2SVP in toluene solvent as model for environment

6	2.457496000	-2.684197000	-0.024249000
6	1.794679000	-2.421631000	-1.232474000
6	0.890071000	-1.374979000	-1.360011000
6	0.600418000	-0.513953000	-0.254959000
6	1.259178000	-0.803629000	0.977522000
6	2.176554000	-1.852873000	1.063225000
1	3.170746000	-3.505888000	0.061457000
1	1.985702000	-3.048977000	-2.108588000
1	0.396826000	-1.183631000	-2.316161000
1	1.081519000	-0.161047000	1.841269000
1	2.682007000	-2.015032000	2.020265000
7	-1.078239000	-0.368466000	-0.054168000
6	-1.627899000	-1.077871000	0.941292000
6	-1.852037000	0.265195000	-0.954993000
6	-3.007819000	-1.164509000	1.090994000
1	-0.918240000	-1.579257000	1.604017000
6	-3.238449000	0.195298000	-0.880800000
1	-1.325975000	0.837285000	-1.717604000
1	-3.423458000	-1.736592000	1.921964000
1	-3.840994000	0.714369000	-1.627864000
6	-3.830343000	-0.524583000	0.161207000
1	-4.917474000	-0.587352000	0.245174000
8	0.736726000	1.047926000	-0.802418000
6	0.942943000	2.036934000	0.027425000
9	0.070137000	2.056850000	1.083082000
9	2.177537000	2.053993000	0.583834000
9	0.793932000	3.221020000	-0.605858000

pyridium+OCF3- main product B3LYP/def2SVP in toluene solvent as model for environment

6	3.193960000	-2.684179000	0.034927000
6	3.890931000	-1.546531000	-0.385248000
6	3.212368000	-0.342898000	-0.599366000
6	1.835212000	-0.301385000	-0.375720000
6	1.117499000	-1.423232000	0.037703000
6	1.811624000	-2.620256000	0.241210000
1	3.728865000	-3.623150000	0.196832000
1	4.969383000	-1.593676000	-0.554868000

1	3.737829000	0.550578000	-0.938875000
1	0.034725000	-1.348738000	0.182065000
1	1.264277000	-3.509669000	0.563234000
7	-2.335933000	-0.869872000	0.106490000
6	-3.387152000	-1.596078000	0.500074000
6	-2.571347000	0.309546000	-0.479159000
6	-4.712414000	-1.183624000	0.329468000
1	-3.167115000	-2.558088000	0.977388000
6	-3.858085000	0.812999000	-0.696214000
1	-1.692064000	0.884700000	-0.789255000
1	-5.534199000	-1.817359000	0.670583000
1	-3.993666000	1.783506000	-1.178836000
6	-4.951770000	0.048979000	-0.282777000
1	-5.973092000	0.407934000	-0.434489000
8	1.116650000	0.878644000	-0.635166000
6	1.209532000	1.904994000	0.231634000
9	0.944856000	1.542579000	1.493610000
9	2.429161000	2.473374000	0.234707000
9	0.326120000	2.830035000	-0.139102000

pyridium+OCF3- TS2 B3LYP/def2SVP in toluene solvent as model for environment

6	5.269678000	-0.439300000	-0.567590000
6	4.385283000	-1.512904000	-0.425456000
6	3.040474000	-1.283910000	-0.123855000
6	2.585863000	0.029287000	0.048928000
6	3.467209000	1.109425000	-0.085554000
6	4.806941000	0.869700000	-0.400264000
1	6.319108000	-0.622345000	-0.809298000
1	4.737849000	-2.537569000	-0.563742000
1	2.341712000	-2.119075000	-0.043390000
1	3.111869000	2.129668000	0.073027000
1	5.494207000	1.712764000	-0.502075000
7	1.202398000	0.266329000	0.367120000
6	0.609124000	-0.391465000	1.415722000
6	0.467089000	1.178847000	-0.349006000
6	-0.704318000	-0.195750000	1.735444000
6	-0.848975000	1.415232000	-0.071906000
1	0.999529000	1.693197000	-1.148939000
1	-1.125503000	-0.716197000	2.595881000
1	-1.397126000	2.147848000	-0.663998000
6	-1.529699000	0.639991000	0.919388000
1	-2.467062000	1.008585000	1.332333000
1	1.264463000	-1.040006000	1.997534000

8	-2.516088000	-0.686330000	-0.149974000
6	-3.712962000	-0.334875000	-0.403592000
9	-4.494493000	-0.099057000	0.718313000
9	-3.825642000	0.848065000	-1.119377000
9	-4.411129000	-1.253031000	-1.131264000

pyridium+OCF3- product2(p- position) B3LYP/def2SVP in toluene solvent as model for environment

6	5.363048000	-0.156722000	-0.491865000
6	4.542367000	-1.276067000	-0.659512000
6	3.171274000	-1.194077000	-0.405334000
6	2.609331000	0.012606000	0.041097000
6	3.434994000	1.134311000	0.219309000
6	4.801408000	1.047530000	-0.056288000
1	6.433542000	-0.222056000	-0.699441000
1	4.966896000	-2.220746000	-1.008229000
1	2.531498000	-2.062430000	-0.574347000
1	3.014185000	2.069332000	0.593778000
1	5.433561000	1.927197000	0.088106000
7	1.211545000	0.091093000	0.313262000
6	0.561480000	-0.917957000	1.008172000
6	0.464096000	1.200717000	-0.052794000
6	-0.761185000	-0.887260000	1.281724000
6	-0.861934000	1.303038000	0.184949000
1	1.018760000	1.996055000	-0.551100000
1	-1.196693000	-1.689018000	1.880499000
1	-1.385435000	2.211548000	-0.116814000
6	-1.650788000	0.180941000	0.759327000
1	-2.387313000	0.511031000	1.506574000
1	1.204303000	-1.717768000	1.377526000
8	-2.501497000	-0.440220000	-0.347105000
6	-3.784672000	-0.117289000	-0.377998000
9	-4.460576000	-0.502206000	0.732972000
9	-3.997381000	1.218583000	-0.486910000
9	-4.368560000	-0.705005000	-1.425759000

pyridium+OCF3- TS3 B3LYP/def2SVP in toluene solvent as model for environment

6	-3.945396000	-1.488557000	-0.085593000
6	-2.671019000	-2.045438000	0.053205000
6	-1.537257000	-1.229824000	0.103881000
6	-1.682565000	0.160559000	0.001614000
6	-2.958463000	0.724089000	-0.153394000
6	-4.084337000	-0.100996000	-0.187693000
1	-4.826978000	-2.132948000	-0.118517000
1	-2.551365000	-3.128301000	0.135982000

1	-0.549531000	-1.667985000	0.235045000
1	-3.070196000	1.803725000	-0.271051000
1	-5.073789000	0.345695000	-0.311470000
7	-0.534815000	1.026178000	0.036508000
6	0.639867000	0.658091000	-0.598033000
6	-0.596417000	2.209899000	0.748225000
6	1.554765000	1.732640000	-0.918489000
6	0.367308000	3.165609000	0.624106000
1	-1.467597000	2.325916000	1.392173000
1	2.357981000	1.517530000	-1.622616000
1	0.289672000	4.090780000	1.195286000
6	1.439224000	2.938593000	-0.292451000
1	2.156873000	3.736528000	-0.498130000
1	0.503631000	-0.133160000	-1.336820000
8	1.504600000	-0.340451000	0.627484000
6	2.301165000	-1.245983000	0.169933000
9	3.226610000	-0.783872000	-0.732648000
9	1.653423000	-2.258833000	-0.511630000
9	3.006295000	-1.856131000	1.147925000

pyridium+OCF3- product2(o- position) B3LYP/def2SVP in toluene solvent as model for environment

6	4.016087000	-1.381824000	0.116631000
6	2.763098000	-1.998065000	0.060161000
6	1.594219000	-1.236171000	-0.014844000
6	1.669723000	0.166786000	-0.016791000
6	2.929936000	0.786262000	0.058059000
6	4.091741000	0.014274000	0.112876000
1	4.926112000	-1.984022000	0.167416000
1	2.687594000	-3.088280000	0.059022000
1	0.630640000	-1.737034000	-0.089365000
1	3.001031000	1.874546000	0.100437000
1	5.062702000	0.512328000	0.172632000
7	0.492361000	0.970329000	-0.077182000
6	-0.717439000	0.523466000	0.519811000
6	0.508261000	2.177716000	-0.759229000
6	-1.580920000	1.639951000	0.979184000
6	-0.458733000	3.115399000	-0.579613000
1	1.352317000	2.323458000	-1.433909000
1	-2.320542000	1.419306000	1.749497000
1	-0.421980000	4.050771000	-1.137968000
6	-1.481371000	2.860892000	0.399595000
1	-2.159104000	3.666339000	0.695055000
1	-0.501227000	-0.210492000	1.305184000
8	-1.467315000	-0.290870000	-0.566546000

6	-2.290336000	-1.247684000	-0.175891000
9	-3.264642000	-0.820874000	0.662746000
9	-1.654466000	-2.250074000	0.495391000
9	-2.884352000	-1.797750000	-1.237589000

Molecule 1e in dioxane - reactants B3LYP/def2SVP

6	2.175550000	-0.615249000	0.067381000
6	1.224861000	0.323340000	-0.337472000
6	-0.121826000	-0.041339000	-0.389605000
6	-0.491157000	-1.330683000	0.006349000
6	0.458218000	-2.257003000	0.459829000
6	1.805432000	-1.897974000	0.478234000
1	1.506354000	1.346683000	-0.592050000
1	-0.869598000	0.701902000	-0.687985000
1	0.162743000	-3.239195000	0.831664000
1	2.565924000	-2.592292000	0.838776000
7	-1.898710000	-1.706015000	-0.023513000
6	-2.247489000	-2.989286000	-0.319677000
6	-2.851916000	-0.771814000	0.239416000
6	-3.571476000	-3.384966000	-0.324113000
1	-1.436431000	-3.669807000	-0.571677000
6	-4.195065000	-1.130277000	0.233196000
1	-2.538474000	0.277855000	0.396888000
1	-3.812995000	-4.420426000	-0.567251000
1	-4.931208000	-0.352553000	0.440674000
6	-4.569162000	-2.445000000	-0.037201000
1	-5.621571000	-2.736662000	-0.039709000
8	-2.393307000	1.984441000	-0.198948000
6	-1.793407000	3.057021000	0.003545000
9	-0.438679000	3.066229000	-0.415307000
9	-2.313983000	4.170686000	-0.639735000
9	-1.700640000	3.462071000	1.330623000
16	3.924340000	-0.153566000	0.138485000
6	4.493049000	-0.312095000	-1.564710000
1	4.372999000	-1.353741000	-1.888599000
1	3.929711000	0.381957000	-2.201482000
1	5.556183000	-0.034009000	-1.536243000
8	4.616681000	-1.178961000	0.936121000
8	4.002118000	1.265836000	0.507148000

Molecule 1e in dioxane - TS B3LYP/def2SVP

6	-2.211609000	-0.023848000	0.119514000
6	-1.545286000	-0.335534000	1.320243000

6	-0.169850000	-0.303327000	1.398235000
6	0.648567000	0.093329000	0.268800000
6	-0.068198000	0.351840000	-0.961247000
6	-1.451137000	0.329501000	-1.005876000
1	-2.128368000	-0.599424000	2.205432000
1	0.331967000	-0.538616000	2.339834000
1	0.497756000	0.657100000	-1.842768000
1	-1.960600000	0.599466000	-1.933752000
7	1.857394000	-0.937957000	0.071160000
6	1.750440000	-1.847607000	-0.914445000
6	2.881080000	-0.993366000	0.950384000
6	2.696325000	-2.852477000	-1.070729000
1	0.875474000	-1.739756000	-1.557657000
6	3.842406000	-1.990635000	0.860617000
1	2.906556000	-0.208115000	1.703171000
1	2.587198000	-3.564672000	-1.889893000
1	4.655236000	-2.011841000	1.587882000
6	3.758051000	-2.933840000	-0.167400000
1	4.507992000	-3.722149000	-0.260975000
8	1.568703000	1.332084000	0.785963000
6	2.125636000	2.173368000	-0.060997000
9	2.778988000	1.546547000	-1.085904000
9	1.259619000	3.024756000	-0.645705000
9	3.042778000	2.923997000	0.576091000
16	-3.973438000	-0.068389000	0.035164000
6	-4.416044000	-1.755168000	-0.451218000
1	-3.987441000	-1.970386000	-1.438523000
1	-4.049309000	-2.458232000	0.307754000
1	-5.514347000	-1.770940000	-0.494507000
8	-4.417879000	0.805000000	-1.068660000
8	-4.516273000	0.119960000	1.394899000

Molecule 4e in dioxane - products B3LYP/def2SVP

6	1.469786000	-0.124454000	-0.108326000
6	0.961236000	-1.426454000	-0.058159000
6	-0.417753000	-1.617334000	-0.048887000
6	-1.270957000	-0.508901000	-0.101999000
6	-0.765110000	0.794225000	-0.174209000
6	0.619034000	0.979950000	-0.177931000
1	1.642457000	-2.278983000	-0.038979000
1	-0.852392000	-2.617441000	-0.008980000
1	-1.423949000	1.658455000	-0.240759000
1	1.038442000	1.984818000	-0.253373000
8	-2.619490000	-0.824406000	-0.098604000

**6**	**-3.590509000**	**0.105913000**	**0.056394000**
**9**	**-3.438991000**	**0.814496000**	**1.182838000**
**9**	**-3.631836000**	**0.976575000**	**-0.962912000**
**9**	**-4.748834000**	**-0.533756000**	**0.108154000**
**16**	**3.252171000**	**0.130795000**	**-0.129150000**
**6**	**3.715196000**	**0.266598000**	**1.608817000**
**1**	**3.201262000**	**1.128462000**	**2.053095000**
**1**	**3.461299000**	**-0.668000000**	**2.125046000**
**1**	**4.803117000**	**0.423696000**	**1.603409000**
**8**	**3.512673000**	**1.436031000**	**-0.757167000**
**8**	**3.875123000**	**-1.093727000**	**-0.656725000**
